# Iminoboronates as Dual‐Purpose Linkers in Chemical Probe Development

**DOI:** 10.1002/chem.202005115

**Published:** 2021-01-14

**Authors:** Antonie J. van der Zouwen, Aike Jeucken, Roy Steneker, Katharina F. Hohmann, Jonas Lohse, Dirk J. Slotboom, Martin D. Witte

**Affiliations:** ^1^ Chemical Biology II Stratingh Institute for Chemistry Nijenborgh 7 9747 AG Groningen The Netherlands; ^2^ Membrane Enzymology Groningen Biomolecular Sciences and Biotechnology Institute 9747 AG Groningen The Netherlands

**Keywords:** affinity-based probes, bio-orthogonal chemistry, chemical probes, iminoboronate, protein labeling

## Abstract

Chemical probes that covalently modify proteins of interest are powerful tools for the research of biological processes. Important in the design of a probe is the choice of reactive group that forms the covalent bond, as it decides the success of a probe. However, choosing the right reactive group is not a simple feat and methodologies for expedient screening of different groups are needed. We herein report a modular approach that allows easy coupling of a reactive group to a ligand. α‐Nucleophile ligands are combined with 2‐formylphenylboronic acid derived reactive groups to form iminoboronate probes that selectively label their target proteins. A transimination reaction on the labeled proteins with an α‐amino hydrazide provides further modification, for example to introduce a fluorophore.

## Graphical abstract

**Iminoboronate chemistry** enables the synthesis of chemical probes that selectively label proteins of interest from 2‐formylphenylboronic acid reactive groups and hydrazide‐functionalized ligands. The reversibility of the iminoboronate linkage is exploited to detect the labeled proteins by ligand‐fluorophore exchange. Remarkably, the fast iminoboronate chemistry even allows probe formation within protein mixtures.

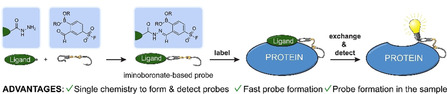

Protein profiling with chemical probes is commonly employed to study biological processes in both fundamental and applied settings.[^1^, ^2^, ^3^] The probes used in these profiling studies generally consist of a ligand that binds to the protein of interest (POI), a reporter group (e.g. bio‐orthogonal handle, fluorophore, affinity tag) that allows visualization and/or enrichment of the modified proteins, and a reactive group that covalently links the probe to the POI.

Over the past decade, many reactive groups have been developed that can be employed for probe development.[^4^, ^5^, ^6^, ^7^] Selecting the appropriate reactive group is essential to obtain highly selective and potent chemical probes. The reactive group determines which residues are being targeted and it can even alter the binding profile of the probe.^[8]^ However, determining the optimal reactive group is, in many cases, not trivial. Often multiple probes have to be synthesized, for example by multi‐component reactions, and these probes then have to be screened against the POI.[^9^, ^10^]

To further simplify the identification of an appropriate reactive group, we started to explore means in which probe synthesis and screening could be performed in one simple operation. Capitalizing on the pioneering work on hydrazone‐containing probes by Hamachi and co‐workers,^[6]^ we established a method to prepare probes by linking different reactive groups to ligands via hydrazone and oxime chemistry.^[11]^ The resulting chemical probes could be used without further purification in protein labeling experiments. The labeled proteins could only be detected reliably by a copper‐catalyzed azide alkyne cycloaddition (CuAAC) reaction, which necessitated the incorporation of a click handle in one of the probe fragments.

The hydrazone linkage, which is introduced during the synthesis of the probe, could serve as an alternative bio‐orthogonal handle. Exchange of the ligand for a fluorophore by transimination of the hydrazone would overcome the need for an additional bio‐orthogonal handle. However, attempts to visualize the labeled proteins in this fashion were unsuccessful in our hands due to inefficient exchange. Recently, chemistries have been reported that undergo exchange more readily. In particular, the iminoboronate chemistry attracted our attention. 2‐Formylphenyl boronic acids (2‐FPBA) and pinacol boronate esters thereof have been shown to form iminoboronates with hydrazides and alkoxyamines rapidly even at low concentrations, and have been utilized in bioconjugation reactions.[^12^, ^13^, ^14^, ^15^, ^16^] The resulting reagents can be transiminated under physiological conditions,[^15^, ^17^] and gel‐stable tricyclic diazaborines (DAB) can be obtained by reacting 2‐FPBAs with α‐amino hydrazides.^[18]^ We hypothesized that iminoboronates could serve as a dual‐purpose linker (Figure 1). Probes might be formed by reacting 2‐FPBA reactive groups, such as sulfonyl fluoride **R1**, with α‐nucleophile ligands. The probe‐modified proteins might be detected by transimination of the iminoboronate linkage with α‐amino hydrazide fluorescein (**FITC am‐zide**) using the stability differences between iminoboronates as the driving force. We here demonstrate the feasibility of this approach.

**Figure 1:**
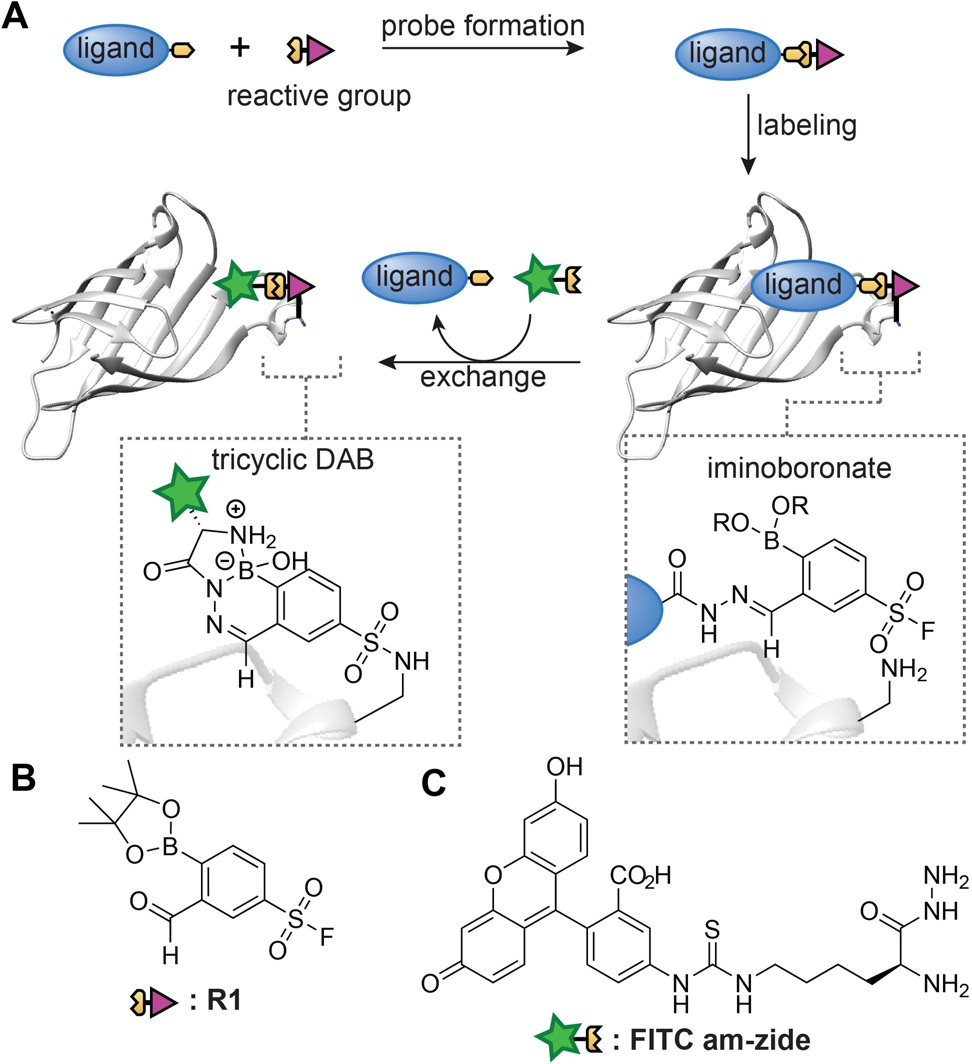
**A**) Schematic representation of our approach. α‐Nucleophile ligands are coupled with 2‐FPBA derived reactive groups to form iminoboronate chemical probes. After labeling the POI with the probe, the ligand is exchanged with an α‐amino hydrazide to introduce a reporter group. **B**, **C**) Structure of sulfonyl fluoride **R1** (**B**) and **FITC am‐zide** (**C**).

In order to determine the suitability of iminoboronate chemistry for probe synthesis and visualization, we synthesized sulfonyl fluoride **R1** and reacted it overnight with an equimolar amount of biotin‐hydrazide ligand **L1** or non‐targeted control hydrazide **C1** (Figure 2 A). This gave the crude probes, which we used to label streptavidin (Strp) against a background of two control proteins, bovine carbonic anhydrase II (CA‐II) and ovalbumin (OVA). To detect the modified proteins, we subjected the samples either to CuAAC with fluorescein alkyne (Lumiprobe B41B0) or to transimination with five equivalents of **FITC am‐zide**. Read‐out by exchange of the iminoboronate linker gave a result that is very similar to CuAAC functionalization of the azido group in ligands **L1** and **C1** (Figure 2 A). Pronounced labeling of the target protein Strp was solely observed in samples containing the biotin‐based probe **L1R1**. Labeling was negligible in samples treated with the control **C1R1**. Small differences between the methods were noticeable. While the fluorescence signal for CA‐II and OVA in the CuAAC samples were comparable to the DMSO control, a small amount of ligand‐independent labeling was visible when transimination was used as read‐out. Nonetheless, these results clearly indicate that tethering of the ligand to the reactive group via an iminoboronate linkage and exchange of the ligand by **FITC am‐zide** are feasible. Labeling experiments on cell lysate of BirA‐overexpressing *E. coli* with ligands **L1–L3**, and on CA‐II with sulfonamide ligand **L4** further confirmed this (Figure 2 B&C).

**Figure 2:**
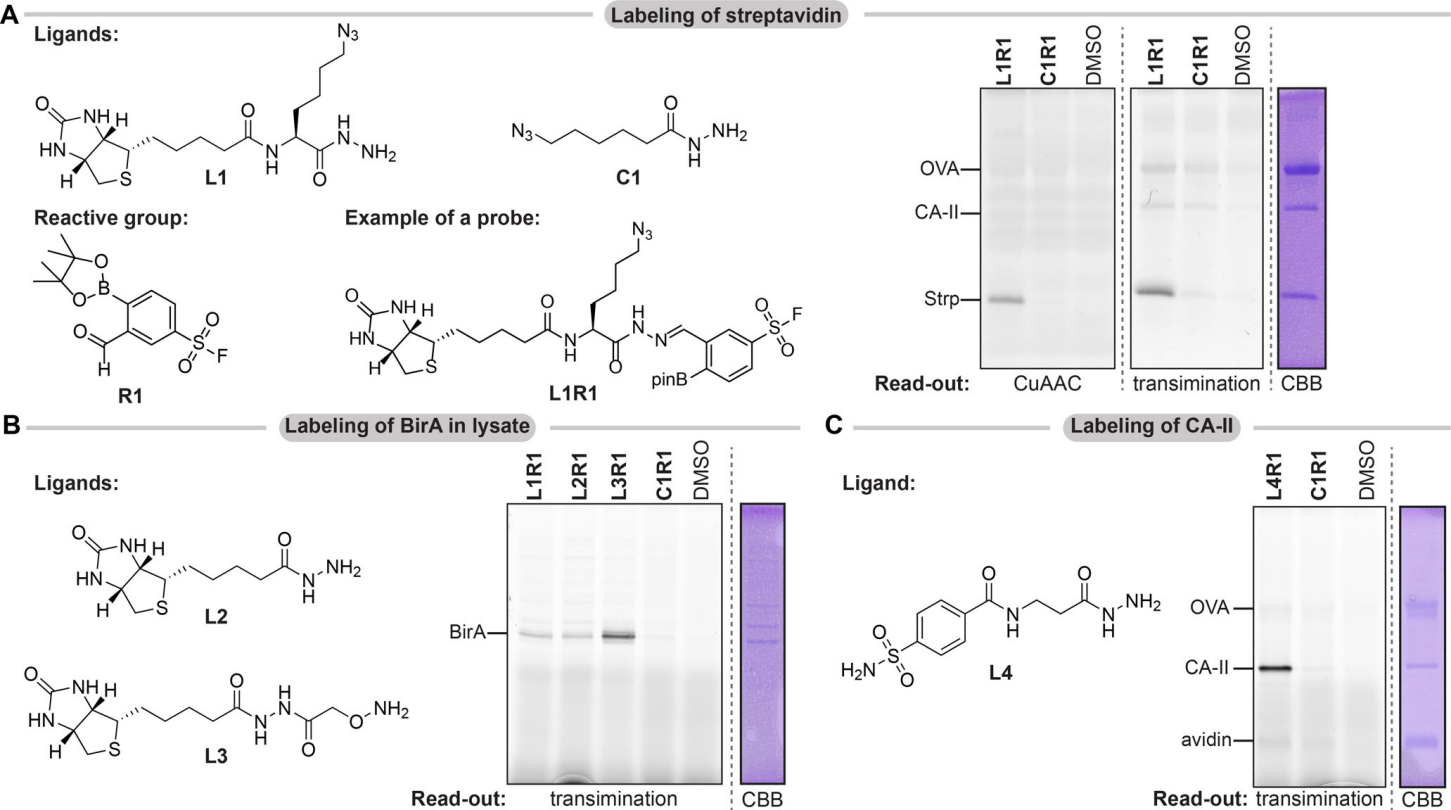
**A**) Proof of concept labeling studies on Strp using biotin ligand **L1**, control **C1** and reactive group **R1**. A mixture of Strp (25 μm), CA‐II (5 μm) and OVA (25 μm) in HEPES (pH 8.2) was incubated with 20 μm probe for 2 h. Read‐out with CuAAC (fluorescein alkyne) or transimination (**FITC am‐zide**). **B**) Labeling of BirA in lysate of BirA‐overexpressing *E. coli* (2.5 mg mL^−1^, HEPES pH 8.2) with probes based on ligands **L1**–**L3**. Incubation with 20 μm probe for 2 h. Read‐out with **FITC am‐zide. C**) Labeling of CA‐II (5 μm) in the presence of OVA (25 μm) and avidin (25 μm) in HEPES (pH 8.2) with probes based on ligand **L4**. Incubation with 20 μm probe for 2 h. Read‐out with **FITC am‐zide**.

The background signals in some of the gels prompted us to optimize the transimination protocol. First, we treated CA‐II labeled with **L4R1** with varying amounts of reporter group. Approximately two to three equivalents of **FITC am‐zide** proved to be optimal when performing the exchange at pH 8.2 (Figure 3 A). At this concentration, the fluorescent signal was still acceptable for the target protein, while the background signal was minimal. Next, we investigated how the pH affected the transimination reaction. Lowering the pH of the samples during the exchange step increased, as expected,^[14]^ the efficiency (Figure 3 B). Compared to exchange at pH 8.2, the signal intensity for samples treated at pH 5–6 with **FITC am‐zide** was increased ≈1.5 times. Finally, we assessed the effect of the reaction time on transimination at pH 5.3. Exchange for 15 minutes resulted in a fluorescent signal that is only 1.15 fold less intense than that obtained after two hours exchange (Figure 3 C). Moreover, extending the incubation to overnight had a marginal effect on the signal intensity. Similar trends for the number of equivalents, pH and time‐dependency were observed for BirA (Figure S2). The rapid exchange is a significant improvement with respect to transimination of non‐iminoboronate linkers, which required twenty‐four hours.^[6]^

**Figure 3:**
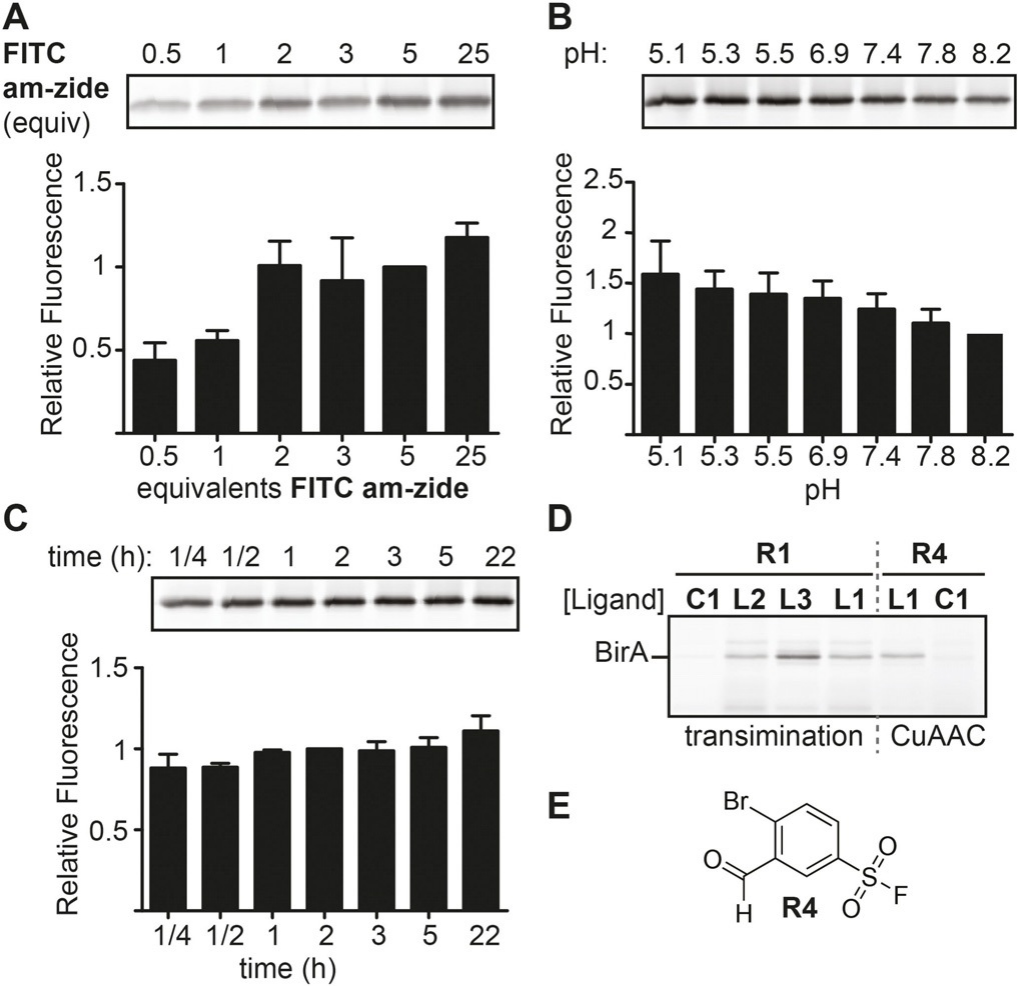
Optimization of the transimination conditions using labeled CA‐II. **A–C**) The effect of the amount of **FITC am‐zide** (**A**), the pH (**B**) and the exchange time (**C**) on the intensity of the fluorescent signal in the transimination reaction. Relative fluorescent intensities were determined by normalizing the signal to 5 equivalents **FITC am‐zide** (**A**), to pH 8.2 (**B**), and to 2 hours (**C**). All experiments were performed in quadruplicate. **D**) Comparison of read‐out by CuAAC with the stable acyl hydrazone of **R4** and transimination of probes made from **R1. E**. Structure of **R4**. General labeling conditions: 5 μm CA‐II or 2.5 mg mL^−1^ BirA‐overexpressing *E. coli* lysate; incubation with 20 μm probe for 2 h.

A head‐to‐head comparison of transimination and CuAAC visualization on BirA demonstrated that the signal was similar for both visualization methods (Figure 3 D). Importantly, the transimination method identified **L3R1** as an efficient probe for BirA, something which could not have been achieved using CuAAC. Similar results were obtained for Strp, albeit that these results are less straightforward to interpret due to the monomeric and tetrameric species present on the gel (Figure S3).

Next, we investigated the effect of the iminoboronate chemistry on the other steps of the protocol. Binding of the probe to glycans during the labeling step, which could be induced by the boronic acid moiety in the probe,[^12^, ^19^] proved to be negligible, since an excess of sucrose neither affected labeling of the target nor of the glycosylated control proteins avidin and OVA (Figure S4). The small amounts of off‐target labeling, which are observed in all experiments, could be suppressed partly by increasing the ligand‐to‐**R1** ratio (Figure S5). This observation suggests that hydrolysis of the iminoboronate linkage may liberate a small amount of **R1**, which reacts in a ligand‐independent fashion.

The iminoboronate chemistry proved to be beneficial for probe formation. Comparison of the labeling intensity of **L4R1** formed overnight with that of **L4R1** prepared by mixing **L4** and **R1** prior to the experiment revealed that the probes were formed within 15 minutes (Figure S6). Excitingly, the iminoboronate chemistry allows the formation of probes in the presence of proteins. Already 30 minutes after adding ligand **L4** and reactive group **R1** to a protein mixture, we observed fluorescent labeling of CA‐II, albeit not as high as the signal for the preformed **L4R1** probe (Figure S7). Importantly, the differences in signal intensities of the POI disappeared when we increased the labeling time to 5 h. Only a minimal increase in labeling by the non‐targeted **C1R1** is observed when the probe is formed in the labeling mixture. Experiments with **L3** and **R1** and BirA in lysate revealed that the probes can even be formed in the context of a lysate, although in this case the labeling was not as efficient as with the preformed probe.

To demonstrate the suitability of the iminoboronate chemistry in probe development, we expanded the panel of ligands with sulfonamides **L4–L9** and the panel of reactive groups with epoxide **R2** and arylazide photocrosslinker **R3** (Figure 4). The iminoboronate chemistry allowed straightforward screening of different combinations of the reactive groups and ligands (Figure 4 B, S8–S10) and it could be used to assess the influence of the linker length on labeling efficiency (Figure S11).

**Figure 4:**
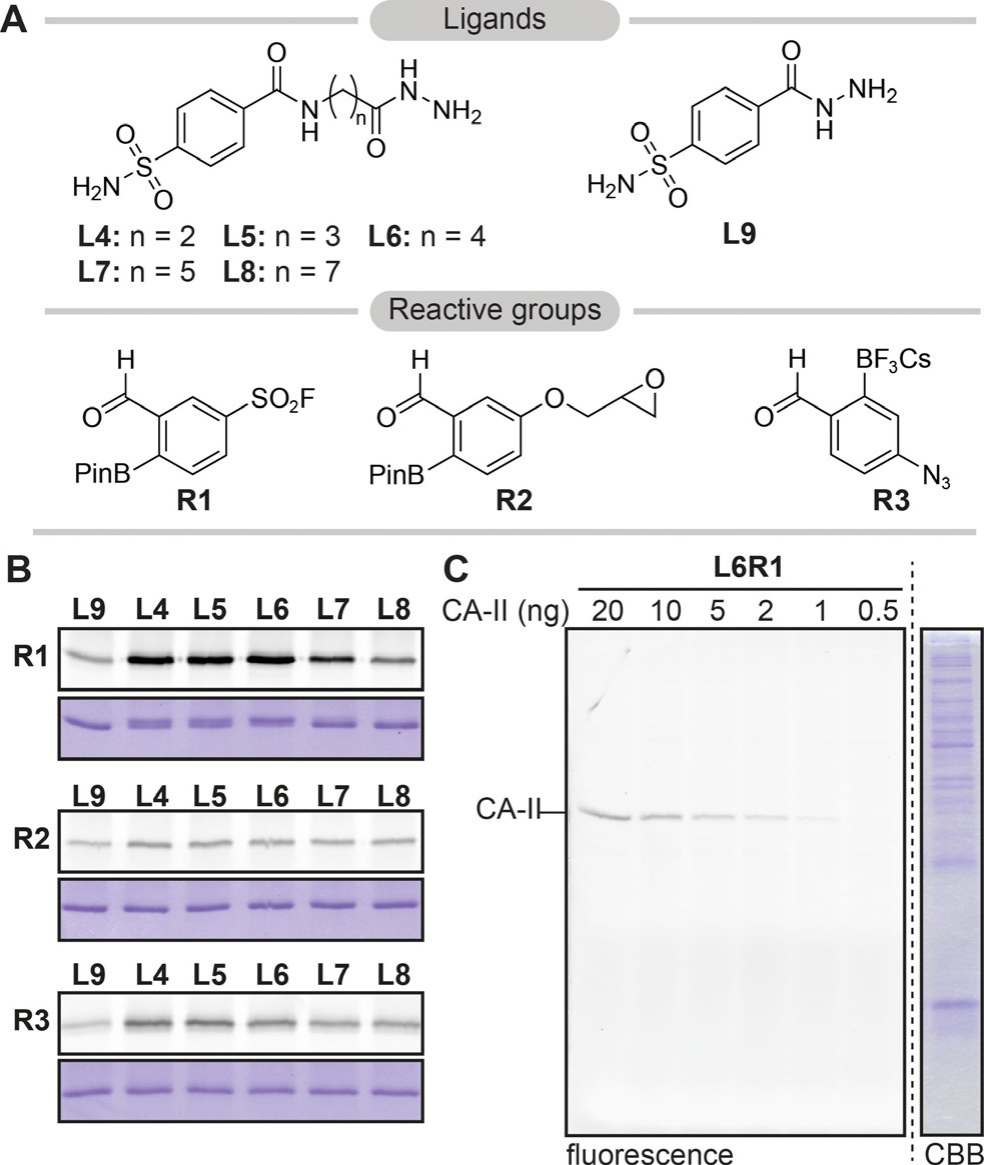
**A**) Structures of sulfonamide ligands **L4–L9**, reactive groups **R2** and **R3**. Prior to probe formation, the trifluoroborate salt of **R3** is converted into the boronic acid by incubation in aqueous solution. **B**) Labeling of CA‐II (5 μm) with the new probes (20 μm). **R3** is activated by irradiation at 312 nm for 15 minutes. **C**) The indicated amount of CA‐II was spiked in *E. coli* lysate (2 mg mL^−1^) and labeled with **L6R1** (100 nm) in order to determine the sensitivity of the iminoboronate probe.

The labeling properties of several of the new probe leads were studied in greater detail (Figure S12–S14). Time‐dependency studies revealed that maximal labeling was reached within two to four hours for **R1** probes and after overnight incubation for **R2** probes. Furthermore, irradiation with 312 nm light for 1 minute was sufficient to label the target proteins with **R3**‐based photocrosslinker probes. For **L6R1**, the most promising CA‐II probe, we also assessed the selectivity and the sensitivity. We spiked CA‐II in *E. coli* lysate and subjected this to labeling with **L6R1** (Figure 4 C). Some background labeling was observed when we used 20 μm of **L6R1** (Figure S15). However, lowering the probe concentration to 100 nm allowed us to detect as little as 1–2 ng CA‐II in 10 μg lysate, while we did not observe any off‐target labeling in the lysate.

Finally, we investigated if the iminoboronate probes could be applied on cells using BioY^[20]^ as a model protein. Our group previously employed a His‐tagged version of this biotin transporter and the mutants BioY‐N79K and BioY‐R93K, which carry an additional lysine near the binding site, to study on‐cell labeling of targeted diazotransfer reagents.^[21]^ To perform the on‐cell labeling studies, we first had to identify an iminoboronate probe for BioY. Therefore, we prepared a library of probes based on ligands **L1–L3** and reactive groups **R1‐R3**. To increase the chances further, we included two additional ligands (**L10** and **L11**, Figure 5 A). We screened the library on membrane fractions of *Lactococcus lactis* cells that either overexpressed BioY‐*wt* or the mutants BioY‐N79K and BioY‐R93K. As a control, we took the membrane fraction of non‐induced BioY‐*wt* cells. For several of the probes, a fluorescent signal at ≈20 kDa, which corresponds with the molecular weight of BioY, was observed. This signal was absent in the non‐induced samples and was less pronounced for the non‐targeted probes (Figure S16). Incubation of BioY‐R93K lysate with the probe **L10R1** resulted in the most pronounced labeling. Labeling of this protein by **L10R1** was blocked in the presence of an excess of biotin (Figure 5 B), confirming that labeling is ligand dependent and that BioY‐R93K is being labeled. We therefore used **L10R1** for the labeling of BioY on *L. lactis* cells. For the cell labeling, we added **L10R1** to a suspension of either *L. lactis* overexpressing BioY‐R93K or, as a control, non‐induced BioY‐*wt* in HEPES (pH 8.0) and left it to react for two hours. We removed the excess of probe by washing the cells with buffer prior to on‐cell transimination with **FITC am‐zide** at pH 5.3. To analyze the labeled proteins, we lysed the cells, isolated the membrane fraction, and subjected it to SDS‐PAGE. Analysis of the in‐gel fluorescence revealed that BioY‐R93K had been successfully labeled with **L10R1**, while limited or no labeling of BioY was observed for the control **C1R1** and for the cells where expression had not been induced (Figure 5 C). We also observed a fluorescent band at high molecular weight in both samples, indicating off‐target labeling. Promisingly, the labeling of BioY‐R93K indicates that chemical probes containing an iminoboronate linker can be used in cell labeling studies.

**Figure 5:**
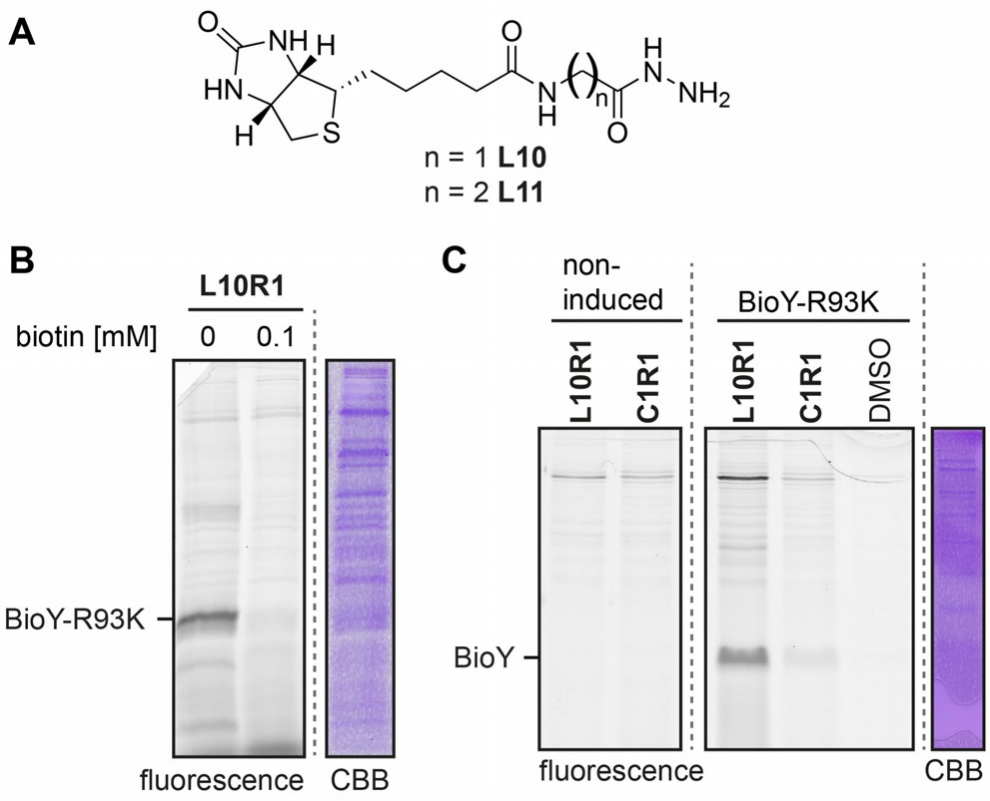
**A**) Biotin‐ligands **L10** and **L11. B**) Labeling of membrane extracts (2 mg mL^−1^) overexpressing BioY‐R93K with **L10R1** in the presence and absence of biotin. Incubation with 20 μm probe for 2 h; Read‐out: **FITC am‐zide. C**) In‐gel fluorescence of *L. lactis* cells that were not induced or overexpressing mutant BioY‐R93K. Cells were labelled for 2 h with the indicated probe and the ligand was subsequently exchanged on the cell surface by **FITC am‐zide**.

In conclusion, we have shown that α‐nucleophile ligands can be coupled successfully to 2‐FPBA reactive groups and the resulting iminoboronate probes can be used without further purification to label proteins in complex mixtures. Transimination with an α‐amino hydrazide reporter group enables reliable read‐out of the labeled proteins. Due to the fast reaction kinetics and the bio‐orthogonality of iminoboronate formation, the probes can even be prepared in the context of a protein mixture. Due to the modular nature of the iminoboronate linker, this methodology should be widely applicable with any ligand that can be converted into an α‐nucleophile. Therefore, we believe that using iminoboronate probes can greatly expedite the screening of reactive groups towards the identification of new chemical probes.

## Conflict of interest

The authors declare no conflict of interest.

## Supporting information

As a service to our authors and readers, this journal provides supporting information supplied by the authors. Such materials are peer reviewed and may be re‐organized for online delivery, but are not copy‐edited or typeset. Technical support issues arising from supporting information (other than missing files) should be addressed to the authors.

Supplementary
