# Supplementary material for: Iminoboronates as Dual‐Purpose Linkers in Chemical Probe Development
Source: Chemistry. 2021 Jan 14;27(10):3292–6. doi: 10.1002/chem.202005115 (PMC7898632; doi:10.1002/chem.202005115)
Supplement: Supplementary file 1 — Supplementary [file CHEM-27-3292-s001.pdf]

Chemistry–A European Journal

Supporting Information

# Iminoboronates as Dual-Purpose Linkers in Chemical Probe Development

Antonie J. van der Zouwen,[a] Aike Jeucken,[b] Roy Steneker,[a] Katharina F. Hohmann,[a] Jonas Lohse,[a] Dirk J. Slotboom,[b] and Martin D. Witte*[a]

| | | *Page nr* |
|---|---|---|
| Table S1 | Structures of the iminoboronate probes | 2 |
| Figure S1 | Full gels belonging to Figure 2 in the main paper | 7 |
| Figure S2 | Optimization of the transimination with **FITC am-zide** | 8 |
| Figure S3 | Comparison of labeling efficiency between transimination or CuAAC | 12 |
| Figure S4 | Assessment of potential glycan binding | 13 |
| Figure S5 | Different ligand versus reactive group ratios | 14 |
| Figure S6 | Time required to form iminoboronate probes | 15 |
| Figure S7 | Labeling efficiency of iminoboronate probes formed in the presence of protein | 16 |
| Figure S8-S10 | Screening of ligands and reactive groups against streptavidin, avidin and BirA | 17 |
| Figure S11 | Screening the effect of the linker length on the labeling efficiency | 20 |
| Figure S12-S14 | Optimization of labeling with reactive groups **R1-R3** | 21 |
| Figure S15 | Spiking experiments with CA-II | 24 |
| Figure S16 | Screening of ligands and reactive groups against BioY in cell lysate | 27 |
| Figure S17 | Competition experiment on labeling of BioYR93K | 31 |
| Figure S18 | Cell labeling of *Lactococcus lactis* BioYR93K | 32 |
| Biochemical procedures | | 34 |
| Synthetic procedures | | 40 |
| References | | 51 |
| Spectra | | 52 |

**Table S1** Structures of probes used in this study

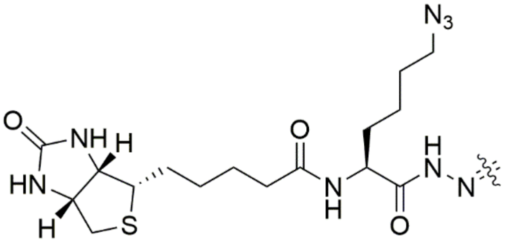

**L1**

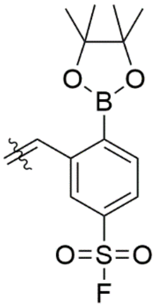

**L1R1**

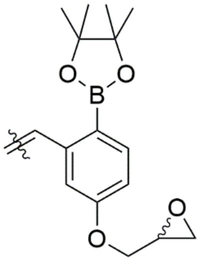

**L1R2**

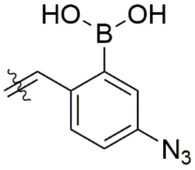

**L1R3**

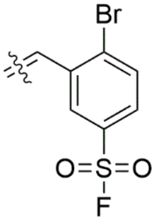

**L1R4**

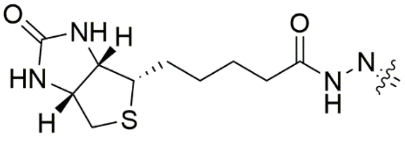

**L2**

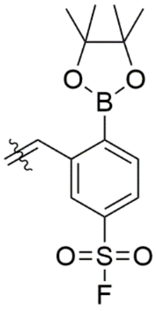

**L2R1**

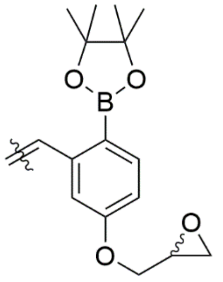

**L2R2**

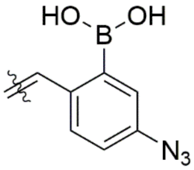

**L2R3**

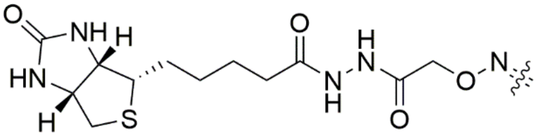

**L3**

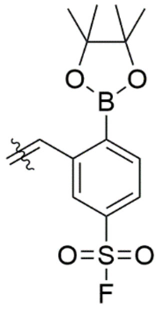

**L3R1**

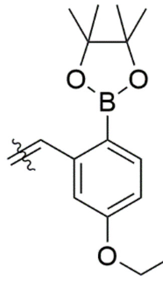

**L3R2**

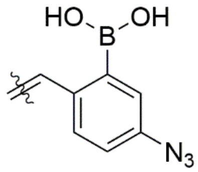

**L3R3**

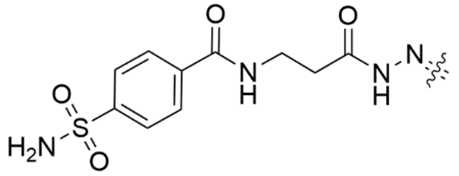

**L4**

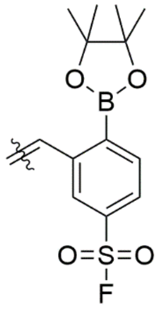

**L4R1**

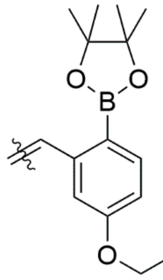

**L4R2**

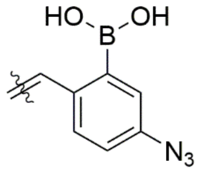

**L4R3**

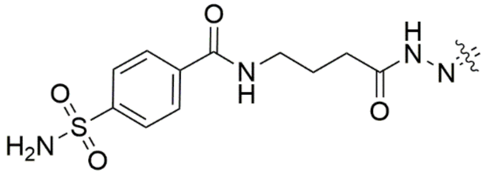

**L5**

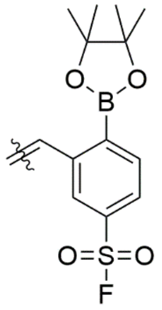

**L5R1**

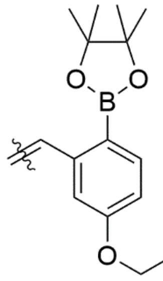

**L5R2**

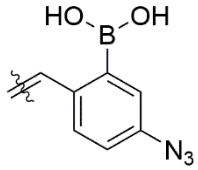

**L5R3**

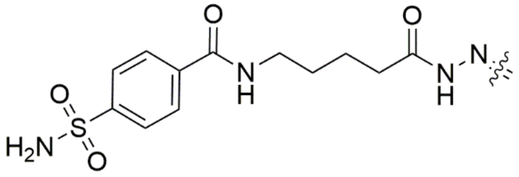

**L6**

<table>
<tr><td>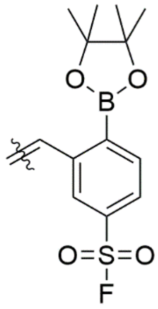<br>**L6R1**</td><td>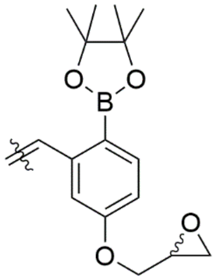<br>**L6R2**</td><td>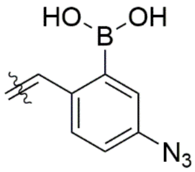<br>**L6R3**</td></tr>
<tr><td colspan="3">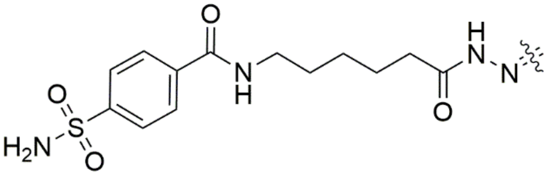<br>**L7**</td></tr>
<tr><td>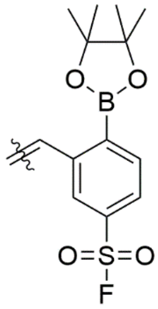<br>**L7R1**</td><td>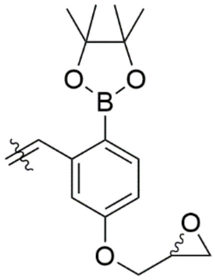<br>**L7R2**</td><td>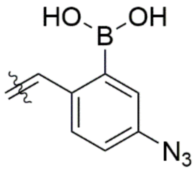<br>**L7R3**</td></tr>
<tr><td colspan="3">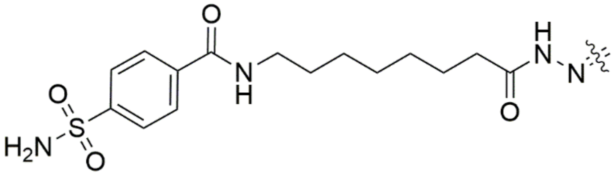<br>**L8**</td></tr>
<tr><td>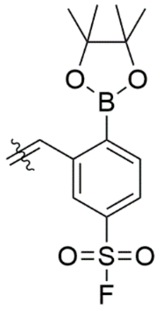<br>**L8R1**</td><td>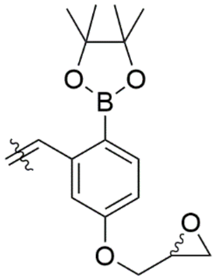<br>**L8R2**</td><td>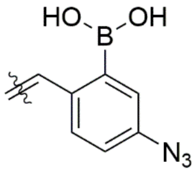<br>**L8R3**</td></tr>
<tr><td colspan="3">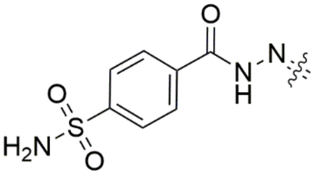<br>**L9**</td></tr>
</table>

| | | |
|---|---|---|
| 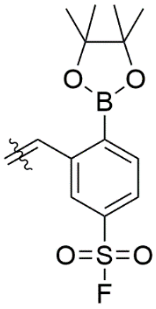<br>**L9R1** | 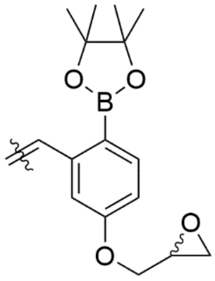<br>**L9R2** | 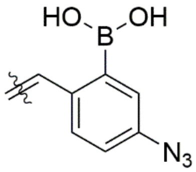<br>**L9R3** |
| 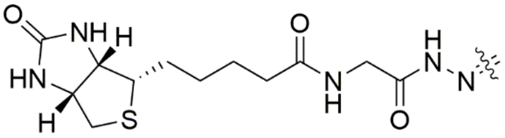<br>**L10** | | |
| 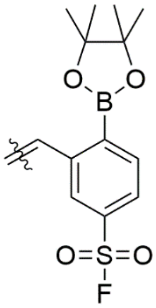<br>**L10R1** | 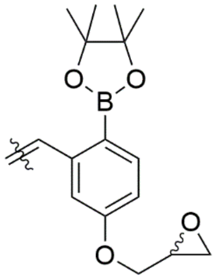<br>**L10R2** | 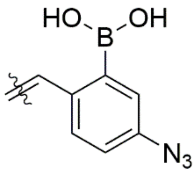<br>**L10R3** |
| 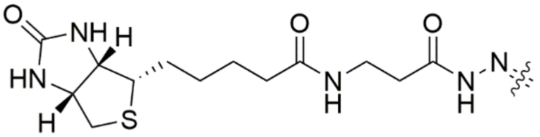<br>**L11** | | |
| 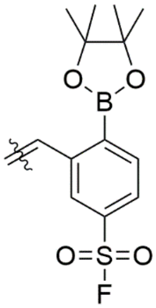<br>**L11R1** | 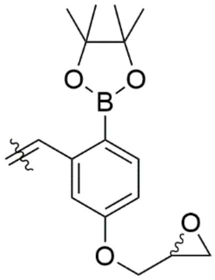<br>**L11R2** | 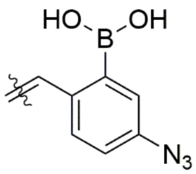<br>**L11R3** |
| 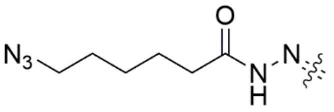<br>**C1** | | |

<table>
<tr><td>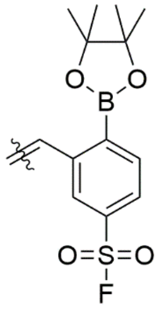<br>**C1R1**</td><td>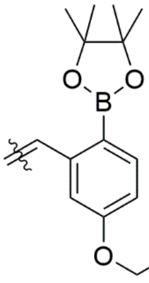<br>**C1R2**</td><td>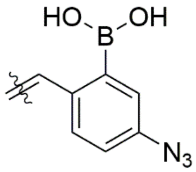<br>**C1R3**</td><td>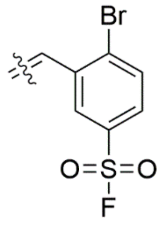<br>**C1R4**</td></tr>
<tr><td colspan="4">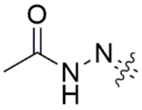<br>**C2**</td></tr>
<tr><td>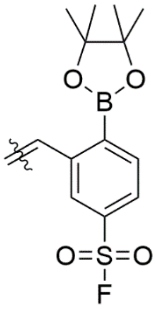<br>**C2R1**</td><td>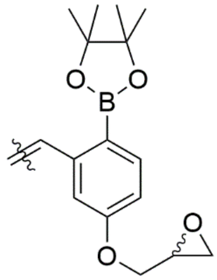<br>**C2R2**</td><td colspan="2">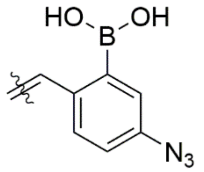<br>**C2R3**</td></tr>
</table>

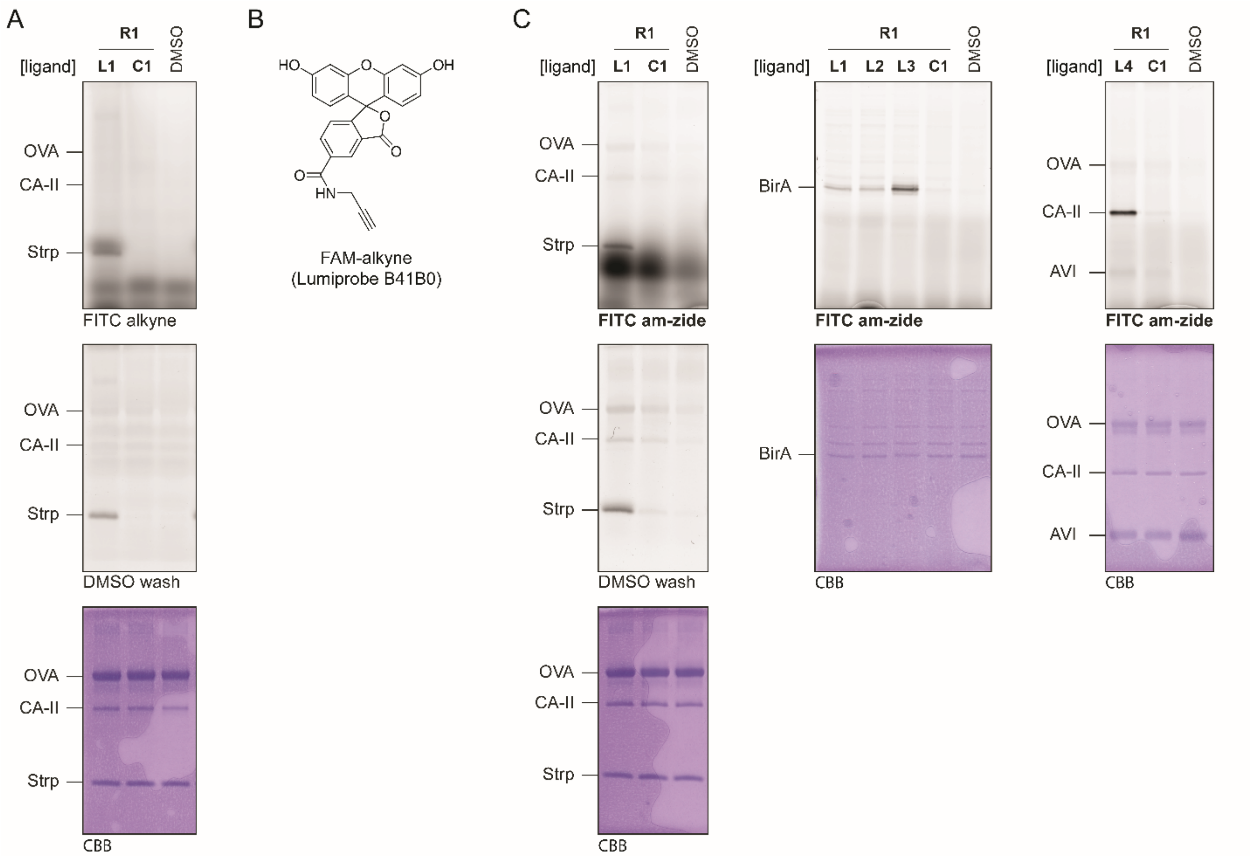


**Figure S1**. Full gels belonging to Figure 2 in the main paper. **A.** Labeling of Strp with probe **L1R1** and control **C1R1**; read-out with FAM-alkyne. **B.** Structure of FAM-alkyne (Lumiprobe B41B0). **C.** Labeling of Strp with probe **L1R1**, labeling of BirA with **L1R1**, **L2R1** and **L3R1**, labeling of CA-II with **L4R1**; read-out with **FITC am-zide**. Leftover **FITC am-zide** or an adduct thereof sometimes hampered analysis of the labeling reactions, but washing the gels in DMSO overnight mitigated this.

Conditions: 5 µM CA-II, 25 µM OVA, 25 µM AVI in 50 mM HEPES pH 8.2 or 2.5 mg/mL *E. coli* lysate overexpressing BirA in 50 mM HEPES pH 8.2; labeling with 20 µM probe for 2 hours; CuAAC with 19 equivalents FAM-alkyne, 96 equivalents $CuSO_4$ and 96 equivalents THPTA for 2 hours; transimination with 5 equivalents **FITC am-zide** for 2 hours.

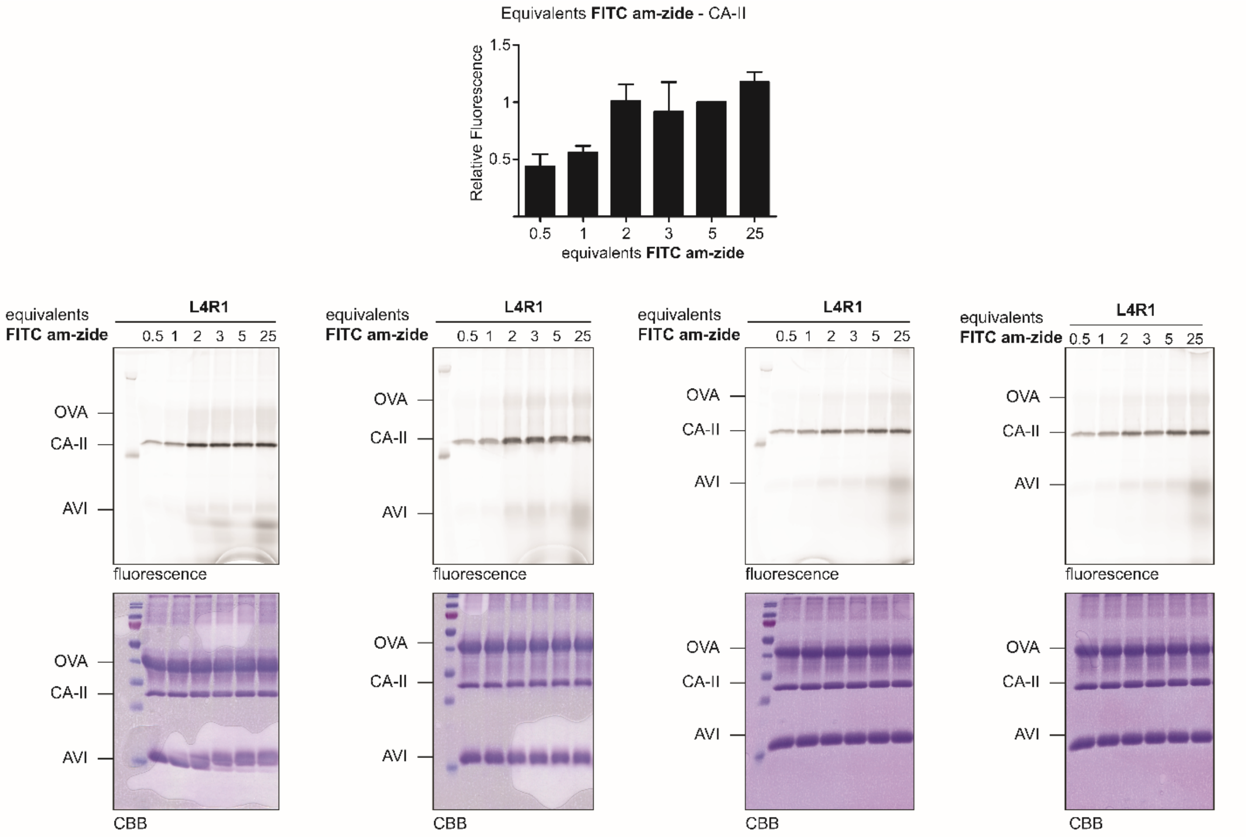


**Figure S2A**. Determination of the influence of the equivalents of **FITC am-zide** on the transimination efficiency using CA-II labeled with **L4R1** as a model substrate. The fluorescent signals in all gels were quantified using ImageJ and normalized to the fluorescence obtained with 5 equivalents **FITC am-zide**. All experiments were carried out in quadruplicate. General conditions: 5 μM CA-II, 25 μM OVA, 25 μM AVI in 50 mM HEPES pH 8.2; labeling with 20 μM **L4R1** for 2 hours; transimination at pH 8.2 with the indicated amount of **FITC am-zide** for 2 hours.

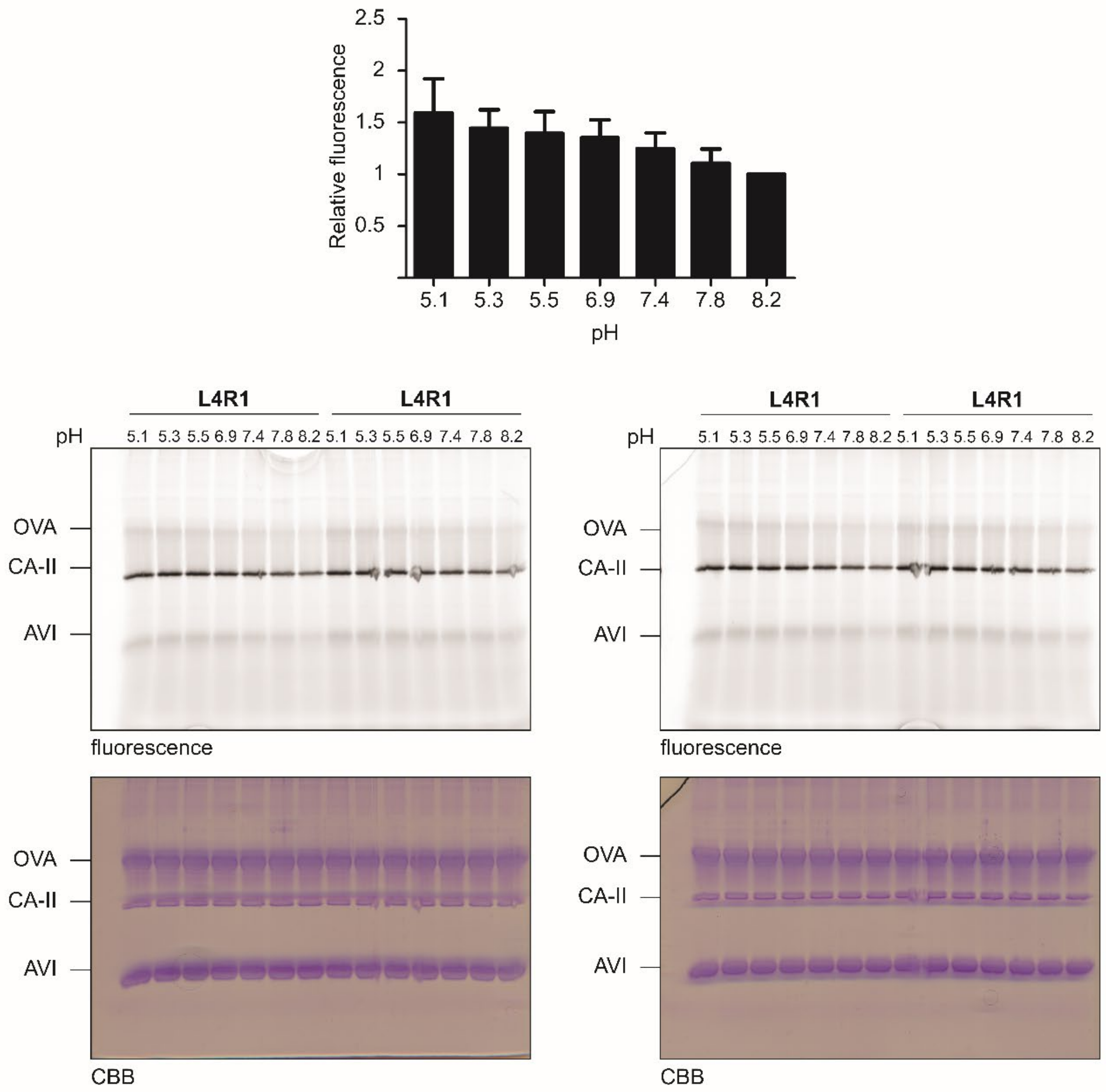


**Figure S2B**. Determination of the influence of pH on the transimination efficiency using CA-II labeled with **L4R1** as a model substrate. The fluorescent signals in all gels were quantified using ImageJ and normalized to the fluorescence obtained at pH 8.2 with 3 equivalents **FITC am-zide**. General conditions: 5 μM CA-II, 25 μM OVA, 25 μM AVI in 50 mM HEPES pH 8.2; labeling with 20 μM **L4R1** for 2 hours; transimination with 3 equivalents **FITC am-zide** at the indicated pH for 2 hours.

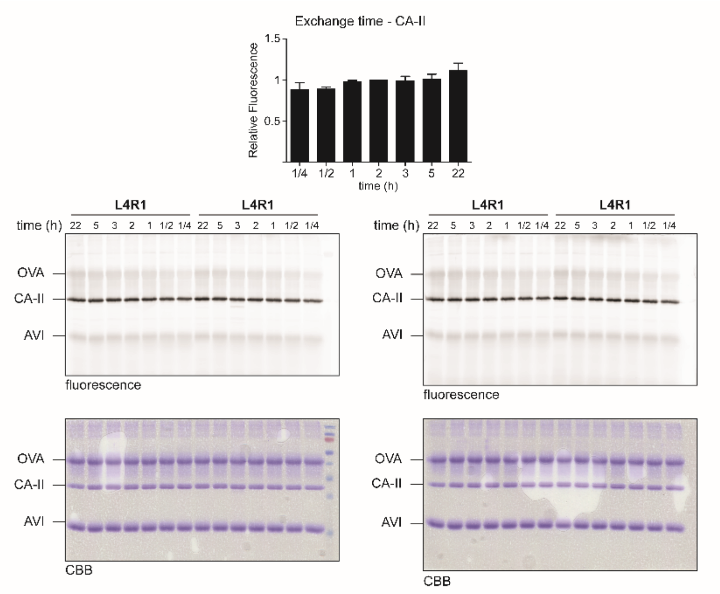

**Figure S2C**. Determination of the influence of the exchange time on the transimination efficiency using CA-II labeled with **L4R1** as a model substrate. The fluorescent signals in all gels were quantified using ImageJ and normalized to the fluorescence obtained at 2 hours exchange time. All experiments were carried out in quadruplicate. General conditions: 5 μM CA-II, 25 μM OVA, 25 μM AVI in 50 mM HEPES pH 8.2; labeling with 20 μM **L4R1** for 2 hours; transimination with 3 equivalents **FITC am-zide** at pH 5.3 for the indicated time.

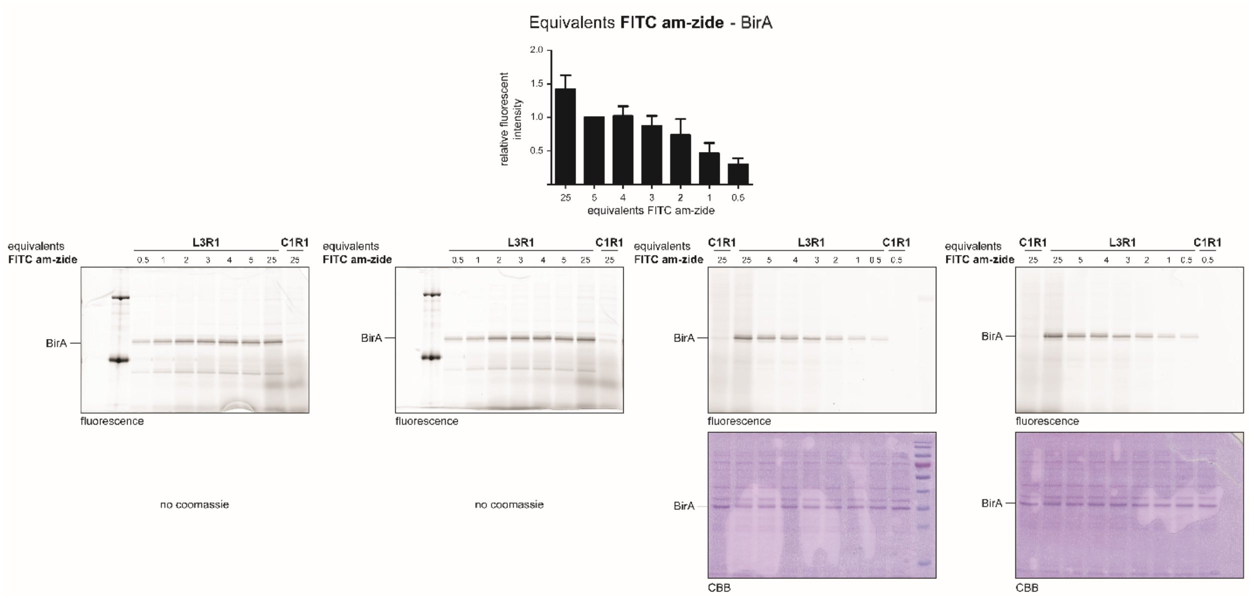

**Figure S2D**. Determination of the influence of the equivalents of **FITC am-zide** on the transimination efficiency using BirA labeled with **L3R1** as a model substrate. The fluorescent signals in all gels were quantified using ImageJ and normalized to the fluorescence obtained with 5 equivalents **FITC am-zide**. All experiments were carried out in quadruplicate. General conditions: 2.5 mg/mL *E. coli* lysate overexpressing BirA in 50 mM HEPES pH 8.2; labeling with 20 μM **L3R1** for 2 hours; transamination at pH8.2 with the indicated amount of **FITC am-zide** for 2 hours.

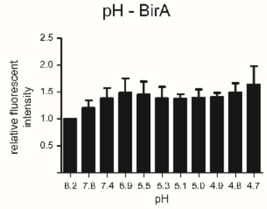


| 1 μL of [M] acetic acid | - | 0.1 | 0.2 | 0.3 | 0.4 | 0.5 | 0.6 | 0.7 | 0.8 | 0.9 | 1.0 |
|---|---|---|---|---|---|---|---|---|---|---|---|
| Final pH of the exchange solution | 8.2 | 7.8 | 7.4 | 6.9 | 5.5 | 5.3 | 5.1 | 5.0 | 4.9 | 4.8 | 4.7 |

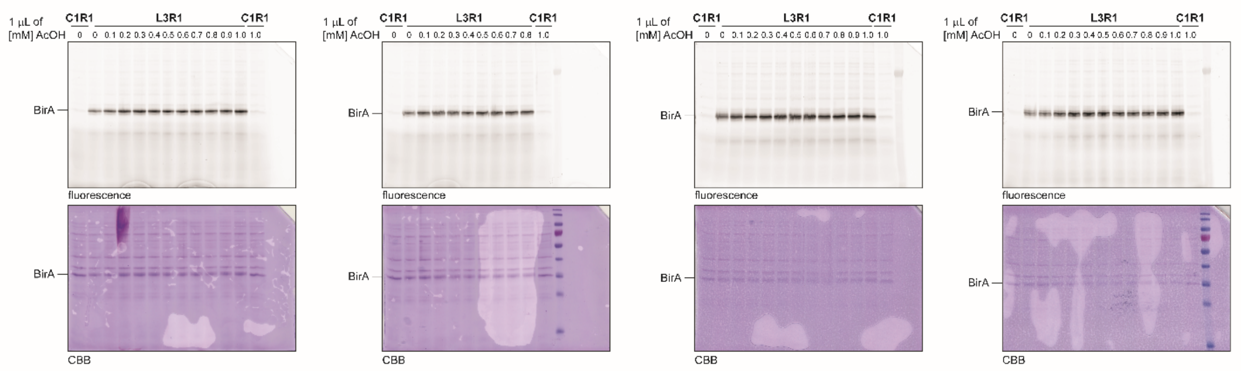


**Figure S2E**. Determination of the influence of pH on the transimination efficiency using BirA labeled with **L3R1** as a model substrate. The samples were boiled after addition of Laemmli sample buffer. The fluorescent signals in all gels were quantified using ImageJ and normalized to the fluorescence obtained at 8.2. All experiments were carried out in quadruplicate, except for the experiments for pH 4.8 and pH 4.7, which were carried out in triplicate. General conditions: 2.5 mg/mL *E. coli* lysate overexpressing BirA in 50 mM HEPES pH 8.2; labeling with 20 μM **L3R1** for 2 hours; transimination with 3 equivalents **FITC am-zide** at the indicated pH for 2 hours.

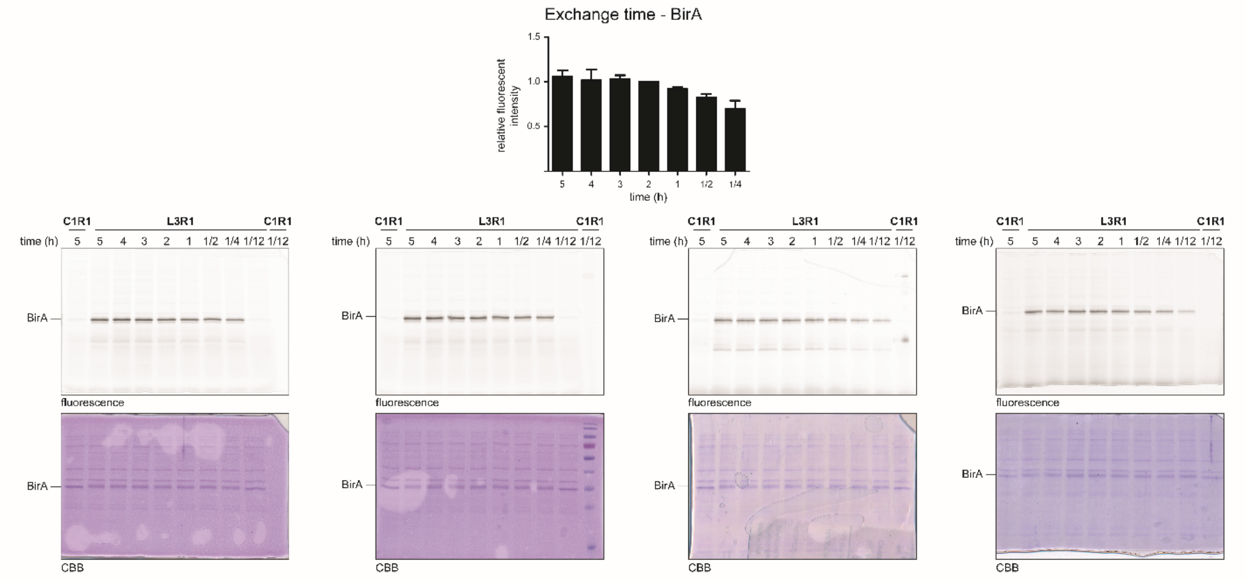


**Figure S2F**. Determination of the influence of the exchange time on the transimination efficiency using BirA labeled with **L3R1** as a model substrate. The fluorescent signals in all gels were quantified using ImageJ and normalized to the fluorescence obtained at 2 hours exchange time. All experiments were carried out in quadruplicate. General conditions: 2.5 mg/mL *E. coli* lysate overexpressing BirA in 50 mM HEPES pH 8.2; labeling with 20 μM **L3R1** for 2 hours; transimination with 3 equivalents **FITC am-zide** at pH 5.3 for the indicated time.

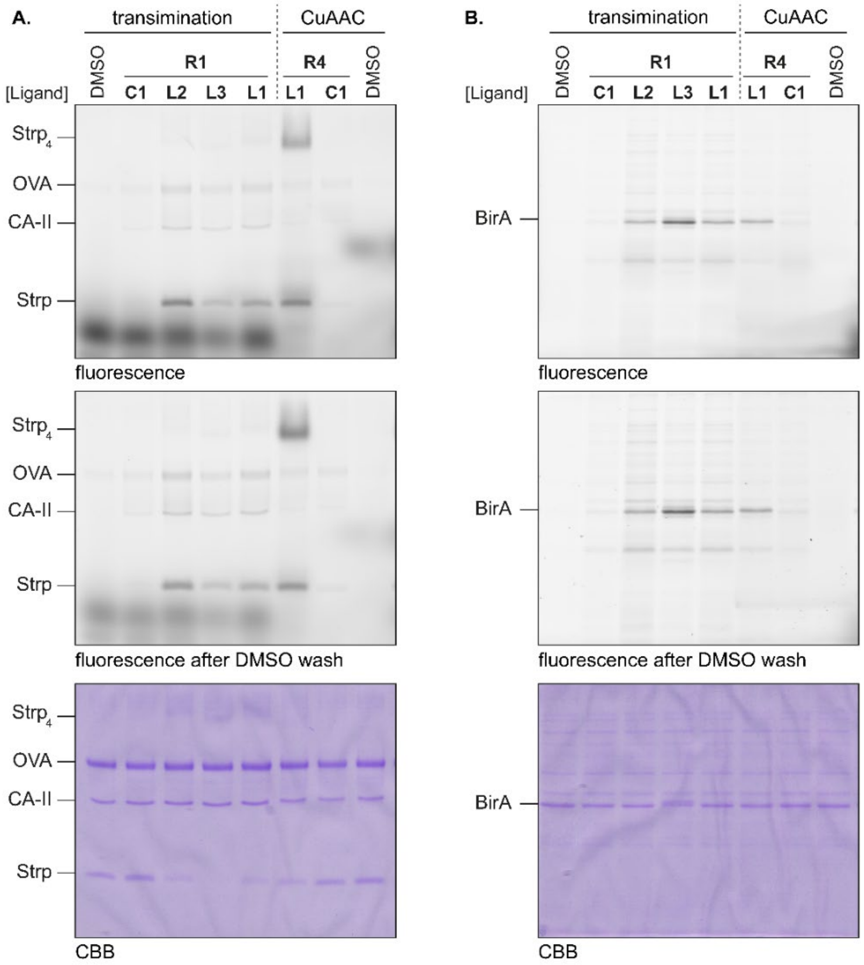


**Figure S3**. Comparison of the labeling efficiency of Strp (A.) and BirA (B.) when visualized with transimination (**FITC am-zide**) or CuAAC (FAM-alkyne). The indicated ligands were incubated with **R1** overnight to form the iminoboronate probes. The resulting probes (final concentration 20μM) were incubated in the presence of Strp (25 μM), CA-II (5 μM) and ovalbumin (25 μM) in HEPES (50 mM, pH 8.2) or in the presence of lysate of *E. coli* overexpressing BirA (2.5 mL/mL) for 2 hours, after which the proteins were denatured by the addition of 1% SDS and boiling. The labeled proteins were detected either by transamination with three equivalents of **FITC am-zide** at pH 5.3 for 2 hours or by CuAAC with FAM-alkyne (19 equivalents FAM-alkyne, 96 equivalents $CuSO_4$, 96 equivalents THPTA).

Potentially, partial hydrolysis of the iminoboronate linker in the CuAAC step and/or the subsequent SDS-PAGE might lead to loss of signal in the CuAAC samples. Therefore, for a more reliable comparison, we prepared probes with **R4**, a precursor of **R1** (see table S1 and Figure 3). As these form regular hydrazone probes, we reasoned they should be stable during the CuAAC conditions. The results indicate that for Strp, read-out with CuAAC may be more efficient (A). However, it is important to note that that the drawing conclusions from this experiment is hampered by the varying amounts of tetrameric and monomeric streptavidin (see CBB) and potential differences in transimination/CuAAC efficiencies between the monomeric and tetrameric forms. For BirA, however, the signal for transimination of **L1R1** is comparable to that of CuAAC of **L1-4** (B.). Furthermore, the signal for **L3R1**, that could only be visualized with transimination, is even more pronounced.

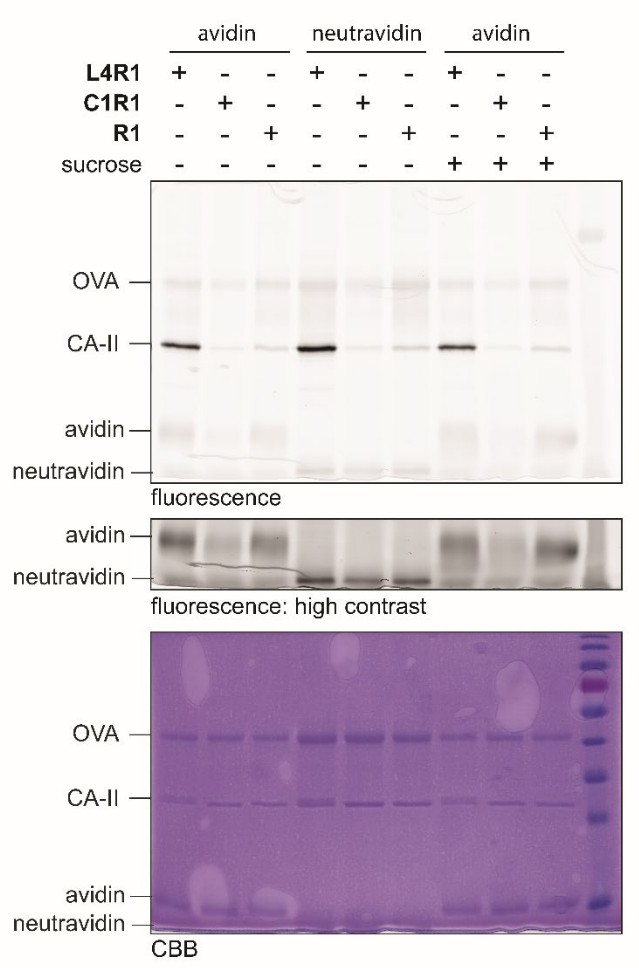


**Figure S4**. Effect of glycans on probe labeling. Potential glycan binding of the iminoboronate probes was assessed by incubating a mixture of ovalbumin (25 μM), CA-II (5 μM) and either avidin (25 μM) or neutravidin (25 μM) in HEPES (50 mM, pH 8.2) with **L4R1** (20 μM) in the presence or absence of sucrose (1 mM) for 2 hours. After this, the samples were subjected to transimination with three equivalents of **FITC am-zide** at pH5.3 for 2 hours, separated on an SDS-PAGE and the labeled proteins were visualized by in-gel fluorescence scanning.

As can be seen, addition of sucrose did not alter the labeling of CA-II by **L4R1**. The background is also similar to that of labeling without sucrose. Furthermore, replacing avidin for the deglycosylated neutravidin did also not make a difference for the labeling profile.

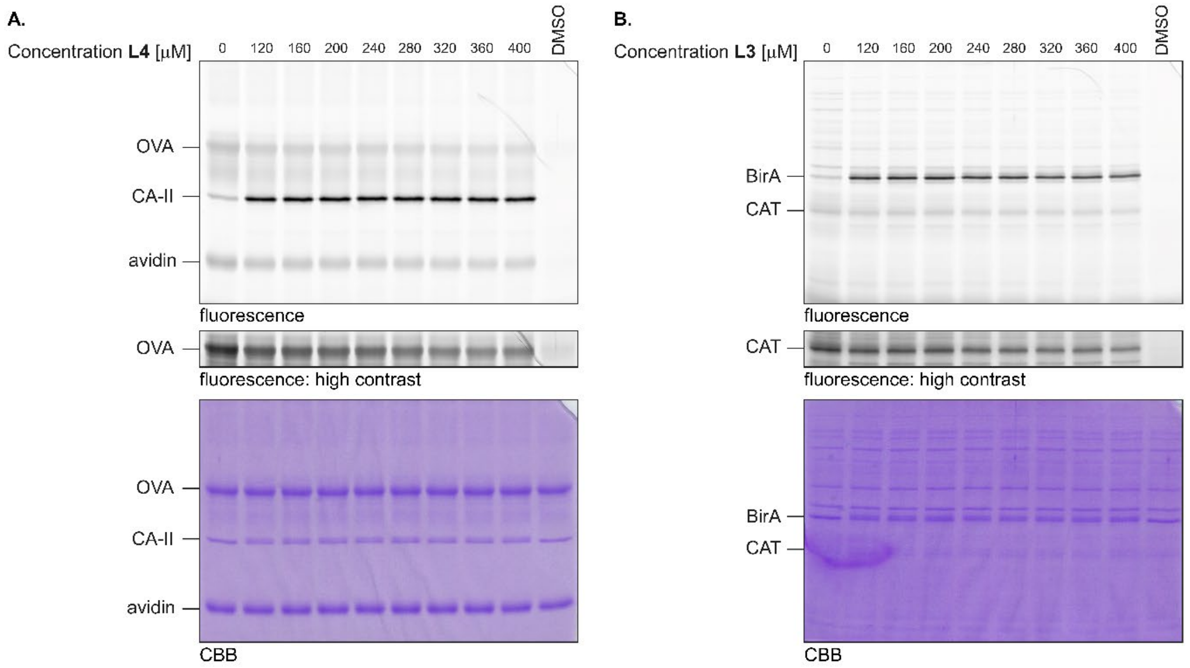


**Figure S5**. Effect of different ligand-reactive group ratios on labeling of the POI and off-targets. Varying concentrations of ligands **L3** and **L4** were incubated with a fixed concentration of **R1** (200 μM) to form the iminoboronate probes. The resulting probe mixtures were incubated either with a mixture of OVA (25 μM), CA-II (5 μM) and avidin (25 μM) in HEPES (50 mM, pH 8.2) (**A.**) or with cell lysate of *E. coli* cells overexpressing BirA (2.5 mg/mL) in HEPES (50 mM, pH 8.2) (B.) for 2 hours (final concentration of **R1** in the labeling reaction is 20 μM). The labeled proteins were subjected to transimination with three equivalents of **FITC am-zide** at pH5.3 for 2 hours, separated on an SDS-PAGE and visualized by in-gel fluorescence scanning.

The fluorescence scans (top gels) show minor labeling of CA-II or BirA when only **R1** is added. Upon addition of **L3** or **L4**, the fluorescent signals for BirA and CA-II, respectively, are increased significantly, while, the background signal goes down. At higher concentrations of ligand, the labeling signal starts to decrease, likely due to competition between ligand and iminoboronate probe. However, the background signal does decrease from the point that excess of ligand is present as well, albeit minimal (see high contrast fluorescence in the middle). This indicates that free **R1** to some extent may contribute to background labeling.

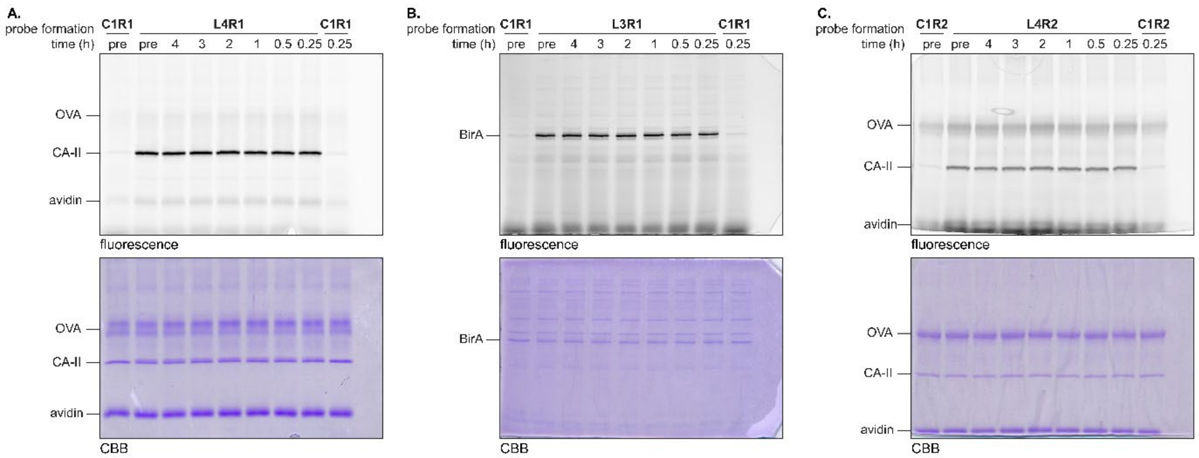


**Figure S6**. Time required to form iminoboronate probes. Ligands **C1**, **L3** and **L4** and reactive groups **R1** were added together in equimolar amounts and incubated for the indicated time, after which they were added to a mixture of CA-II (5 μM), avidin (25 μM) and ovalbumin (25 μM) in HEPES (50 mM, pH 8.2) (**A.**) or to lysate of *E. coli* cells overexpressing BirA (2.5 mg/mL) in HEPES (50 mM, pH 8.2) (**B.**) to give a final concentration of 20 μM probe. The labeled proteins were subjected to transimination with three equivalents of **FITC am-zide** at pH5.3 for 2 hours, separated on an SDS-PAGE and visualized by in-gel fluorescence scanning. Preformed probes were used as control and are indicated with “pre”. (**C.**) The same experiment as in panel A. was performed, but now using **R2** as reactive group rather than **R1**. As can be seen from the in-gel fluorescence, probes that were formed in 15 minutes resulted in the same intensity bands as the probes that were preformed overnight. This indicates that the iminoboronate probes rapidly form within 15 minutes.

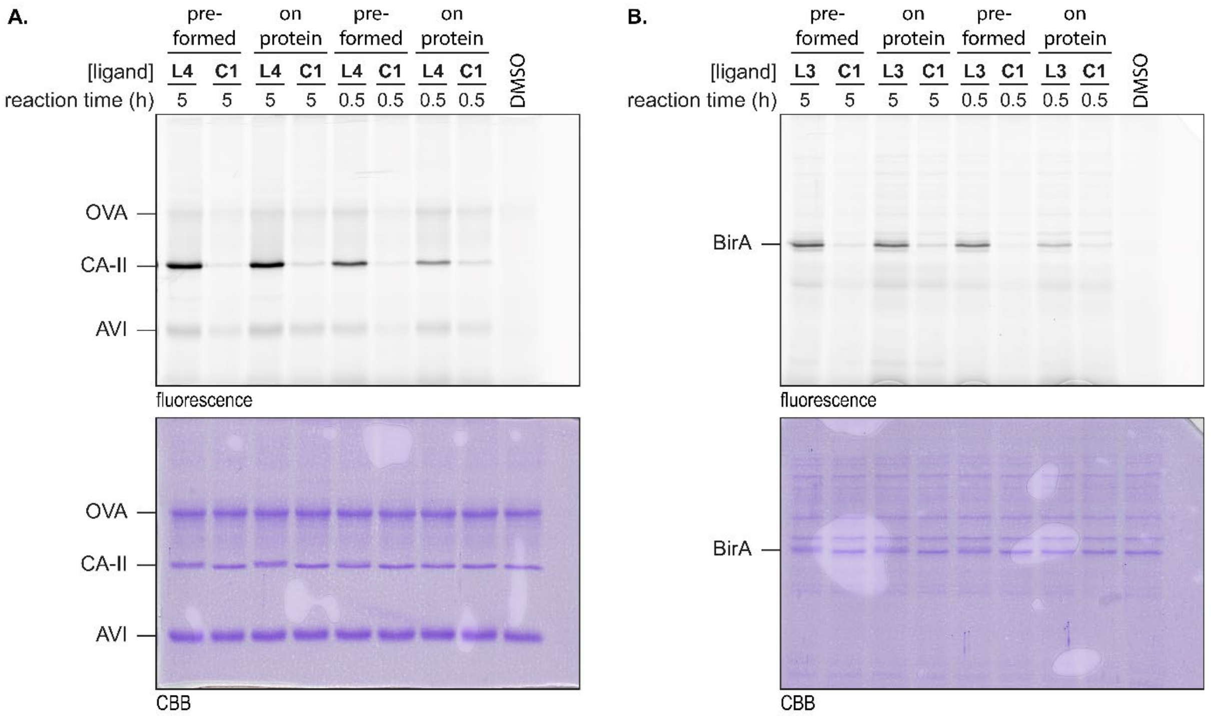


**Figure S7**. Probe formation in the presence of protein mixtures. Iminoboronate probes targeting CA-II (**A.**) and BirA (**B.**) were formed during the labeling reaction by adding an equimolar amount of the ligands **C1**, **L3** or **L4** (final concentration 20 µM) and the reactive group **R1** (final concentration 20 µM) to the protein mixture (5 µM CA-II with 25 µM avidin and 25 µM ovalbumin) or lysate of *E. coli* overexpressing BirA(2.5 mg/mL) in HEPES (50 mM, pH 8.2). The labeling reaction was either quenched after half an hour or after 5 hours by boiling in the presence of 1% SDS. The samples were subjected to transimination with three equivalents of **FITC am-zide** at pH 5.3 for 2 hours, separated on an SDS-PAGE and visualized by in-gel fluorescence scanning.

As a control, preformed probe was incubated for the same time with the protein mixtures and the fluorescent signals of the preformed probes were compared with those prepared in the protein mixture. Already 30 minutes after addition, fluorescent labeling can be observed, albeit not as high as the signal for probes preformed overnight in the absence of protein. Importantly, the difference between the signal intensities disappeared with increased labeling time, while labeling by non-targeted **C1R1** remained minimal. The background seems slightly higher for the probes that were formed in the presence of protein.

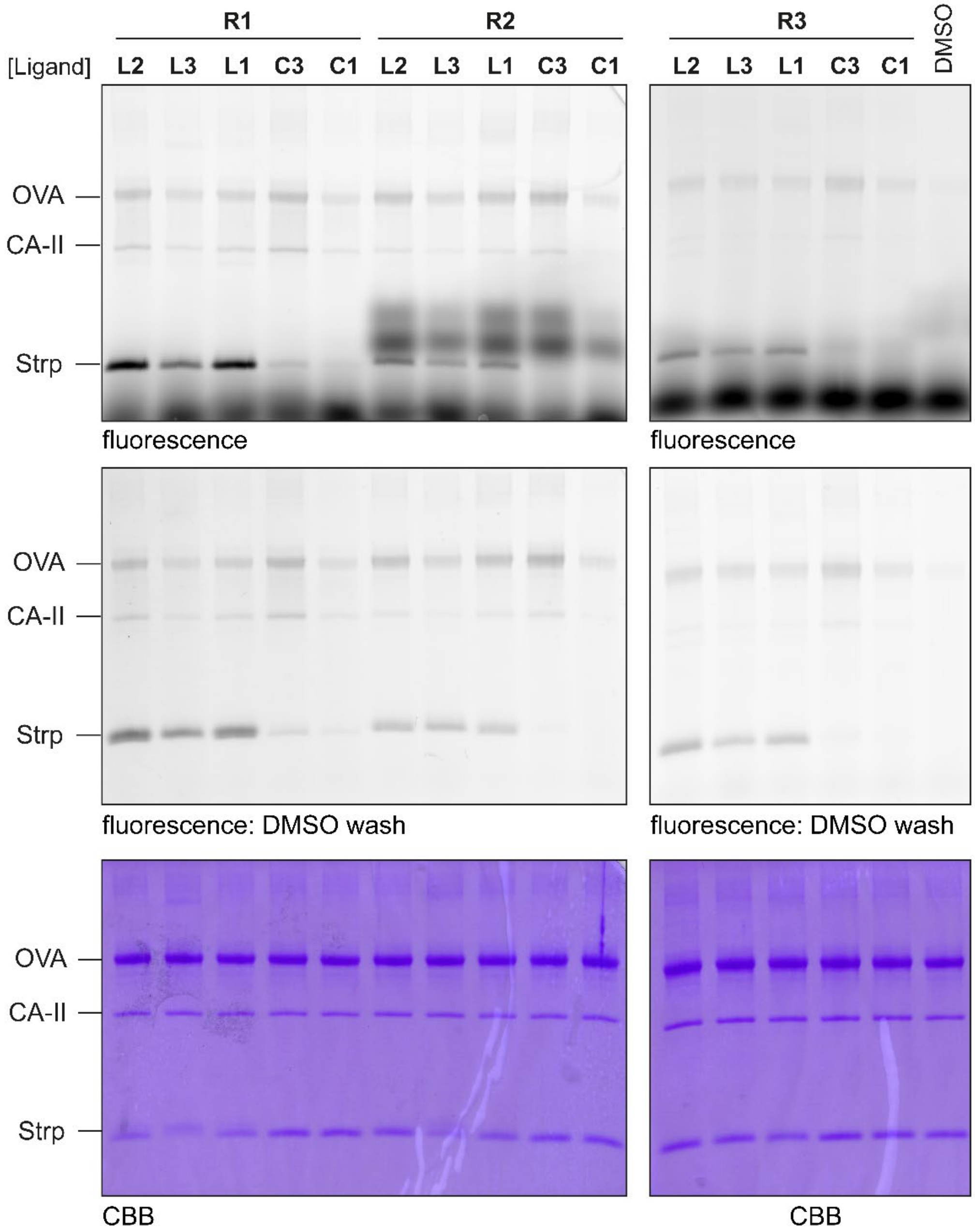


**Figure S8**. Screening of ligands and reactive groups against streptavidin. Iminoboronate probe (20 μM) was incubated for 2 hours in the presence of Strp (25 μM), CA-II (5 μM) and ovalbumin (25 μM). Samples containing **R3** were irradiation at 312 nm for 15 minutes. To the detect the labeled proteins, the ligand was exchanged by transimination with three equivalents of **FITC am-zide** at pH 5.3 for 2 hours. In-gel fluorescence showed that probes with **R1** labeled Strp most efficient, while the probes with **R2** and **R3** did label Strp but to a lesser extent. **L1R1** and **L2R1** were more efficient in labeling Strp than **L3R1**. Higher backgrounds were observed for control **C2** then control **C1**, which could be explained due to the smaller size of **C2**. This indicates that **C2** likely is not a good control ligand. Interestingly, the fluorescent signal for probe **L1R2** was much lower than expected from the CuAAC results, indicating less efficient transimination. This difference in reactivity might be caused by the electron-donating character of the

ether in **R2** versus the electron-withdrawing sulfonyl fluoride in **R1** and it suggest that the results have to be interpreted with care.

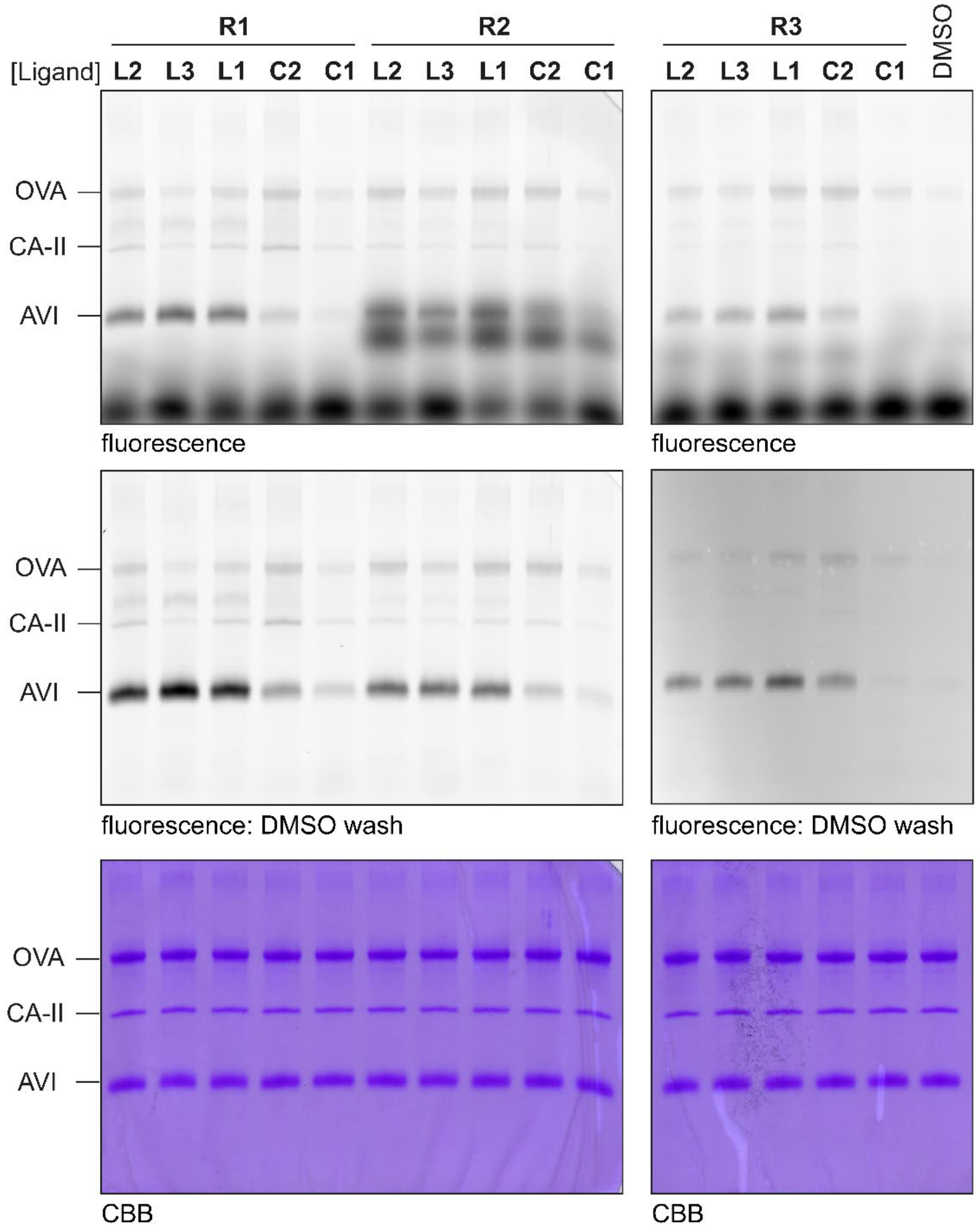


**Figure S9**. Screening of ligands and reactive groups against avidin. Iminoboronate probe (20 μM) was incubated for 2 hours in the presence of avidin (25 μM), CA-II (5 μM) and ovalbumin (25 μM). Samples containing **R3** were irradiation at 312 nm for 15 minutes. To the detect the labeled proteins, the ligand was exchanged by transimination with three equivalents of **FITC am-zide** at pH 5.3 for 2 hours. In-gel fluorescence showed that probes with **R1** labeled AVI most efficient, while the probes with **R2** and **R3** did label AVI but to a lesser extent. No real difference in labeling was observed between the different ligands. Higher backgrounds were observed for control **C2** then control **C1**, which could be explained due to the smaller size of **C2**. This indicates that **C2** likely is not a good control ligand.

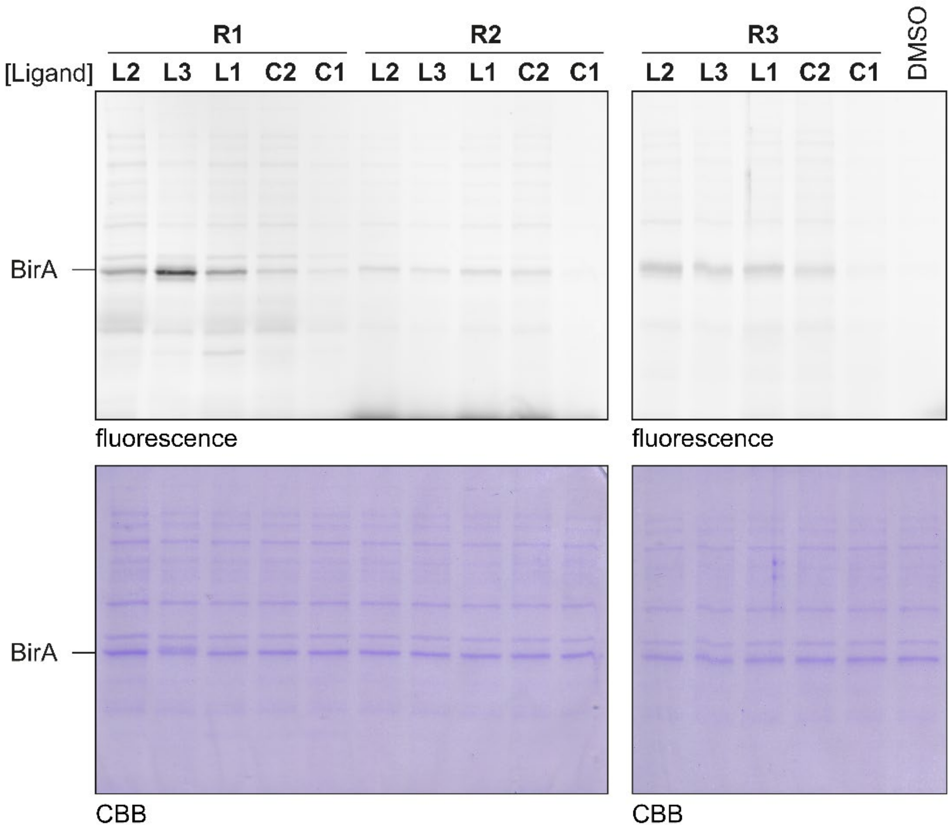


**Figure S10**. Screening of ligands and reactive groups against BirA. Iminoboronate probe (20 μM) was incubated for 2 hours in the presence of lysate of *E. coli* overexpressing BirA (2.5 mg/mL) Samples containing **R3** were irradiation at 312 nm for 15 minutes. To the detect the labeled proteins, the ligand was exchanged by transimination with three equivalents of **FITC am-zide** at pH 5.3 for 2 hours. In-gel fluorescence showed that probes with **R1** labeled BirA most efficient, while the probes with **R3** did label BirA but to a lesser extent and the probes with **R2** barely labeled BirA. Especially **L3R1** was efficient in labeling BirA. A similar ligand dependency was not observed for **R2** and **R3**.

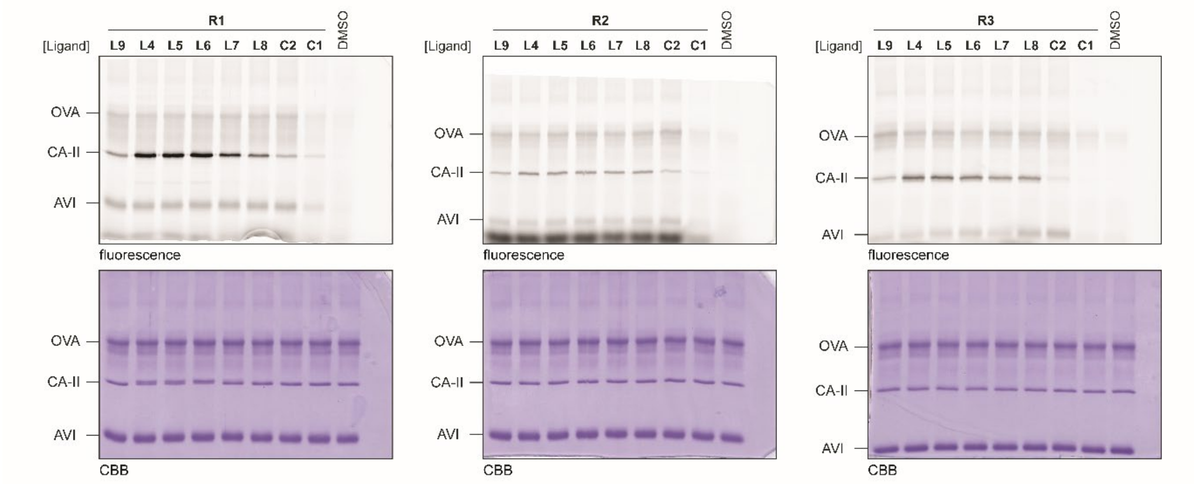


**Figure S11**. Screening the effect of the linker length on the labeling efficiency of CA-II. Ligands with different linker lengths **L4-L9** were reacted with **R1** (**A.**), **R2** (**B.**) or **R3** (**C.**). The resulting iminoboronate probes (20 μM) were incubated for 2 hours in the presence of CA-II (5 μM), avidin (25 μM) and ovalbumin (25 μM). Samples containing **R3** were irradiation at 312 nm for 15 minutes.

The labeled proteins were subjected to transamination with three equivalents of **FITC am-zide** at pH 5.3 for 2 hours and visualized after SDS-PAGE by in-gel fluorescence scanning.

The fluorescence scans showed that probes with **R1** labeled CA-II most efficient (A.), while the probes with **R2** (B.) and **R3** (C.) did label CA-II but to a lesser extent. Higher backgrounds were observed for control **C2** then control **C1**, which could be explained due to the smaller size of **C2**. This indicates that **C2** likely is not a good control ligand. Labeling was linker length-dependent for **R1** and **R3**, with **L6R1** and **L4R3** being most efficient, respectively, but less so for **R2**. Interestingly, upon Coomassie brilliant blue staining a second, slightly higher running band became visible for CA-II labeled with **R1**. Likely, this band represents **FITC am-zide**-modified CA-II. Based on the Coomassie, **L6R1** had modified at least 50% of CA-II.

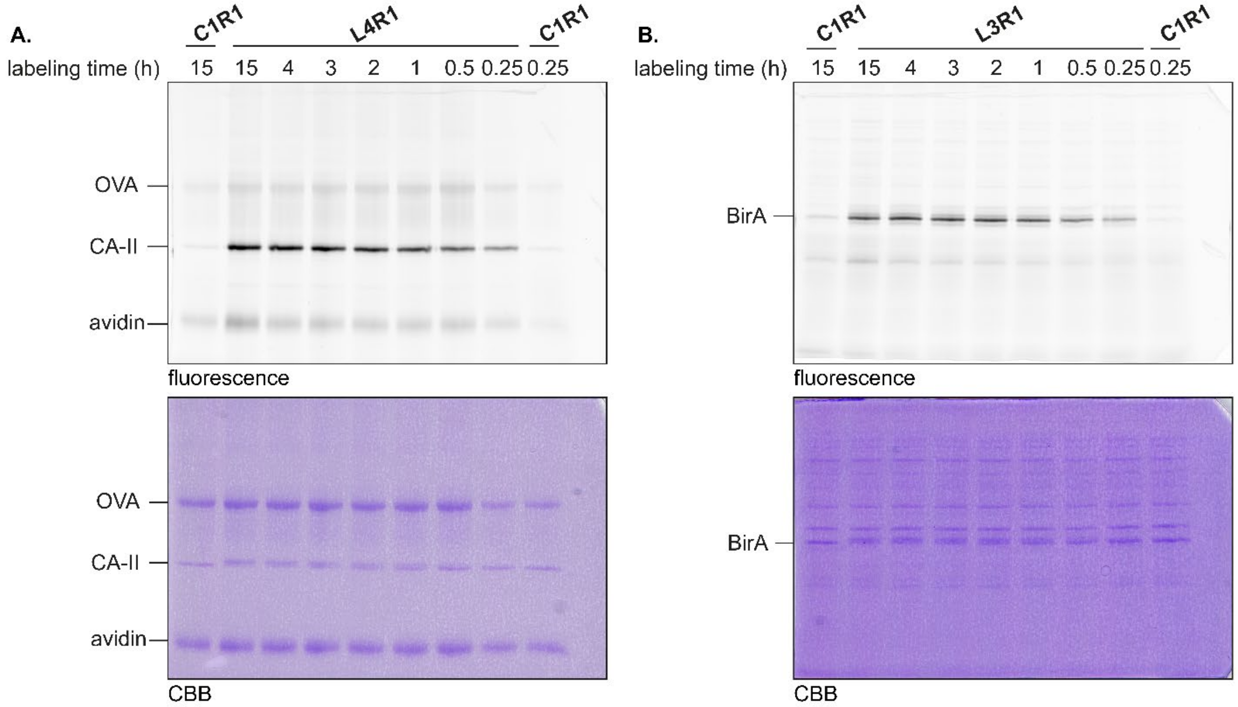


**Figure S12**. Determination of the optimal labeling time for reactive group **R1** using CA-II (**A.**) and BirA (**B.**) as model proteins. The effect of the labeling time was assessed by varying the incubation time of the target proteins (5 μM CA-II with 25 μM avidin and 25 μM ovalbumin, or 2.5 mg/mL lysate of *E. coli* overexpressing BirA) in HEPES (50 mM, pH 8.2) with their respective probes **L4R1** and **L3R1** (final concentration probe 20 μM). The labeled proteins were visualized by transimination with three equivalents of **FITC am-zide** at pH 5.3 for 2 hours, separation of the proteins with SDS-PAGE and subsequent in-gel fluorescence scanning. In case of labeling of CA-II by **L4R1**, maximal signal seems to be achieved after 4 h. However, the difference with the signals after 3 h and 2 h is minimal. In case of labeling of BirA by **L3R1**, maximal signal seems to be achieved after 2 h.

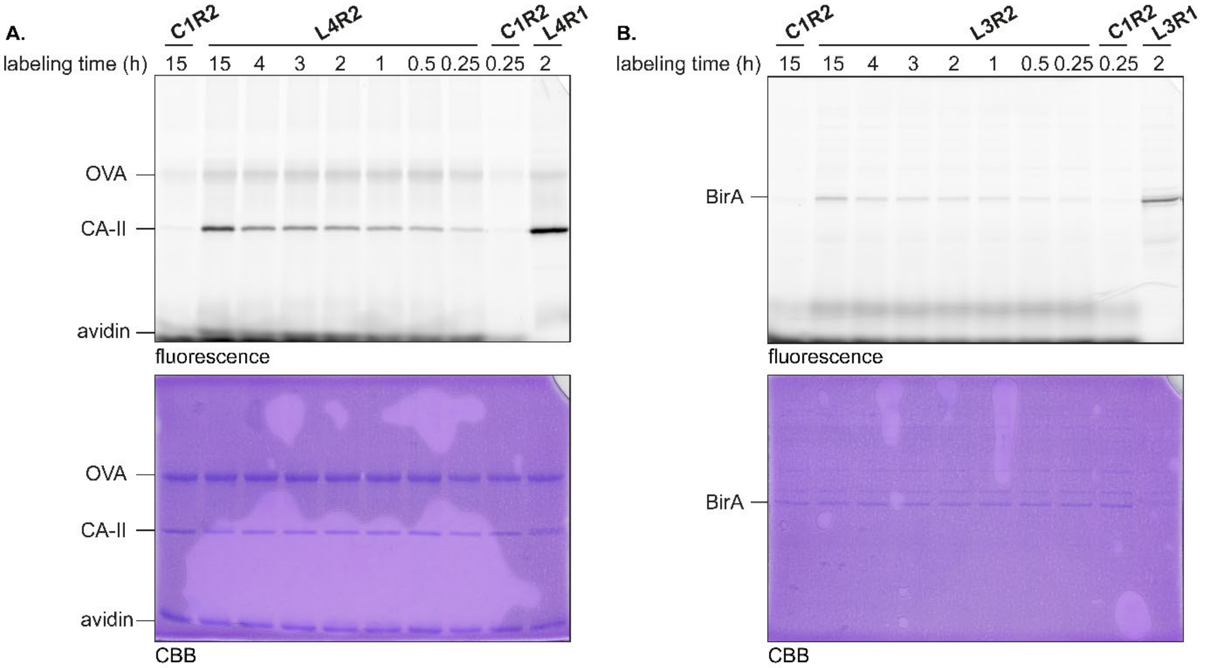


**Figure S13**. Determination of the optimal labeling time for reactive group **R2** using CA-II (**A.**) and BirA (**B.**) as model proteins. The effect of the labeling time was assessed by varying the incubation time of the target proteins (5 μM CA-II with 25 μM avidin and 25 μM ovalbumin, or 2.5 mg/mL lysate of *E. coli* overexpressing BirA) in HEPES (50 mM, pH 8.2) with their respective probes **L4R2** and **L3R2** (final concentration probe 20 μM). The labeled proteins were visualized by transimination with three equivalents of **FITC am-zide** at pH 5.3 for 2 hours, separation of the proteins with SDS-PAGE and subsequent in-gel fluorescence scanning. In both cases, maximal labeling intensity is achieved after 15 h.

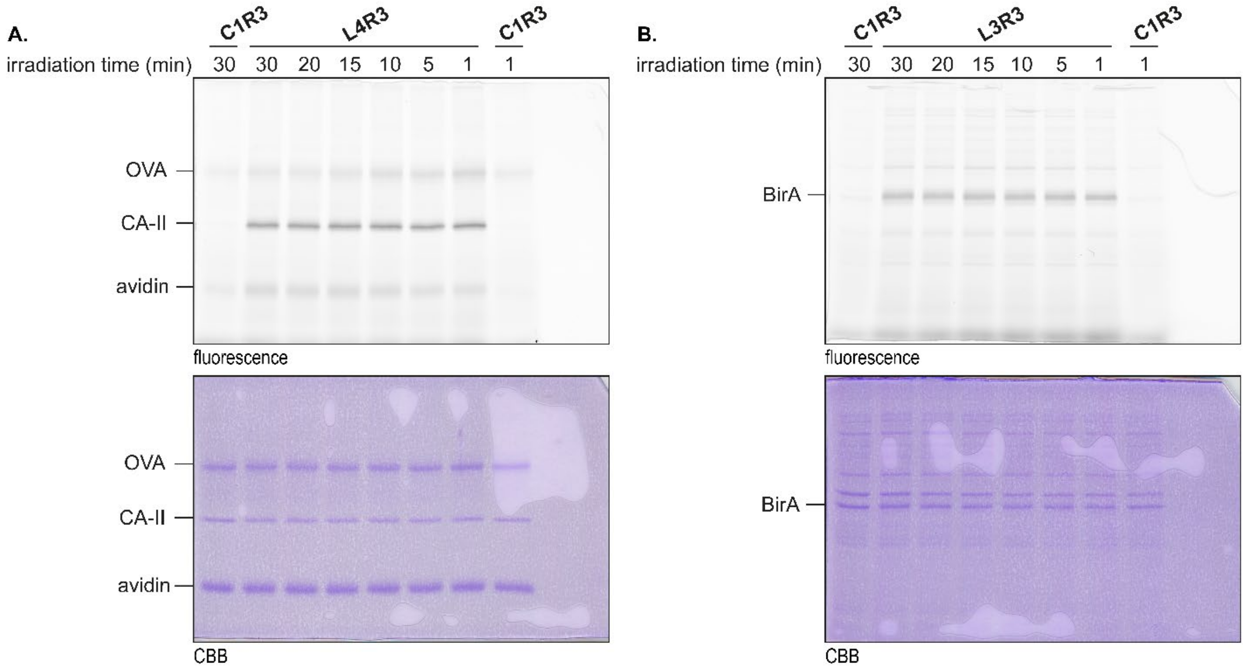


**Figure S14**. Determination of the optimal irradiation time for reactive group **R3** using CA-II (**A.**) and BirA (**B.**) as model proteins. The labeling efficiency of probes **L4R3** and **L3R3** was assessed by irradiating their respecting target proteins (5 μM CA-II with 25 μM avidin and 25 μM ovalbumin, or 2.5 mg/mL lysate of *E. coli* overexpressing BirA) in HEPES (50 mM, pH 8.2) in the presence of the probes (20 μM) for different times at 312 nm. The labeled proteins were subjected to transamination with three equivalents of **FITC am-zide** at pH 5.3 for 2 hours, separated on an SDS-PAGE and visualized by in-gel fluorescence scanning. In both cases, labeling can already be observed after 1 minute of irradiation. However, the background at that point seemed to be a bit higher. At 15 minutes or longer, the labeling efficiency seemed to be maximal while background is minimal.

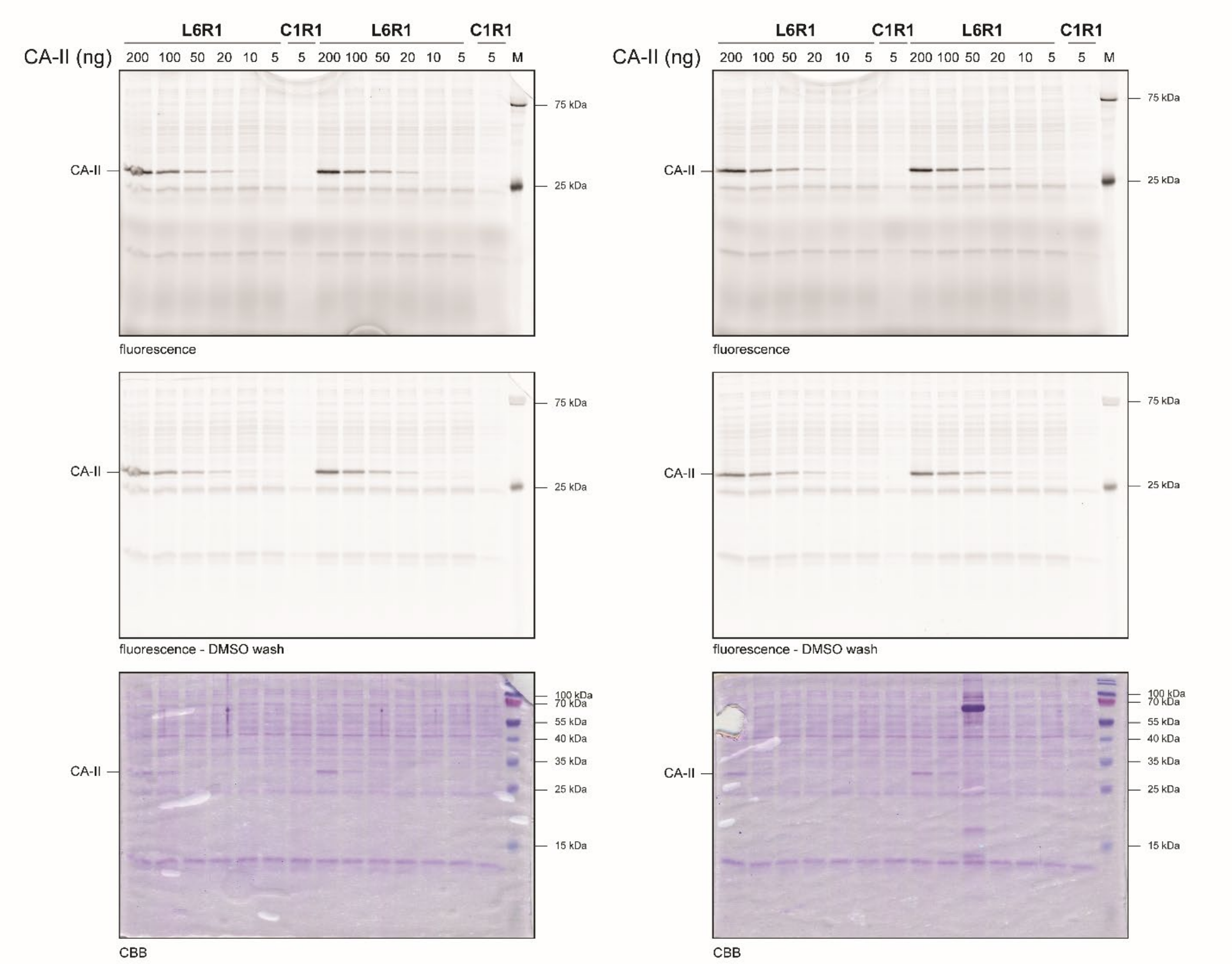


**Figure S15A.** Labeling of CA-II spiked in *E. coli* lysate with iminoboronate probe **L6R1**. Different amounts of CA-II were spiked in *E. coli* lysate in order to determine the sensitivity of **L6R1** in a complex mixture. The spiked *E. coli* lysate (2 mg/mL) was labeled with 20 μM **L6R1** for 2 hours and visualized by exchange with three equivalents of **FITC am-zide** at pH 5.3 for 2 hours. Using these conditions, 20 ng of CA-II (4 μg/mL) could be detected. However, off-target labeling of other proteins was observed, which was likely caused by an excess of probe. We also observed background signal caused by **FITC am-zide**, but this could be remedied by washing the gels overnight in DMSO.

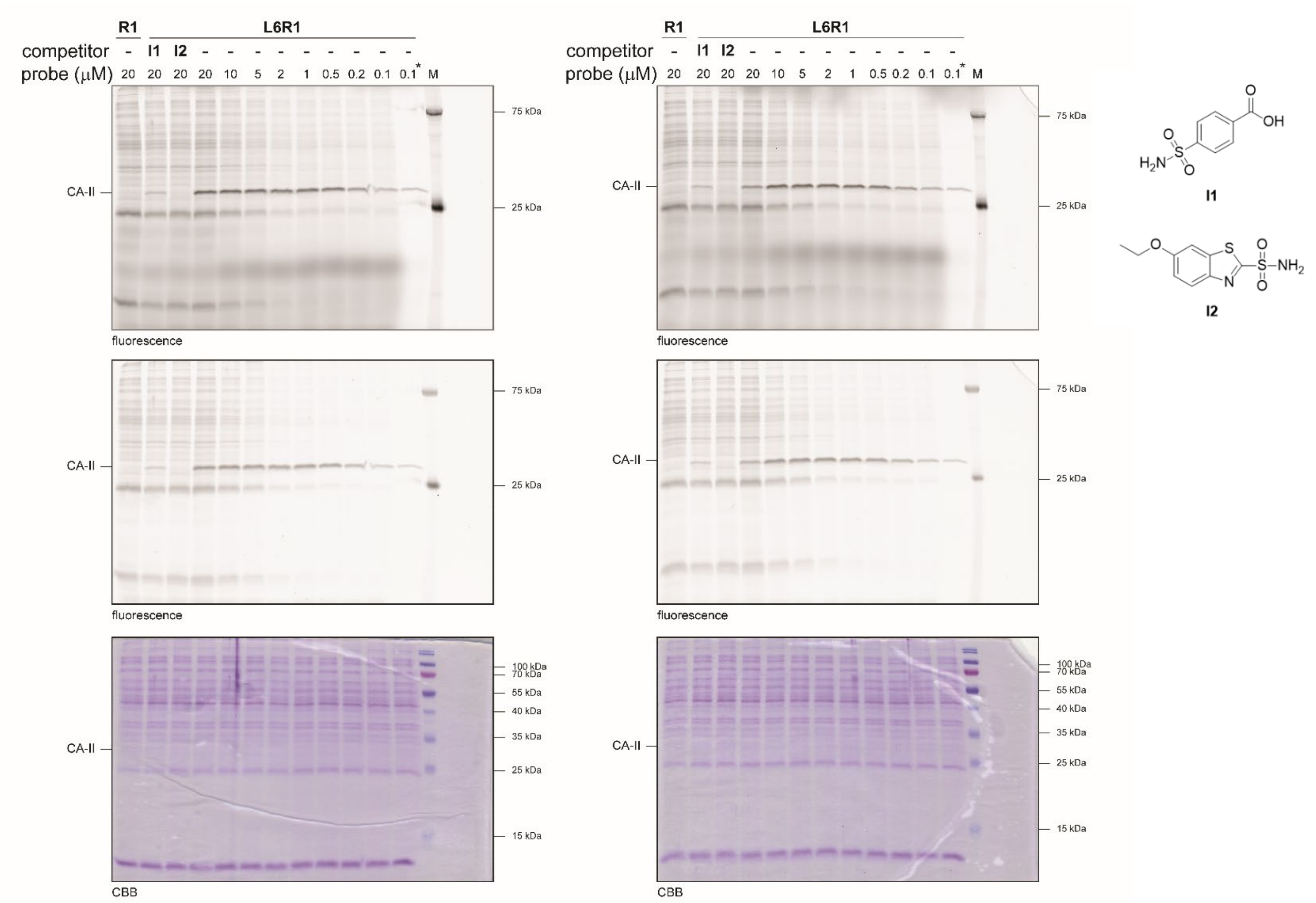


**Figure S15B.** Influence of competitors and probe concentration on the labeling of CA-II spiked in *E. coli* lysate by **L6R1**. To investigate if the off-target labeling in the *E. coli* lysate was ligand-directed, we treated *E. coli* lysate (2 mg/mL) spiked with CA-II (20 ng, or 4 μg/mL) with 100 equivalents of CA-II inhibitors 4-sulfamoylbenzoic acid **I1** or 6-ethoxy-2-benzothiazolesulfonamide **I2** prior to labeling with 20 μM **L6R1**. Addition of the competitor strongly decreases the fluorescent signal (**I1**) or completely abolishes it (**I2**), indicating that labeling of CA-II with **L6R1** is ligand-dependent. In contrast, off-target labeling was not diminished, indicating it was caused by non-specific labeling by the sulfonyl fluoride reactive group. Therefore, we lowered the amount of probe, hoping it would decrease off-target labeling. Indeed, when the concentration of **L6R1** was 1 μM or lower, off-target labeling was strongly diminished or even no longer observed. To deal with the background signal from **FITC am-zide**, we also once tested the labeling with 0.1 μM probe and 200× less **FITC am-zide** than under regular conditions (0.5 μL of 3 μM **FITC am-zide** instead of 0.5 μL of 600 μM **FITC am-zide**), as denoted with * in the figure. Gratifyingly, we could still observe labeling of CA-II was, but no off-target labeling, nor background signal from **FITC am-zide**.

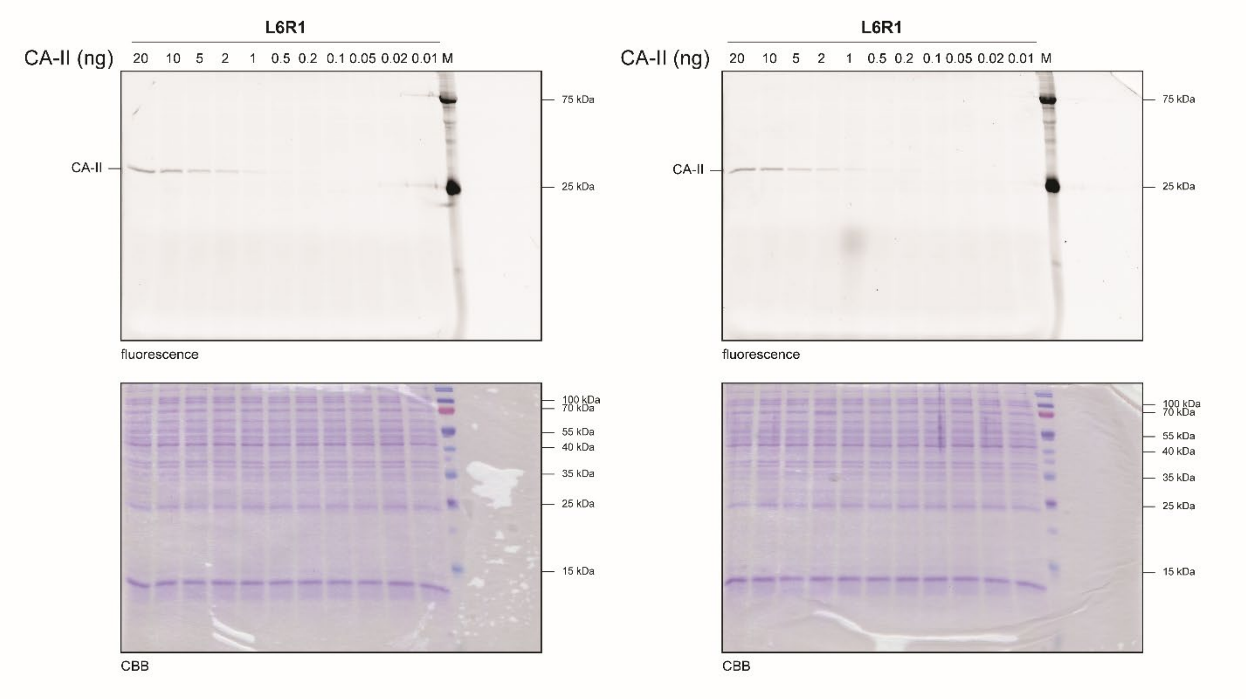


**Figure S15C.** Labeling of different amounts of CA-II, spiked in *E. coli* lysate, with 0.1 μM **L6R1** and visualization with 30 equivalents **FITC am-zide** (0.5 μL of 3 μM **FITC am-zide**). Since there was no interfering off-target labeling or background from the fluorophore, it was possible to detect as low as 1–2 ng CA-II (0.2–0.4 μg/mL) in 10 μg *E. coli* lysate (2 mg/mL).

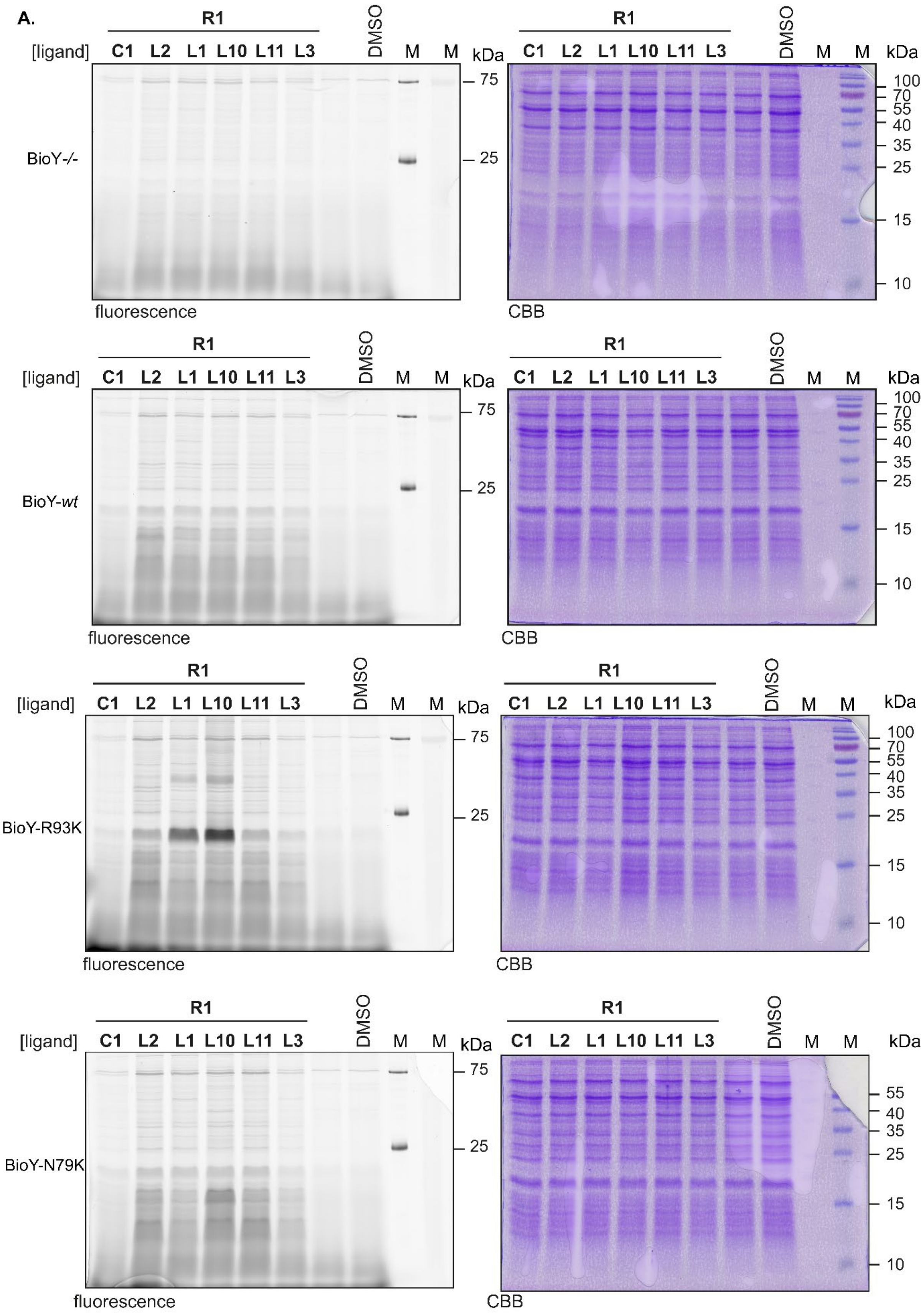

A.
R1
[ligand]
C1 L2 L1 L10 L11 L3
DMSO
M M kDa
75
25
BioY-/-
fluorescence
CBB
100
70
55
40
35
25
15
10
BioY-wt
BioY-R93K
BioY-N79K

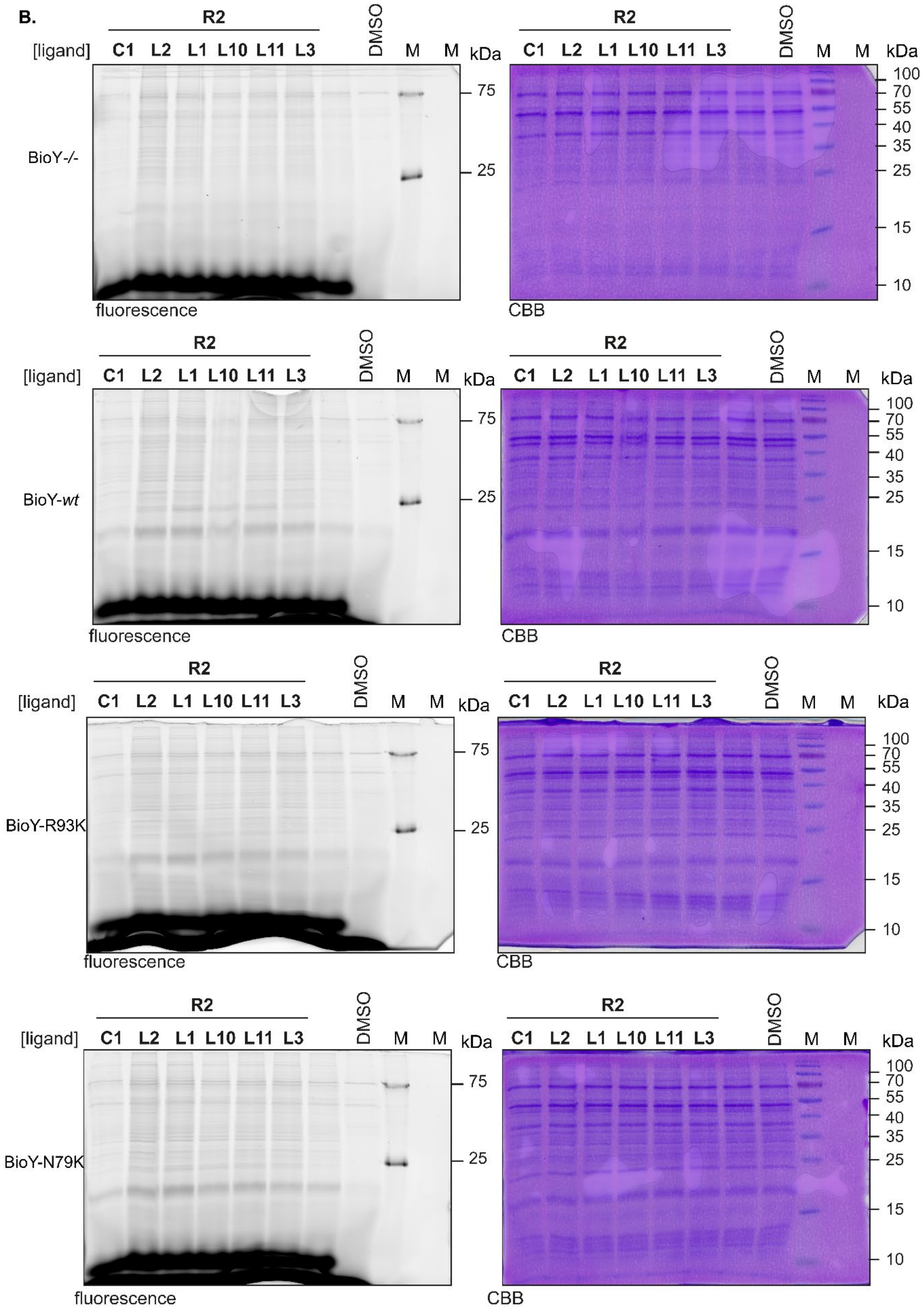
B.
R2
[ligand]
C1
L2
L1
L10
L11
L3
DMSO
M
M
kDa
75
25
100
70
55
40
35
15
10
BioY-/-
BioY-wt
BioY-R93K
BioY-N79K
fluorescence
CBB

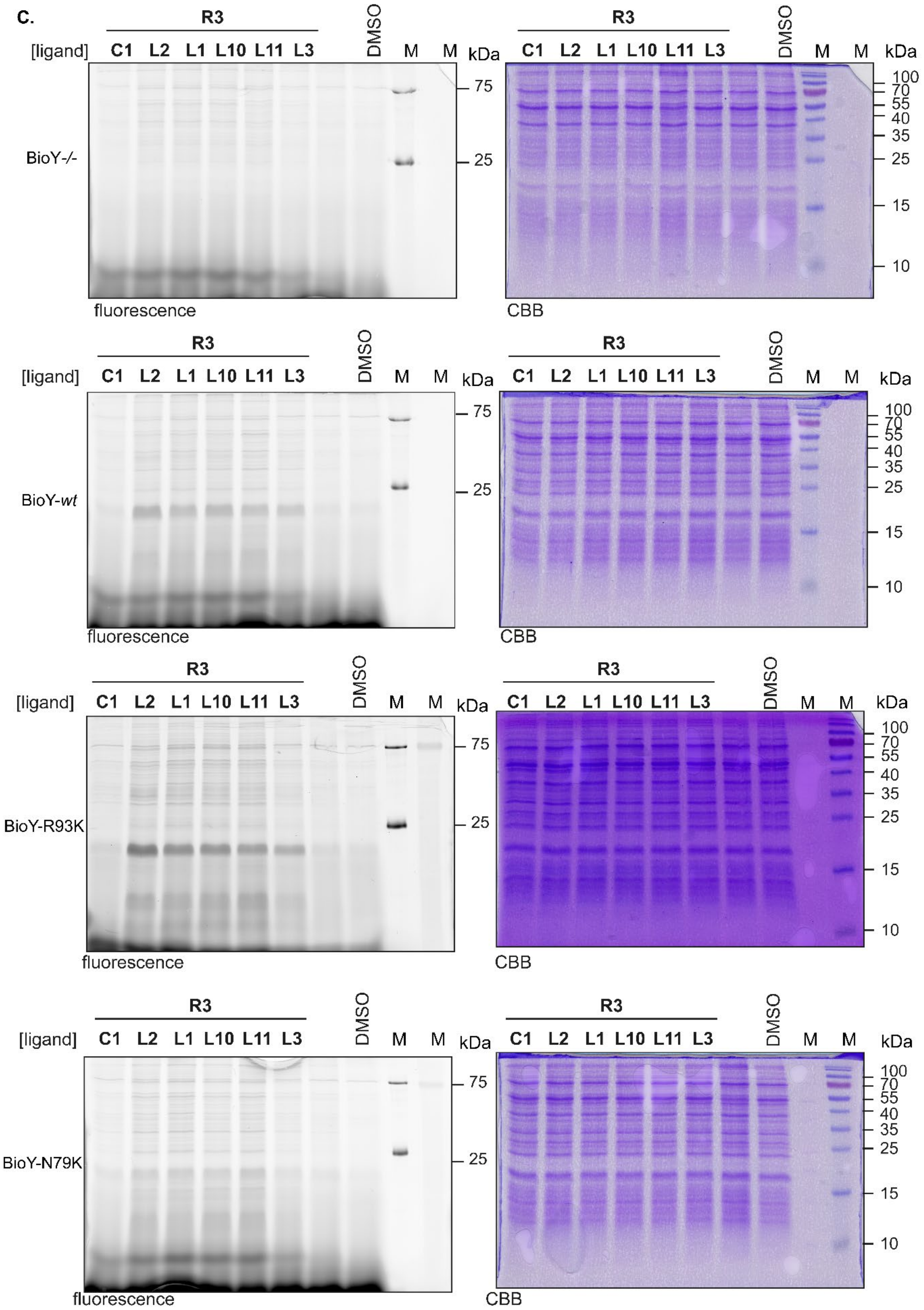
C.
R3
[ligand]
C1 L2 L1 L10 L11 L3
DMSO
M M
kDa
75
25
100
70
55
40
35
25
15
10
BioY-/-
BioY-wt
BioY-R93K
BioY-N79K
fluorescence
CBB

**Figure S16**. Screening of probes prepared from sulfonyl fluoride **R1** (**A.**), epoxide **R2** (**B.**) and photocrosslinker **R3** (**C.**) against BioY. The membrane fraction of BioY-overexpressing *Lactococcus lactis* (2 mg/mL) in HEPES (50 mM, pH 8.2) was treated with the iminoboronate probes (final concentration 20 μM) with the optimized labeling or irradiation time to find the most efficient probe, that could then be used in cell labeling studies. As a control, the membrane fraction of cells where overexpression was not induced was used. Furthermore, the mutants BioY-N79K and BioY-R93K, that have an extra lysine within the binding pocket, were also screened against. The labeled proteins were subjected to transimination with three equivalents of **FITC am-zide** at pH 5.3 for 2 hours.

Most efficient labeling of BioY (20.5 kDa) was achieved with **L1R1** and **L10R1** on BioY-R93K. The linker length-dependency that was observed indicates the need for proper positioning of the reactive group towards a suitable amino acid. In almost all cases an off-target was observed at higher molecular weight (about 75 kDa).

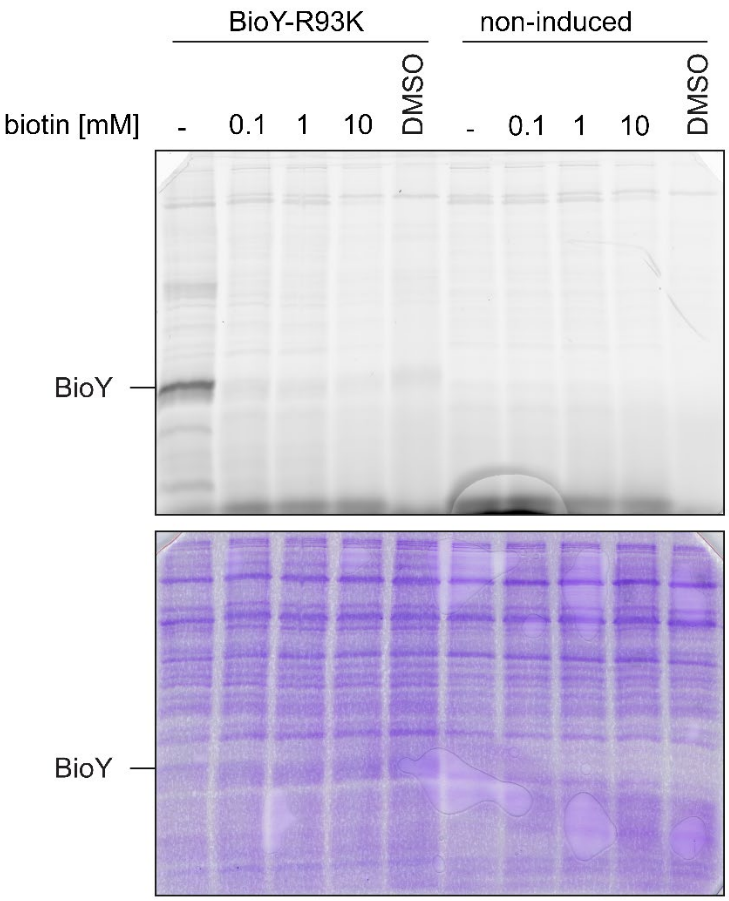


**Figure S17**. Competition of **L10R1** BioYR93K labeling with biotin. Increasing amounts of biotin were added to BioY lysate (2 mg/mL) in HEPES (50 mM, pH 8.2) before labeling with probe **L10R1** (20 μM) for 2 hours. The labeled proteins were subjected to transimination with three equivalents of **FITC am-zide** at pH 5.3 for 2 hours, separated on SDS-PAGE and visualized by in-gel fluorescence scanning. Full gels belonging to **Figure 4B** in the main paper. Already with a small excess of biotin, the fluorescent signal from **L10R1** labeling is strongly diminished, indicating ligand-dependent labeling. The labeling of the off-target at higher molecular weight was only decreased at a very high excess of biotin, which shows that labeling of this off-target is very likely not ligand dependent.

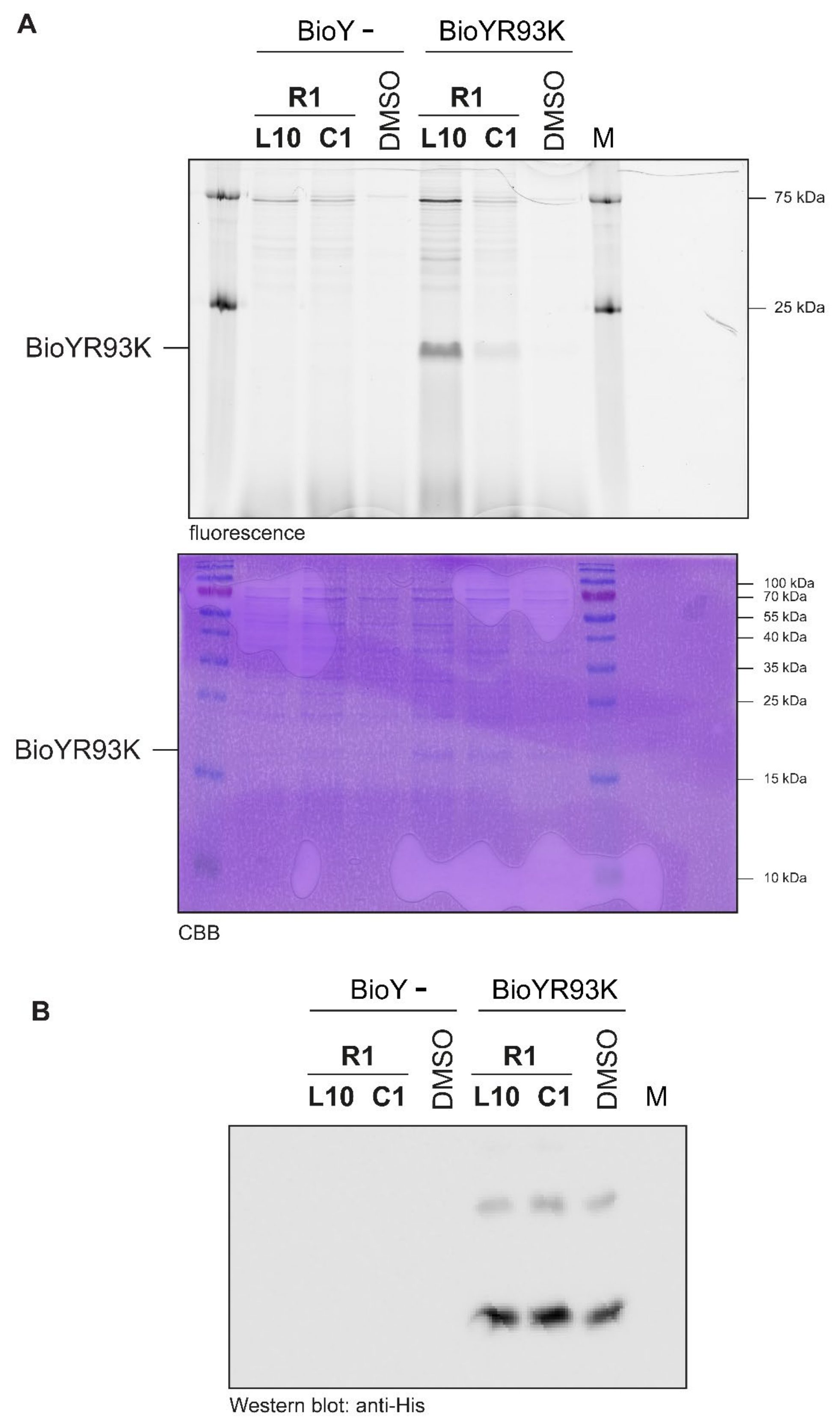
A
BioY -
BioYR93K
R1
L10
C1
DMSO
R1
L10
C1
DMSO
M
75 kDa
25 kDa
BioYR93K
fluorescence
100 kDa
70 kDa
55 kDa
40 kDa
35 kDa
25 kDa
15 kDa
10 kDa
BioYR93K
CBB
B
BioY -
BioYR93K
R1
L10
C1
DMSO
R1
L10
C1
DMSO
M
Western blot: anti-His

**Figure S18.** On cell labeling of His-tagged BioYR93K with **L10R1**. Full gels belonging to **Figure 4**C in the main paper. In-gel fluorescence of *L. lactis* cells that were not induced or overexpressing mutant BioY-R93K. Cells were labelled for 2 h with the indicated probe and the ligand was subsequently exchanged on the cell surface by **FITC am-zide**. Half of the sample was subjected to SDS-PAGE and analyzed by in-gel fluorescence scanning followed by coomassie brilliant blue staining (**A.**). The remaining part of the sample was resolved on SDS-PAGE, subsequently transferred to a PVDF membrane and analyzed immunoblotting of the His-tag (**B.**)

# Biochemical procedures

## *proteins*

Streptavidin (Strp) and avidin (Avi) were purchased from Thermo Fisher (catalog numbers 434302 and 21121, respectively). Bovine carbonic anhydrase II (CA-II) was purchased from Serva and chicken egg ovalbumin (OVA) was purchased from Sigma-Aldrich. Lysate of BirA-overexpressing *E. coli* BL21-CodonPlus(DE3)-RIL (16 mg/mL) was produced as described previously.[1] Stock solutions of Strp (277.8 µM monomer), Avi (277.8 µM monomer), CA-II (111.1 µM) and OVA (277.8 µM) were prepared in HEPES (50 mM, pH 8.2, 150 mM NaCl) and stored at -20 °C. For the labeling experiments, the concentrations of the protein mixtures were 27.78 µM Strp, 5.55 µM CA-II and 27.78 µM OVA in HEPES (50 mM, pH 8.2, 150 mM NaCl) for the Strp/CA-II/OVA mixture and 27.78 µM avidin, 5.55 µM CA-II and 27.78 µM OVA in HEPES (50 mM, pH 8.2, 150 mM NaCl) for the avidin/CA-II/OVA mixture. The concentration of the BirA lysate mixture was 2.5 mg/mL in HEPES (50 mM, pH 8.2, 150 mM NaCl).

## *Expression of His-tagged BioY wild-type and mutants in Lactococcus lactis*

His-tagged wild-type BioY and the N79K- and R93K-mutants were expressed in *L. lactis* as described previously with minor modifications.[2–4] Briefly, *L. lactis* NZ9000 cells[5], transformed with pNZnHis-BioY or mutated plasmids, were grown semi-anaerobically in 10 mL pre-cultures in M17 medium (supplemented with 1.0% (w/v) of glucose and 5 µg/mL of chloramphenicol) at 30 °C without shaking. 1 L main cultures were started by diluting the pre-culture 100× into chemically defined medium[2] without biotin and supplemented with 0.8 % (w/v) of glucose and 5 µg/mL of chloramphenicol in 1 L flasks at 30 °C without shaking. Expression was induced when an OD of around 0.25 was reached using 0.05% (v/v) of culture supernatant from a nisin-A producing strain. Expression was done for 3 hours and afterwards cells were harvested (12 min 7460 ×g, 4 °C). Cells were washed once with cold 50 mM KPi, pH 7.5, and the pellet was frozen using liquid nitrogen and stored at -80 °C.

## *Preparation of membrane fractions*

Cells were thawed and resuspended in 50 mM KPi, pH 7.5, 200 µM PMSF, 1 mM $MgSO_4$, supplemented with ~50 µg DNase per mL and lysed using a high pressure cell lyser HPL6 (Maximator, Nordhausen, Germany), at 30 kPa for 2 cycles. Cell debris was removed by centrifugation (30 min, 38.400 ×g, 4 °C) and the membrane fractions were collected by ultra-centrifugation (180 min, 186.000 ×g, 4 °C). The membrane fractions were resuspended in 50 mM KPi, pH 7.5, then frozen using liquid nitrogen and stored at -80 °C.

## *SDS-PAGE, fluorescence scanning, Western Blotting*

Laemmli sample buffer (4×) contained Tris (pH 6.8, 1M, 10 mL), water (10 mL), SDS (4 g) bromophenol blue (100 mg), glycerol (20 mL) and β-mercaptoethanol (10 mL). Labeling experiments on Strp and avidin were resolved on 12% TRIS-tricine type SDS-PAGE gel[6], while labeling experiments on CA-II and BirA were

resolved on 12% or 15% Laemmli-type SDS-PAGE gel and labeling experiments on BioY were resolved on 15% Laemmli-type SDS-PAGE gel. Gels were prepared using acrylamide-bis ready-to-use solution 40% (37.5:1) (Bio-Rad) and separated on a Mini-PROTEAN Tetra cell (Bio-Rad). In-gel fluorescence scanning of the SDS-PAGE gels was performed on a Typhoon FLA 9500 (GE Healthcare) using the FITC-settings for FITC ((laser excitation at 473 nm and ≥510 nm long pass emission filter. Western Blot was performed using a Mini-PROTEAN Tetra cell (Bio-Rad), using standard procedures. Visualization after electroblotting was performed using monoclonal polyhistidine peroxidase-conjugated antibody (Sigma-Aldrich, A7058), at 1:6,000 dilution, and treatment with SuperSignal West Pico PLUS Chemiluminescent Substrate (Thermo Fisher). After fluorescent scanning of the gel, the proteins were stained with Coomassie brilliant blue R250 according to standard protocols.

*Probes and bio-reagents*

Biotin hydrazide **L2** was purchased from Combi-Blocks (catalog numbers QB-7914). FAM-alkyne was purchased from Lumiprobe (B41B0). Biotin alkoxyamine **L3** was purchased from Thermo Fisher. The protein ladders used during SDS-PAGE were purchased from Thermo Fisher Scientific (PageRuler™ Prestained Protein Ladder, 10 to 180 kDa; 26617) and Bio-Rad (Precision Plus Protein™ Dual Color Standards; 1610374). All ligands and reactive groups were prepared as stock solutions (50 mM) in DMSO and stored at -20 °C, except for biotin alkoxyamine **L3** (40 mM). In order to generate the probes, ligand stock (1 µL) was mixed with an equimolar amount of the reactive group (1 µL) and incubated at room temperature overnight. Subsequently, the probes were diluted in DMSO until the desired concentration was obtained for biochemical testing. The probe solutions were stored at -20 °C and could be used for at least a month before fresh probe solutions had to be made again. A stock solution of SDS (20% w/v) was prepared in water and stored at room temperature. A stock solution of THPTA/$CuSO_4$ (20 mM) was prepared in water and stored at -20 °C. Solutions of sodium ascorbate (20 mM) in water were always prepared fresh from the salt. Stock solutions of FAM-alkyne (5 mM) and **FITC am-zide** (5 mM) were prepared in DMSO and stored at -20 °C. Click mixtures for CuAAC reactions were prepared as follows: FAM-alkyne (16 µL, 5 mM), DMSO (48 µL), THPTA/$CuSO_4$ (20 µL, 20 mM) and water (20 µL) were added together in this exact order, followed by addition of sodium ascorbate (20 µL, 20 mM; freshly prepared). This click mixture was added to the protein sample. UV irradiation was performed with a Spectroline ENB-280C/FE UV lamp (312 nm).

*Procedure for the labeling experiments and visualization with FAM-alkyne*

Probe (1 µL of 200 µM) or DMSO (1 µL) was incubated with the protein mixture (9 µL Strp/CA-II/OVA, Avi/CA-II/OVA or BirA lysate) for 2 h. SDS (0.5 µL of 20%) was added and the samples were heated at 100 °C for 15 minutes. The samples were shortly centrifuged with a mini centrifuge, followed by addition of the FAM-alkyne click mixture (5 µL) and incubation for 2 h. Laemmli sample buffer (5 µL of 4×) was added, the samples were heated at 100 °C for 15 minutes, 10.3 µL per sample was loaded on SDS-PAGE gel, resolved and analyzed by in-gel fluorescence.

*Initial procedure for the labeling experiments and visualization with* ***FITC am-zide***

Probe (1 μL of 200 μM) or DMSO (1 μL) was incubated with protein mixture (9 μL Strp/CA-II/OVA, Avi/CA-II/OVA or BirA lysate) for 2 h. SDS (0.5 μL of 20% w/v solution in water) was added and the samples were heated at 100 °C for 15 minutes. The samples were shortly centrifuged with a mini centrifuge, followed by addition of **FITC am-zide** (1 μL of 1 mM) and incubation for 2 h. Laemmli sample buffer (11.5 μL of 2× stock) was added, the samples were heated at 100 °C for 15 minutes, 11.5 μL per sample was loaded on SDS-PAGE gel, resolved and analyzed by in-gel fluorescence.

*Optimization for the labeling experiments and visualization with* ***FITC am-zide***

*Optimization of the concentration of* ***FITC am-zide***: Probe (1 μL of 200 μM) was incubated with protein mixture (9 μL Strp/CA-II/OVA, Avi/CA-II/OVA or BirA lysate) for 2 h. SDS (0.5 μL of 20% w/v solution in water) was added and the samples were heated at 100 °C for 15 minutes. The samples were shortly centrifuged with a mini centrifuge, followed by addition of **FITC am-zide** (1 μL of different concentrations as indicated) and incubation for 2 h. Laemmli sample buffer (11.5 μL of 2× stock) was added, 11.5 μL per sample was loaded on SDS-PAGE gel, resolved and analyzed by in-gel fluorescence.

*Influence of the pH on the transimination*: Probe (1 μL of 200 μM) was incubated with protein mixture (9 μL Strp/CA-II/OVA, Avi/CA-II/OVA or BirA lysate) for 2 h. SDS (0.5 μL of 20% w/v solution in water) was added and the samples were heated at 100 °C for 15 minutes. The samples were shortly centrifuged with a mini centrifuge, followed by addition of **FITC am-zide** (1 μL of 600 μM), acetic acid (1 μL of different concentrations as indicated in the table below) and incubation for 2 h. Laemmli sample buffer (12.5 μL of 2× stock) was added, 12.5 μL per sample was loaded on SDS-PAGE gel, resolved and analyzed by in-gel fluorescence.

| 1 μL of [M] acetic acid | - | 0.1 | 0.2 | 0.3 | 0.4 | 0.5 | 0.6 | 0.7 | 0.8 | 0.9 | 1.0 |
|---|---|---|---|---|---|---|---|---|---|---|---|
| Final pH of the exchange solution | 8.2 | 7.8 | 7.4 | 6.9 | 5.5 | 5.3 | 5.1 | 5.0 | 4.9 | 4.8 | 4.7 |

*Optimization of the time of transimination*: Probe (1 μL of 200 μM) was incubated with protein mixture (9 μL Strp/CA-II/OVA, Avi/CA-II/OVA or BirA lysate) for 2 h. SDS (0.5 μL of 20% w/v solution in water) was added and the samples were heated at 100 °C for 15 minutes. The samples were shortly centrifuged with a mini centrifuge, followed by addition of **FITC am-zide** (1 μL of 600 μM), acetic acid (1 μL of 500 mM) and incubation for different times as indicated. Laemmli sample buffer (12.5 μL of 2× stock) was added, 12.5 μL per sample was loaded on SDS-PAGE gel, resolved and analyzed by in-gel fluorescence.

*Probe formation time*

Ligand (0.5 μL of 400 μM) and reactive group (0.5 μL of 400 μM) were added together at different time points as indicated. Then, protein mixture (9 μL Strp/CA-II/OVA, Avi/CA-II/OVA or BirA lysate) was added

and the samples were incubated for 2 h. SDS (0.5 μL of 20% w/v solution in water) was added and the samples were heated at 100 °C for 15 minutes. The samples were shortly centrifuged with a mini centrifuge, followed by addition of **FITC am-zide** (1 μL of 600 μM), acetic acid (1 μL of 500 mM) and incubation for 2 h. Laemmli sample buffer (12.5 μL of 2× stock) was added, 12.5 μL per sample was loaded on SDS-PAGE gel, resolved and analyzed by in-gel fluorescence.

*Probe formation in the presence of protein*

Ligand (0.5 μL of 400 μM) and reactive group (0.5 μL of 400 μM), or pre-formed probe (1 μL of 200 μM) were added to protein mixture (9 μL Strp/CA-II/OVA, Avi/CA-II/OVA or BirA lysate) and left to incubate for different times as indicated. SDS (0.5 μL of 20% w/v solution in water) was added and the samples were heated at 100 °C for 15 minutes. The samples were shortly centrifuged with a mini centrifuge, followed by addition of **FITC am-zide** (1 μL of 600 μM), acetic acid (1 μL of 500 mM) and incubation for 2 h. Laemmli sample buffer (12.5 μL of 2× stock) was added, 12.5 μL per sample was loaded on SDS-PAGE gel, resolved and analyzed by in-gel fluorescence.

*Screening of ligands and reactive groups.*

*Optimization of labeling with **R1** and **R2***: Probe (1 μL of 200 μM) or DMSO (1 μL) was incubated with protein mixture (9 μL Strp/CA-II/OVA, Avi/CA-II/OVA or BirA lysate) for different times as indicated. SDS (0.5 μL of 20% w/v solution in water) was added and the samples were heated at 100 °C for 15 minutes. The samples were shortly centrifuged with a mini centrifuge, followed by addition of **FITC am-zide** (1 μL of 600 μM), acetic acid (1 μL of 500 mM) and incubation for 2 h. Laemmli sample buffer (12.5 μL of 2× stock) was added, 12.5 μL per sample was loaded on SDS-PAGE gel, resolved and analyzed by in-gel fluorescence.

*Optimization of labeling with **R3***: Probe (1 μL of 200 μM) or DMSO (1 μL) was incubated with protein mixture (9 μL Strp/CA-II/OVA, Avi/CA-II/OVA or BirA lysate) for 40 minutes and subsequently irradiated at 312 nm for different times as indicated. SDS (0.5 μL of 20% w/v solution) was added and the samples were heated at 100 °C for 15 minutes. The samples were shortly centrifuged with a mini centrifuge, followed by addition of **FITC am-zide** (1 μL of 600 μM), acetic acid (1 μL of 500 mM) and incubation for 2 h. Laemmli sample buffer (12.5 μL of 2× stock) was added, 12.5 μL per sample was loaded on SDS-PAGE gel, resolved and analyzed by in-gel fluorescence.

*Screening of ligands and reactive group*: Probe (1 μL of 200 μM) or DMSO (1 μL) was incubated with protein mixture (9 μL Strp/CA-II/OVA for screening on Strp, Avi/CA-II/OVA for screening on AVI and CA-II, or BirA lysate) for 2 h (**R1** and **R3**) or 15 h (**R2**). Samples with **R3** were irradiated after 30 minutes of incubation at 312 nm for 15 minutes. SDS (0.5 μL of 20% w/v solution in water) was added and the samples were heated at 100 °C for 15 minutes. The samples were shortly centrifuged with a mini centrifuge, followed by addition of **FITC am-zide** (1 μL of 600 μM), acetic acid (1 μL of 500 mM) and incubation for 2 h. Laemmli sample buffer (12.5 μL of 2× stock) was added, 12.5 μL per sample was loaded on SDS-PAGE gel, resolved and analyzed by in-gel fluorescence.

*Labeling of CA-II spiked in E. coli lysate with **L6R1***

Probe (0.5 μL, 10× the final concentrations indicated) was incubated with *E. coli* lysate (4.5 μL of 2.22 mg/mL) that was spiked with CA-II (amount of ng as indicated) in HEPES (50 mM, pH 8.2) for 2 h. SDS (0.25 μL of 20% w/v solution in water) was added and the samples were heated at 100 °C for 15 minutes. The samples were shortly centrifuged, followed by addition of **FITC am-zide** (0.5 μL of 600 μM or 30 μM, as indicated) and acetic acid (0.5 μL of 500 mM), and the samples were incubated for 2 h. Laemmli sample buffer (6.5 μL of 2× stock) was added, the samples were loaded on SDS-PAGE gel, resolved and analyzed by in-gel fluorescence.

*Labeling of the membrane fraction of His-tagged BioY lysate*

Probe (0.5 μL of 200 μM) or DMSO (0.5 μL) was incubated with the membrane fraction of lysate of BioY-overexpressing *L. lactis* (4.5 μL of 2.22 mg/mL) for 2h. 30 minutes after the start of incubation, samples with **R3** were irradiated at 312 nm for 15 minutes. SDS (0.25 μL of 20% w/v solution in water) was added, followed by addition of **FITC am-zide** (0.5 μL of 600 μM) and acetic acid (0.5 μL of 500 mM), and the samples were incubated for 2 h. Laemmli sample buffer (6.5 μL of 2× stock) was added, the samples were loaded on SDS-PAGE gel, resolved and analyzed by in-gel fluorescence.

*Biotin competition*: Biotin (0.5 μL of 1 mM, 10 mM or 100 mM) was incubated with the membrane fraction of lysate of BioY-overexpressing *L. lactis* (4.5 μL of 2.22 mg/mL) for 1 h. **L10R1** (0.5 μL of 200 μM) or DMSO (0.5 μL) were added and the samples were further incubated for 2 h. SDS (0.25 μL of 20% w/v solution in water) was added, followed by addition of **FITC am-zide** (0.5 μL of 600 μM) and acetic acid (0.5 μL of 500 mM), and the samples were incubated for 2 h. Laemmli sample buffer (6.5 μL of 2× stock) was added, the samples were loaded on SDS-PAGE gel, resolved and analyzed by in-gel fluorescence.

*Labeling of His-tagged BioY-R93K-overexpressing Lactococcus lactis*

**L10R1** (10 μL of 5 mM), **C1R1** (10 μL of 5 mM) or DMSO (10 μL) was added to a suspension of *L. lactis* cells over expressing His-tagged BioY-R93K(0.5 mL of OD 50) in HEPES (50 mM, pH 8.2) and the samples were incubated under constant shaking for 2 h. The samples were centrifuged at 12.000 RCF for 1 minute, the supernatant was discarded and the cells were washed with HEPES (0.5 mL, 2×). The cells were resuspended in HEPES (0.5 mL), followed by addition of acetic acid (10 μL of 5 M) and **FITC am-zide** (10 μL of 6 mM). The samples were incubated in the dark, under constant shaking, for 2 h. The supernatant was removed as before and the cells were washed with HEPES (0.5 mL, 3×). The cell pellet was stored at -20 °C overnight.

The cell pellet was dissolved in 30 mL lysis buffer (50 mM KPi pH 7.5, 5 mM $MgSO_4$, 200 μM PMSF, supplemented with 1.5 mg DNAse) and the cells were lysed using a high-pressure cell lyser HPL6 (maximator, Nordhausen, Germany), at 30 KPs for 2 cycles. The lysate was centrifuged at 16.000 rpm for 30 minutes, the supernatant was isolated and centrifuged at 40.000 rpm for 180 minutes. The supernatant was removed and the remaining pellet of the membrane fraction was dissolved in Laemmli sample buffer

(50 μL of 2.5×). Due to some water still being present in the centrifugation flasks, the total volume was now approximately 150 μL per sample. Two aliquots were taken from each sample (5 μL) and to this was added additional Laemmli sample buffer (5 μL of 2× stock). One set of the aliquots was loaded on SDS-PAGE gel, resolved and analyzed by in-gel fluorescence, while the other set was also loaded on SDS-PAGE gel and resolved, but then transferred to a membrane using standard Western Blotting techniques and visualized using monoclonal polyhistidine peroxidase-conjugated antibody (Sigma-Aldrich, A7058), at 1:6,000 dilution, and treatment with SuperSignal West Pico PLUS Chemiluminescent Substrate (Thermo Fisher).

# Synthetic procedures

## *General synthetic procedures*

All solvents used were of commercial grade and used without further purification. Unless reported otherwise, reagents were purchased from Sigma-Aldrich, TCI, Fisher Scientific or Combi-blocks and were used without further purification, or were synthesized according to literature procedures. TLC was performed on Merck TLC Silica gel 60/Kieselguhr F254 plates and visualization was done by UV light, ninhydrin, potassium permanganate, phosphomolybdic acid or dinitrophenylhydrazine staining solutions. Manual column chromatography was performed using silica (SilicaFlash P60, 230-400 mesh, Silicycle, Canada) as the stationary phase. Reverse phase flash chromatography was performed using a Reveleris X2 flash chromatography system purchased from Büchi. $^{1}$H-, $^{11}$B, $^{13}$C-, $^{19}$F-, APT and HSQC NMR were recorded on a Varian AMX400 spectrometer on a Bruker Avance NEO 600, using acetonitrile-$d_3$, chloroform-*d*, DMSO-$d_6$, deuterium oxide or methanol-$d_4$ as solvent. Chemical shift values are reported in ppm with the solvent resonance as the internal standard (acetonitrile-$d_3$: δ 1.94 for $^{1}$H, δ 1.32 for $^{13}$C; chloroform-*d*: δ 7.26 for $^{1}$H, δ 77.16 for $^{13}$C; methanol-$d_4$: δ 3.31 for $^{1}$H, δ 49.00 for $^{13}$C; DMSO-$d_6$: δ2.50 for $^{1}$H δ 39.52 for $^{13}$C; $D_2O$: δ 4.79 for $^{1}$H). Data are reported as follows: chemical shifts (δ), multiplicity (s = singlet, d = doublet, dd = double doublet, ddd = double double doublet, t = triplet, q = quartet, p = quintet, m = multiplet, apparent quartet = app q), coupling constants J (Hz), and integration. High resolution mass measurements were performed using a ThermoScientific LTQ OrbitrapXL spectrometer using methanol as eluent.

## *Synthesis of 2-formylphenylboronic acid reactive group derivatives **R1***

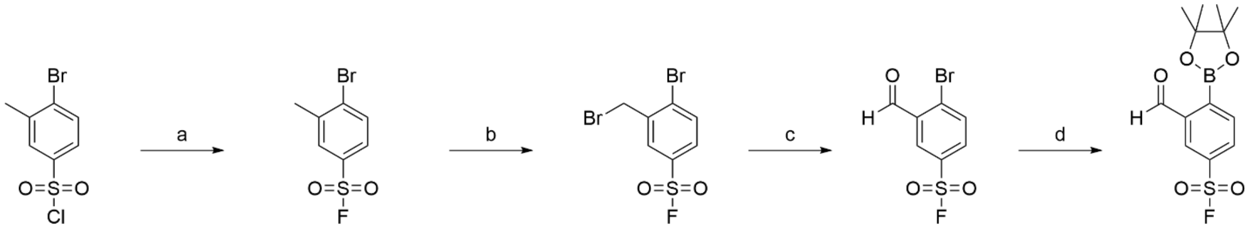


1 2 3 R4 R1

a) $KHF_2$, $H_2O$, $CH_3CN$ (95%); b) NBS, AIBN, benzene, reflux (77%); c) NMO, propionitrile, 60 °C (51%); d) $B_2pin_2$, KOAc, Pd(dppf)$Cl_2$, 1,4-dioxane, reflux (37%).

4-bromo-3-methylbenzenesulfonyl fluoride (**2**)

4-bromo-3-methylbenzenesulfonyl chloride **1** (1.0 g, 3.7 mmol) dissolved in acetonitrile (4.2 mL) was added to a solution of $KHF_2$ (669 mg, 8.6 mmol) in water (1.9 mL) in a 50 mL plastic tube under vigorously stirring. After 2h, the reaction mixture was diluted with water (20 mL), extracted with EtOAc (15 mL, 3×), the combined organic layers were washed with brine (30 mL, 1×), dried over $Na_2SO_4$, filtered over cotton and concentrated under reduced pressure to yield **2** (890 mg, 3.5 mmol, 95%) as a white solid. $^{1}$H NMR (400 MHz, Chloroform-*d*) δ 7.86 (d, J = 2.3 Hz, 1H), 7.82 – 7.78 (m, 1H), 7.68 (dd, J = 8.4, 2.3 Hz, 1H), 2.52 (s, 3H). $^{13}$C NMR (101 MHz, Chloroform-*d*) δ 140.7, 134.0, 133.8, 132.2, 132.0, 130.2, 127.0, 23.2. $^{19}$F NMR

(376 MHz, Chloroform-*d*) δ 66.30. *HRMS measurements were carried out, but none of the attempted methods (ESI positive, APCI positive, and ESI negative) succeeded in ionization of* ***2****.*

4-bromo-3-(bromomethyl)benzenesulfonyl fluoride (**3**)

4-bromo-3-methylbenzenesulfonyl fluoride **2** (854 mg, 3.4 mmol), NBS (779 mg, 4.4 mmol) and AIBN (103 mg, 0.63 mmol) were dissolved in benzene (18 mL) under nitrogen atmosphere. The reaction mixture was heated at reflux for 15h, then more AIBN (155 mg, 0.94 mmol) was added and the mixture was heated at reflux for another 7h. The mixture was cooled to room temperature and filtered over cotton. The filtrate was diluted with toluene (80 mL), washed with $NaHCO_3$ (saturated, 60 mL, 2×) and brine (60 mL, 1×), dried over $Na_2SO_4$, filtered over cotton and concentrated under reduced pressure. Purification by column chromatography (dry-loaded on celite, 0% -> 1% ether/pentane) yielded 4-bromo-3-(bromomethyl)benzenesulfonyl fluoride **3** (867 mg, 2.6 mmol, 77%) as a white solid with 87% purity. The remaining 13% impurity was the dibrominated species. $^1$H NMR (400 MHz, Chloroform-*d*) δ 8.08 (d, J = 2.3 Hz, 1H), 7.87 (dd, J = 8.4, 0.9 Hz, 1H), 7.79 (dd, J = 8.5, 2.2 Hz, 1H), 4.63 (s, 2H). $^{13}$C NMR (101 MHz, Chloroform-*d*) δ 139.7, 135.1, 134.7, 133.1, 131.2, 130.8, 129.9, 129.5, 129.3, 37.0, 31.2. $^{19}$F NMR (376 MHz, Chloroform-*d*) δ 66.48. *HRMS measurements were carried out, but none of the attempted methods (ESI positive, APCI positive, and ESI negative) succeeded in ionization of* ***3****.*

4-bromo-3-formylbenzenesulfonyl fluoride **R4**

A solution of 3 (786 mg, 2.4 mmol) and NMO (577 mg, 4.9 mmol)) in propionitrile (25 mL) was stirred at 60 °C for 2h and then at room temperature for 3h. The reaction mixture was diluted with EtOAc (100 mL), washed with water (70 mL, 1×), HCl (1M, 70 mL, 2×) and brine (70 mL, 1×), dried over $Na_2SO_4$, filtered and concentrated under reduced pressure. Purification by column chromatography (dry-loaded on celite, 2% -> 4% diethylether/pentane) yielded 4-bromo-3-formylbenzenesulfonyl fluoride **R4** (322 mg, 1.2 mmol, 51%) as a white solid. $^1$H NMR (400 MHz, Chloroform-*d*) δ 10.40 (s, 1H), 8.51 (d, J = 2.4 Hz, 1H), 8.04 (dd, J = 8.4, 2.4 Hz, 1H), 7.97 (d, J = 8.5 Hz, 1H). $^{13}$C NMR (101 MHz, Chloroform-*d*) δ 189.1, 135.9, 134.8, 134.5, 133.5, 130.0. $^{19}$F NMR (376 MHz, Chloroform-*d*) δ 66.18. HRMS (ESI-orbitrap) m/z calculated for $[M+CH_3]^+$ 280.9278, found 280.9277. *Note: this methyl adduct is formed due to the use of methanol as solvent for the HRMS. In situ hemiacetal formation followed by elimination of water results in the methyl oxonium species.*

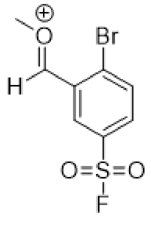


Methyl oxonium species of 4-bromo-3-formylbenzenesulfonyl fluoride (**4**)

3-formyl-4-(4,4,5,5-tetramethyl-1,3,2-dioxaborolan-2-yl)benzenesulfonyl fluoride (**R1**)

4-bromo-3-formylbenzenesulfonyl fluoride **4** (311 mg, 1.2 mmol), bis(pinacolato)diboron (464 mg, 1.8 mmol), potassium acetate (386 mg, 3.9 mmol) and Pd(dppf)$Cl_2$ (36 mg, 0.049 mmol) were added to degassed anhydrous 1,4-dioxane (18 mL) and heated at reflux for 3½ h. The reaction mixture was cooled to room temperature, dry-loaded onto celite and purified by column chromatography (30% EtOAc/pentane). The fractions containing the product as determined by NMR were concentrated under reduced pressure and then triturated with pentane (NMR showed that the dioxaborolane peak (around 1.26 ppm) disappeared while product peaks were still present) to yield 35 mg white solid. The filtrate was concentrated under reduced pressure and triturated another two times. Finally, the remaining crude was recrystallized from heptane and combined with the triturated fractions to in total yield 3-formyl-4-(4,4,5,5-tetramethyl-1,3,2-dioxaborolan-2-yl)benzenesulfonyl fluoride **R1** (136 mg, 0.43 mmol, 37%) as an off-white solid. $^{1}$H NMR (400 MHz, Chloroform-*d*) δ 10.61 (s, 1H), 8.55 (d, J = 1.8 Hz, 1H), 8.19 – 8.11 (m, 2H), 1.42 (s, 12H). $^{13}$C NMR (101 MHz, Chloroform-*d*) δ 192.0, 142.4, 137.2, 135.9, 135.6, 131.3, 127.5, 85.6, 25.0. $^{19}$F NMR (376 MHz, Chloroform-*d*) δ 65.65. $^{11}$B NMR (128 MHz, Chloroform-*d*) δ 30.45. HRMS (ESI-orbitrap) m/z calculated for $[M+CH_3O]^-$ 345.0974, found 345.0998. *Note: the methoxyboronate anion is observed in the negative mode measurements due to the use of methanol as the solvent for sample preparation for the HRMS.*

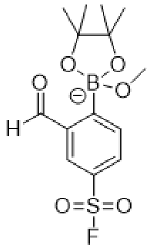


Methoxyboronate anion of 3-formyl-4-(4,4,5,5-tetramethyl-1,3,2-dioxaborolan-2-yl)benzenesulfonyl fluoride (**R1**)

*Synthesis of 2-formylphenylboronic acid reactive group derivatives **R2***

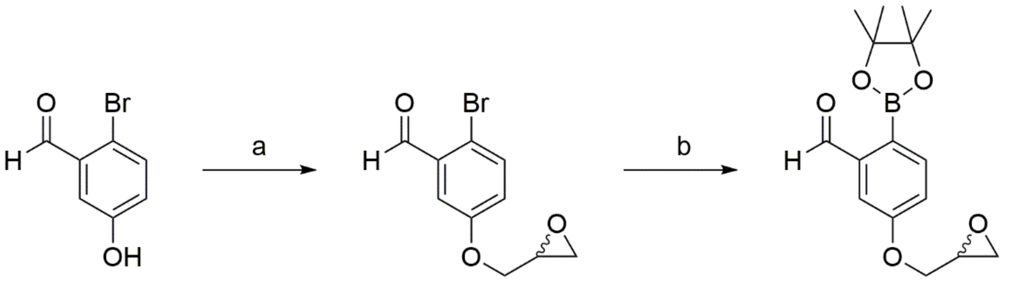


**5** **6** **R2**

a) epibromohydrin, $Cs_2CO_3$, DMF (75%); b) $B_2pin_2$, KOAc, Pd(dppf)$Cl_2$, 1,4-dioxane, reflux (79%)

2-bromo-5-(oxiran-2-ylmethoxy)benzaldehyde (**6**)

2-bromo-5-hydroxybenzaldehyde **5** (200 mg, 1.0 mmol) and cesium carbonate (974 mg, 3.0 mmol) were dissolved in DMF (10 mL). After 35 minutes, epibromohydrin (0.6 mL, 7.0 mmol) was added and the reaction mixture was allowed to stir at room temperature overnight. After 19 h, the mixture was diluted

with water, extracted with diethyl ether (2×) and the combined organic layers were washed with water (2×) and brine (2×), dried over $Na_2SO_4$, filtered over cotton and concentrated under reduced pressure. Purification by column chromatography (dry-loaded on celite, 20% EtOAc/pentane) yielded 2-bromo-5-(oxiran-2-ylmethoxy)benzaldehyde **6** (192 mg, 0.75 mmol, 75%) as a light-pink solid. $^{1}$H NMR (400 MHz, Chloroform-*d*) δ 10.28 (s, 1H), 7.52 (d, J = 8.8 Hz, 1H), 7.40 (d, J = 3.3 Hz, 1H), 7.07 (dd, J = 8.8, 3.2 Hz, 1H), 4.31 (dd, J = 11.1, 2.7 Hz, 1H), 3.93 (dd, J = 11.0, 5.9 Hz, 1H), 3.35 (m, 1H), 2.91 (t, J = 4.5 Hz, 1H), 2.75 (dd, J = 4.9, 2.6 Hz, 1H). $^{13}$C NMR (101 MHz, Chloroform-d) δ 191.7, 158.3, 134.8, 134.1, 123.7, 118.6, 113.5, 69.4, 49.9, 44.6. $[M+CH_3]^+$ 270.9964, found 270.9964. *Note: this methyl adduct is formed due to the use of methanol as solvent for the HRMS. In situ hemiacetal formation followed by elimination of water results in the methyl oxonium species.*

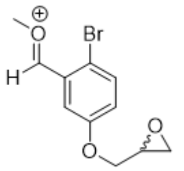


Methyl oxonium species of 2-bromo-5-(oxiran-2-ylmethoxy)benzaldehyde (**6**)

5-(oxiran-2-ylmethoxy)-2-(4,4,5,5-tetramethyl-1,3,2-dioxaborolan-2-yl)benzaldehyde (**R2**)

In a dried Schlenk flask, 2-bromo-5-(oxiran-2-ylmethoxy)benzaldehyde **6** (100 mg, 0.39 mmol), bis(pinacolato)diboron (129 mg, 0.51 mmol), Pd(dppf)$Cl_2$ (14 mg, 0.019 mmol) and potassium acetate (114.8 mg, 1.170 mmol, 3.007 eq.) were added to degassed anhydrous 1,4-dioxane (3.9 mL) and heated at reflux for 1.5 h. After complete conversion was observed by $^{1}$H-NMR spectroscopy, the reaction mixture was allowed to cool down to room temperature. The mixture was concentrated under reduced pressure, dry-loaded on celite and purified by column chromatography (15% EtOAc/pentane) to yield 5-(oxiran-2-ylmethoxy)-2-(4,4,5,5-tetramethyl-1,3,2-dioxaborolan-2-yl)benzaldehyde **R2** (93 mg, 0.31 mmol, 79%) as a yellow oil. $^{1}$H NMR (400 MHz, Chloroform-d) δ 10.66 (s, 1H), 7.87 (d, J = 8.3 Hz, 1H), 7.49 (d, J = 2.7 Hz, 1H), 7.16 (dd, J = 8.3, 2.7 Hz, 1H), 4.34 (dd, J = 11.0, 2.9 Hz, 1H), 4.00 (dd, J = 11.1, 5.8 Hz, 1H), 3.37 (m, 1H), 2.92 (t, J = 4.5 Hz, 1H), 2.77 (dd, J = 4.9, 2.6 Hz, 1H), 1.36 (s, 12H). $^{13}$C NMR (101 MHz, Chloroform-d) δ 194.8, 160.9, 143.7, 138.2, 120.6, 111.0, 84.4, 68.9, 50.0, 44.7, 25.0. $^{11}$B NMR (128 MHz, Chloroform-d) δ 30.75. HRMS (ESI-orbitrap) m/z calculated for $[M+H]^+$ 305.1555, found 305.1558.

*Synthesis of 2-formylphenylboronic acid reactive group derivatives **R3***

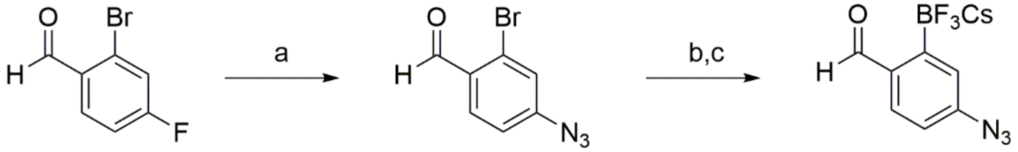


**7** **8** **R3**

a) $NaN_3$, DMF, 90 °C (80%); b) $B_2pin_2$, KOAc, Pd(dppf)$Cl_2$, 1,4-dioxane, reflux; c) CsF, L-tartaric acid, $CH_3CN$, THF (24% over two steps).

4-azido-2-bromobenzaldehyde (**8**)

A mixture of 2-bromo-4-fluorobenzaldehyde **7** (1.13 g, 5.6 mmol) and sodium azide (428 mg, 6.6 mmol) in anhydrous DMF (13 mL) was heated to 90 °C under nitrogen atmosphere for 16 h. The mixture was cooled to room temperature, filtered over cotton and concentrated under reduced pressure. The crude was dissolved in ether (120 mL) and washed with $NaHCO_3$ (saturated aqueous, 80 mL, 2×) and brine (80 mL, 1×), dried over $Na_2SO_4$, filtered over cotton and concentrated under reduced pressure. Purification by column chromatography (dry-loaded on celite, 1% ether/pentane) yielded 4-azido-2-bromobenzaldehyde **8** (1.0 g, 4.4 mmol, 80%) as a white solid. $^1$H NMR (400 MHz, Chloroform-*d*) δ 10.23 (s, 1H), 7.89 (d, J = 8.4 Hz, 1H), 7.25 (d, J = 2.2 Hz, 1H), 7.12 – 6.96 (m, 1H). $^{13}$C NMR (101 MHz, Chloroform-*d*) δ 190.3, 147.0, 131.4, 130.3, 128.4 123.9, 118.6. IR: $\tilde{v}$ = 1585 (vs), 1692 (s), 2117 (vs) ($N_3$). *HRMS measurements were carried out, but none of the attempted methods (ESI positive, APCI positive, and ESI negative) succeeded in ionization of **8**.*

4-azido-2-(trifluoro-λ4-boraneyl)benzaldehyde cesium salt (**R3**)

A mixture of 4-azido-2-bromobenzaldehyde **8** (227 mg, 1.0 mmol), bis(pinacolato)diboron (331 mg, 1.3 mmol), Pd(dppf)$Cl_2$ (35 mg, 0.05 mmol), potassium acetate (299 mg, 3.0 mmol) and anhydrous 1,4-dioxane (4 mL) was heated at reflux for 5 h. The reaction mixture was filtered over Celite, diluted with dichloromethane (80 mL), washed with water (50 mL, 3×) and brine (70 mL, 1×), dried over $Na_2SO_4$, filtered over cotton and concentrated under reduced pressure. The crude was redissolved in acetonitrile, followed by addition of a solution of cesium fluoride (604 mg, 4.0 mmol) in water (0.4 mL). After 10 minutes, a mixture of L-tartaric acid (311 mg, 2.1 mmol) in THF (1.5 mL) was added dropwise and the resulting suspension was stirred for 10 minutes. Subsequently, the reaction mixture was diluted with acetonitrile (4 mL), filtered over cotton, the residue washed with acetonitrile (10 mL, 3x) and the combined filtrate was concentrated under reduced pressure. Trituration with ether (30 mL), then trituration with chloroform (50 mL) yielded 4-azido-2-(trifluoro-λ4-boraneyl)benzaldehyde cesium salt **R3** (82 mg, 0.24 mmol, 24%, 3% impurity in the form of $CsBF_4$ based on $^{19}$F NMR) as a brown solid. $^1$H NMR (400 MHz, Acetonitrile-*d₃*) δ 10.44 (s, 1H), 7.80 (d, J = 8.4 Hz, 1H), 7.37 (s, 1H), 6.90 (d, J = 8.2 Hz, 1H). $^{13}$C NMR (101 MHz, Acetonitrile-$d_3$) δ 196.5, 144.5, 137.9, 137.7, 128.1, 123.5 (q, J = 3.6 Hz), 118.0. $^{19}$F NMR (376 MHz, Acetonitrile-$d_3$) δ -135.05 (dd, J = 99.8, 47.4 Hz). $^{11}$B NMR (128 MHz, Acetonitrile-$d_3$) δ 2.87 (q, J = 51.9 Hz). HRMS (ESI-orbitrap) m/z calculated for $[M]^-$ 214.0394, found 214.0409. IR: $\tilde{v}$ = 2123 ($N_3$).

Cesium tetrafluoroborate impurity: $^{19}$F NMR (376 MHz, Acetonitrile-$d_3$) δ -150.98 (d, J = 5.6 Hz). $^{11}$B NMR (128 MHz, Acetonitrile-$d_3$) δ -1.19.

*Synthesis of ligands **L4-L9***

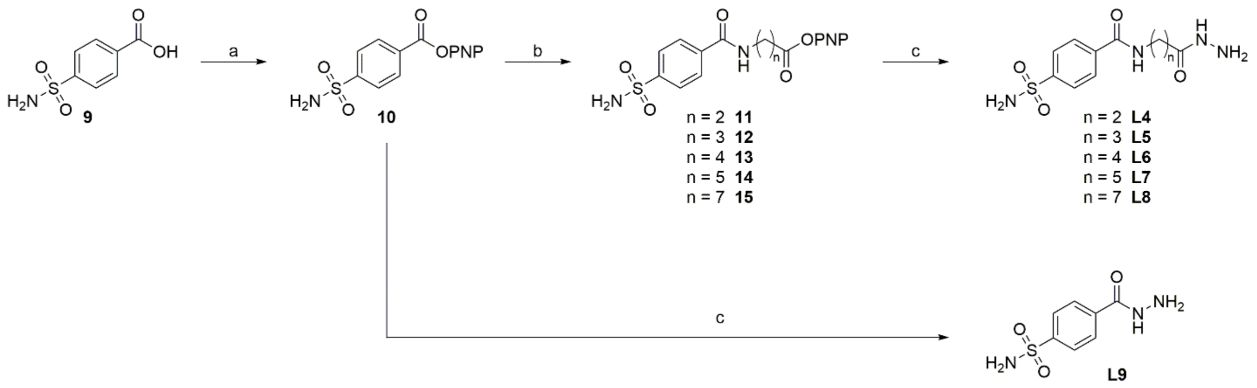

a) EDC.HCl, *p*-nitrophenol, DMF (78%); b) amino acid, EDC.HCl, DMF, $H_2O$ (**13** 62%; **14** %; **15** %; **16** 69%; **17** %); c) hydrazine hydrate, ethanol, DMF (**L4** 55%; **L5** 40%; **L6** 81%; **L7** 31%; **L8** 62%; **L9** 41%)

4-nitrophenyl 4-sulfamoylbenzoate (**10**)

4-Sulfamoylbenzoic acid **9** (2.01 g, 10 mmol) and *N*-(3-dimethylaminopropyl)-*N'*-ethylcarbodiimide hydrochloride (EDC. HCl) (2.30 g, 12 mmol) were suspended in anhydrous DMF (50 mL). After 1 h at room temperature under constant stirring, *p*-nitrophenol (1.53 g, 11 mmol) was added and the mixture was stirred at room temperature for 24 h. The reaction volume was reduced to about one-tenth by warming under reduced pressure. Then HCl (1 M, 100 mL) was stirred into the concentrate. The resulting precipitate was filtered over a glass filter and successively washed with HCl (1 M, 50 mL), water (50 mL) and ether (50 mL). The precipitate was dried under high vacuum and subsequently recrystallized from ethanol to yield 4-nitrophenyl 4-sulfamoylbenzoate **10** (2.51 g, 78%) as white crystals. $^1$H NMR (400 MHz, methanol-$d_4$) δ H = 8.37 (d, J = 9.1 Hz, 2H), 8.36 (d, J = 8.4 Hz, 2H), 8.10 (d, J=8.5, 2H), 7.56 (d, J = 9.1 Hz, 2H). $^{13}$C NMR (101 MHz, methanol-$d_4$) δ C = 164.5, 156.9, 150.1, 147.1, 133.2, 131.9, 127.6, 126.2, 124.0. Elemental analysis [Found: C, 48.1; H, 3.2; N, 8.9%. $C_{13}H_{10}N_2O_6S$ calculated: C, 48.4; H, 3.1; N, 8.7%].

4-Nitrophenyl 3-(4-sulfamoylbenzamido)propanoate (**11**)

4-nitrophenyl 4-sulfamoylbenzoate **10** (1.61 g, 5.0 mmol) in anhydrous DMF (50 mL), β-alanine (0.40 g, 4.5 mmol) dissolved in water (2 mL) was added drop-wise over 6 h and the mixture was stirred at room temperature. Upon depletion of the amino acid according to TLC (24 h), EDC.HCl (1.44 g, 7.5 mmol) was added and stirred at room temperature for another 24 h. Then, the reaction volume was reduced to about one-tenth by warming under reduced pressure and HCl (1 M, 100 mL) was stirred into the concentrate. The resulting white precipitate was filtered off and successively washed with HCl (1 M, 50 mL), water (50 mL) and ether (50 mL). The resulting solid was dissolved in acetone and dry-loaded onto celite. The crude was then purified with automated flash column chromatography using a gradient from 2% to 10% methanol in dichloromethane over 30 min. The combined fractions were concentrated *in vacuo* to yield 4-nitrophenyl 3-(4-sulfamoylbenzamido)propanoate **11** (1.10 g, 62%) as pale, off-white powder. $^1$H NMR (400 MHz, DMSO-$d_6$) δ H = 8.90 (t, J = 5.5 Hz, 1 H), 8.32 (m, 2 H), 8.00 (d, J = 8.4 Hz, 2 H), 7.91 (d, J = 8.4

Hz, 2 H), 7.48 (m, 3 H), 3.66 (q, J = 6.4 Hz, 2 H), 2.93 (t, J = 6.8, 2 H). $^{13}$C NMR (101 MHz, DMSO-$d_6$) δ 169.6, 165.5, 155.4, 146.3, 145.0, 137.1, 127.9, 125.7, 125.3, 123.2, 35.5, 34.0. HRMS (ESI-orbitrap) m/z calculated for $[M+H]^+$ 394.0721, found 394.0704.

4-nitrophenyl 4-(4-sulfamoylbenzamido)butanoate (**12**)

A solution of 4-nitrophenyl 4-sulfamoylbenzoate **10** (352 mg, 1.1 mmol) in DMF (2 mL) was heated to 50 °C. Subsequently, a solution of γ-aminobutyric acid (103 mg, 1.0 mmol) in water (1 mL) was added dropwise over the course of 1 h. After stirring at 50 °C overnight, EDC.HCl (192 mg, 1.0 mmol) was added and the mixture was left stirring for another 3 h. Then, HCl (2N, 30 mL) was added and the resultant precipitate was stored overnight at 4 °C, followed by filtration over a glass filter. The residue was washed with HCl (2N, 50 mL, 3×) and diethylether (50 mL, 3×) to yield 4-nitrophenyl 4-(4-sulfamoylbenzamido)butanoate **12** (280 mg, 0.69 mmol, 69%) as a white powder. $^{1}$H NMR (400 MHz, Methanol-$d_4$) δ 8.28 (d, J = 9.0 Hz, 2H), 7.97 (m, 4H), 7.37 (d, J = 9.0 Hz, 2H), 3.54 (t, J = 6.8 Hz, 2H), 2.76 (t, J = 7.1 Hz, 2H), 2.06 (p, J = 7.0 Hz, 2H). $^{13}$C NMR (101 MHz, Methanol-$d_4$) δ 172.5, 169.0, 157.1, 147.8, 146.8, 139.0, 129.0, 127.4, 126.1, 123.9, 40.2, 32.3, 25.5. HRMS (ESI-orbitrap) m/z calculated for $[M+H]^+$ 408.0860, found 408.0855.

4-nitrophenyl 5-(4-sulfamoylbenzamido)pentanoate (**13**)

A solution of 4-nitrophenyl 4-sulfamoylbenzoate **10** (1.77 g, 5.5 mmol) in DMF (10 mL) was heated to 50 °C. Subsequently, a solution of δ-aminovaleric acid (585 mg, 5.0 mmol) in water (4 mL) was added dropwise over the course of 4 h. After stirring at 50 °C overnight, EDC.HCl (288, 1.5 mmol) was added and the mixture was left stirring at room temperature for 3 h. Then, HCl (2N, 30 mL) was added and the resultant precipitate was stored overnight at 4 °C, followed by filtration over a glass filter. The residue was washed with HCl (2N, 50 mL, 3×) and diethylether (50 mL, 3×), then further purified by column chromatography (dry-loaded on celite, 2% -> 10% methanol/dichloromethane) to yield 4-nitrophenyl 5-(4-sulfamoylbenzamido)pentanoate **13** (500 mg, 1.2 mmol, 22%) as a white solid. $^{1}$H NMR (400 MHz, DMSO-$d_6$) δ 8.69 (t, J = 5.7 Hz, 1H), 8.30 (d, J = 9.1 Hz, 2H), 7.99 (d, J = 8.5 Hz, 2H), 7.89 (d, J = 8.4 Hz, 2H), 7.47 (s, 2H), 7.44 (d, J = 9.1 Hz, 2H), 3.37 – 3.28 (m, 2H), 2.70 (t, J = 7.0 Hz, 2H), 1.76 – 1.59 (m, 4H). $^{13}$C NMR (101 MHz, DMSO-$d_6$) δ 171.2, 165.1, 155.4, 146.1, 145.0, 137.5, 127.8, 125.6, 125.3, 123.2, 33.1, 28.3, 21.7. HRMS (ESI-orbitrap) m/z calculated for $[M+H]^+$ 422.1017, found 422.1015.

4-Nitrophenyl 6-(4-sulfamoylbenzamido)hexanoate (**14**)

To a solution of 4-nitrophenyl 4-sulfamoylbenzoate **10** (1.61 g, 5.0 mmol) in anhydrous DMF (50 mL) 6-aminohexanoic acid (0.59 g, 4.5 mmol) dissolved in water (2 mL) was added drop-wise over 6 h and the mixture was then stirred at room temperature for 24 h. EDC.HCl (1.44 g, 7.5 mmol) was added and stirred at room temperature for another 24 h. Then, the reaction volume was reduced to about one-tenth by warming under reduced pressure and HCl (1 M, 100 mL) was stirred into the concentrate. The resulting white precipitate was filtered off and successively washed with HCl (1 M, 50 mL), water (50 mL) and ether (50 mL). The resulting solid was dissolved in acetone and dry-loaded onto celite. The crude was then

purified with automated flash column chromatography using a gradient from 2% to 10% methanol in dichloromethane over 30 min. The combined fractions were concentrated *in vacuo* to yield 4-nitrophenyl 6-(4-sulfamoylbenzamido)hexanoate **14** (1.35 g, 69%) as pale, off-white powder. $^{1}$H NMR (400 MHz, methanol-$d_4$) δ H = 8.28 (d, J = 9.1 Hz, 2H), 7.97 (d, J = 8.7 Hz, 2H), 7.94 (d, J = 8.9 Hz, 2H), 7.35 (d, J = 9.1 Hz, 2H), 3.43 (t, J = 7.1 Hz, 2H), 2.68 (t, J = 7.4 Hz, 2H), 1.81 (app p, J = 7.4 Hz, 2H), 1.71 (app p, J = 7.2 Hz, 2H), 1.54 (m, 2H). $^{13}$C NMR (101 MHz, DMSO-$d_6$) δ C = 171.6, 165.5, 155.8, 146.5, 145.4, 137.9, 128.2, 126.0, 125.7, 123.6, 33.8, 29.1, 26.2, 24.3. HRMS (ESI-orbitrap) m/z calculated for $[M+H]^+$ 463.1170, found 463.1167.

General procedure for the synthesis of hydrazides **L4-L7** and **L9**

Under nitrogen atmosphere, *p*-nitrophenol-activated sulfonamide **10-14** dissolved in DMF (1 mL) were dropwise added to a solution of hydrazine hydrate (50-60%; 10 equivalents, 0.1 M in ethanol) and stirred at room temperature overnight. Ether was added (30 mL) and the mixture was stirred for 2 h. Subsequently, the resulting precipitate was filtered over a glass filter, washed with ether (10 mL, 2×) and ethanol (5 mL, 1×) and dried under vacuum to yield the hydrazides **L4-L7** and **L9**. Optionally, the yields could be improved by concentrating the filtrates under reduced pressure and performing another round of precipitation with ether.

*N*-(3-hydrazineyl-3-oxopropyl)-4-sulfamoylbenzamide (**L4**)

4-Nitrophenyl 3-(4-sulfamoylbenzamido)propanoate **11** (100 mg, 0.254 mmol) was converted into the hydrazide according to the general procedure. Yielded *N*-(3-hydrazineyl-3-oxopropyl)-4-sulfamoylbenzamide (**L4**) (40 mg, 0.14 mmol, 55%) as a white solid. $^{1}$H NMR (400 MHz, DMSO-$d_6$) δ 9.04 (s, 1H), 8.70 (t, J = 5.6 Hz, 1H), 7.97 (d, J = 8.4 Hz, 2H), 7.88 (d, J = 8.4 Hz, 2H), 7.39 (s, 2H), 4.19 (s, 2H), 3.47 (td, J = 7.3, 5.6 Hz, 2H), 2.33 (t, J = 7.2 Hz, 2H). $^{13}$C NMR (101 MHz, DMSO-$d_6$) δ 169.6, 165.1, 146.2, 137.3, 127.8, 125.6, 36.2, 33.4. HRMS (ESI-orbitrap) m/z calculated for $[M+H]^+$ 287.0809, found 287.0818.

*N*-(4-hydrazineyl-4-oxobutyl)-4-sulfamoylbenzamide (**L5**)

4-nitrophenyl 4-(4-sulfamoylbenzamido)butanoate **12** (100 mg, 0.245 mmol) was converted into the hydrazide according to the general procedure. Yielded *N*-(4-hydrazineyl-4-oxobutyl)-4-sulfamoylbenzamide **L5** (29 mg, 0.097 mmol, 39%) as a white solid. $^{1}$H NMR (400 MHz, DMSO-$d_6$) δ 8.96 (s, 1H), 8.66 (t, J = 5.5 Hz, 1H), 7.98 (d, J = 8.2 Hz, 2H), 7.89 (d, J = 8.1 Hz, 2H), 7.41 (s, 2H), 4.16 (s, 2H), 3.26 (q, J = 6.6 Hz, 2H), 2.08 (t, J = 7.5 Hz, 2H), 1.75 (q, J = 7.3 Hz, 2H). $^{13}$C NMR (101 MHz, DMSO-$d_6$) δ 171.3, 165.1, 146.1, 137.5, 127.8, 125.6, 31.0, 25.1. HRMS (ESI-orbitrap) m/z calculated for $[M+H]^+$ 301.0965, found 301.0976.

*N*-(5-hydrazineyl-5-oxopentyl)-4-sulfamoylbenzamide (**L6**)

4-nitrophenyl 5-(4-sulfamoylbenzamido)pentanoate **13** (100 mg, 0.24 mmol) was converted into the hydrazide according to the general procedure. Yielded *N*-(5-hydrazineyl-5-oxopentyl)-4-

sulfamoylbenzamide **L6** (60 mg, 0.19 mmol, 81%) as a white solid. $^{1}$H NMR (400 MHz, DMSO-$d_6$) δ 8.93 (s, 1H), 8.63 (t, J = 5.6 Hz, 1H), 7.98 (d, J = 8.5, 2H), 7.88 (d, J = 8.4, 2H), 7.46 (s, 2H), 4.14 (d, J = 3.6 Hz, 2H), 3.25 (q, J = 6.3 Hz, 2H), 2.04 (t, J = 6.9 Hz, 2H), 1.52 (m, 4H). $^{13}$C NMR (101 MHz, DMSO-$d_6$) δ 171.4, 165.0, 146.1, 137.5, 127.8. 125.6, 33.1, 28.7, 22.8. HRMS (ESI-orbitrap) m/z calculated for $[M+H]^+$ 315.1122, found 315.1133.

*N*-(6-hydrazineyl-6-oxohexyl)-4-sulfamoylbenzamide (**L7**)

4-Nitrophenyl 6-(4-sulfamoylbenzamido)hexanoate **14** (100 mg, 0.23 mmol) was converted into the hydrazide according to the general procedure. Yielded *N*-(6-hydrazineyl-6-oxohexyl)-4-sulfamoylbenzamide **L7** (23 mg, 0.070 mmol, 30%) as a white solid. $^{1}$H NMR (400 MHz, DMSO-$d_6$) δ 8.90 (s, 1H), 8.61 (t, J = 6.0 Hz, 1H), 7.97 (d, J = 8.1 Hz, 2H), 7.88 (d, J = 8.1 Hz, 2H), 7.46 (s, 2H), 4.13 (d, J = 4.1 Hz, 2H), 3.25 (q, J = 6.4 Hz, 2H), 2.01 (t, J = 7.5 Hz, 2H), 1.52 (m, J = 7.5 Hz, 4H), 1.28 (m, J = 7.8 Hz, 2H). $^{13}$C NMR (101 MHz, DMSO-$d_6$) δ 171.5, 165.0, 146.1, 137.6, 127.8 125.6, 39.2, 33.4, 28.9, 28.8, 26.2, 25.0. HRMS (ESI-orbitrap) m/z calculated for $[M+H]^+$ 329.1278, found 329.1288.

*N*-(8-hydrazineyl-8-oxooctyl)-4-sulfamoylbenzamide (**L8**)

A solution of 4-nitrophenyl 4-sulfamoylbenzoate **10** (320 mg, 1.0 mmol) in DMF (10 mL) was heated to 60 °C. Subsequently, a solution of 8-aminooctanoic acid (175 mg, 1.1 mmol) in water (2.5 mL) was added dropwise over the course of 3 h. After stirring at 60 °C overnight, EDC.HCl (192, 1.0 mmol) was added and the mixture was left stirring at room temperature for 2 h. Then, HCl (2N, 30 mL) was added and the resultant precipitate was stored overnight at 4 °C, followed by filtration over a glass filter. The residue was washed with HCl (2N, 50 mL, 2×) and diethylether (50 mL, 2×) to yield the crude intermediate 4-nitrophenyl 8-(4-sulfamoylbenzamido)octanoate **15** (100 mg) as a white solid. $^{1}$H NMR (400 MHz, DMSO-$d_6$) δ 8.62 (t, J = 5.7 Hz, 1H), 8.30 (d, J = 9.0 Hz, 2H), 7.98 (d, J = 8.4 Hz, 2H), 7.88 (d, J = 8.3 Hz, 2H), 7.47 (s, 2H), 7.43 (d, J = 9.0 Hz, 2H), 3.27 (q, J = 6.7 Hz, 2H), 2.64 (t, J = 7.4 Hz, 2H), 1.69 – 1.62 (m, 2H), 1.56 – 1.49 (m, 2H), 1.40 – 1.27 (m, 6H). $^{13}$C NMR (101 MHz, DMSO-$d_6$) δ 171.4, 165.3, 155.5, 146.2, 145.1, 137.7, 127.9, 125.7, 125.4, 123.3, 39.4, 33.5, 29.0, 28.5, 28.4, 26.4, 24.2.

Subsequently, 4-nitrophenyl 8-(4-sulfamoylbenzamido)octanoate **15** (100 mg, 0.22 mmol) was converted to the hydrazide according to the general procedure to yield *N*-(8-hydrazineyl-8-oxooctyl)-4-sulfamoylbenzamide **L8** (48 mg, 0.13 mmol, 13% over two steps) as a white solid. $^{1}$H NMR (400 MHz, DMSO-$d_6$) δ 8.89 (s, 1H), 8.61 (t, J = 5.6 Hz, 1H), 7.97 (d, J = 8.4 Hz, 2H), 7.88 (d, J = 8.4 Hz, 2H), 7.46 (s, 2H), 4.13 (s, 2H), 3.25 (q, J = 6.7 Hz, 2H), 1.99 (m, 2H), 1.50 (m, J = 14.3, 7.0 Hz, 4H), 1.29–1.23 (m, 6H). $^{13}$C NMR (101 MHz, DMSO-$d_6$) δ 171.6, 165.0, 146.1, 137.6, 127.8, 125.5, 40.1, 39.9, 39.7, 33.4, 29.0, 28.6, 26.4, 25.3, 25.2. HRMS (ESI-orbitrap) m/z calculated for $[M+H]^+$ 359.1591, found 359.1602.

4-(hydrazinecarbonyl)benzenesulfonamide (**L9**)

4-nitrophenyl 4-sulfamoylbenzoate **10** (320 mg, 1.0 mmol) was converted into the hydrazide according to the general procedure. Yielded 4-(hydrazinecarbonyl)benzenesulfonamide **L9** (89 mg, 0.41 mmol, 41%) as a white solid. $^{1}$H NMR (400 MHz, DMSO-$d_6$) δ 9.95 (s, 1H), 7.96 (d, J = 8.2 Hz, 2H), 7.87 (d, J = 8.5 Hz, 2H),

7.46 (s, 2H), 4.56 (s, 2H). $^{13}C$ NMR (101 MHz, DMSO-$d_6$) δ 164.7, 146.1, 136.2, 127.6, 125.6. HRMS (ESI-orbitrap) m/z calculated for $[M+Na]^+$ 238.0257, found 238.0267.

Synthesis of biotin-glycine-hydrazide **L10**

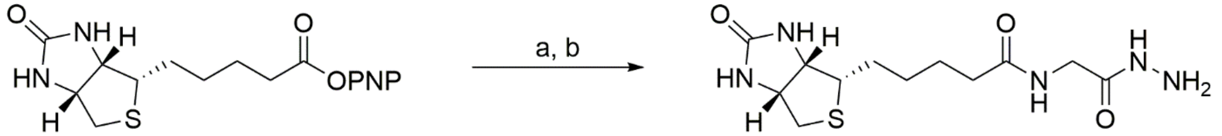


**16** **L10**

a) Glycine methyl ester hydrochloride, DiPEA, DMF; b) hydrazine hydrate, $CH_3OH$ (9 % over two steps).

To a suspension of biotin-OPNP[4] **16** (353 mg, 0.97 mmol) and glycine methylester hydrochloride (140 mg, 1.1 mmol) in DMF (10 mL) was added DiPEA (0.55 mL, 3.2 mmol) and the reaction mixture was stirred at room temperature overnight. The mixture was concentrated under reduced pressure and EtOAc (25 mL) was added. The resultant precipitate was filtered over a glass filter, the residue was washed with EtOAc (50 mL) and ether (50 mL), and dried under vacuum to obtain the crude (265 mg) as white solid.

Without further purification, some of the crude (53 mg, 0.18 mmol) was dissolved in methanol (2 mL), hydrazine hydrate (0.33 mL, 6.8 mmol) was added and the reaction mixture was stirred at room temperature for 2 h. The suspension was filtered over a glass filter, the residue was washed with methanol (10 mL) and dried under vacuum to yield biotin-glycine-hydrazide **L10** (25 mg, 0.083 mmol, 9% over two steps) as a white solid. $^1H$ NMR (400 MHz, DMSO-$d_6$) δ 9.02 (s, 1H), 8.02 (t, J = 5.6 Hz, 1H), 6.45 (s, 1H), 6.37 (s, 1H), 4.34 – 4.09 (m, 4H), 3.62 (d, J = 5.7 Hz, 2H), 3.09 (m, 1H), 2.82 (dd, J = 12.4, 4.8 Hz, 1H), 2.57 (d, J = 12.3 Hz, 1H), 2.12 (t, J = 7.0 Hz, 2H), 1.67 – 1.23 (m, 6H). $^{13}C$ NMR (101 MHz, DMSO-$d_6$) δ 172.5, 168.5, 162.8, 61.0, 59.2, 55.5, 40.7, 39.9, 34.9, 28.2, 28.1, 25.1. HRMS (ESI-orbitrap) m/z calculated for $[M+H]^+$ 316.1438, found 316.1439.

Synthesis of biotin-β-alanine-hydrazide **L11**

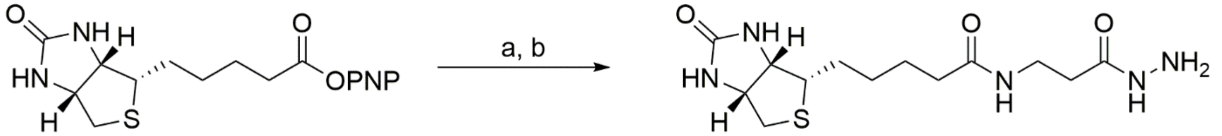


**16** **L11**

a) β-alanine methyl ester hydrochloride, DiPEA, DMF; b) hydrazine hydrate, $CH_3OH$, 50 °C (12% over two steps).

A solution of biotin-OPNP **16** (214 mg, 0.59 mmol), β-alanine methylester hydrochloride (81 mg, 0.58 mmol) and DiPEA (0.31 mL, 1.78 mmol) in DMF (5.6 mL) was stirred at room temperature overnight. Aqueous $KHSO_4$ (1 M, 20 mL) was added and the resultant precipitate was filtered over a glass filter, washed with water (20 mL), EtOAc (20 mL) and ether (20 mL), and dried under reduced pressure to obtain the crude intermediate biotin-βAla-OMe (39 mg).

Without further purification, methanol (5 mL) was added to the crude and the mixture was heated to 50 °C. Subsequently, hydrazine hydrate (0.13 mL, 2.7 mmol) was added and the mixture was stirred at 50 °C for 2 h. After concentrating the mixture under reduced pressure, the crude was triturated with methanol (10 mL) and dried under vacuum to yield biotin-β-alanine-hydrazide **L11** (23 mg, 0.070 mmol, 12% over

two steps) as a white solid. $^{1}H$ NMR (400 MHz, DMSO-$d_6$) δ 8.98 (s, 1H), 7.79 (s, 1H), 6.41 (s, 1H), 6.35 (s, 1H), 4.30 (t, J = 6.2 Hz, 1H), 4.14 (bs, 3H), 3.21 (q, J = 7.0 Hz, 2H), 3.10 (m, 1H), 2.82 (dd, J = 12.2, 4.8 Hz, 1H), 2.57 (d, J = 12.4 Hz, 1H), 2.17 (t, J = 7.0 Hz, 2H), 2.03 (t, J = 7.0 Hz, 2H), 1.68 – 1.20 (m, 6H). $^{13}C$ NMR (101 MHz, DMSO-$d_6$) δ 172.0, 169.8, 162.7, 61.0, 59.2, 55.4, 39.8, 35.2, 35.1, 33.7, 28.2, 28.0, 25.2. HRMS (ESI-orbitrap) m/z calculated for $[M+H]^+$ 330.1594, found 330.1597.

*<u>Synthesis of **FITZ am-zide**</u>*

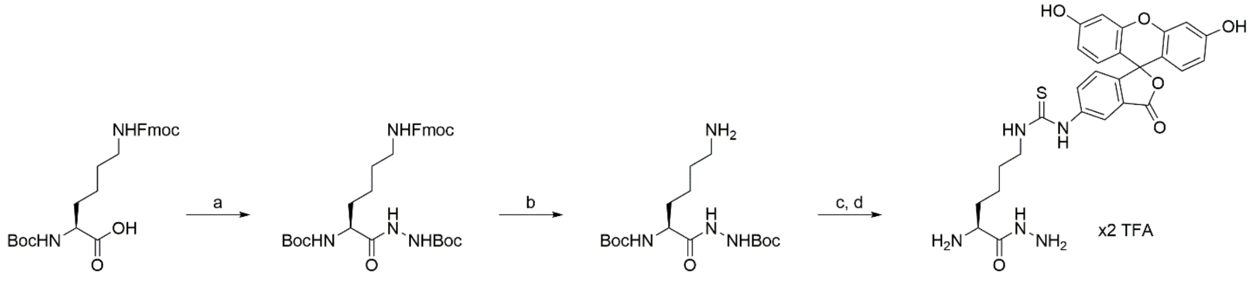


17 18 19 FITC am-zide

a) tert-butyl carbazate, HCTU, DiPEA, DMF (94%); b) diethylamine, methanol (quantitative); c) FITC, DiPEA, THF; d) triethylsilane/water/TFA (43% over two steps).

Boc-Lys(Fmoc)-NHNHBoc (**18**)

DiPEA (1.7 mL, 10 mmol) was added to a solution of Boc-Lys(Fmoc)-OH **17** (1.76 g, 3.7 mmol), tert-butyl carbazate (0.55 g, 4.1 mmol) and HCTU (1.69 g, 4.1 mmol) in DMF (40 mL) and the mixture was stirred at room temperature for 3 h. The reaction mixture was concentrated under reduced pressure and then diluted with EtOAc (150 mL), washed with aqueous $KHSO_4$ (1M, 100 mL, 2×), $NaHCO_3$ (saturated, 100 mL, 3×) and aqueous LiCl (5% w/v, 100 mL, 1×), dried over $Na_2SO_4$, filtered over cotton and concentrated under reduced pressure. Purification by column chromatography (2% methanol/dichloromethane) yielded Boc-Lys(Fmoc)-NHNHBoc **18** (2.09 g, 3.56 mmol, 96%) as a white solid foam. $^{1}H$ NMR (400 MHz, Chloroform-*d*) δ 8.38 (bs, 1H), 7.75 (d, J = 7.5 Hz, 2H), 7.59 (d, J = 7.5 Hz, 2H), 7.39 (t, J = 7.4 Hz, 2H), 7.30 (t, J = 7.4 Hz, 2H), 6.69 (bs, 1H), 5.22 (m, 2H), 4.37 (m, 2H), 4.20 (t, J = 7.1 Hz, 2H), 3.19 (m, 2H), 1.89 – 1.79 (m, 1H), 1.73 – 1.66 (m, 1H), 1.58 – 1.42 (m, 22H). $^{13}C$ NMR (101 MHz, Chloroform-*d*) δ 172.1, 156.8, 156.1, 155.4, 127.8, 127.2, 125.2, 120.1, 81.8, 80.5, 66.7, 52.8, 47.4, 40.5, 32.0, 29.4, 28.5, 28.3, 22.4. HRMS (ESI-orbitrap) m/z calculated for $[M+H]^+$ 583.3126, found 583.3115.

Boc-Lys($NH_2$)-NHNHBoc (**19**)

Diethylamine (15 mL, 145 mmol) was added to a solution of Boc-Lys(Fmoc)-NHNHBoc **18** (2.09 g, 3.56 mmol) in methanol (37 mL) and the mixture was stirred at room temperature overnight. The reaction mixture was diluted with EtOAc (100 mL) and extracted with aqueous $KHSO_4$ (1 M, 150 mL, 2×). The combined water layers were filtered over cotton to remove a white precipitate and basified with aqueous NaOH (2 M) to pH 12, followed by extraction with EtOAc (150 mL, 2×). The combined organic layers were dried over $Na_2SO_4$, filtered over cotton and concentrated under reduced pressure. Purification by column chromatography (1/10/89 -> 1/15/84 $NH_4OH$ (25% in water)/methanol/dichloromethane) yielded Boc-

Lys(NH$_2$)-NHNHBoc **19** (1.02 g, 2.83 mmol, 79%) as a white solid foam. $^1$H NMR (400 MHz, Methanol-*d$_4$*) δ 4.06 (m, 1H), 2.65 (t, J = 6.6 Hz, 2H), 1.84 – 1.71 (m, 1H), 1.68 – 1.59 (m, 1H), 1.51 – 1.44 (m, 22H). $^{13}$C NMR (101 MHz, Methanol-*d$_4$*) δ 174.7, 157.7, 157.5, 81.7, 80.6, 54.5, 42.1, 33.3, 33.1, 28.7, 28.6, 23.9. HRMS (ESI-orbitrap) m/z calculated for $[M+H]^+$ 361.2446, found 361.2447.

**FITC am-zide**

Boc-Lys(NH$_2$)-NHNHBoc **19** (374 mg, 1.0 mmol) was added to a suspension of fluorescein 5-isothiocyanate (FITC; 319 mg, 0.82 mmol) and DiPEA (0.6 mL, 3.4 mmol) in THF (38 mL) and the reaction mixture was stirred at room temperature overnight. The mixture was concentrated under reduced pressure and diluted with EtOAc (80 mL) and aqueous $KHSO_4$ (1M, 80 mL). The layers were separated and the organic layer was dried over $Na_2SO_4$, filtered over cotton and concentrated under reduced pressure to yield crude **Boc-protected FITC am-zide** (421 mg) as an orange solid. $^1$H NMR (400 MHz, Methanol-*d$_4$*) δ 8.14 (s, 1H), 7.76 (d, J = 8.3 Hz, 1H), 7.13 (d, J = 8.1 Hz, 1H), 6.67 (m, 4H), 6.54 (d, J = 8.6 Hz, 2H), 4.11 (s, 1H), 3.61 (s, 2H), 1.84 – 1.41 (m, 26H). $^{13}$C NMR (101 MHz, Methanol-*d$_4$*) δ 182.6, 174.7, 171.1, 161.3, 157.7, 157.5, 154.1, 149.5, 142.4, 131.9, 130.3, 128.8, 126.1, 125.6, 119.9, 113.6, 111.4, 103.5, 81.8, 80.7, 33.2, 29.5 28.7, 28.6, 24.1. HRMS (ESI-orbitrap) m/z calculated for $[M+H]^+$ 750.2803, found 750.2812.

Without further purification, **Boc-protected FITC am-zide** (367 mg, 0.57 mmol) was dissolved in triethylsilane/water/TFA (2.5/2.5/95, 10 mL) and the reaction mixture was stirred at room temperature overnight. Diethyl ether (75 mL) was added and the resultant orange precipitate was filtered over a glass filter, washed with diethyl ether (75 mL) and dried *in vacuo* to yield **FITC am-zide** (272 mg, 0.35 mmol, 43% over two steps) as an orange solid. 1H NMR (400 MHz, Methanol-d4) δ 8.20 (s, 1H), 7.76 (d, J = 8.2 Hz, 1H), 7.17 (d, J = 8.0 Hz, 1H), 6.75 (m, 4H), 6.60 (d, J = 8.6 Hz, 2H), 3.87 (s, 1H), 3.66 (s, 2H), 1.99 – 1.91 (m, 2H), 1.80 – 1.66 (m, 2H), 1.61 – 1.45 (m, 2H). $^{13}$C NMR (151 MHz, Methanol-*d$_4$*) δ 182.9, 174.4, 170.9, 169.5, 162.4, 154.8, 147.9, 142.6, 131.6, 130.6, 129.2, 126.2, 120.6, 114.3, 112.0, 103.5, 53.3, 45.0, 32.2, 29.5, 23.2. $^{19}$F NMR (565 MHz, Methanol-*d$_4$*) δ -76.48, -76.95. HRMS (ESI-orbitrap) m/z calculated for $[M+H]^+$ 550.1755, found 550.1747.

References

[1] J. Lohse, A. Schindl, N. Danda, C. P. Williams, K. Kramer, B. Kuster, M. D. Witte, G. Médard, *Chem. Commun.* **2017**, *53*, 11929–11932.

[2] R. P. A. Berntsson, N. A. Oktaviani, F. Fusetti, A. M. W. H. Thunnissen, B. Poolman, D. J. Slotboom, *Protein Sci.* **2009**, *18*, 1121–1127.

[3] R. P. A. Berntsson, J. Ter Beek, M. Majsnerowska, R. H. Duurkens, P. Puri, B. Poolman, D. J. Slotboom, *Proc. Natl. Acad. Sci. U. S. A.* **2012**, *109*, 13990–13995.

[4] J. Lohse, L. J. Y. M. Swier, R. C. Oudshoorn, G. Médard, B. Kuster, D. J. Slotboom, M. D. Witte, *Bioconjug. Chem.* **2017**, *28*, 913–917.

[5] O. P. Kuipers, P. G. G. A. De Ruyter, M. Kleerebezem, W. M. De Vos, *Trends Biotechnol.* **1997**, *15*, 135–140.

[6] H. Schägger, *Nat. Protoc.* **2006**, *1*, 16–22.

NMR spectra

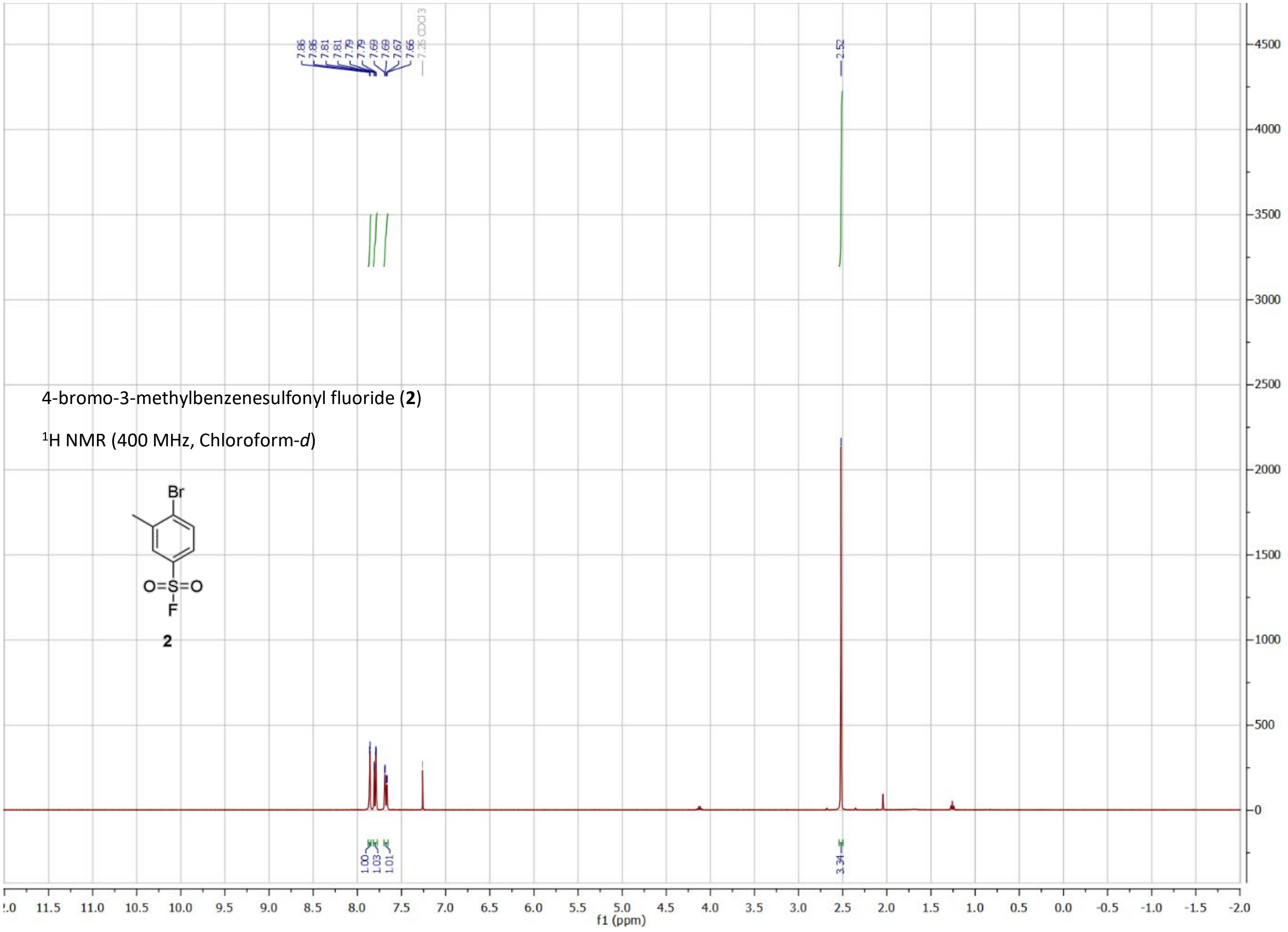

7.86
7.86
7.81
7.81
7.79
7.79
7.69
7.69
7.67
7.66
7.25 CDCl3
2.52
4-bromo-3-methylbenzenesulfonyl fluoride (**2**)
$^{1}$H NMR (400 MHz, Chloroform-*d*)
Br
O=S=O
F
2
1.00
1.03
1.01
3.34
f1 (ppm)

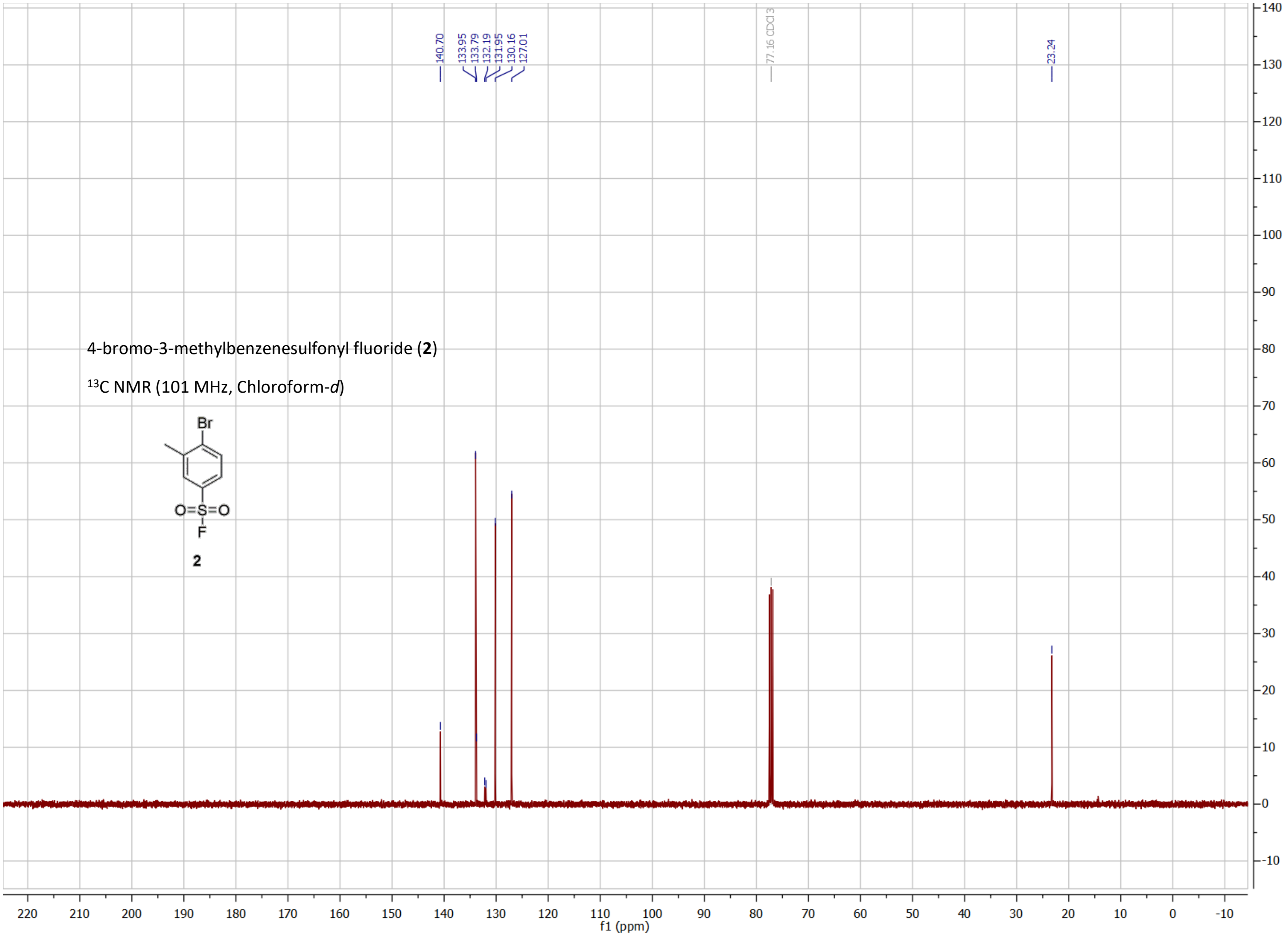

140.70
133.95
133.79
132.19
131.95
130.16
127.01
77.16 CDCl3
23.24
4-bromo-3-methylbenzenesulfonyl fluoride (**2**)
$^{13}$C NMR (101 MHz, Chloroform-*d*)
Br
O=S=O
F
2
f1 (ppm)

4-bromo-3-methylbenzenesulfonyl fluoride (**2**)

$^{19}$F NMR (376 MHz, Chloroform-*d*)

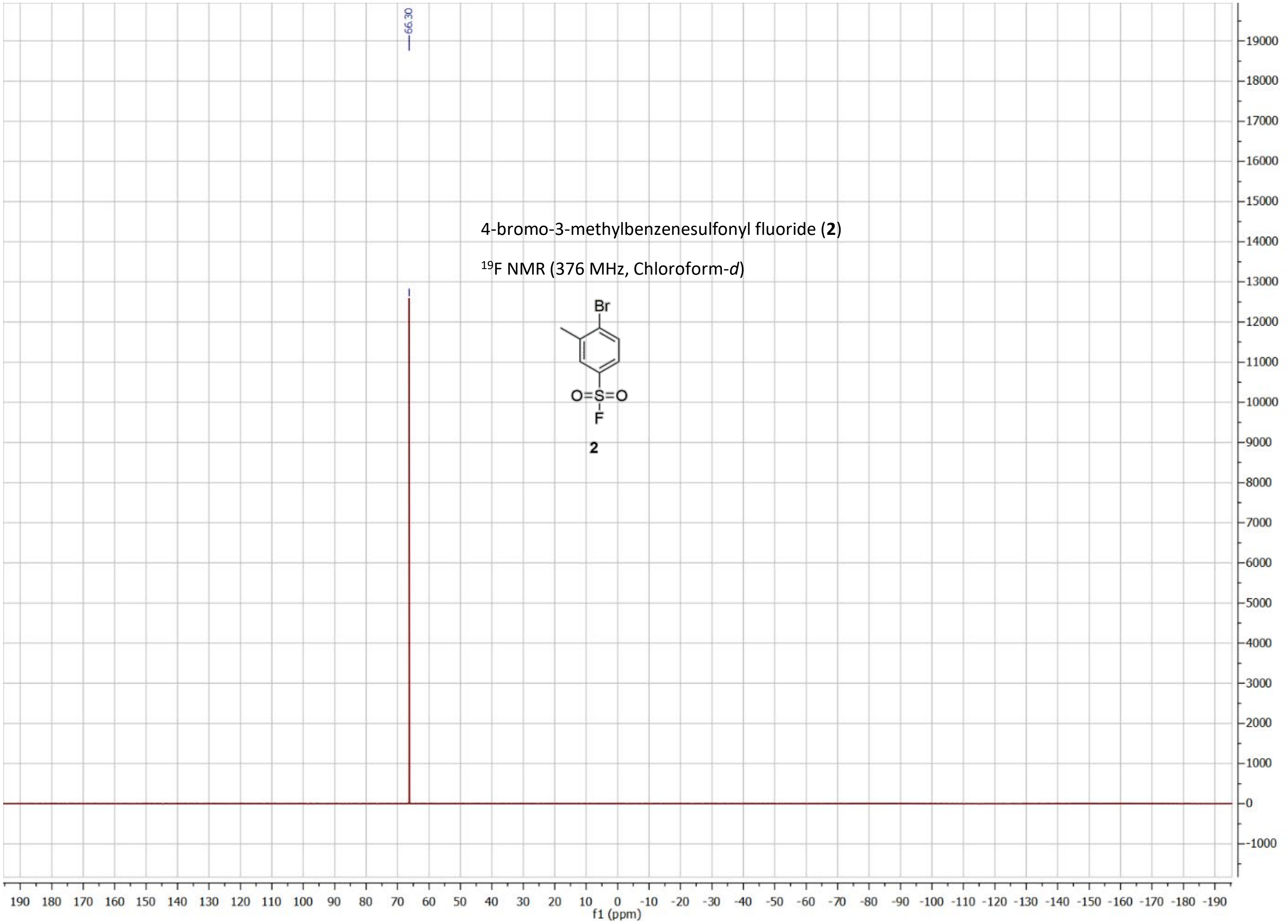

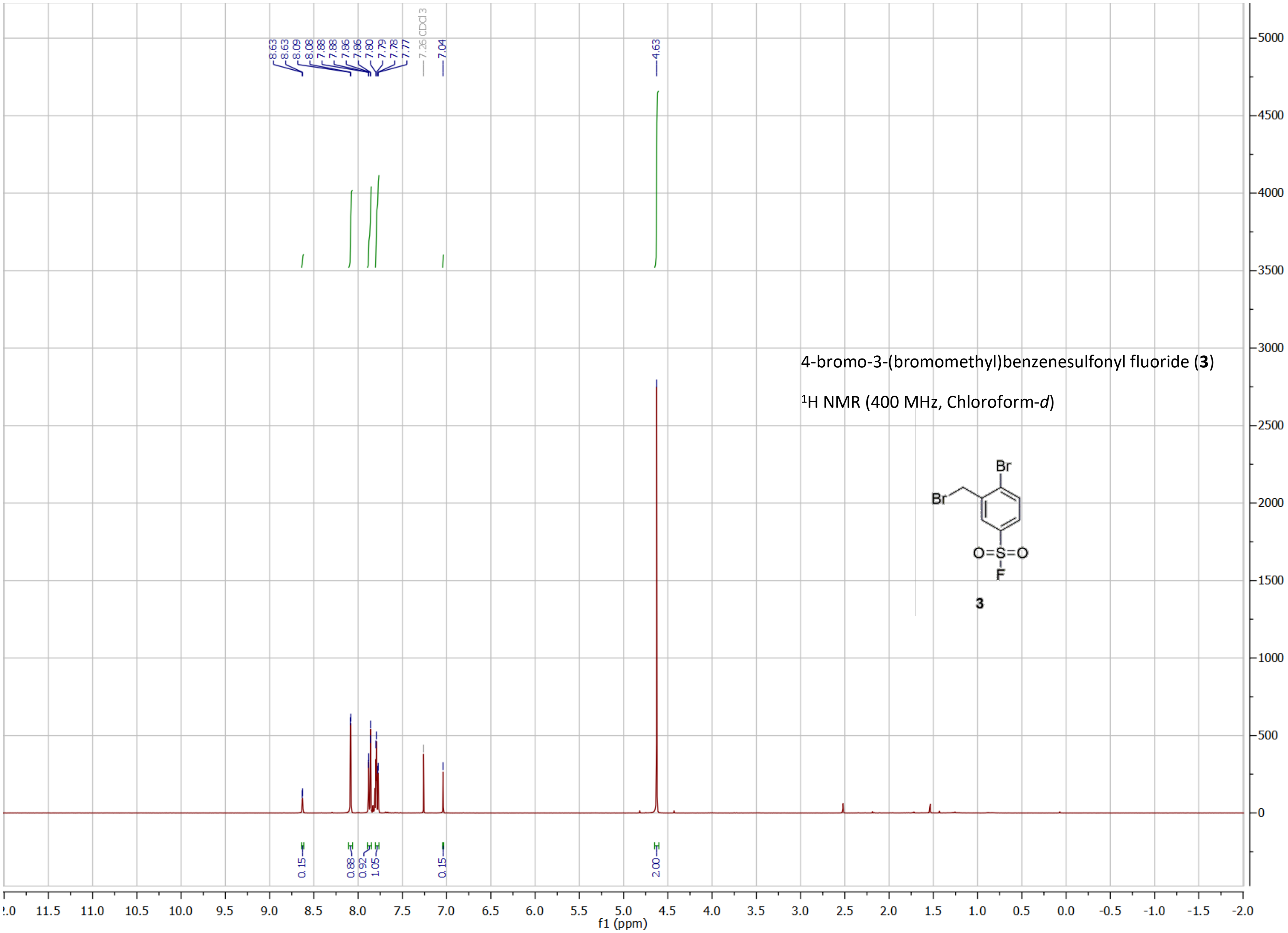

8.63
8.63
8.09
8.08
7.88
7.88
7.86
7.86
7.80
7.79
7.78
7.77
7.26 CDCl3
7.04
4.63
4-bromo-3-(bromomethyl)benzenesulfonyl fluoride (3)
1H NMR (400 MHz, Chloroform-d)
Br
Br
O=S=O
F
3
0.15
0.88
0.92
1.05
0.15
2.00
f1 (ppm)

4-bromo-3-(bromomethyl)benzenesulfonyl fluoride (**3**)

$^{13}$C NMR (101 MHz, Chloroform-*d*)

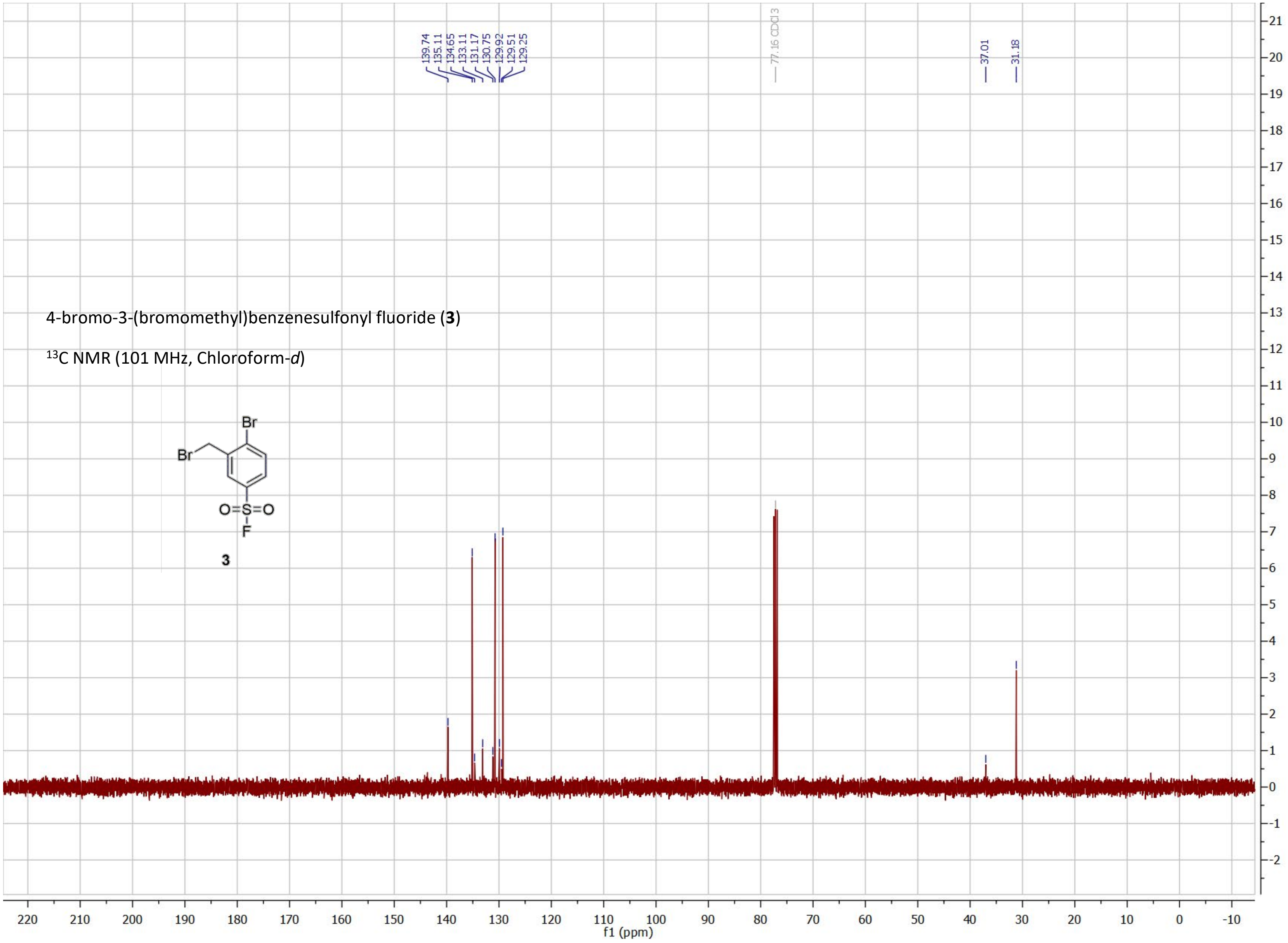

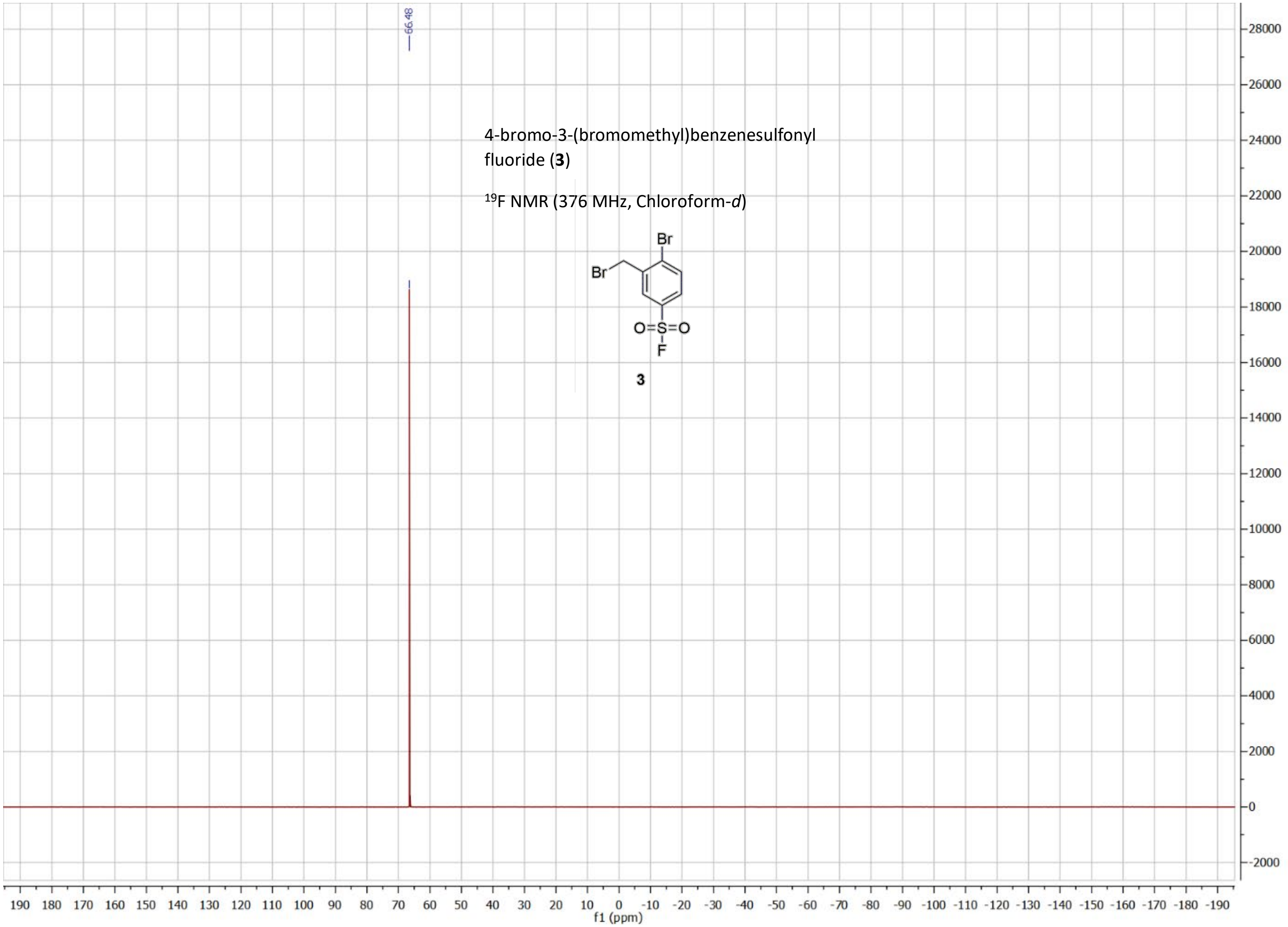
-66.48
4-bromo-3-(bromomethyl)benzenesulfonyl fluoride (**3**)
$^{19}F$ NMR (376 MHz, Chloroform-*d*)
Br
Br
O=S=O
F
3
f1 (ppm)

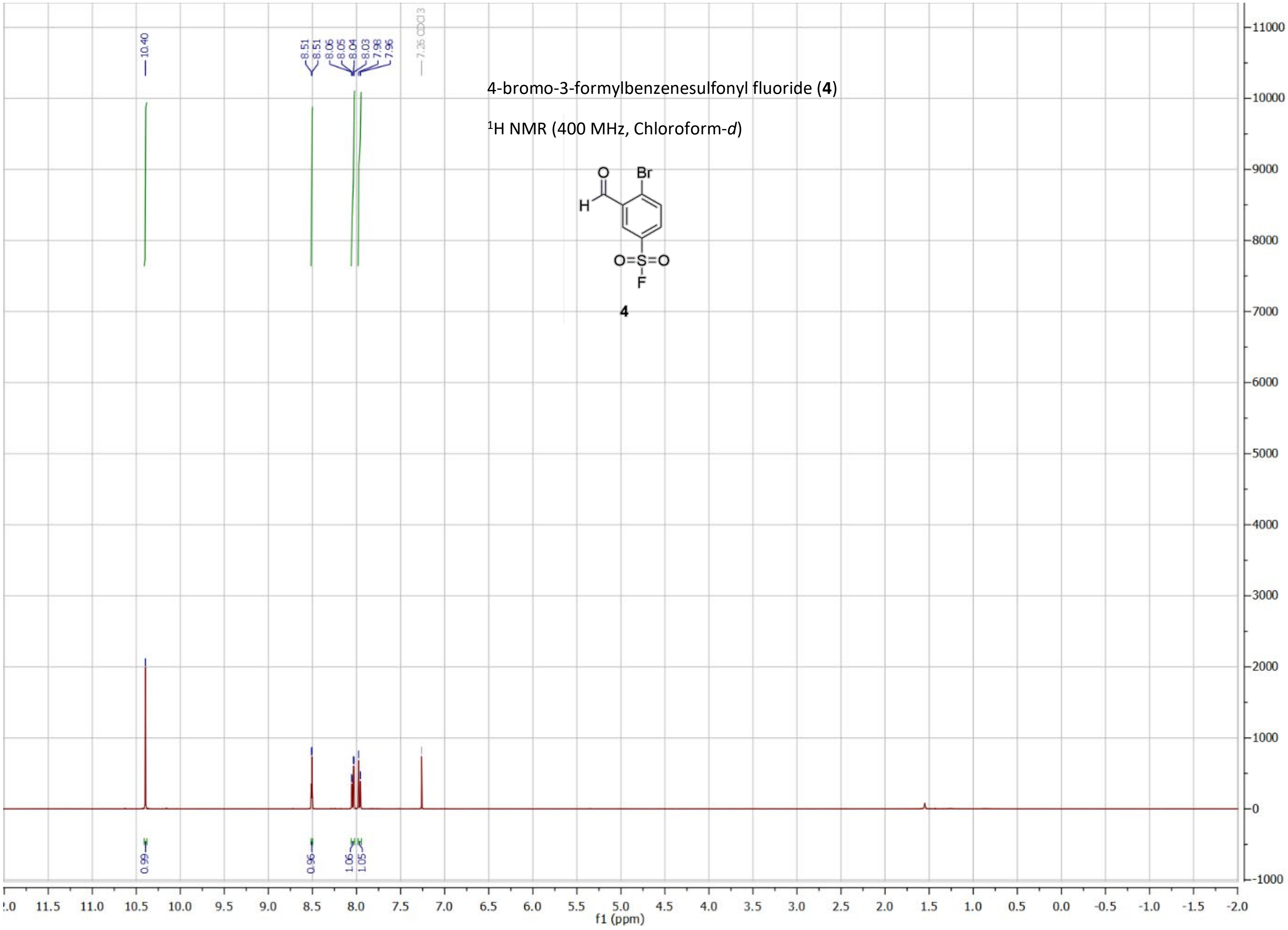
4-bromo-3-formylbenzenesulfonyl fluoride (4)
1H NMR (400 MHz, Chloroform-d)
Br
O
H
O=S=O
F
4
10.40
8.51
8.51
8.06
8.05
8.04
8.03
7.98
7.96
7.26 CDCl3
0.99
0.96
1.06
1.05
f1 (ppm)

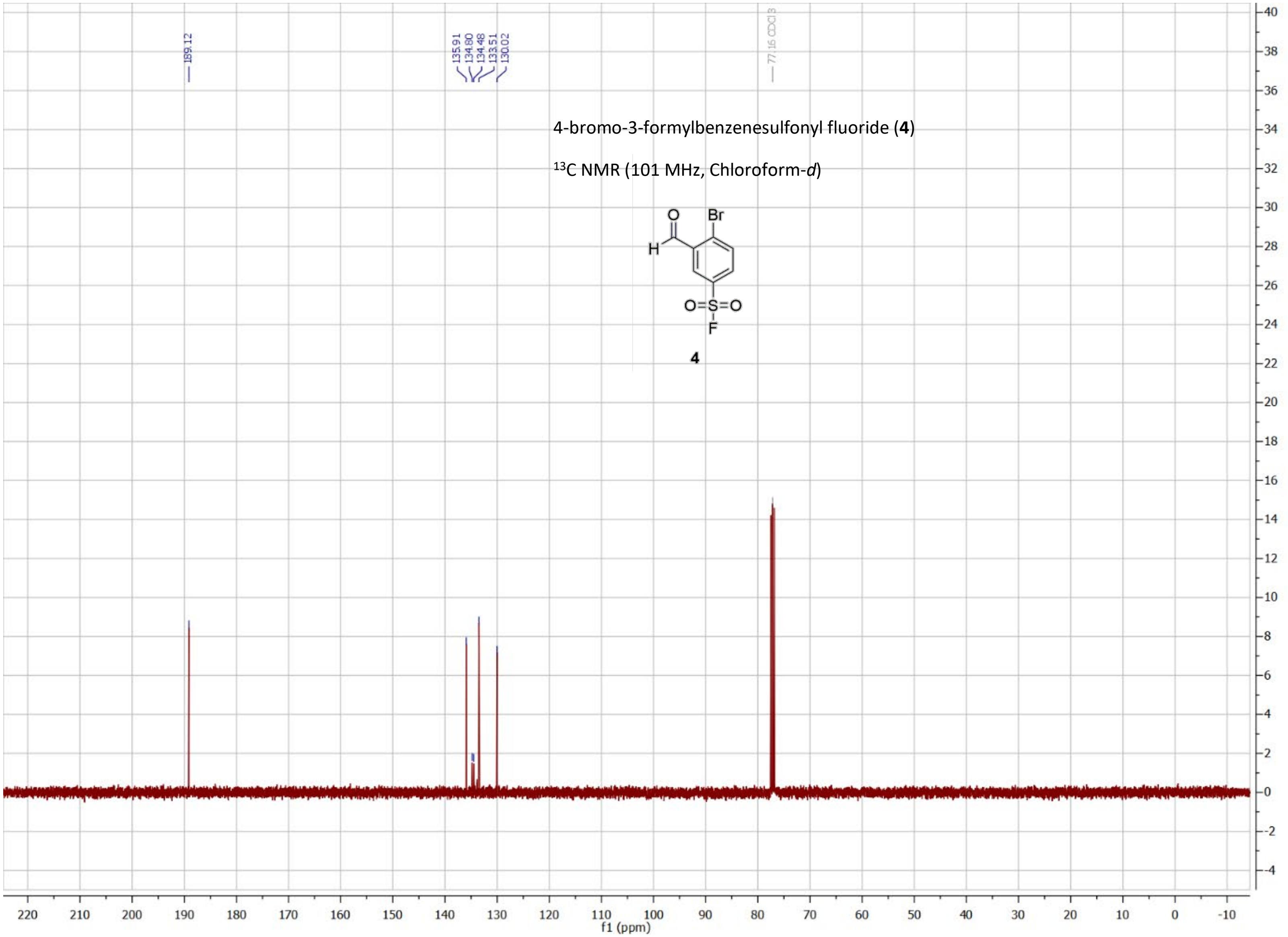
189.12
135.91
134.80
134.48
133.51
130.02
77.16 CDCl3
4-bromo-3-formylbenzenesulfonyl fluoride (4)
13C NMR (101 MHz, Chloroform-d)
O
Br
H
O=S=O
F
4
f1 (ppm)

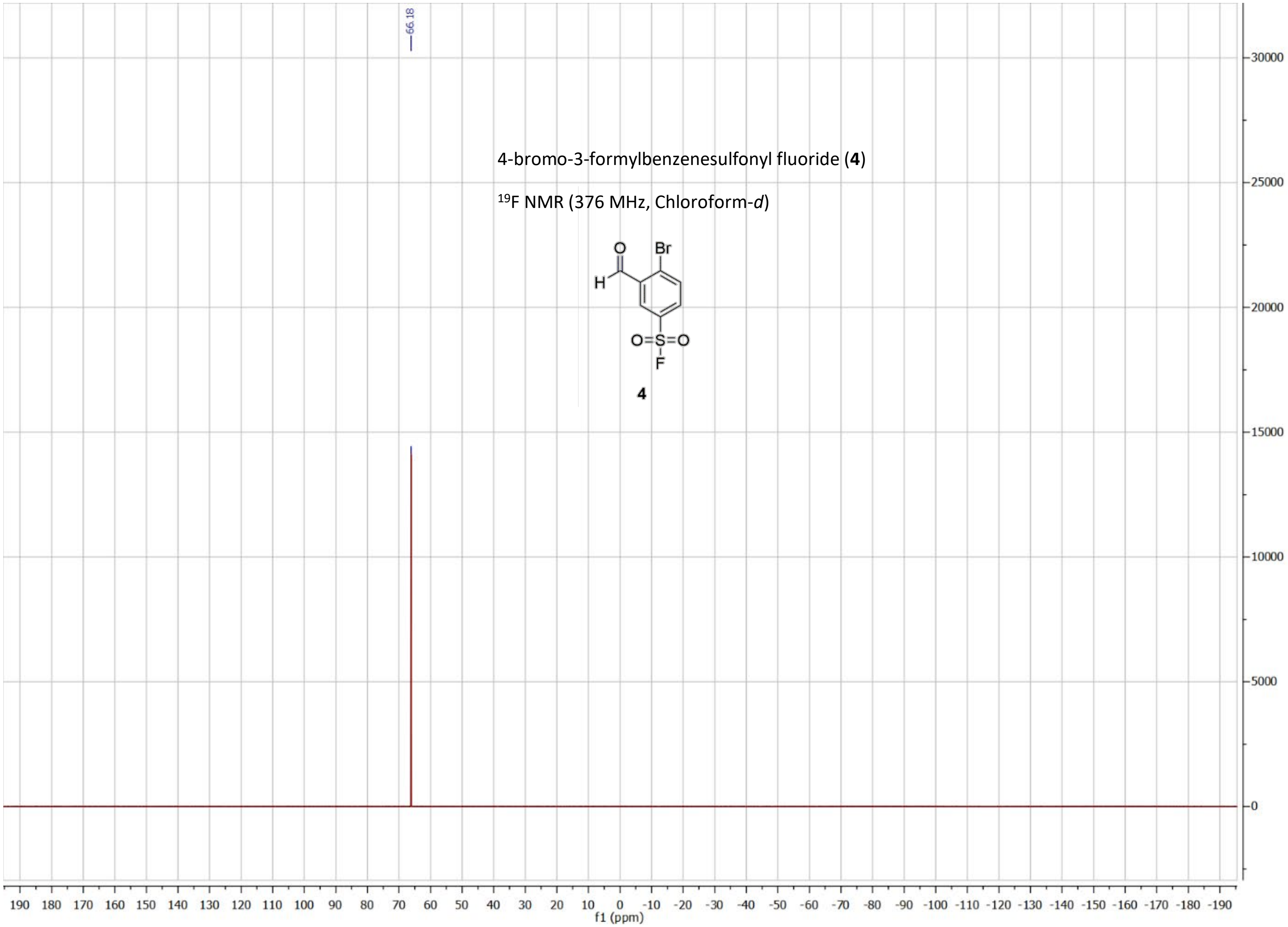
66.18
4-bromo-3-formylbenzenesulfonyl fluoride (4)
19F NMR (376 MHz, Chloroform-d)
Br
O
H
O=S=O
F
4
30000
25000
20000
15000
10000
5000
0
190 180 170 160 150 140 130 120 110 100 90 80 70 60 50 40 30 20 10 0 -10 -20 -30 -40 -50 -60 -70 -80 -90 -100 -110 -120 -130 -140 -150 -160 -170 -180 -190
f1 (ppm)

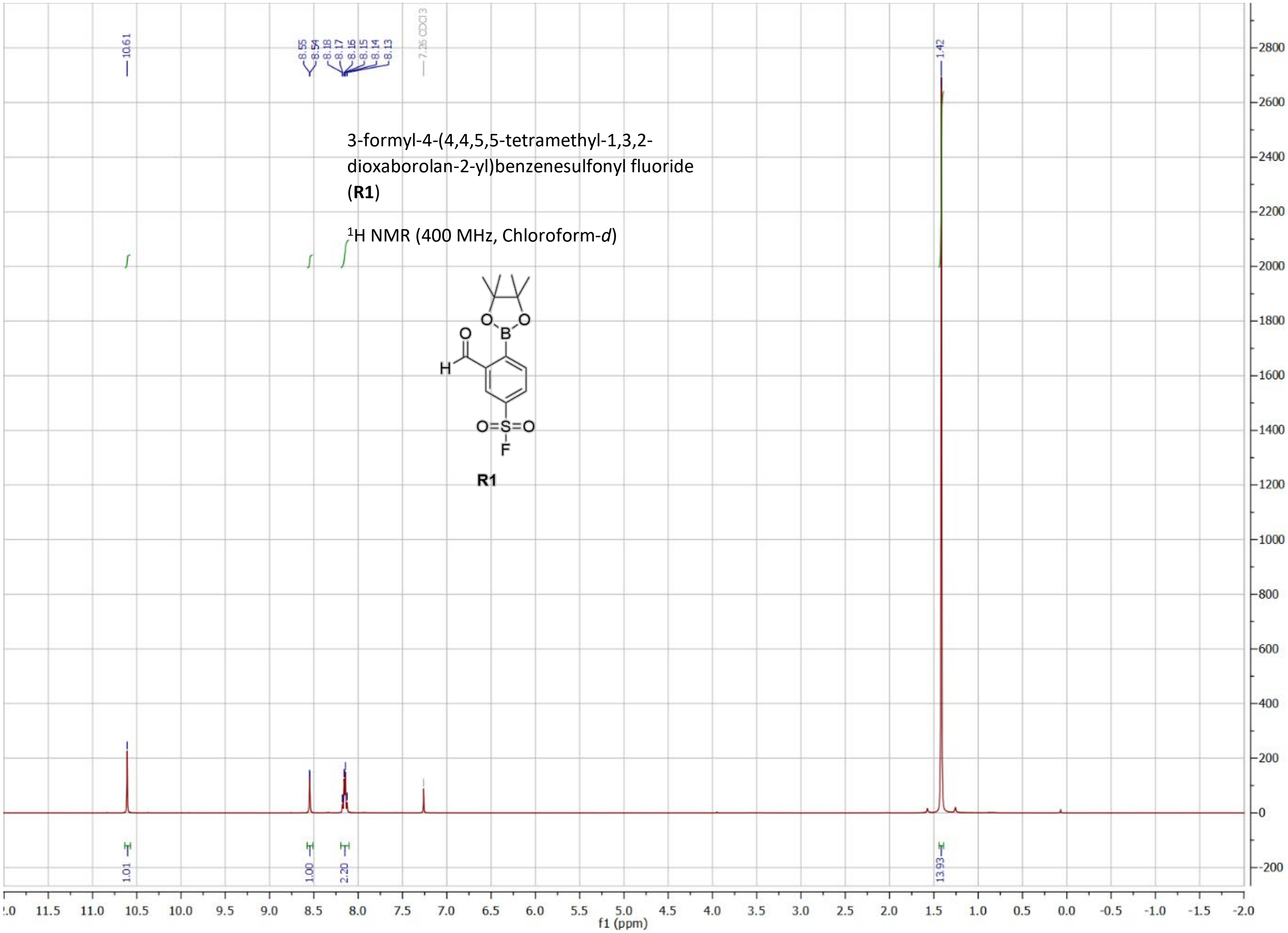
3-formyl-4-(4,4,5,5-tetramethyl-1,3,2-dioxaborolan-2-yl)benzenesulfonyl fluoride (**R1**)
$^{1}$H NMR (400 MHz, Chloroform-*d*)
R1
10.61
8.55
8.54
8.18
8.17
8.16
8.15
8.14
8.13
7.25 CDCl3
1.42
1.01
1.00
2.20
13.93
f1 (ppm)

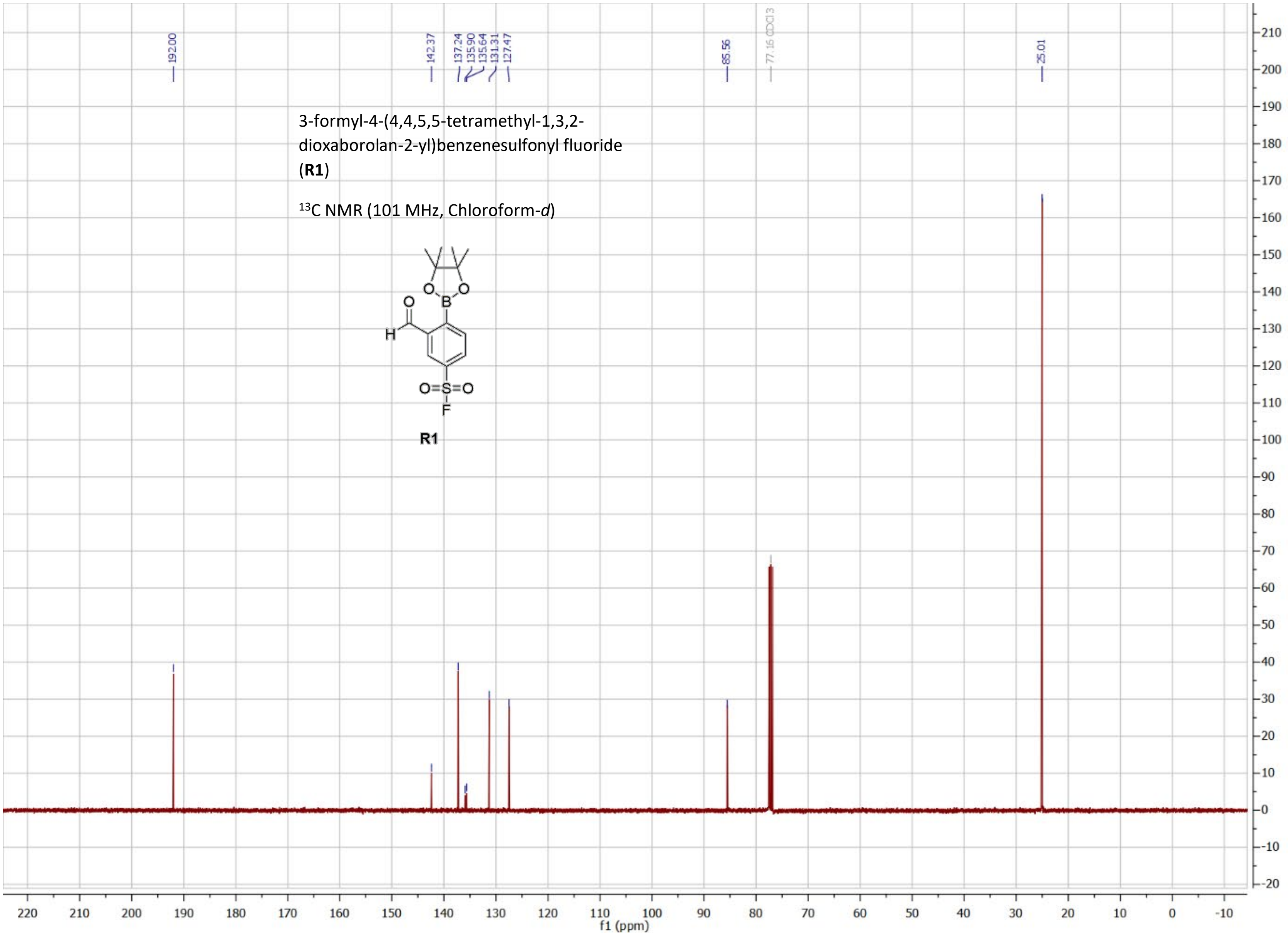
192.00
142.37
137.24
135.90
135.64
131.31
127.47
85.56
77.16 CDCl3
25.01
3-formyl-4-(4,4,5,5-tetramethyl-1,3,2-dioxaborolan-2-yl)benzenesulfonyl fluoride (**R1**)
$^{13}$C NMR (101 MHz, Chloroform-*d*)
R1
f1 (ppm)

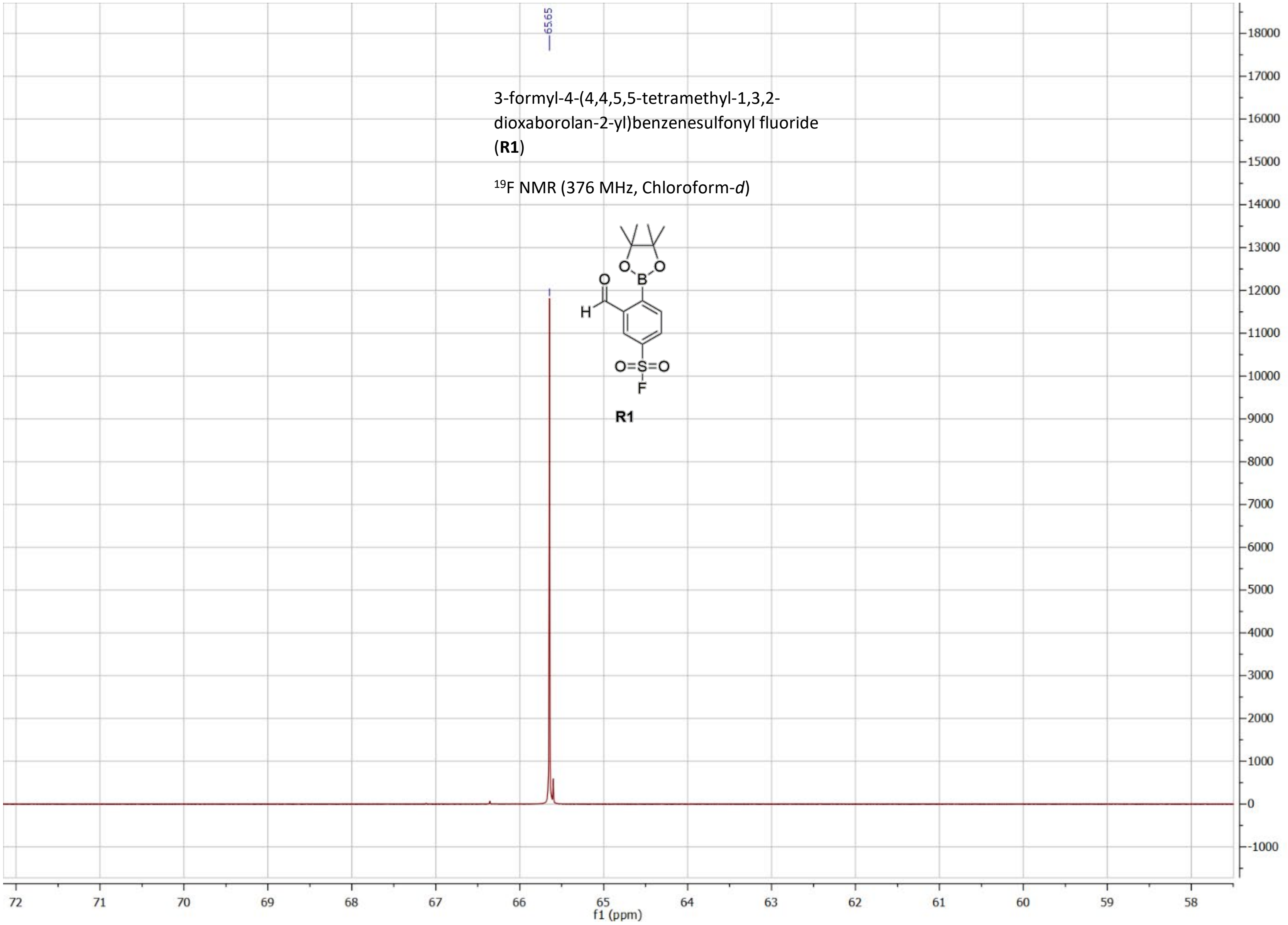

-65.65
3-formyl-4-(4,4,5,5-tetramethyl-1,3,2-dioxaborolan-2-yl)benzenesulfonyl fluoride (R1)
19F NMR (376 MHz, Chloroform-d)
R1
72 71 70 69 68 67 66 65 64 63 62 61 60 59 58
f1 (ppm)
18000 17000 16000 15000 14000 13000 12000 11000 10000 9000 8000 7000 6000 5000 4000 3000 2000 1000 0 -1000

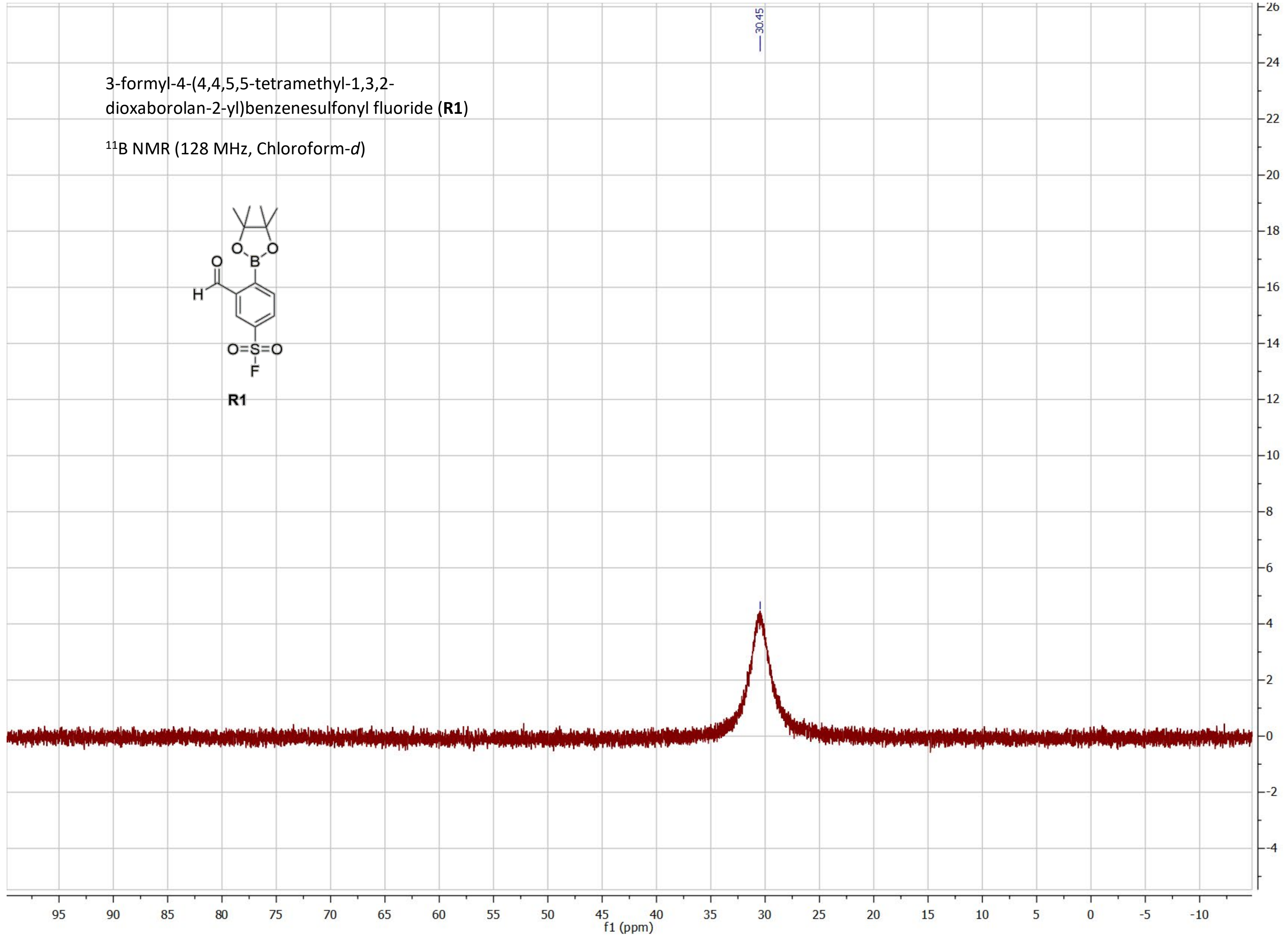
3-formyl-4-(4,4,5,5-tetramethyl-1,3,2-dioxaborolan-2-yl)benzenesulfonyl fluoride (R1)
11B NMR (128 MHz, Chloroform-d)
R1
30.45
f1 (ppm)

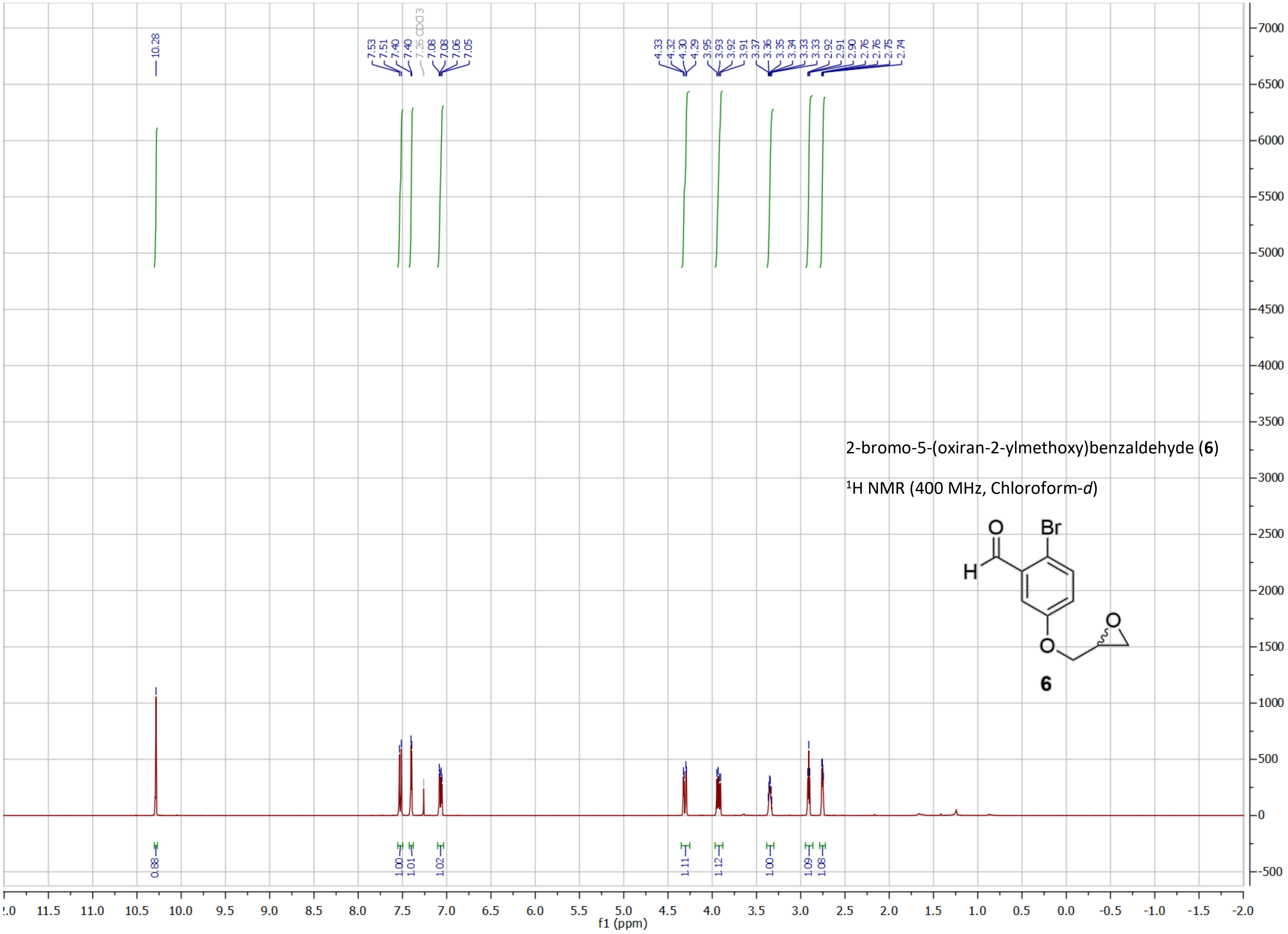
10.28
7.53 7.51 7.40 7.40 7.26 CDCl3 7.08 7.08 7.06 7.05
4.33 4.32 4.30 4.29 3.95 3.93 3.92 3.91 3.37 3.36 3.35 3.34 3.33 3.33 2.92 2.91 2.90 2.76 2.76 2.75 2.74
2-bromo-5-(oxiran-2-ylmethoxy)benzaldehyde (**6**)
$^{1}$H NMR (400 MHz, Chloroform-*d*)
O
Br
H
O
O
6
0.88
1.00
1.01
1.02
1.11
1.12
1.00
1.09
1.08
f1 (ppm)

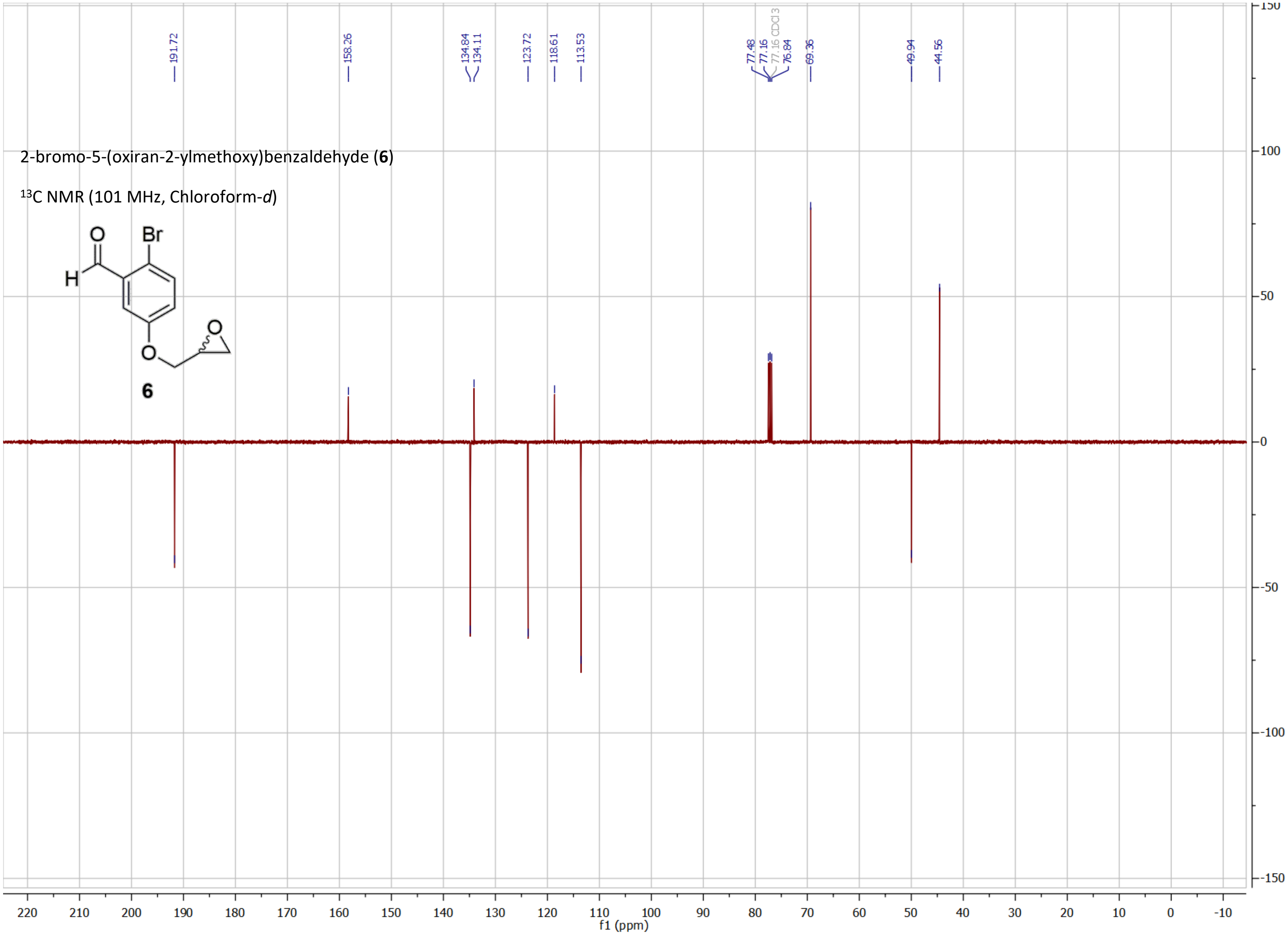
2-bromo-5-(oxiran-2-ylmethoxy)benzaldehyde (**6**)
$^{13}$C NMR (101 MHz, Chloroform-*d*)
O
Br
H
O
O
**6**
191.72
158.26
134.84
134.11
123.72
118.61
113.53
77.48
77.16
77.16 CDCl3
76.84
69.36
49.94
44.56
150
100
50
0
-50
-100
-150
220 210 200 190 180 170 160 150 140 130 120 110 100 90 80 70 60 50 40 30 20 10 0 -10
f1 (ppm)

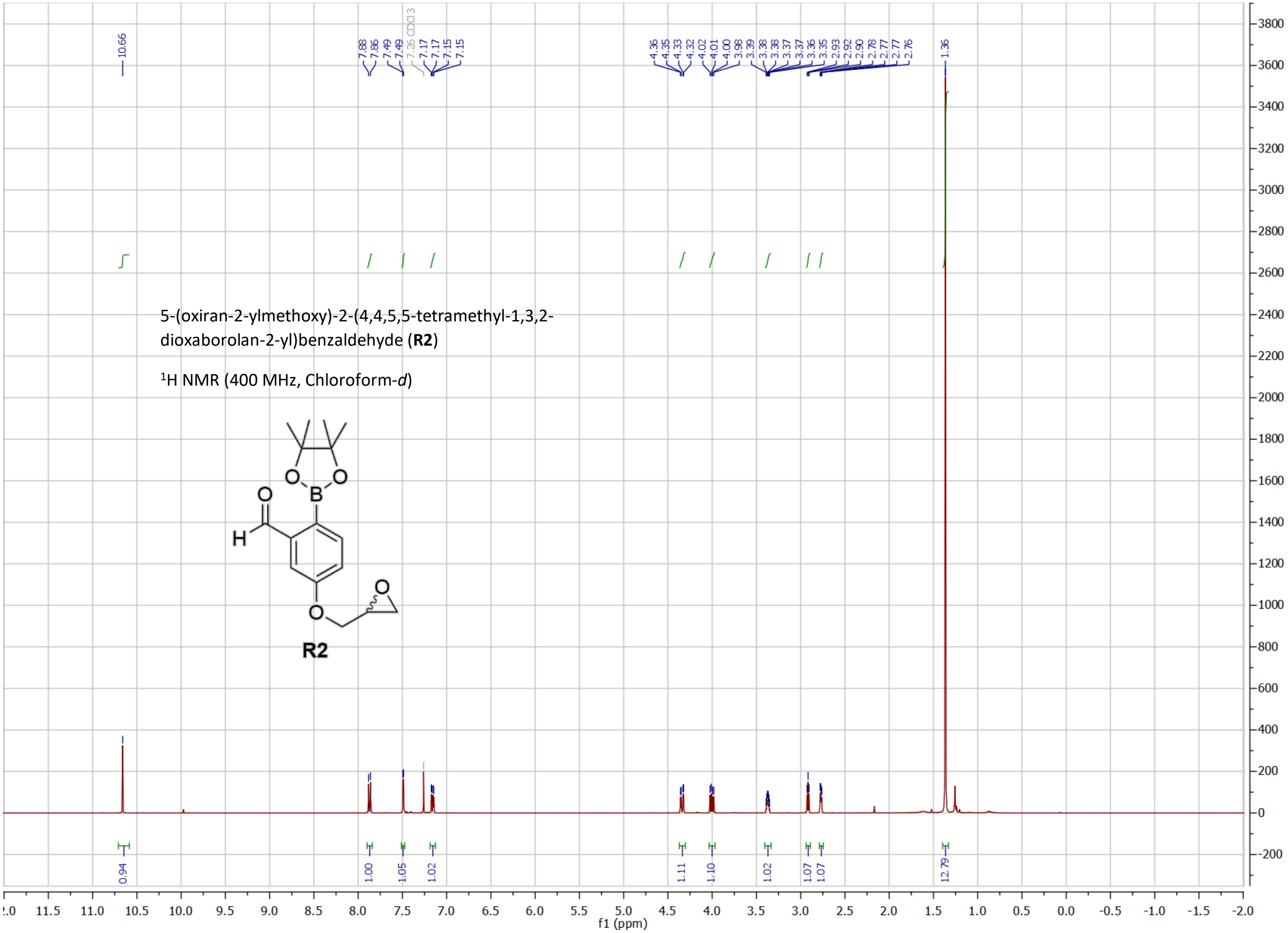
5-(oxiran-2-ylmethoxy)-2-(4,4,5,5-tetramethyl-1,3,2-dioxaborolan-2-yl)benzaldehyde (R2)
1H NMR (400 MHz, Chloroform-d)
R2
10.66
7.88 7.86 7.49 7.49 7.26 CDCl3 7.17 7.17 7.15 7.15
4.36 4.35 4.33 4.32 4.02 4.01 4.00 3.98 3.39 3.38 3.38 3.37 3.36 3.35 2.93 2.92 2.90 2.78 2.77 2.77 2.76
1.36
0.94 1.00 1.05 1.02 1.11 1.10 1.02 1.07 1.07 12.79
f1 (ppm)

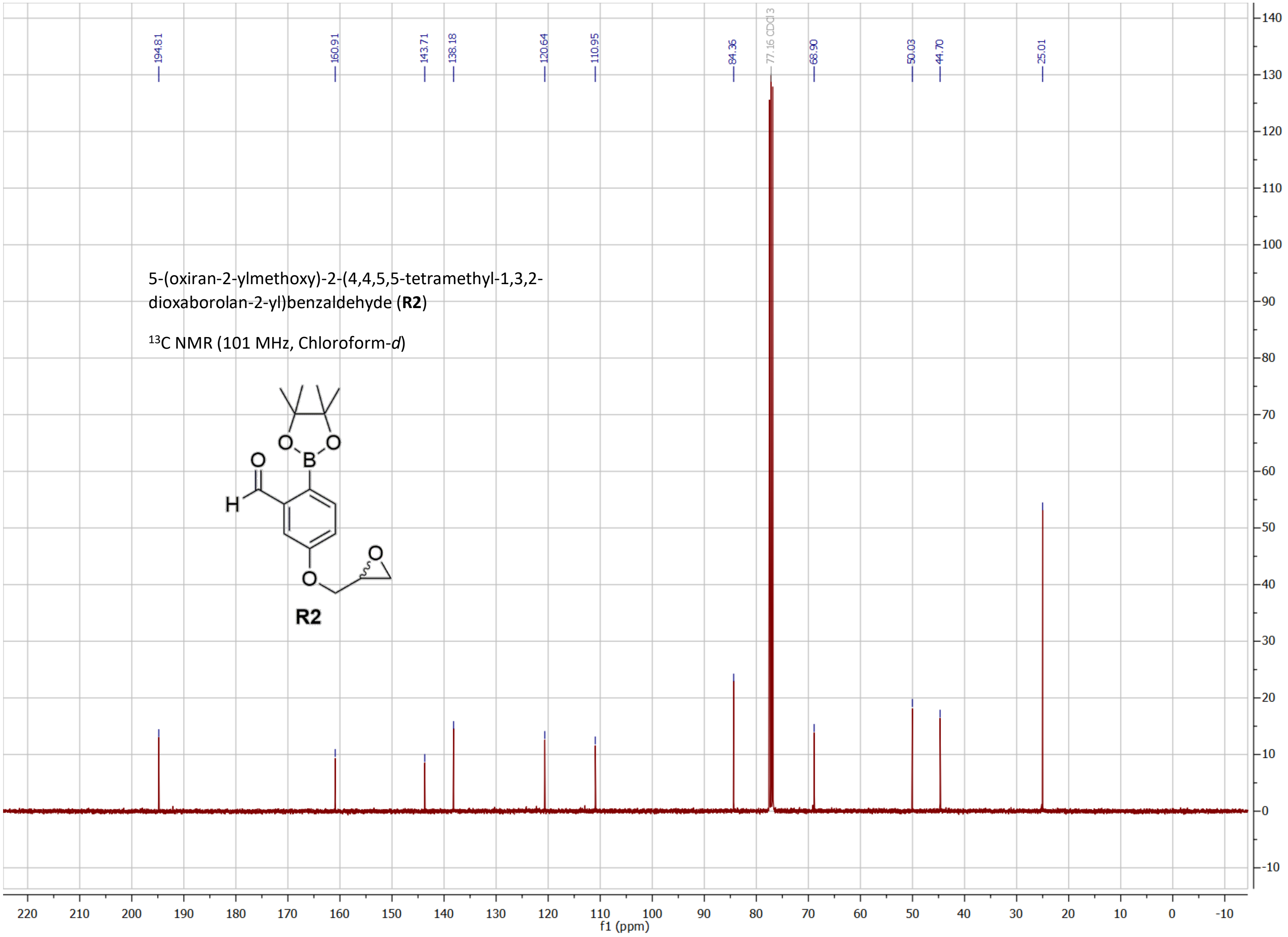

194.81
160.91
143.71
138.18
120.64
110.95
84.36
77.16 CDCl3
68.90
50.03
44.70
25.01
5-(oxiran-2-ylmethoxy)-2-(4,4,5,5-tetramethyl-1,3,2-dioxaborolan-2-yl)benzaldehyde (**R2**)
$^{13}$C NMR (101 MHz, Chloroform-*d*)
O
B
O
O
H
O
O
R2
220 210 200 190 180 170 160 150 140 130 120 110 100 90 80 70 60 50 40 30 20 10 0 -10
f1 (ppm)
140 130 120 110 100 90 80 70 60 50 40 30 20 10 0 -10

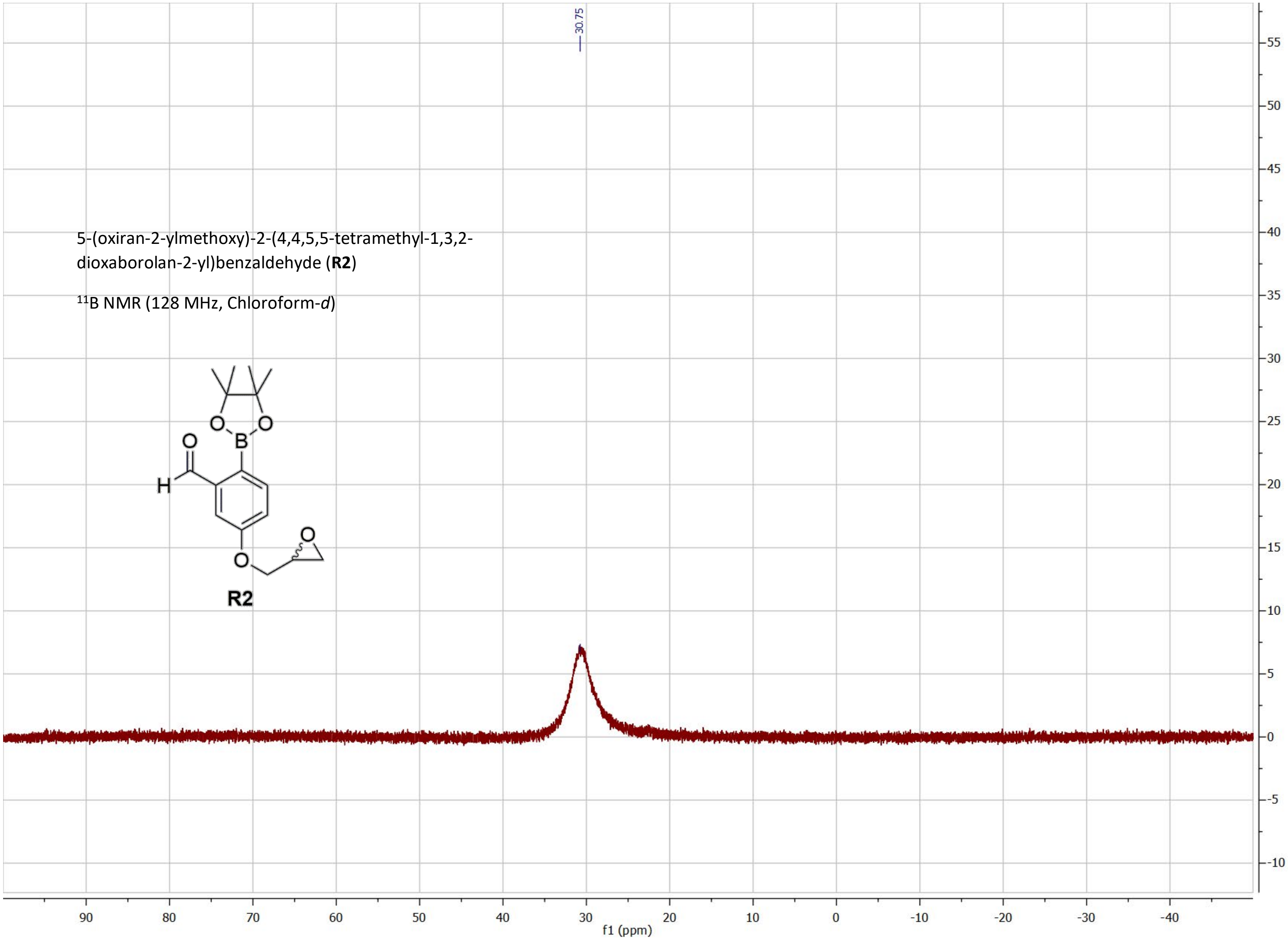
30.75
5-(oxiran-2-ylmethoxy)-2-(4,4,5,5-tetramethyl-1,3,2-dioxaborolan-2-yl)benzaldehyde (**R2**)
$^{11}$B NMR (128 MHz, Chloroform-*d*)
R2
f1 (ppm)

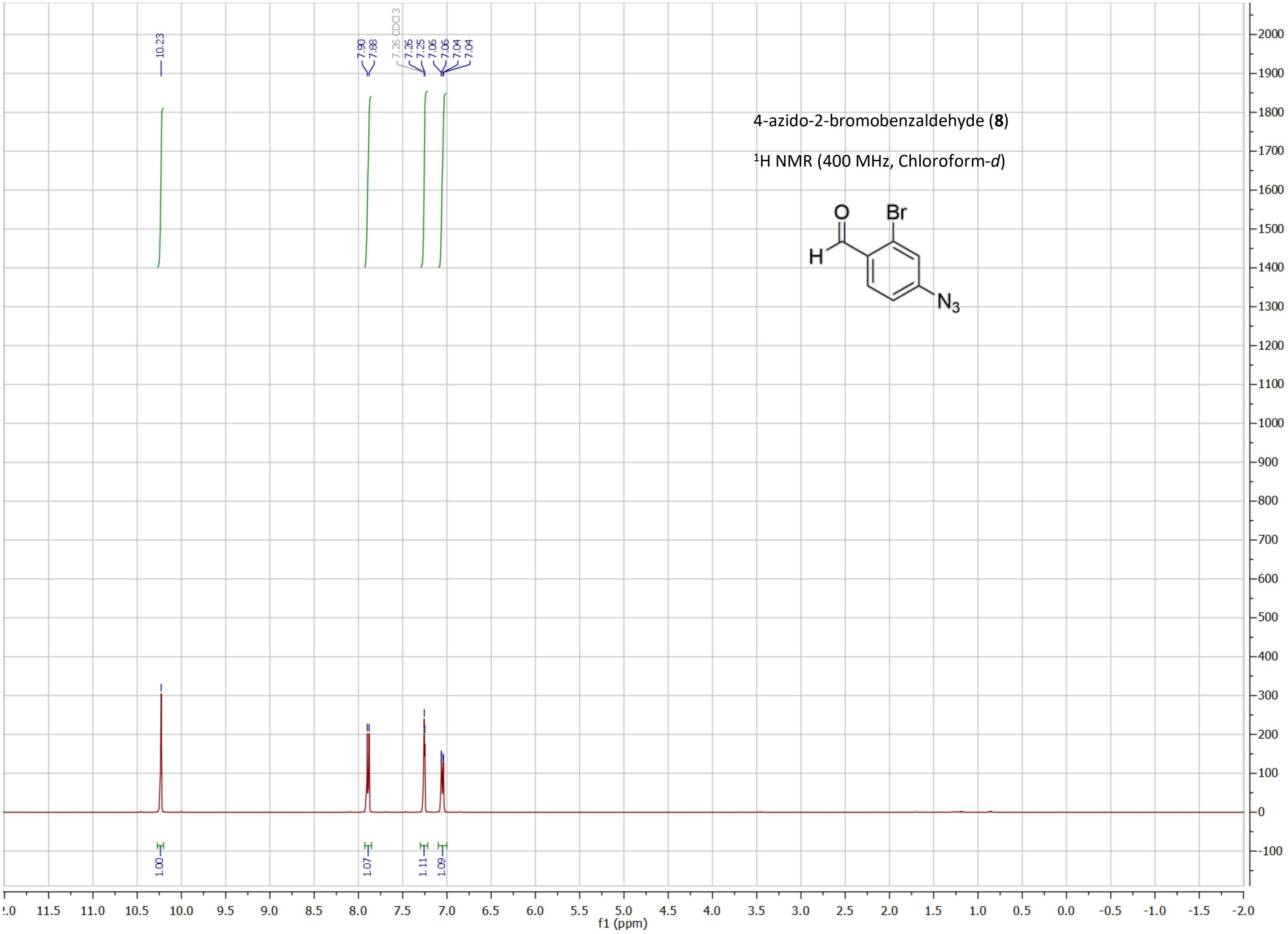

4-azido-2-bromobenzaldehyde (**8**)
1H NMR (400 MHz, Chloroform-*d*)
O
Br
H
N3
10.23
7.90
7.88
7.26 CDCl3
7.25
7.25
7.06
7.06
7.04
7.04
1.00
1.07
1.11
1.09
f1 (ppm)

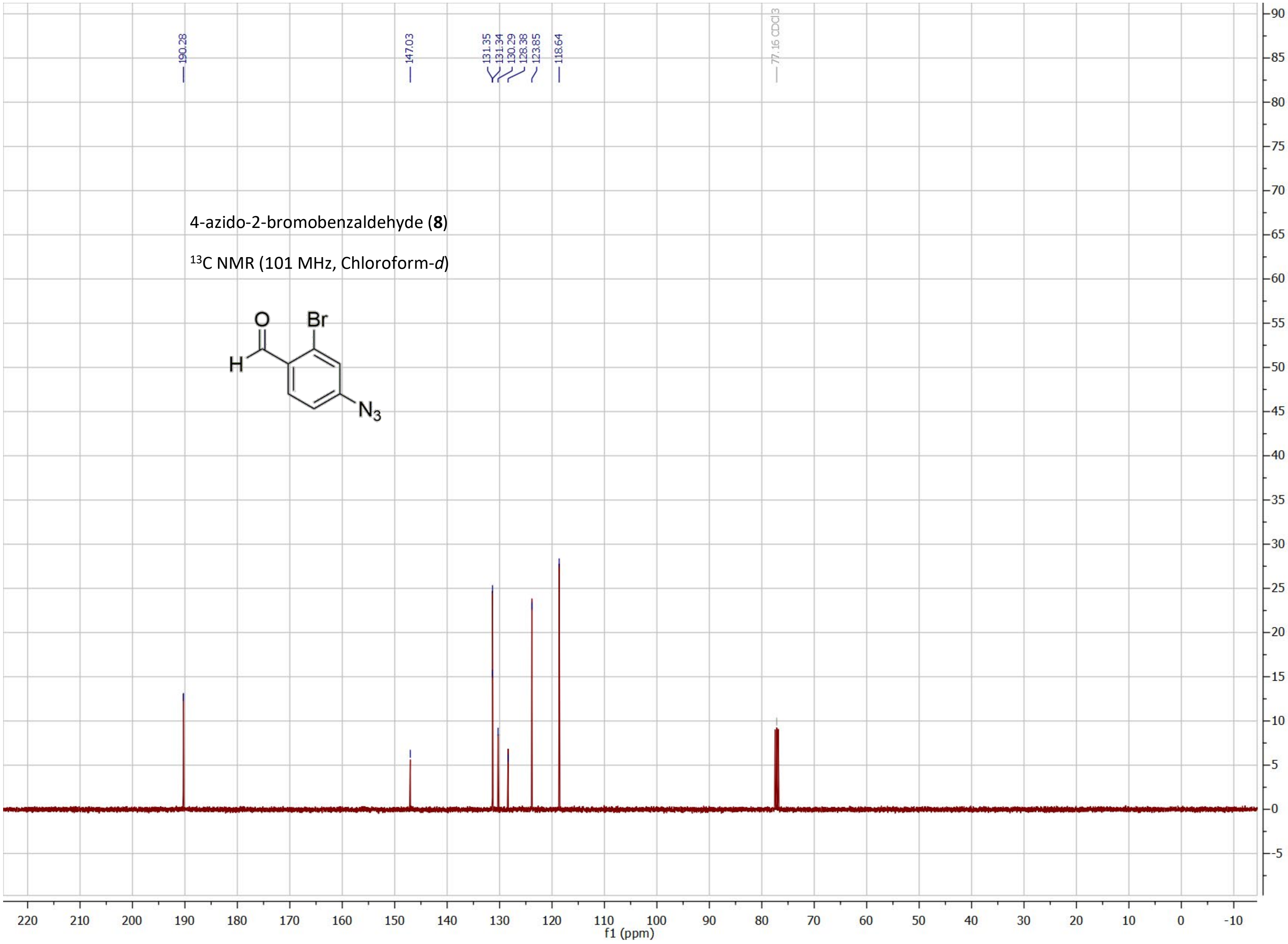
4-azido-2-bromobenzaldehyde (**8**)
$^{13}$C NMR (101 MHz, Chloroform-*d*)
O
Br
H
N3
190.28
147.03
131.35
131.34
130.29
128.38
123.85
118.64
77.16 CDCl3
f1 (ppm)

4-azido-2-bromobenzaldehyde (**8**)

$^{19}$F NMR (376 MHz, Chloroform-*d*)

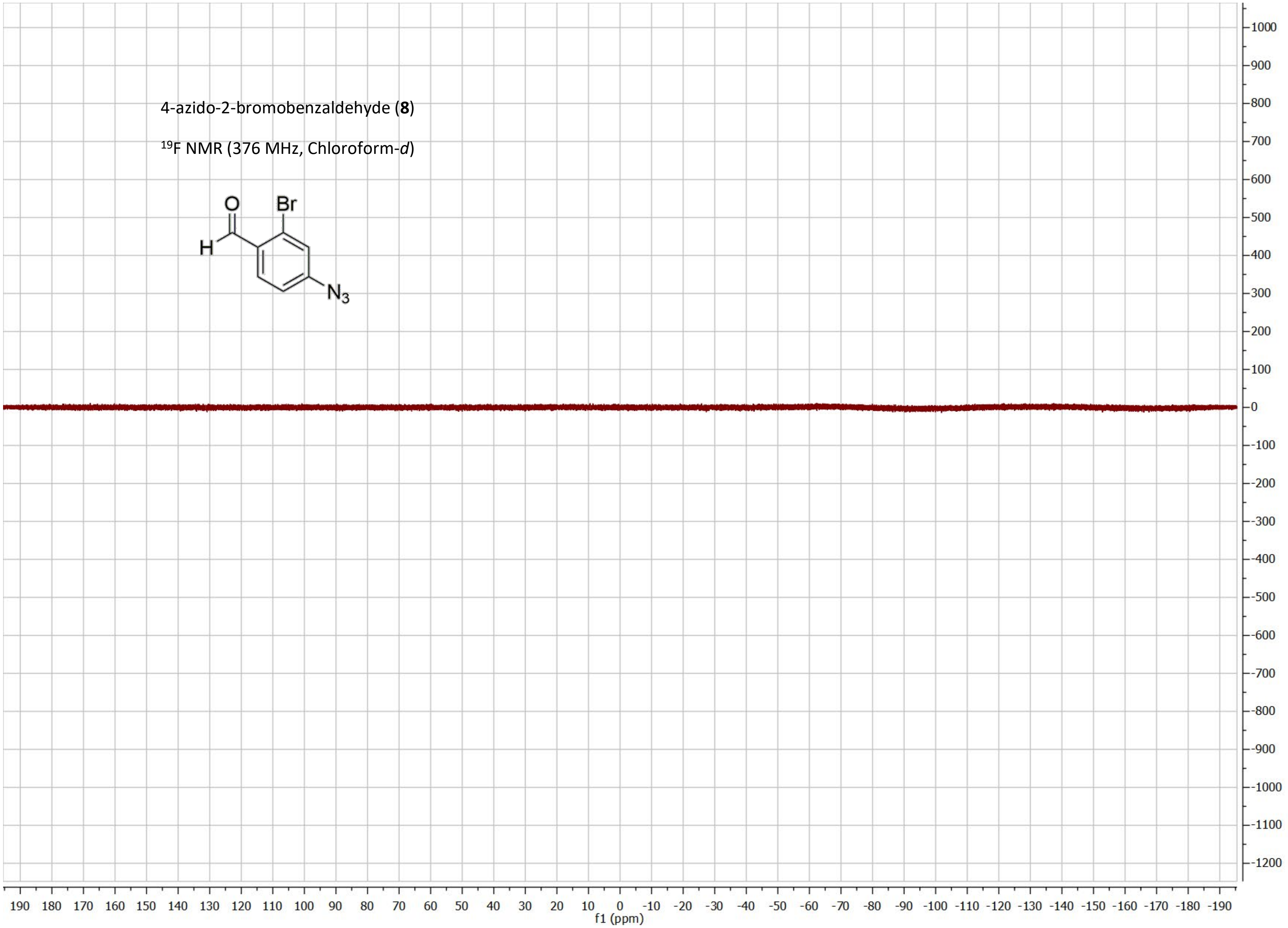

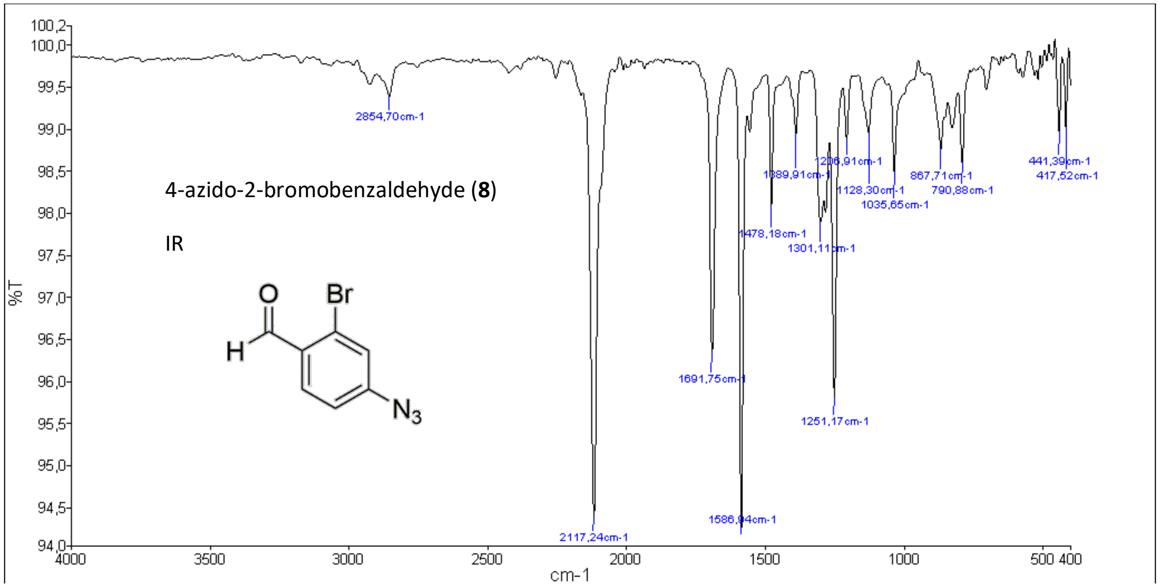

4-azido-2-bromobenzaldehyde (**8**)
IR
%T
cm-1
2854,70cm-1
2117,24cm-1
1691,75cm-1
1586,94cm-1
1478,18cm-1
1389,91cm-1
1301,11cm-1
1251,17cm-1
1128,30cm-1
1035,65cm-1
867,71cm-1
790,88cm-1
441,39cm-1
417,52cm-1


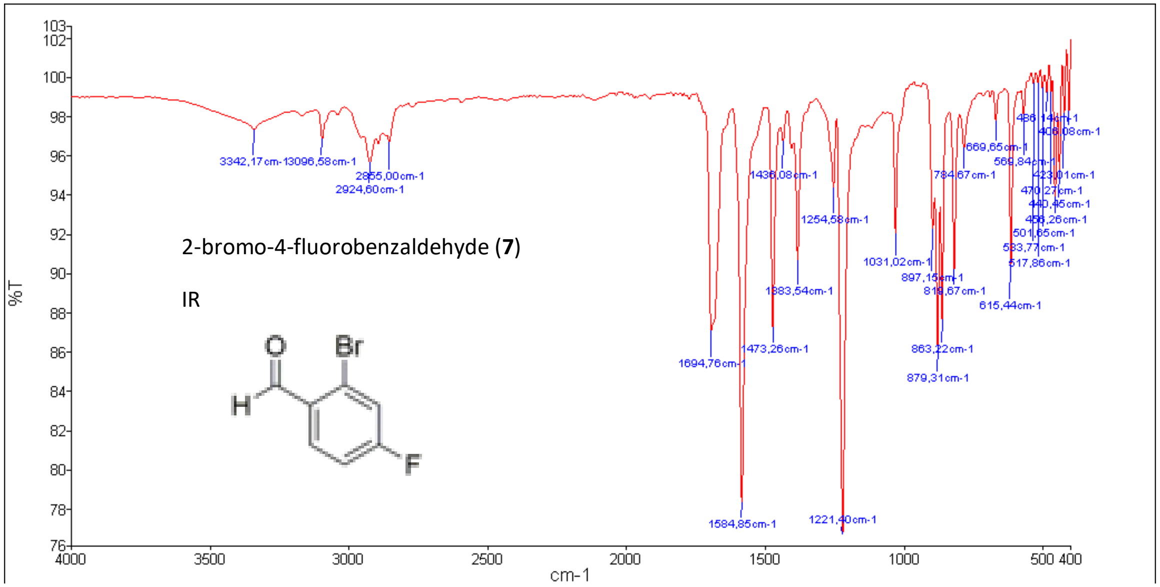

2-bromo-4-fluorobenzaldehyde (**7**)
IR
%T
cm-1
3342,17cm-1
3096,58cm-1
2924,60cm-1
2855,00cm-1
1694,76cm-1
1584,85cm-1
1473,26cm-1
1436,08cm-1
1383,54cm-1
1254,58cm-1
1221,40cm-1
1031,02cm-1
897,15cm-1
879,31cm-1
863,22cm-1
784,67cm-1
669,65cm-1
615,44cm-1

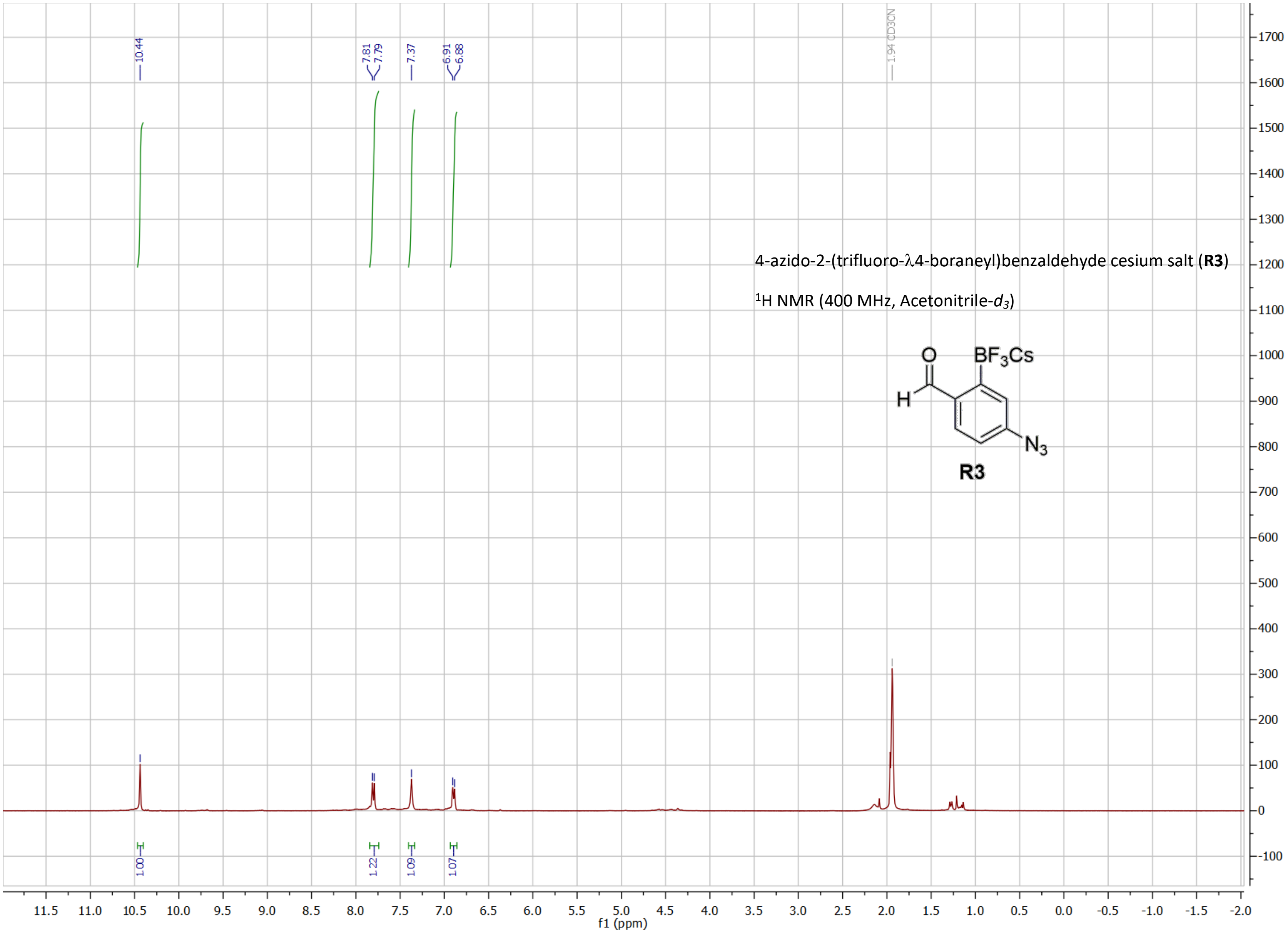
4-azido-2-(trifluoro-λ4-boraneyl)benzaldehyde cesium salt (**R3**)
¹H NMR (400 MHz, Acetonitrile-$d_3$)
O
H
BF$_3$Cs
N$_3$
R3
10.44
7.81
7.79
7.37
6.91
6.88
1.94 CD3CN
1.00
1.22
1.09
1.07
f1 (ppm)

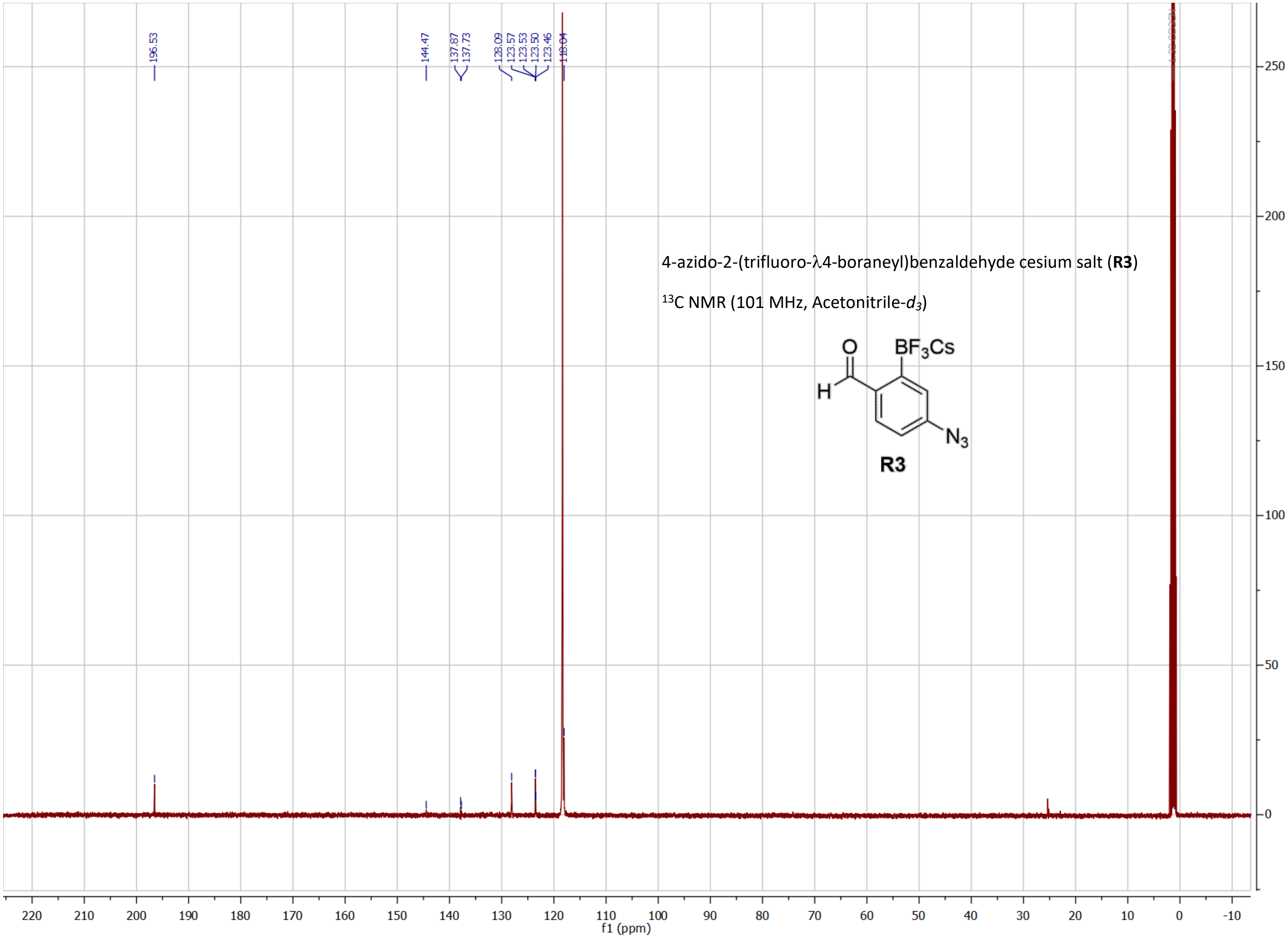

196.53
144.47
137.87
137.73
128.09
123.57
123.53
123.50
123.46
118.04
4-azido-2-(trifluoro-λ4-boraneyl)benzaldehyde cesium salt (R3)
13C NMR (101 MHz, Acetonitrile-d3)
O
H
BF3Cs
N3
R3
f1 (ppm)

4-azido-2-(trifluoro-λ4-boraneyl)benzaldehyde cesium salt (**R3**)

$^{19}$F NMR (376 MHz, Acetonitrile-$d_3$)

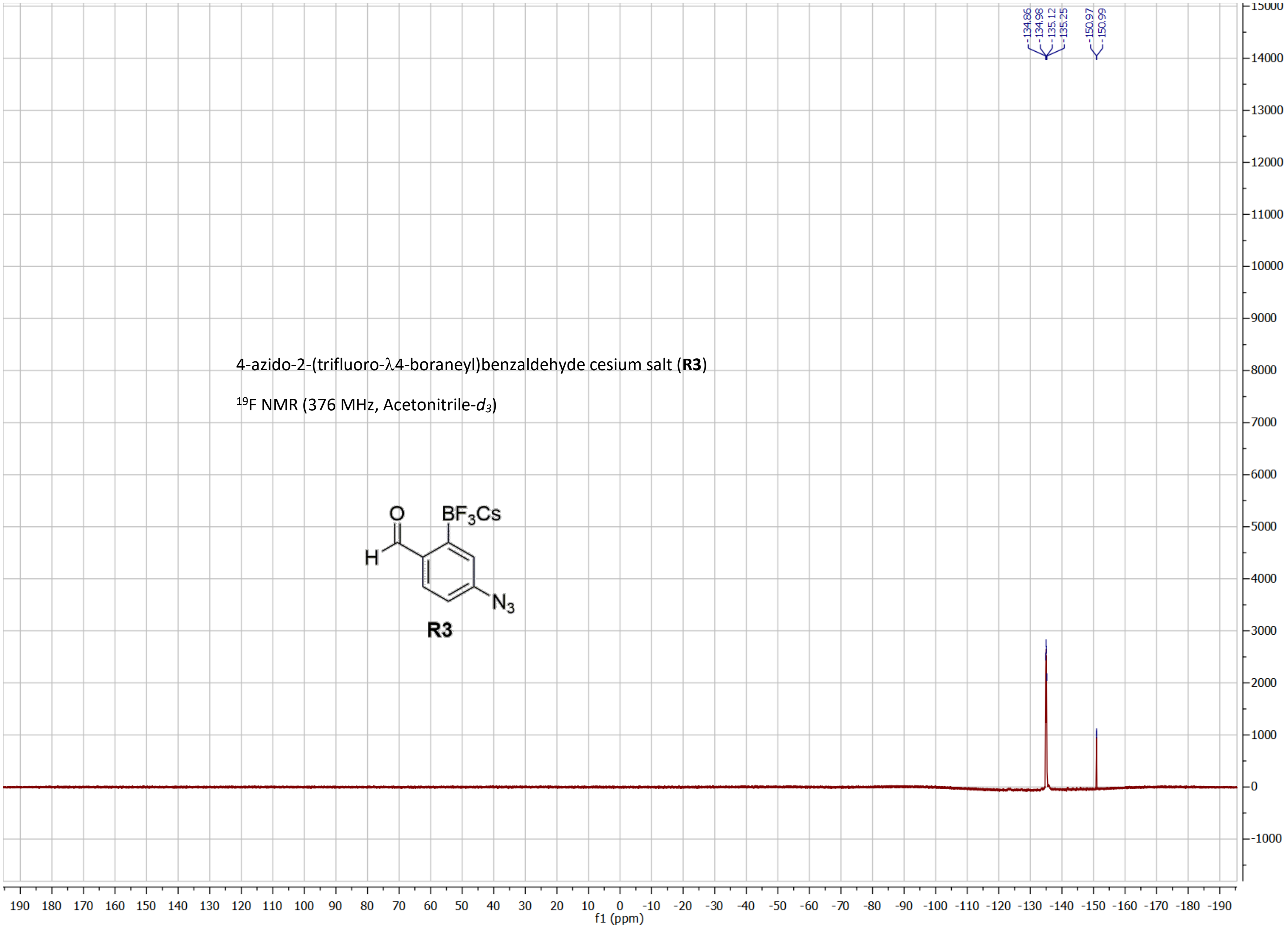

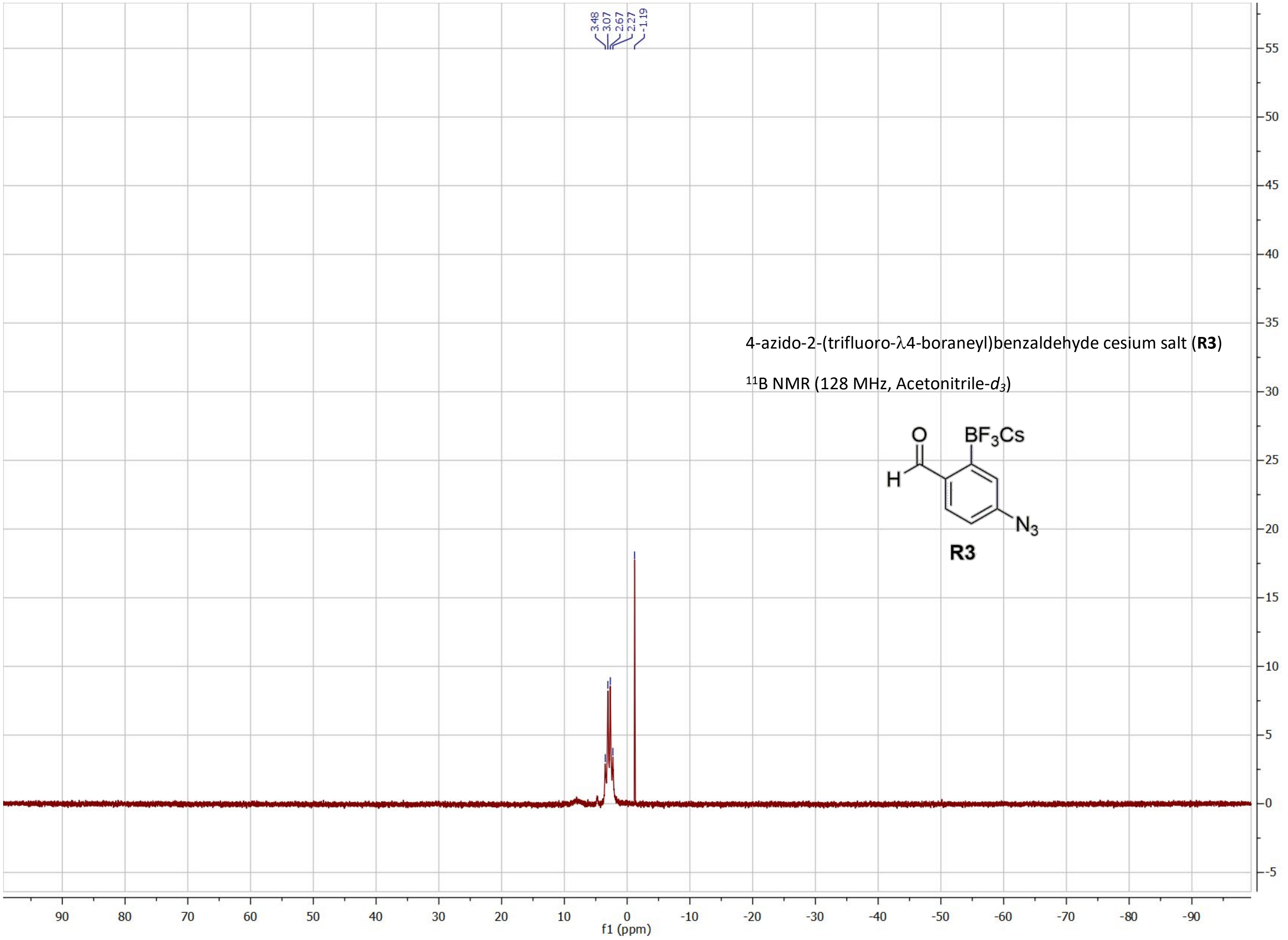


4-azido-2-(trifluoro-λ4-boraneyl)benzaldehyde cesium salt (**R3**)

$^{11}$B NMR (128 MHz, Acetonitrile-$d_3$)

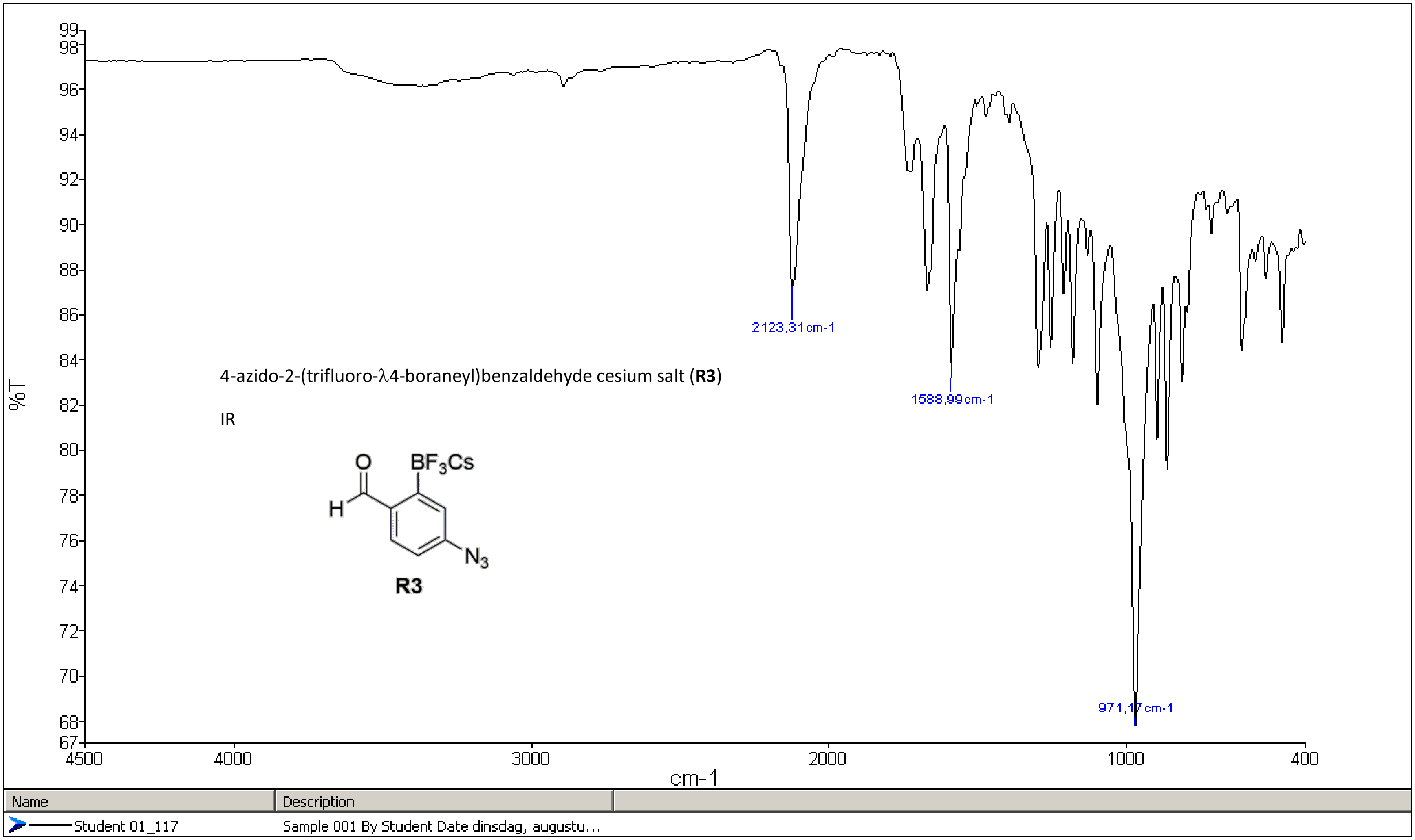

99
98
96
94
92
90
88
86
84
82
80
78
76
74
72
70
68
67
%T
4500
4000
3000
2000
1000
400
cm-1
2123,31cm-1
1588,99cm-1
971,17cm-1
4-azido-2-(trifluoro-λ4-boraneyl)benzaldehyde cesium salt (**R3**)
IR
O
H
$BF_3Cs$
$N_3$
R3
Name
Description
Student 01_117
Sample 001 By Student Date dinsdag, augustu...

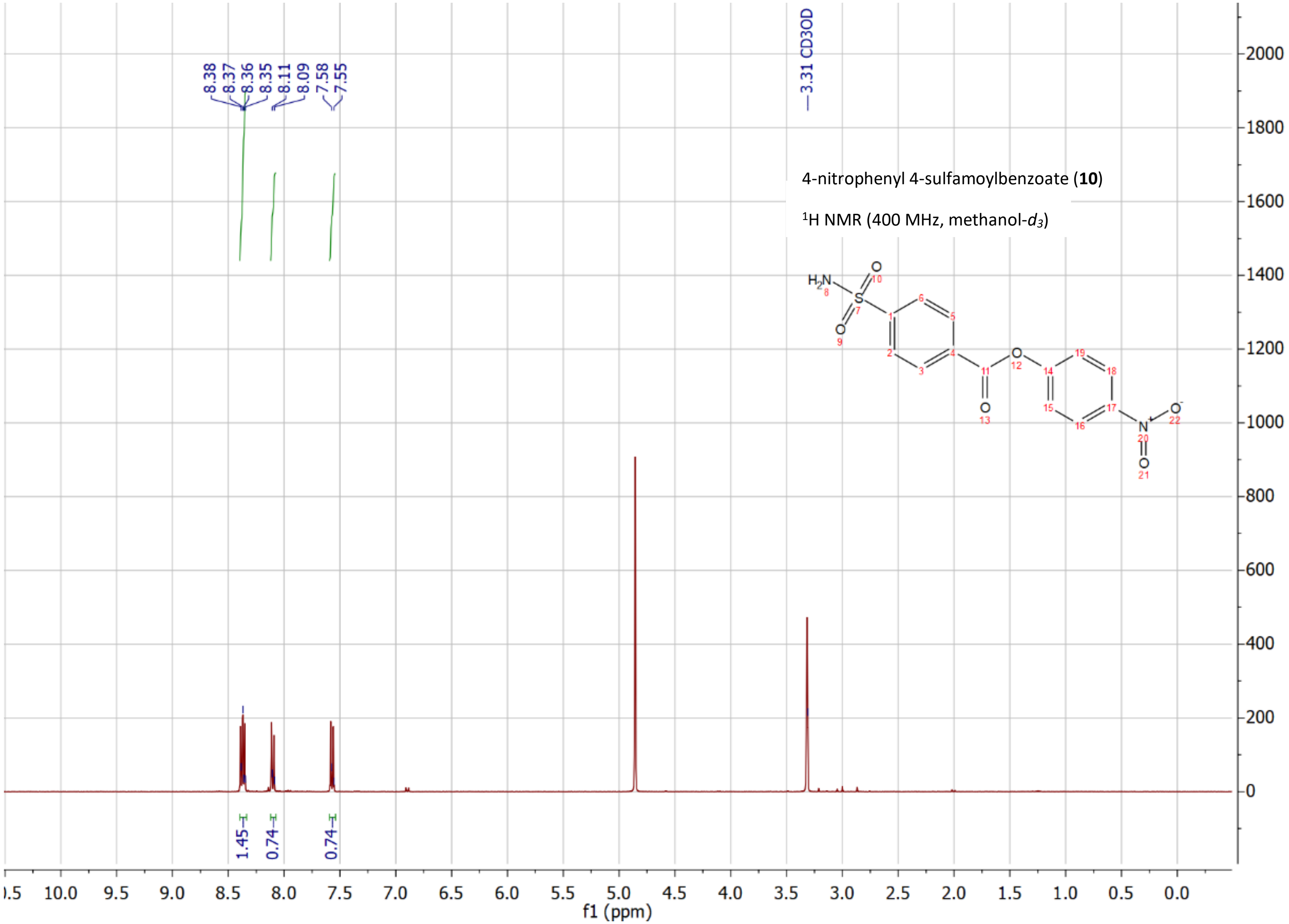
4-nitrophenyl 4-sulfamoylbenzoate (10)
$^1$H NMR (400 MHz, methanol-$d_3$)
8.38
8.37
8.36
8.35
8.11
8.09
7.58
7.55
3.31 CD3OD
1.45
0.74
0.74
f1 (ppm)

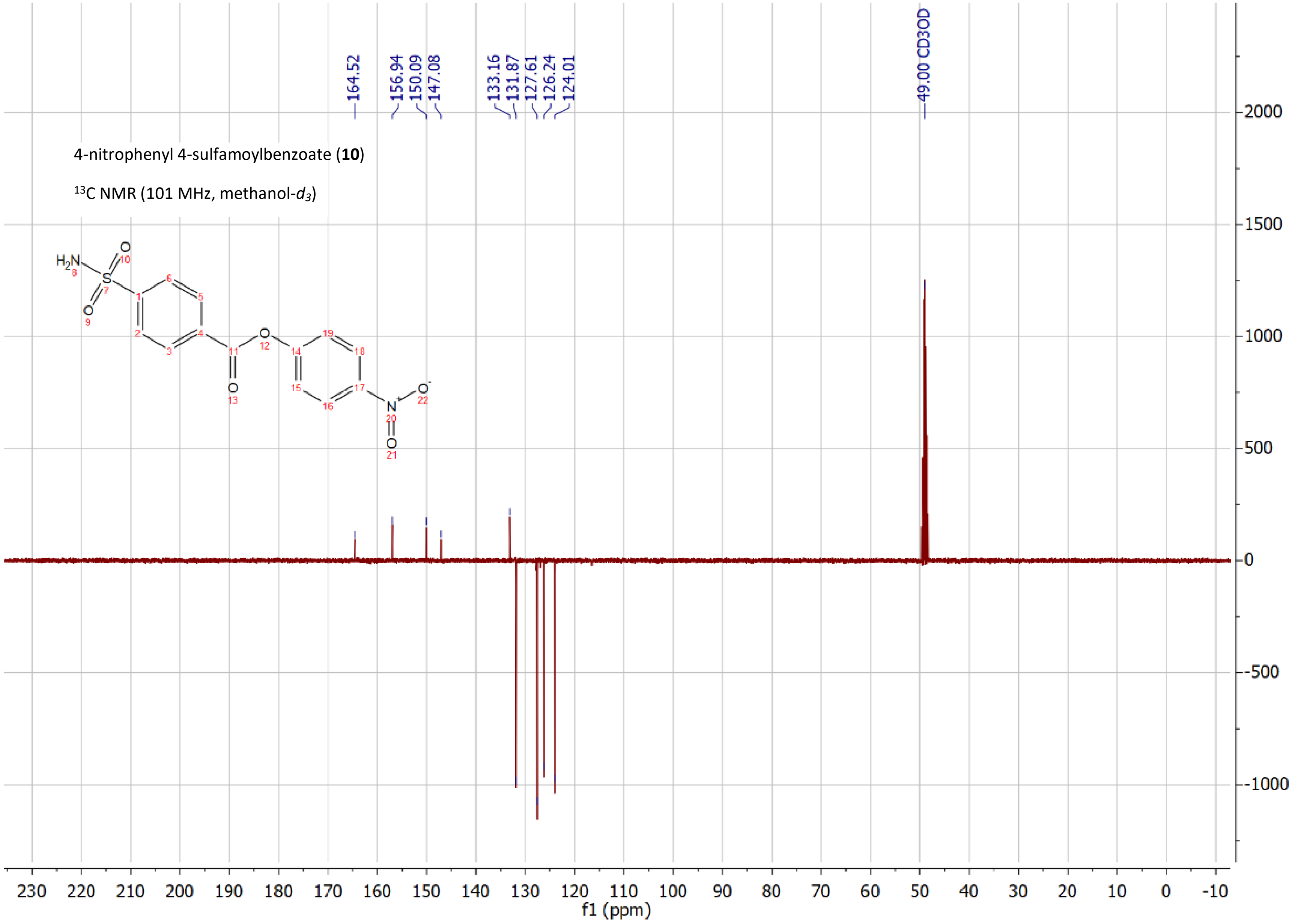

164.52
156.94
150.09
147.08
133.16
131.87
127.61
126.24
124.01
49.00 CD3OD
4-nitrophenyl 4-sulfamoylbenzoate (**10**)
13C NMR (101 MHz, methanol-*d3*)
f1 (ppm)

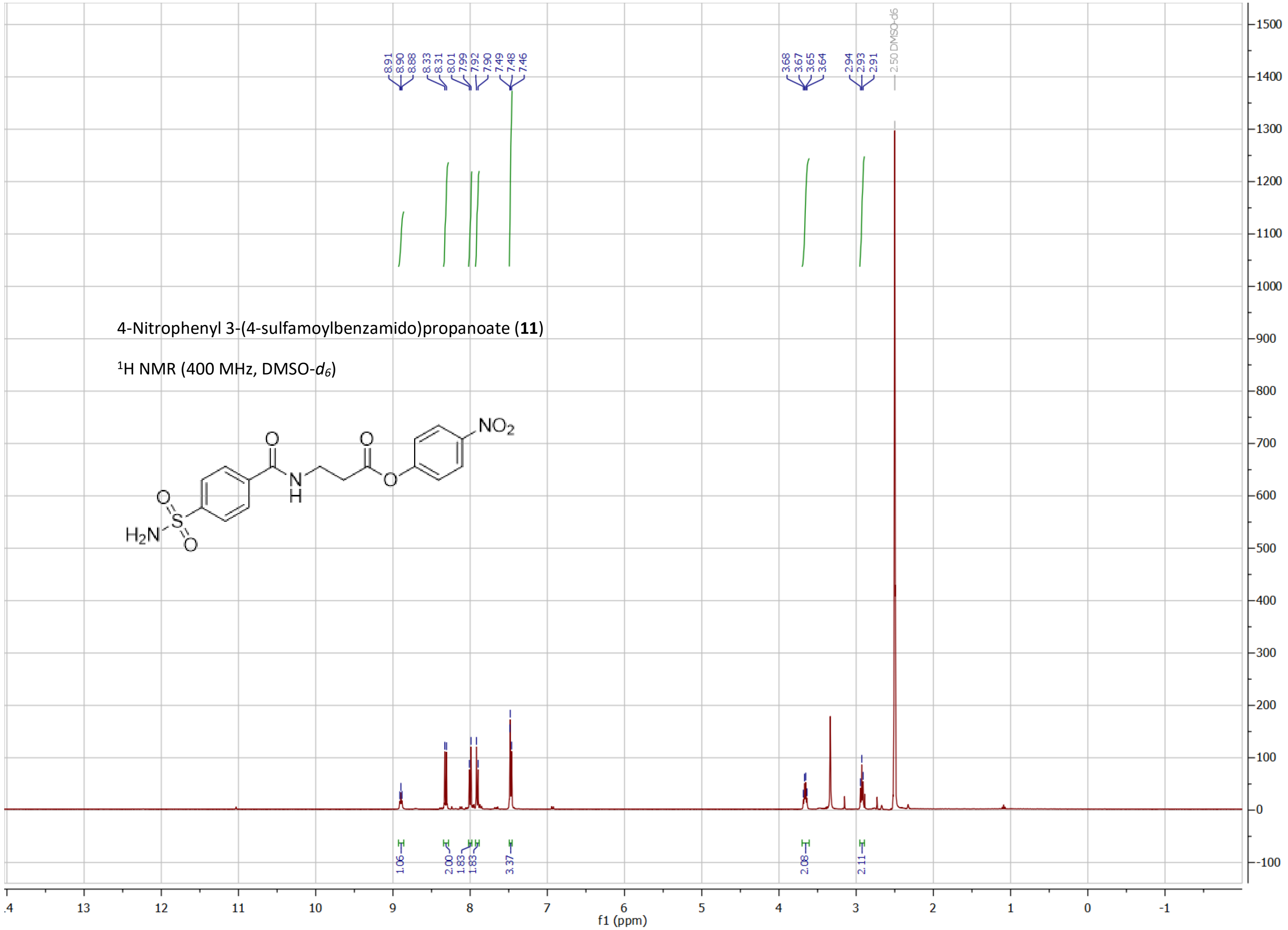
4-Nitrophenyl 3-(4-sulfamoylbenzamido)propanoate (**11**)
$^1$H NMR (400 MHz, DMSO-$d_6$)
NO2
H2N
O
S
N
H
8.91
8.90
8.88
8.33
8.31
8.01
7.99
7.92
7.90
7.49
7.48
7.46
3.68
3.67
3.65
3.64
2.94
2.93
2.91
2.50 DMSO-d6
1.06
2.00
1.83
1.83
3.37
2.08
2.11
f1 (ppm)

4-Nitrophenyl 3-(4-sulfamoylbenzamido)propanoate (**11**)

$^{13}$C NMR (101 MHz, DMSO-$d_6$)

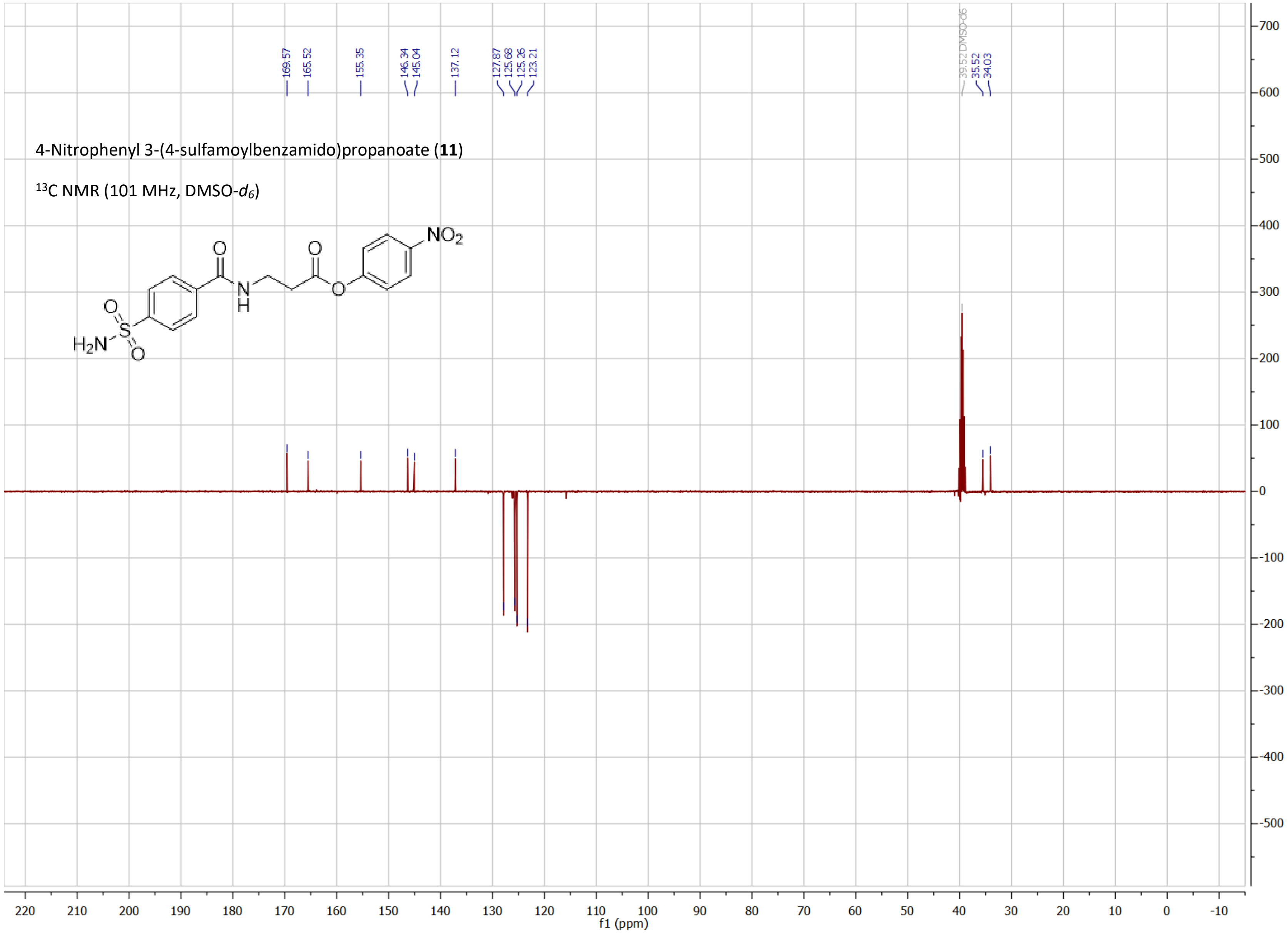

4-Nitrophenyl 4-(4-sulfamoylbenzamido)butanoate (**12**)

$^1$H NMR (400 MHz, DMSO-$d_6$)

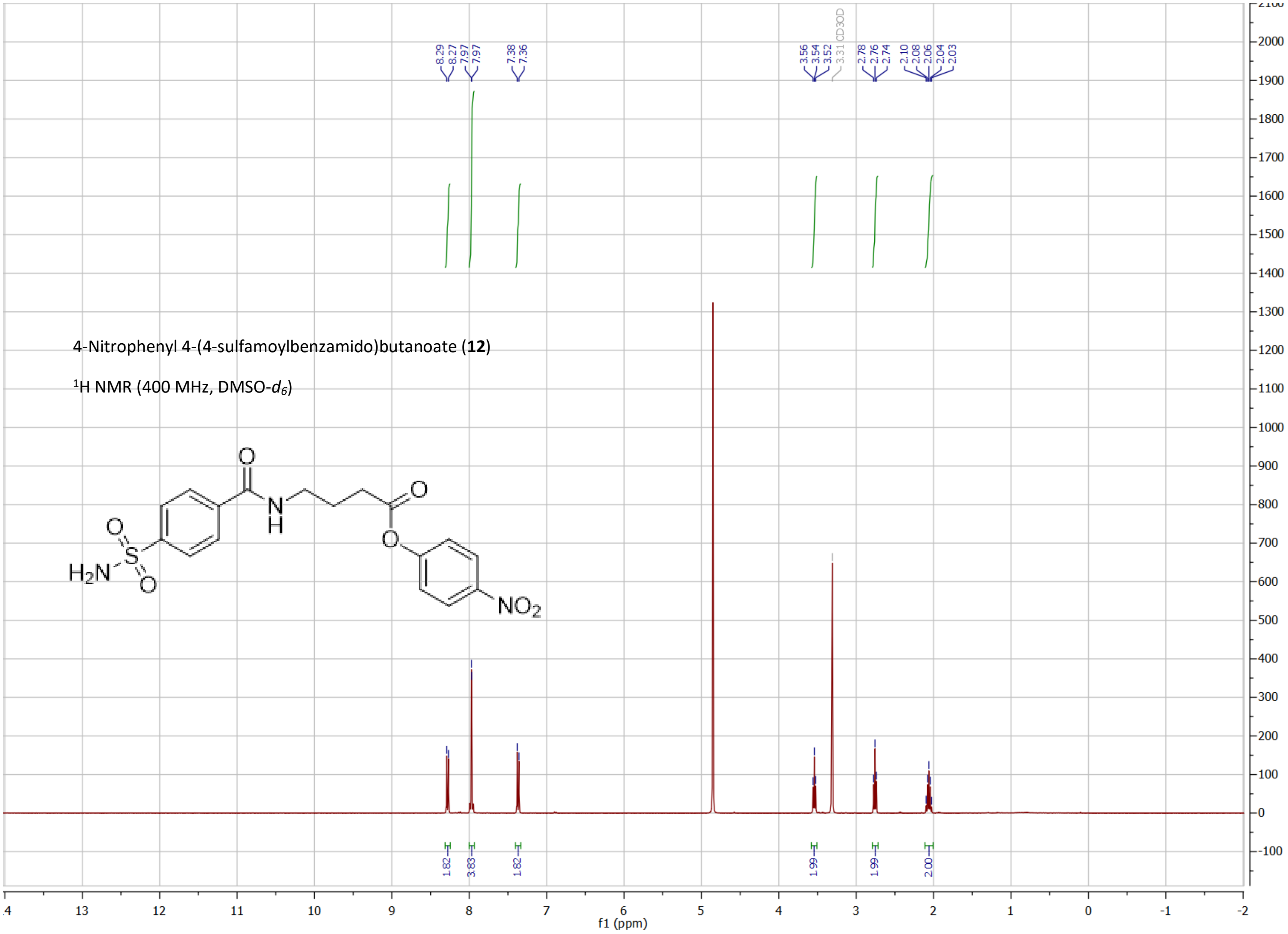

4-Nitrophenyl 4-(4-sulfamoylbenzamido)butanoate (**12**)

$^{13}$C NMR (101 MHz, DMSO-$d_6$)

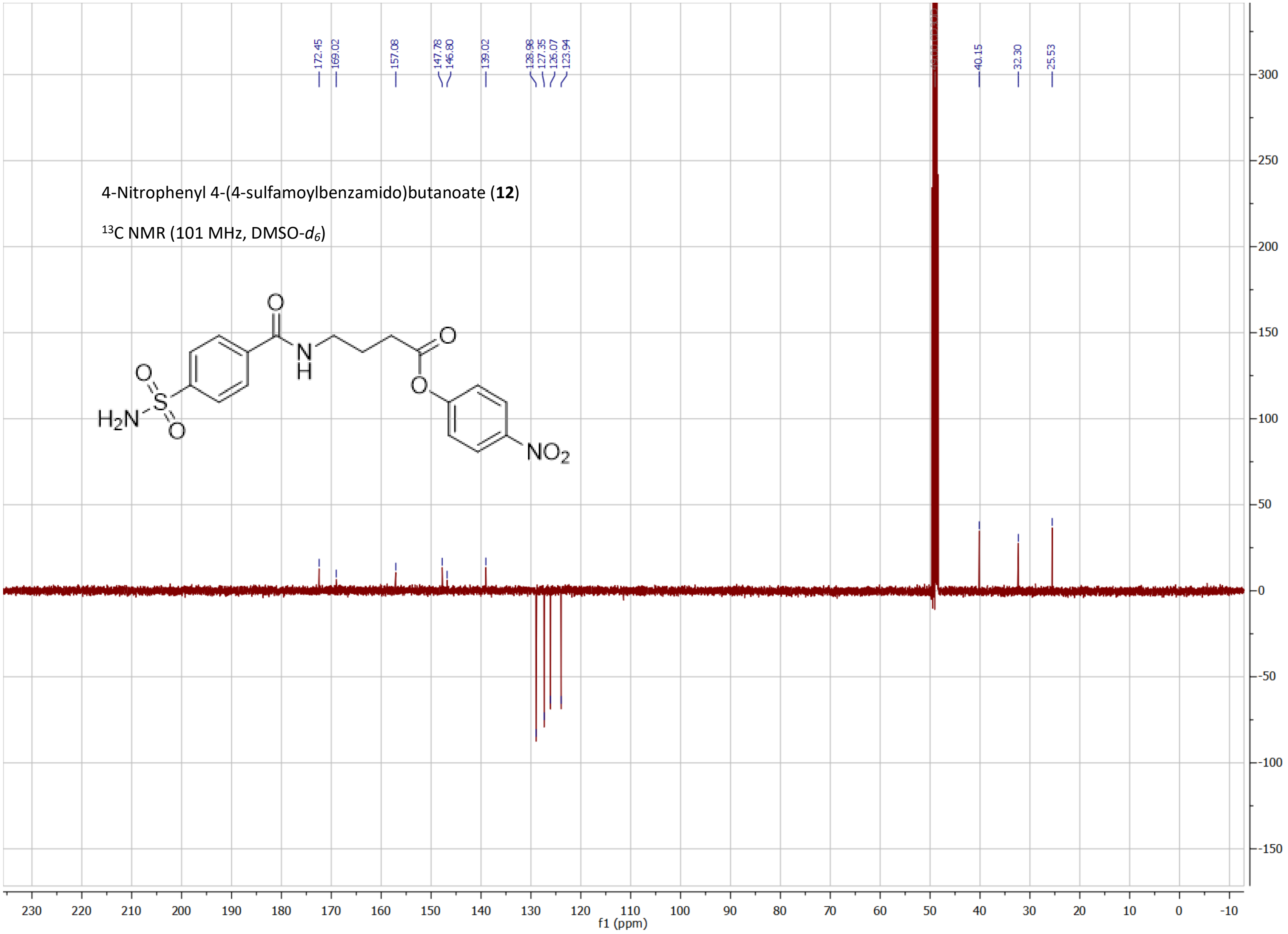

4-Nitrophenyl 5-(4-sulfamoylbenzamido)pentanoate (**13**)

$^{1}$H NMR (400 MHz, DMSO-$d_6$)

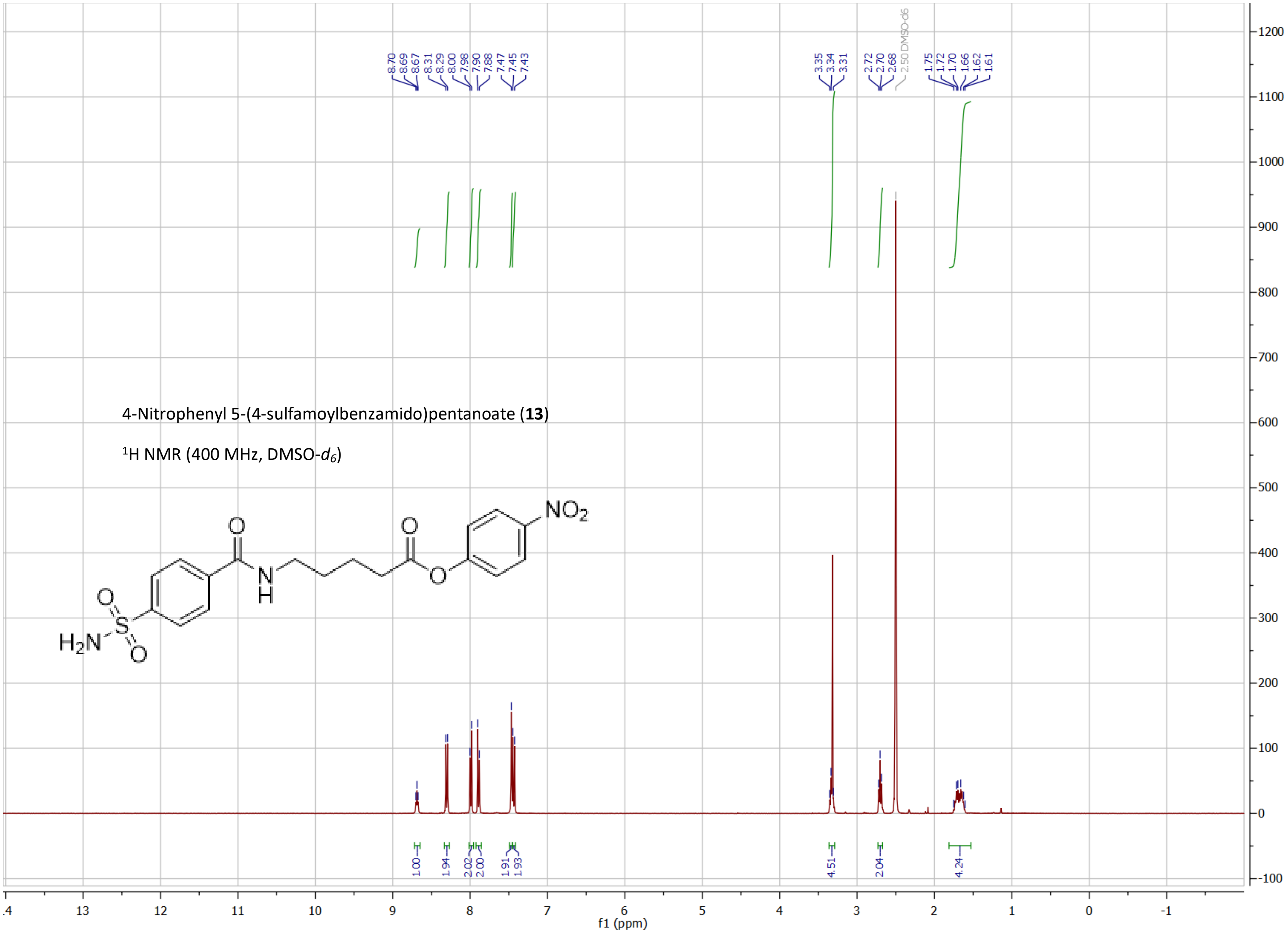

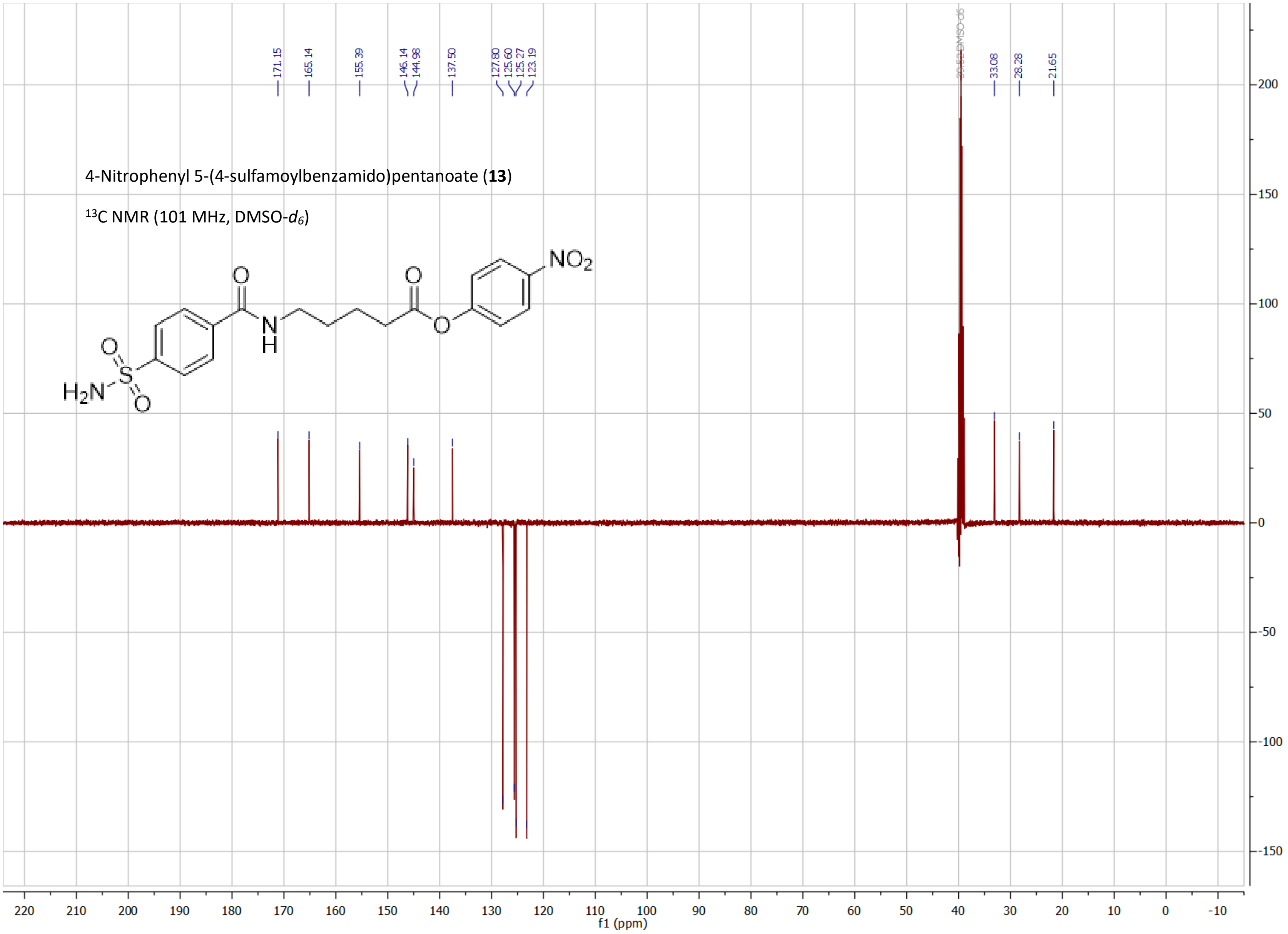

4-Nitrophenyl 5-(4-sulfamoylbenzamido)pentanoate (**13**)
$^{13}C$ NMR (101 MHz, DMSO-$d_6$)
171.15
165.14
155.39
146.14
144.98
137.50
127.80
125.60
125.27
123.19
DMSO-d6
33.08
28.28
21.65
NO2
H2N
f1 (ppm)

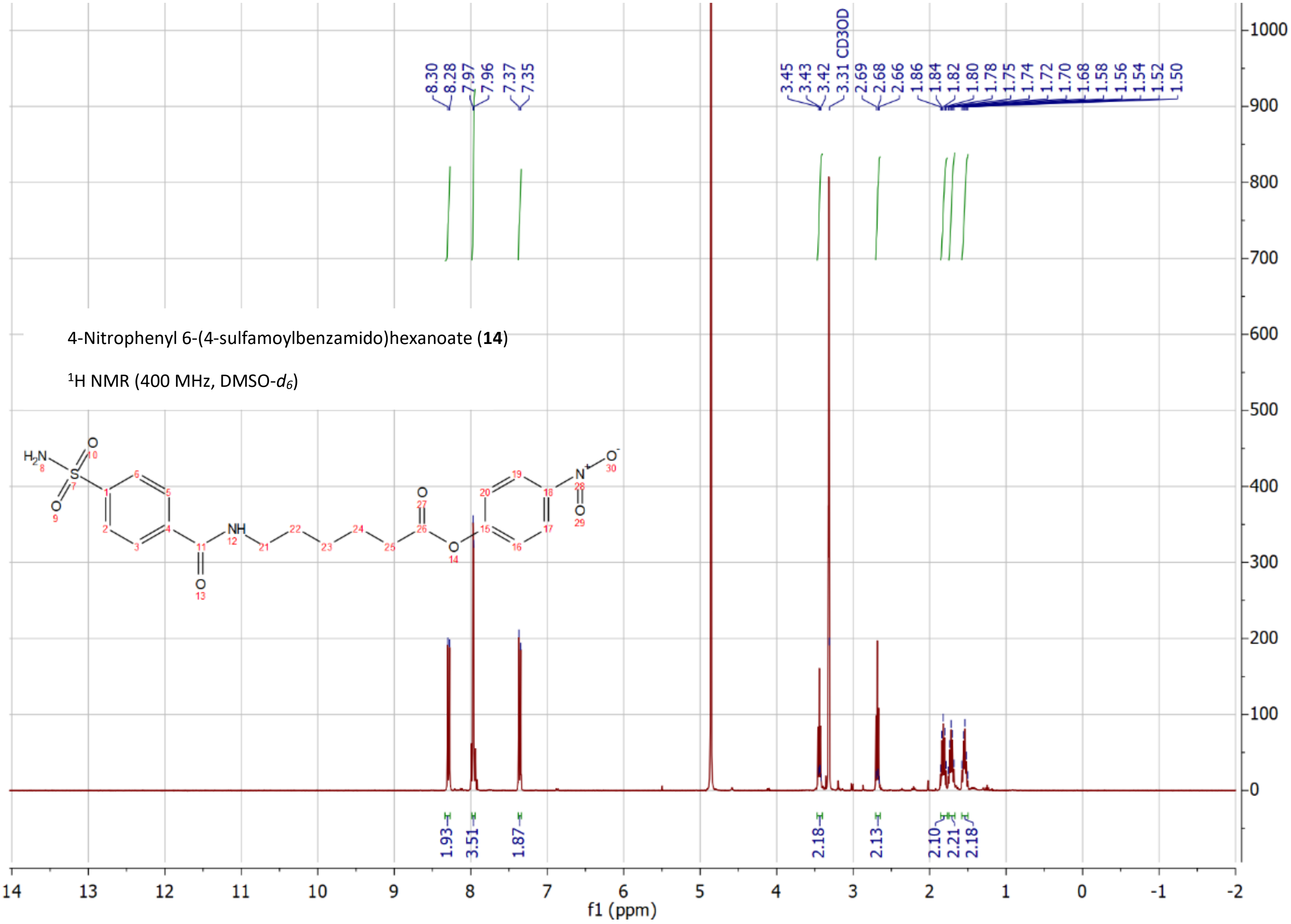
4-Nitrophenyl 6-(4-sulfamoylbenzamido)hexanoate (**14**)
$^{1}$H NMR (400 MHz, DMSO-$d_6$)
8.30
8.28
7.97
7.96
7.37
7.35
3.45
3.43
3.42
3.31 CD3OD
2.69
2.68
2.66
1.86
1.84
1.82
1.80
1.78
1.75
1.74
1.72
1.70
1.68
1.58
1.56
1.54
1.52
1.50
1.93
3.51
1.87
2.18
2.13
2.10
2.21
2.18
f1 (ppm)

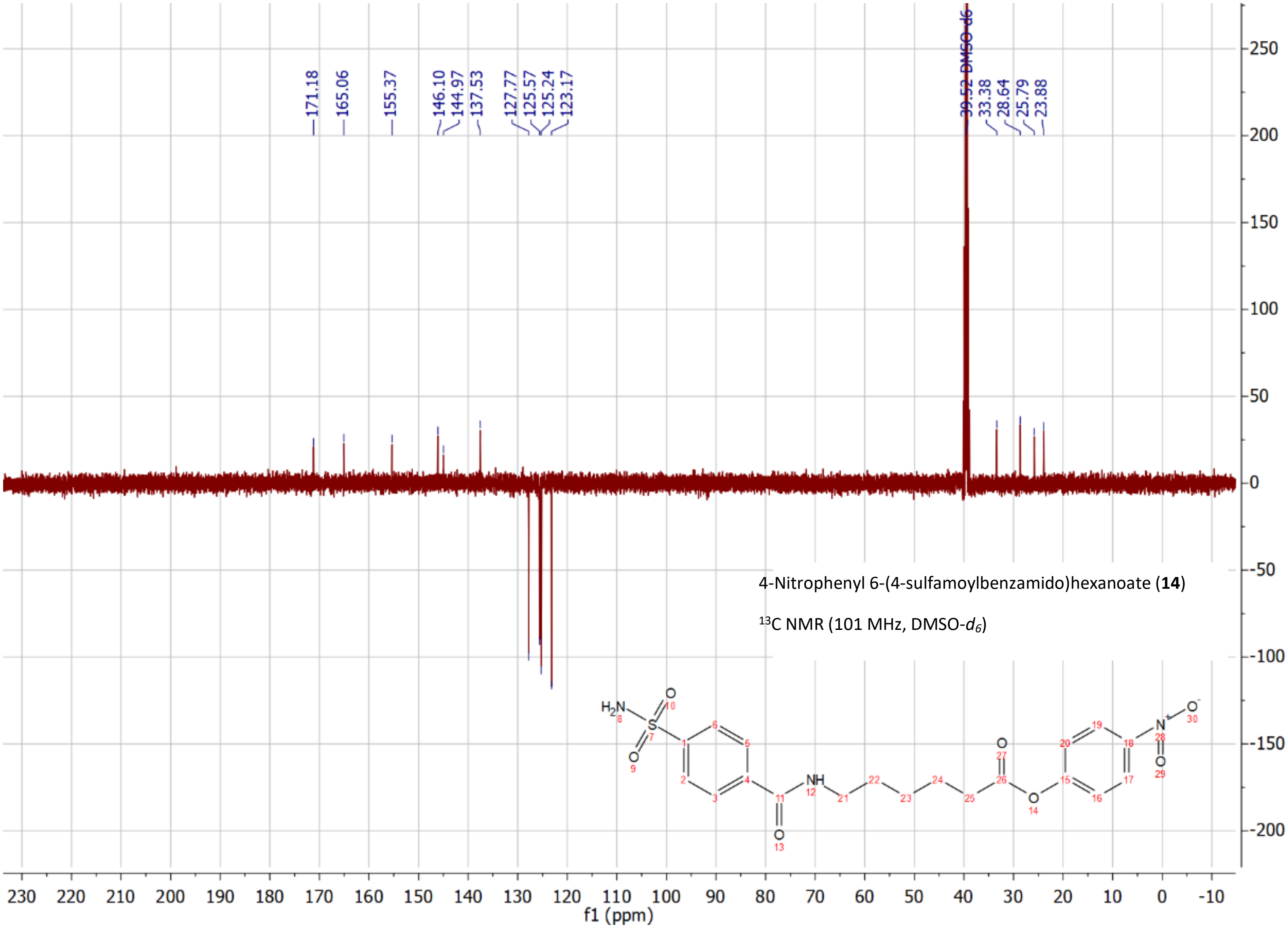
171.18
165.06
155.37
146.10
144.97
137.53
127.77
125.57
125.24
123.17
39.52 DMSO-d6
33.38
28.64
25.79
23.88
4-Nitrophenyl 6-(4-sulfamoylbenzamido)hexanoate (**14**)
$^{13}$C NMR (101 MHz, DMSO-$d_6$)
f1 (ppm)

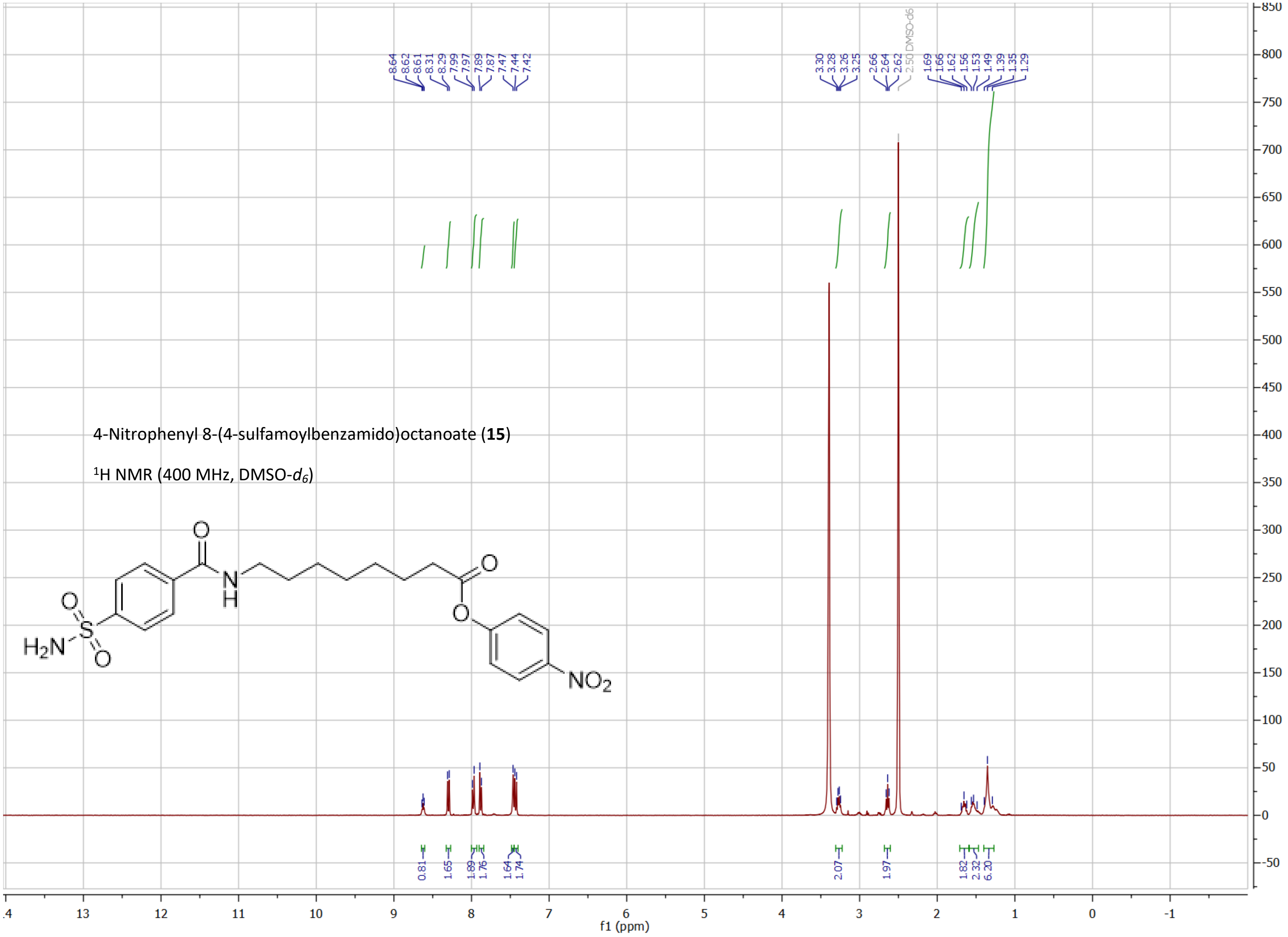
4-Nitrophenyl 8-(4-sulfamoylbenzamido)octanoate (**15**)
$^1$H NMR (400 MHz, DMSO-$d_6$)
8.64 8.62 8.61 8.31 8.29 7.99 7.97 7.89 7.87 7.47 7.44 7.42
3.30 3.28 3.26 3.25 2.66 2.64 2.62 2.50 DMSO-d6 1.69 1.66 1.62 1.56 1.53 1.49 1.39 1.35 1.29
0.81 1.65 1.89 1.76 1.64 1.74 2.07 1.97 1.82 2.32 6.20
$H_2N$
$NO_2$
f1 (ppm)

4-Nitrophenyl 8-(4-sulfamoylbenzamido)octanoate (**15**)

$^{13}C$ NMR (101 MHz, DMSO-$d_6$)

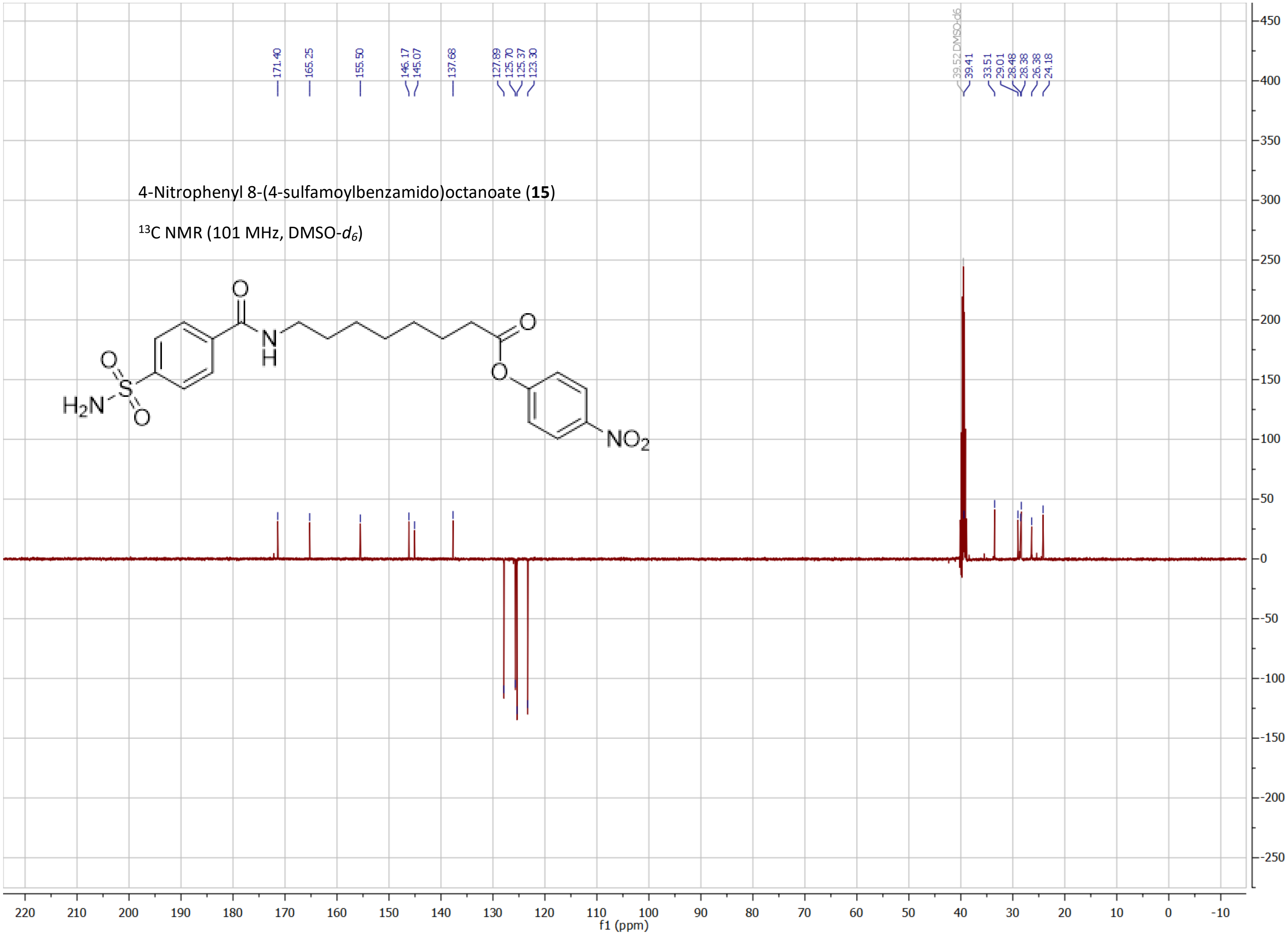

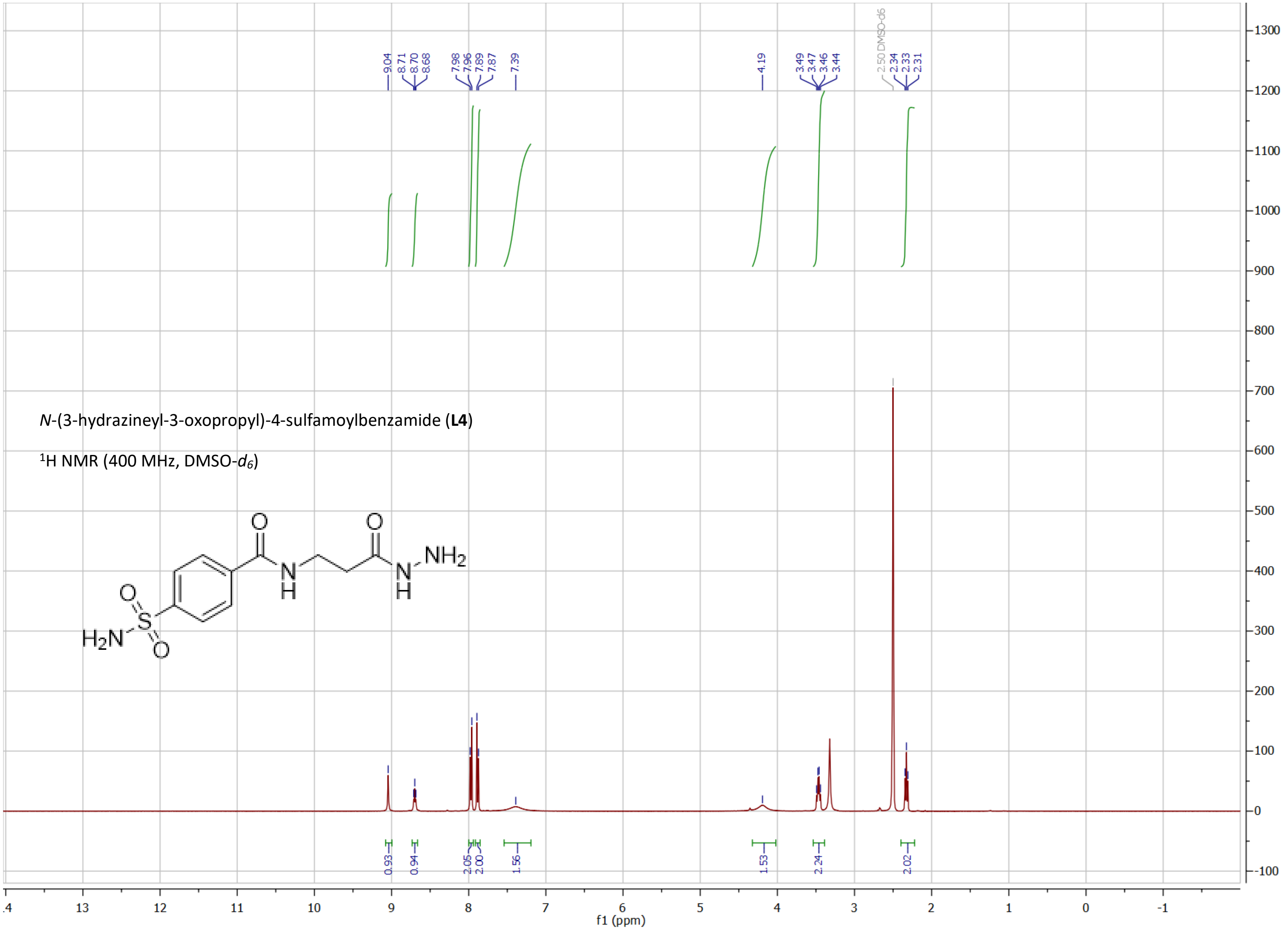
N-(3-hydrazineyl-3-oxopropyl)-4-sulfamoylbenzamide (L4)
1H NMR (400 MHz, DMSO-d6)
9.04
8.71
8.70
8.68
7.98
7.96
7.89
7.87
7.39
4.19
3.49
3.47
3.46
3.44
2.50 DMSO-d6
2.34
2.33
2.31
0.93
0.94
2.05
2.00
1.56
1.53
2.24
2.02
f1 (ppm)

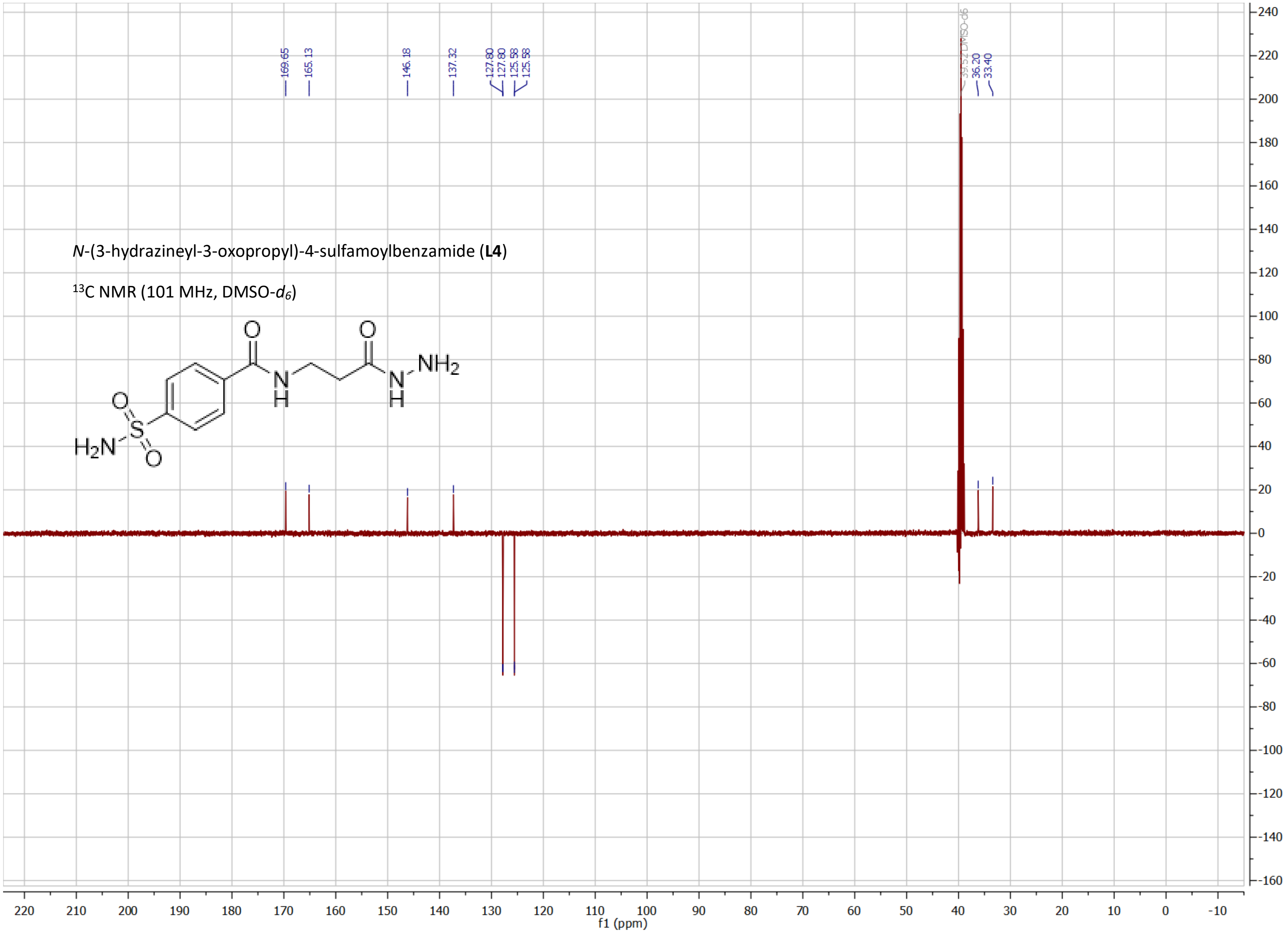
N-(3-hydrazineyl-3-oxopropyl)-4-sulfamoylbenzamide (L4)
13C NMR (101 MHz, DMSO-d6)
169.65
165.13
146.18
137.32
127.80
127.80
125.58
125.58
39.52 DMSO-d6
36.20
33.40
f1 (ppm)

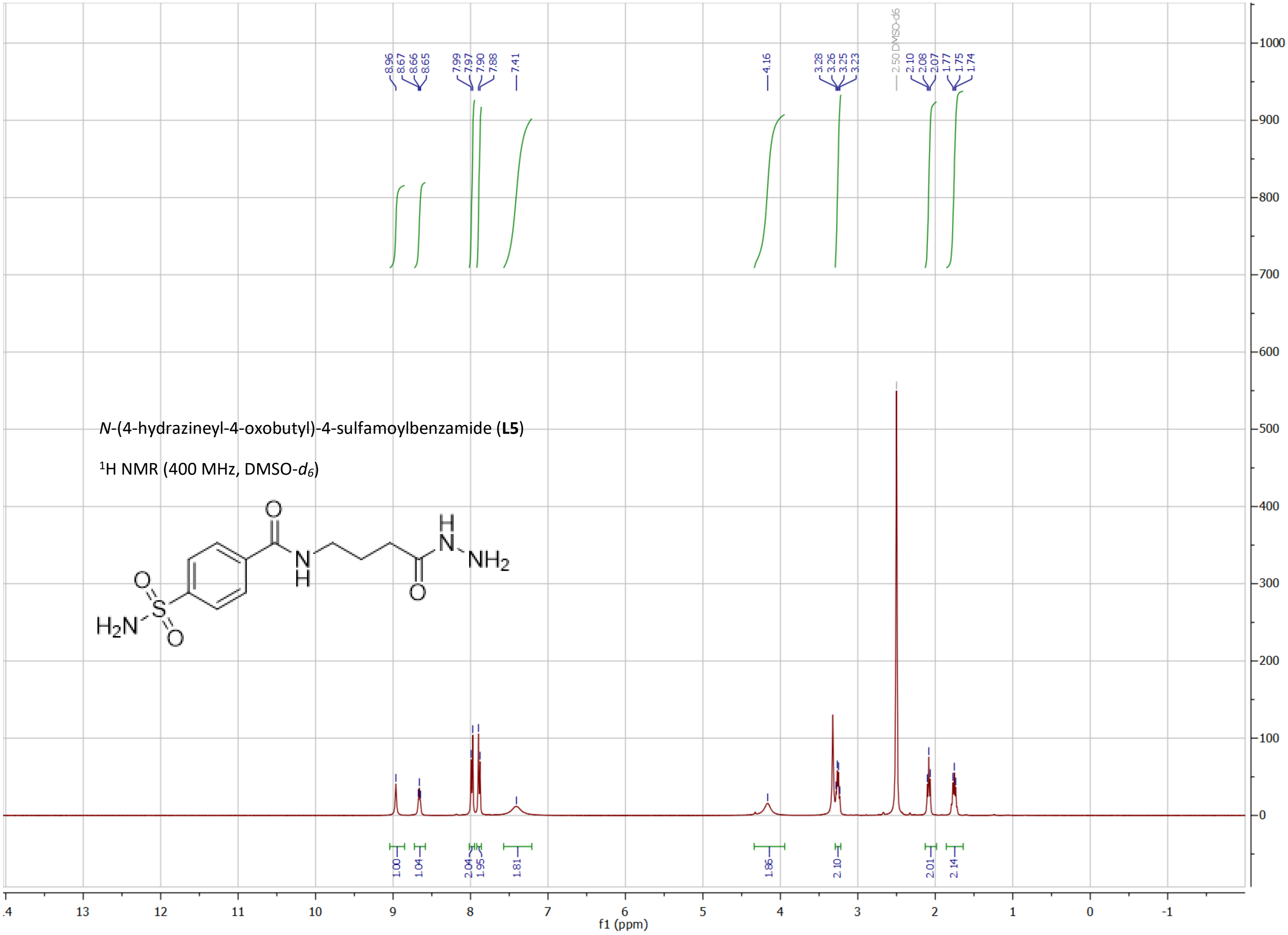
8.96
8.67
8.66
8.65
7.99
7.97
7.90
7.88
7.41
4.16
3.28
3.26
3.25
3.23
2.50 DMSO-d6
2.10
2.08
2.07
1.77
1.75
1.74
N-(4-hydrazineyl-4-oxobutyl)-4-sulfamoylbenzamide (L5)
1H NMR (400 MHz, DMSO-d6)
1.00
1.04
2.04
1.95
1.81
1.86
2.10
2.01
2.14
f1 (ppm)

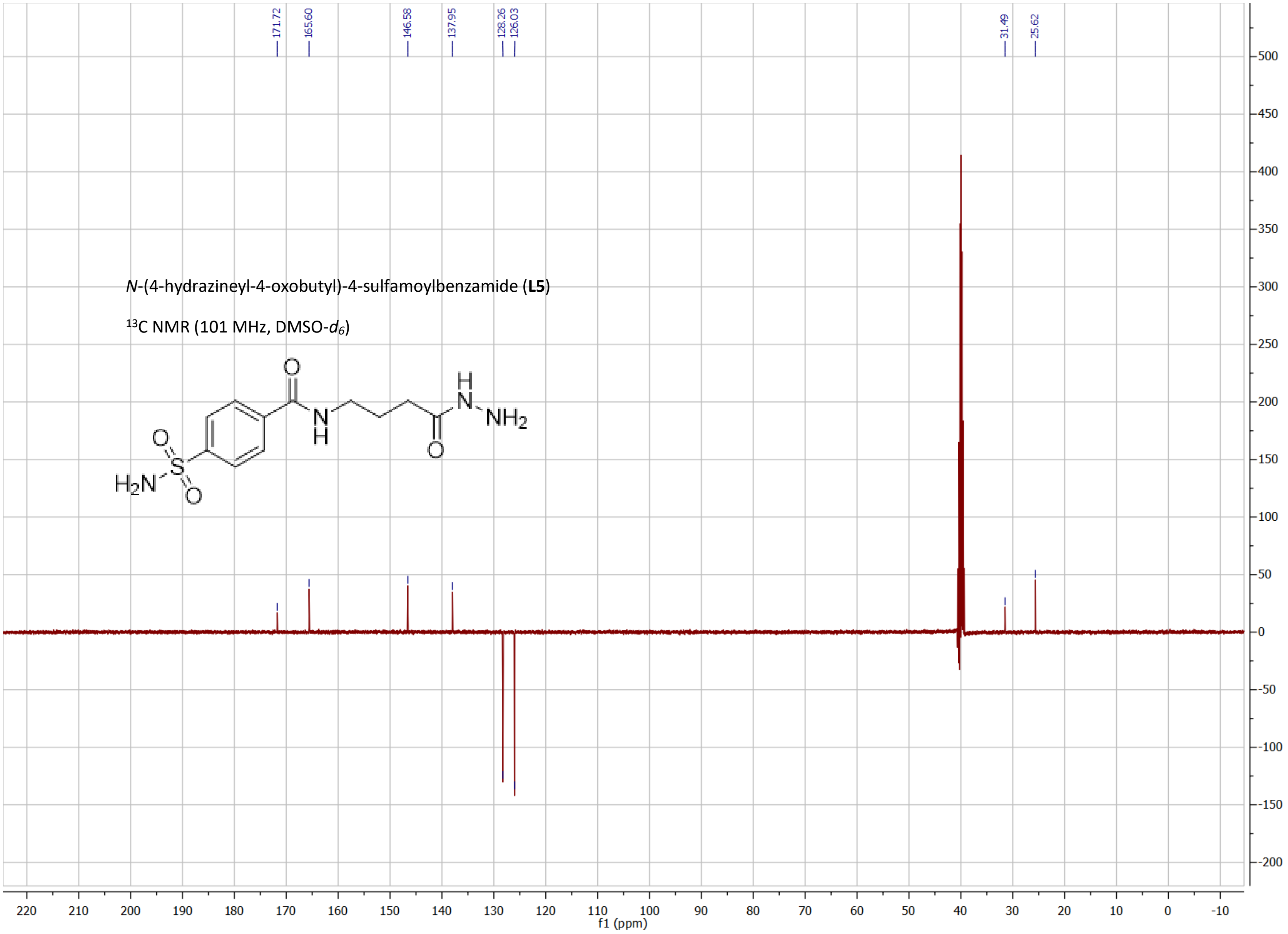

N-(4-hydrazineyl-4-oxobutyl)-4-sulfamoylbenzamide (L5)
13C NMR (101 MHz, DMSO-d6)
171.72
165.60
146.58
137.95
128.26
126.03
31.49
25.62
f1 (ppm)

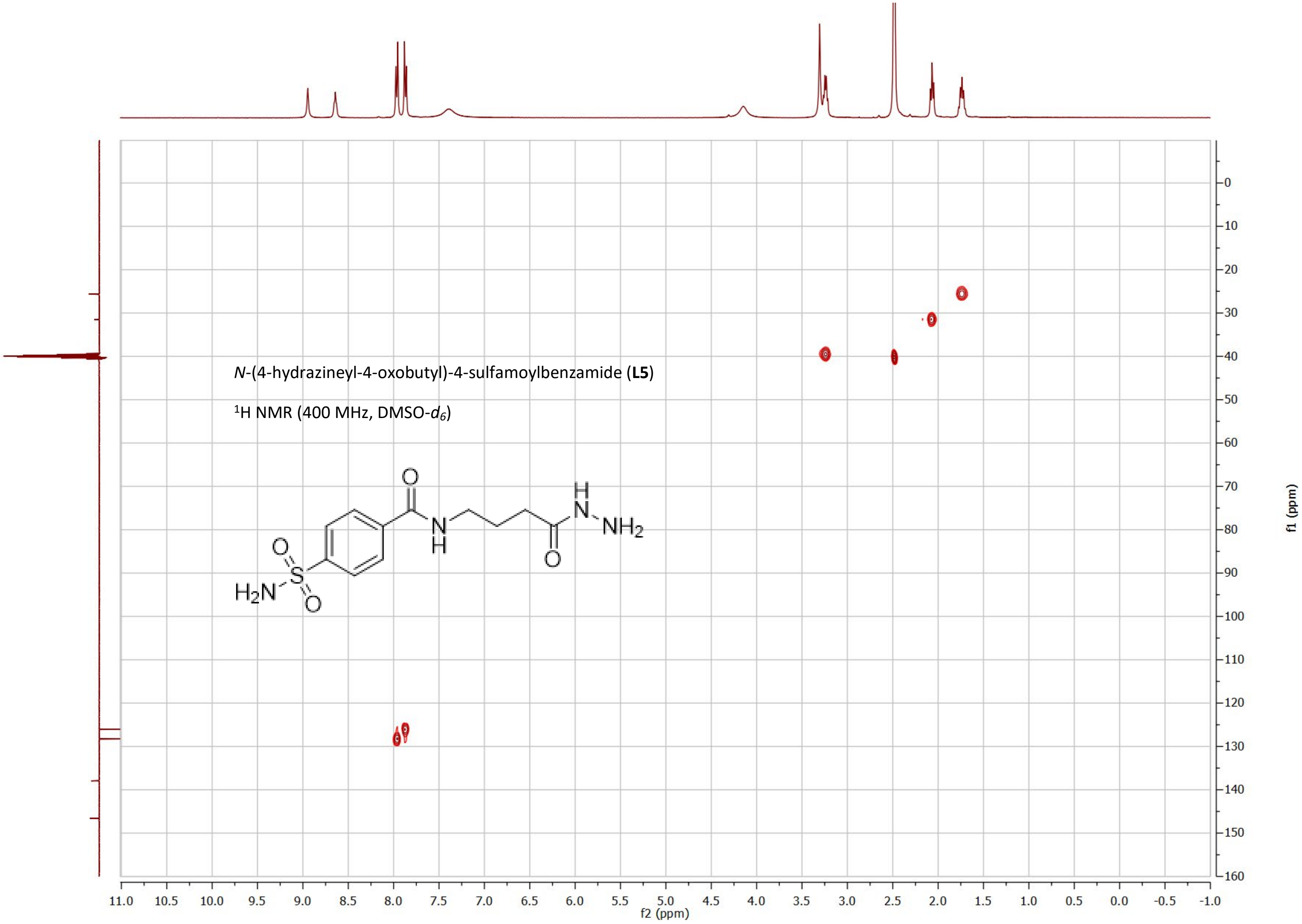
N-(4-hydrazineyl-4-oxobutyl)-4-sulfamoylbenzamide (L5)
1H NMR (400 MHz, DMSO-d6)
f2 (ppm)
f1 (ppm)

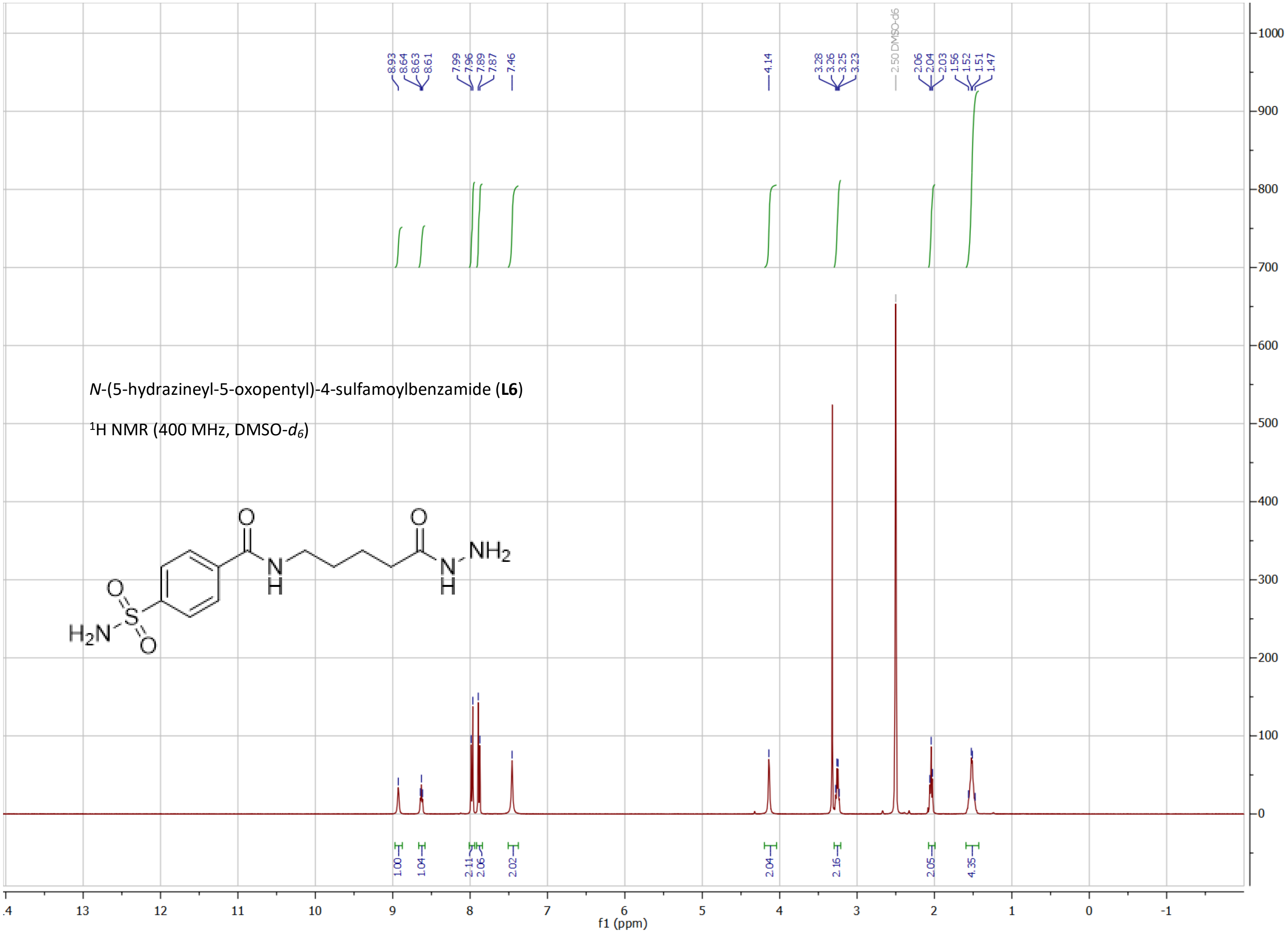
*N*-(5-hydrazineyl-5-oxopentyl)-4-sulfamoylbenzamide (**L6**)
$^{1}$H NMR (400 MHz, DMSO-$d_6$)
8.93
8.64
8.63
8.61
7.99
7.96
7.89
7.87
7.46
4.14
3.28
3.26
3.25
3.23
2.50 DMSO-d6
2.06
2.04
2.03
1.56
1.52
1.51
1.47
1.00
1.04
2.11
2.06
2.02
2.04
2.16
2.05
4.35
f1 (ppm)

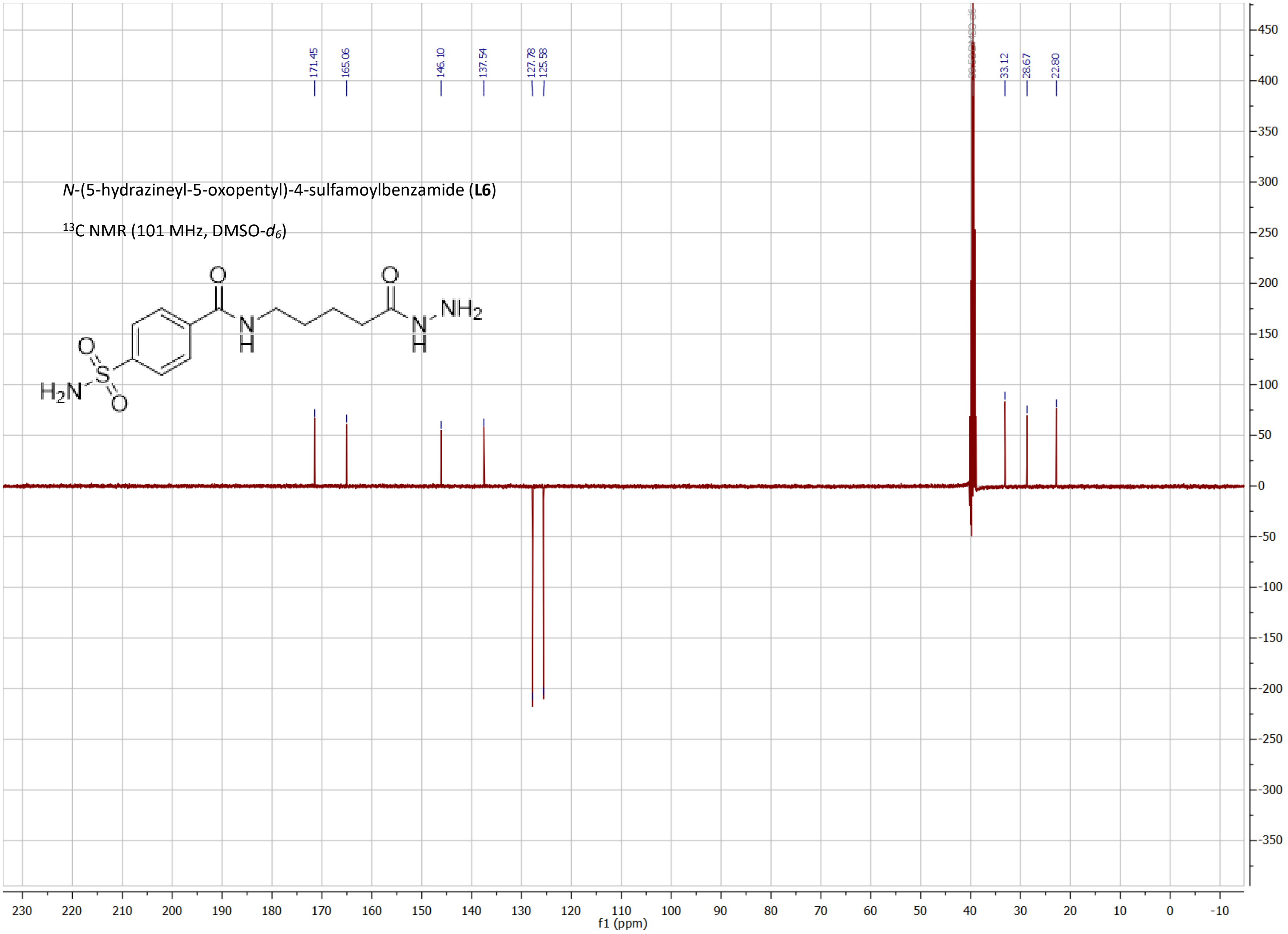
N-(5-hydrazineyl-5-oxopentyl)-4-sulfamoylbenzamide (L6)
13C NMR (101 MHz, DMSO-d6)
171.45
165.06
146.10
137.54
127.78
125.58
33.12
28.67
22.80
f1 (ppm)

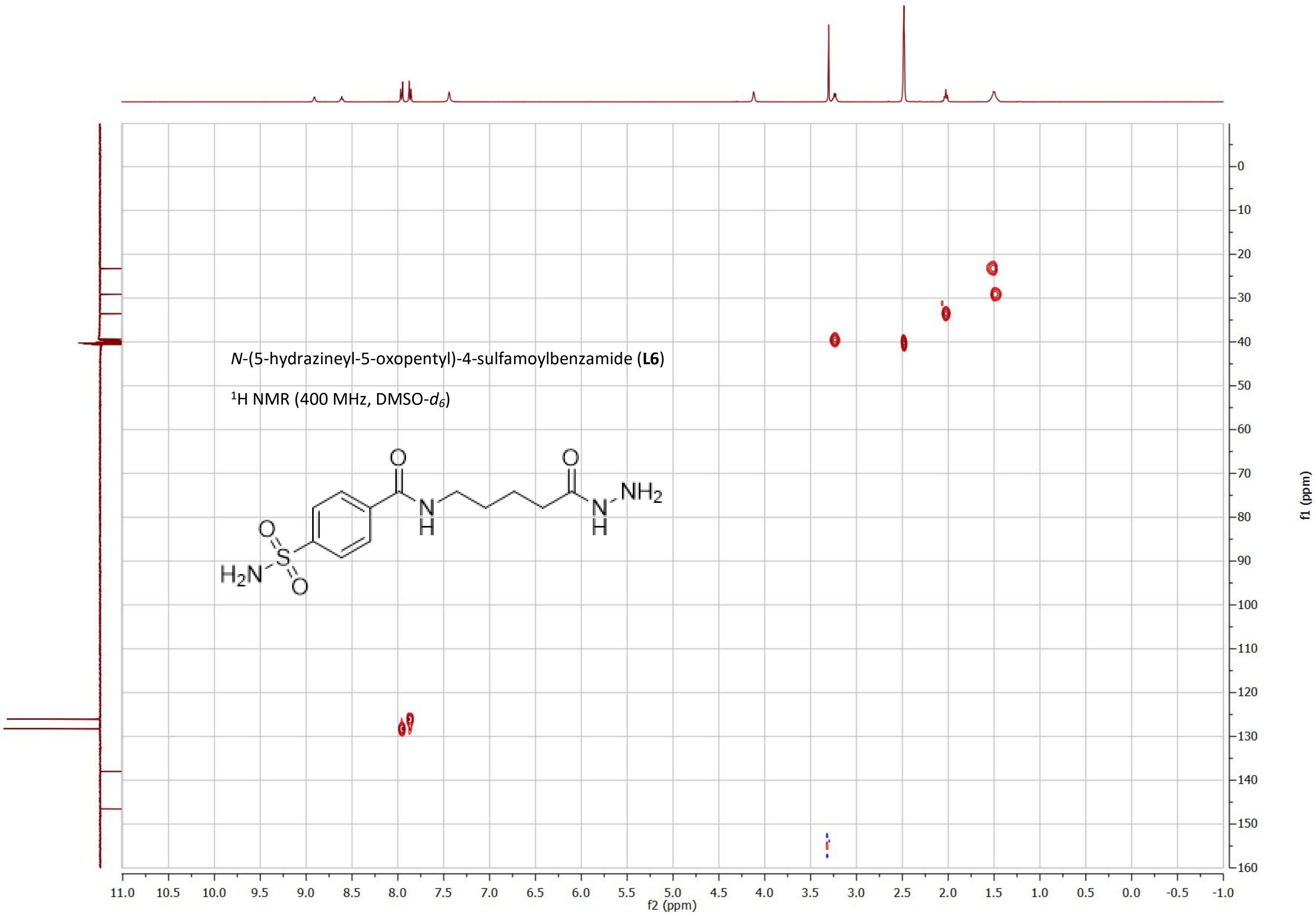
N-(5-hydrazineyl-5-oxopentyl)-4-sulfamoylbenzamide (L6)
1H NMR (400 MHz, DMSO-d6)
f2 (ppm)
f1 (ppm)

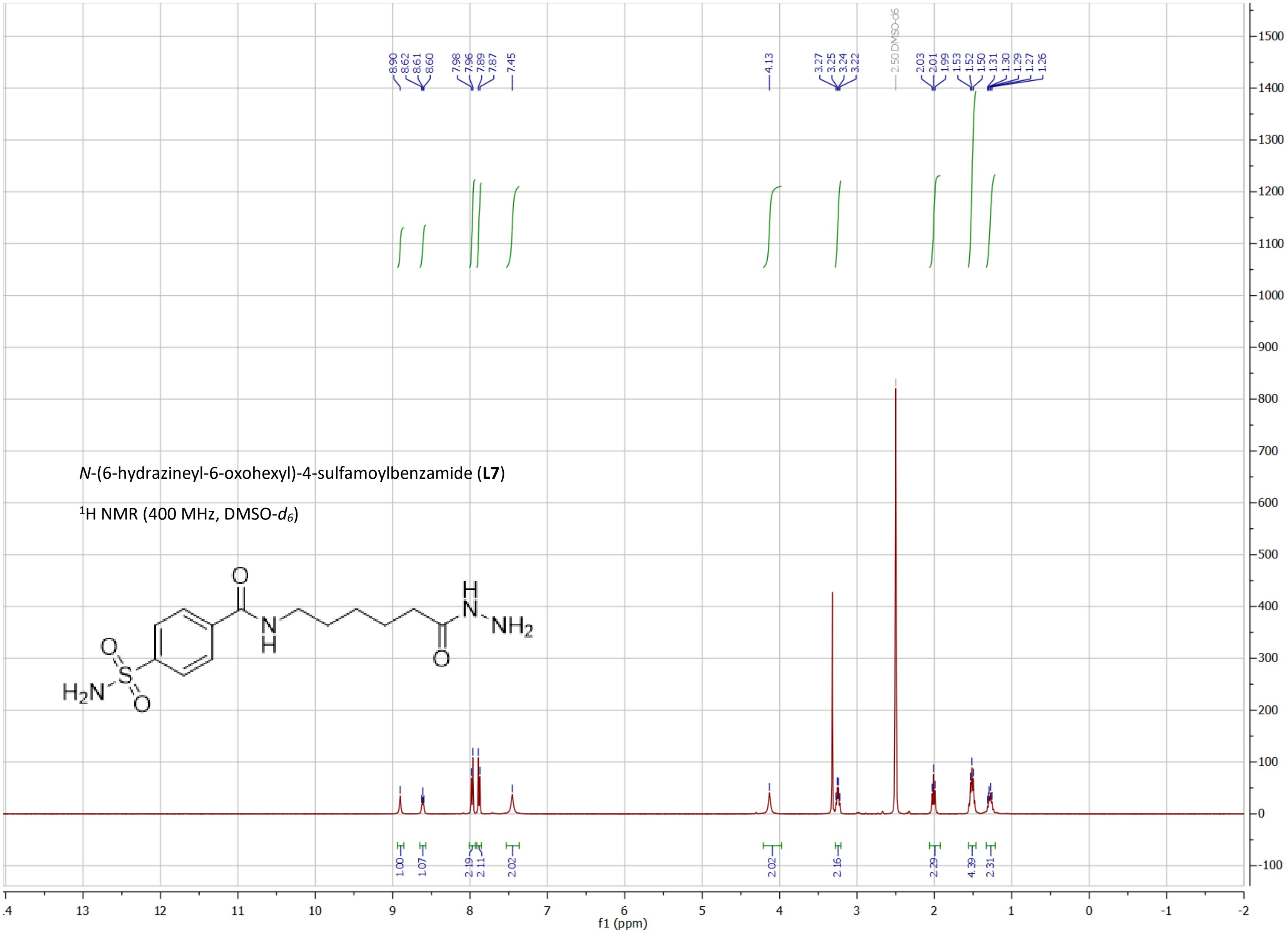
N-(6-hydrazineyl-6-oxohexyl)-4-sulfamoylbenzamide (L7)
1H NMR (400 MHz, DMSO-d6)
H2N
O
S
O
N
H
O
H
N
NH2
O
8.90
8.62
8.61
8.60
7.98
7.96
7.89
7.87
7.45
4.13
3.27
3.25
3.24
3.22
2.50 DMSO-d6
2.03
2.01
1.99
1.53
1.52
1.50
1.31
1.30
1.29
1.27
1.26
1.00
1.07
2.19
2.11
2.02
2.02
2.16
2.29
4.39
2.31
f1 (ppm)

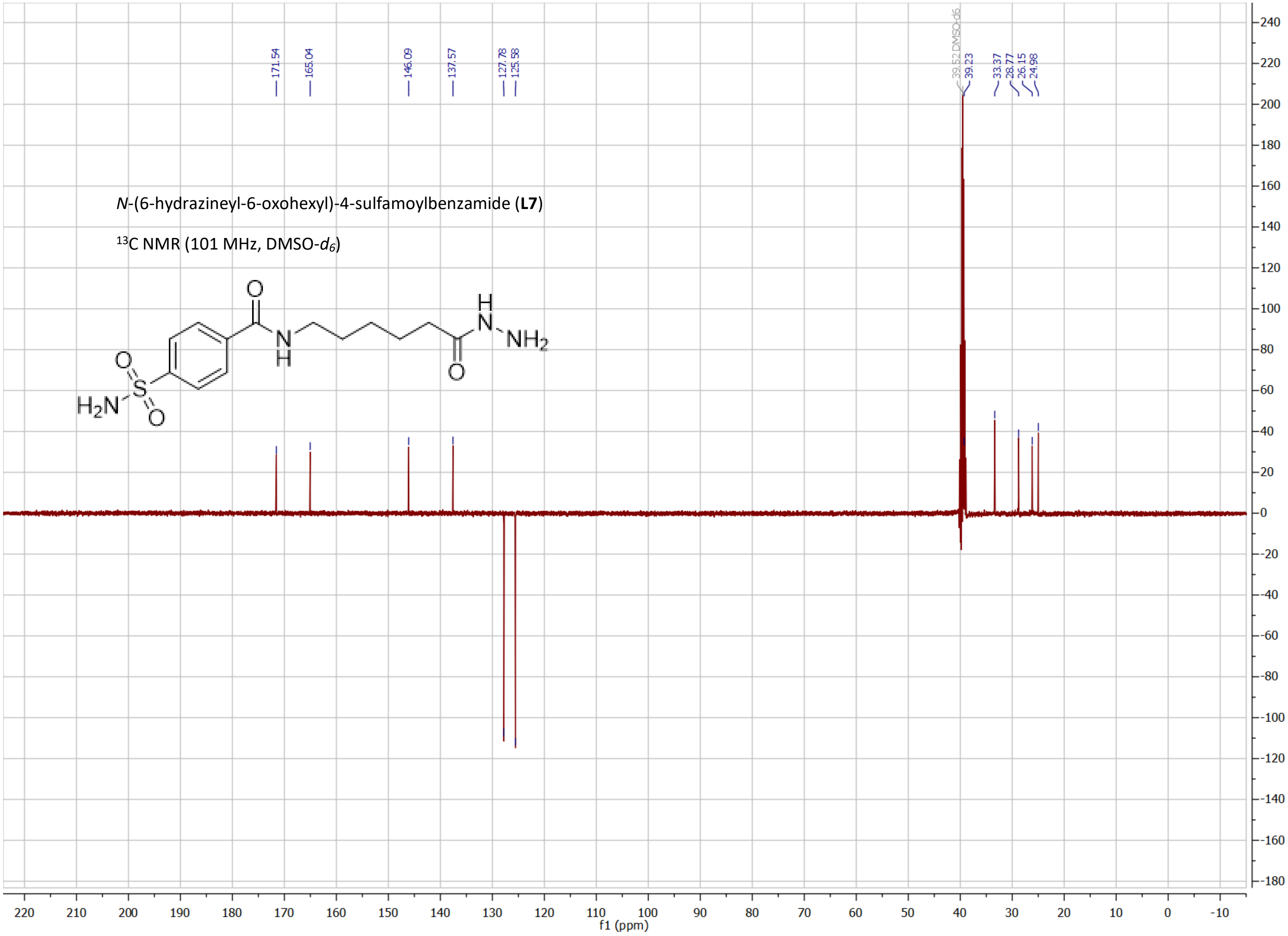
N-(6-hydrazineyl-6-oxohexyl)-4-sulfamoylbenzamide (L7)
13C NMR (101 MHz, DMSO-d6)
171.54
165.04
146.09
137.57
127.78
125.58
39.52 DMSO-d6
39.23
33.37
28.77
26.15
24.98
f1 (ppm)

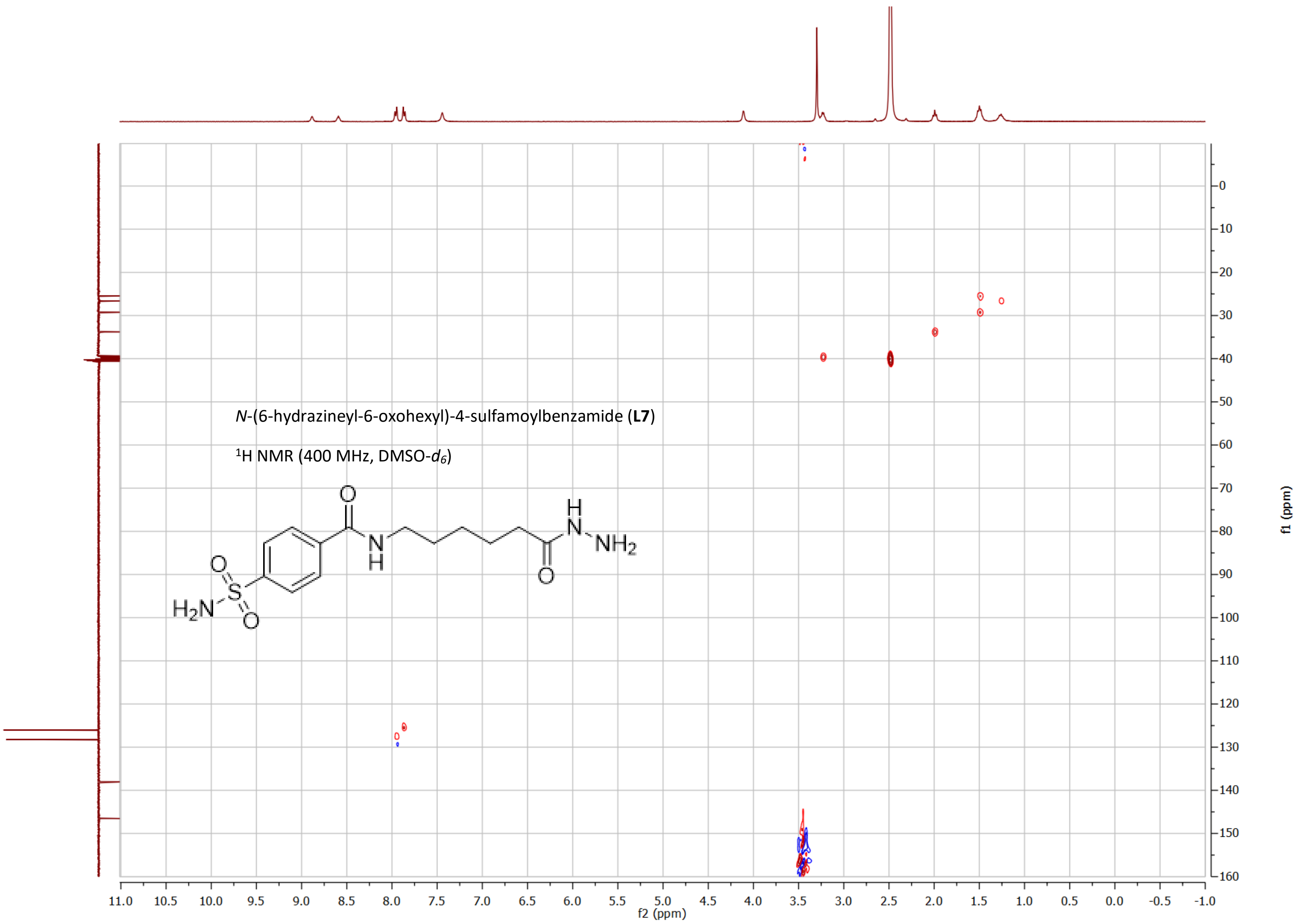
N-(6-hydrazineyl-6-oxohexyl)-4-sulfamoylbenzamide (L7)
1H NMR (400 MHz, DMSO-d6)
f2 (ppm)
f1 (ppm)

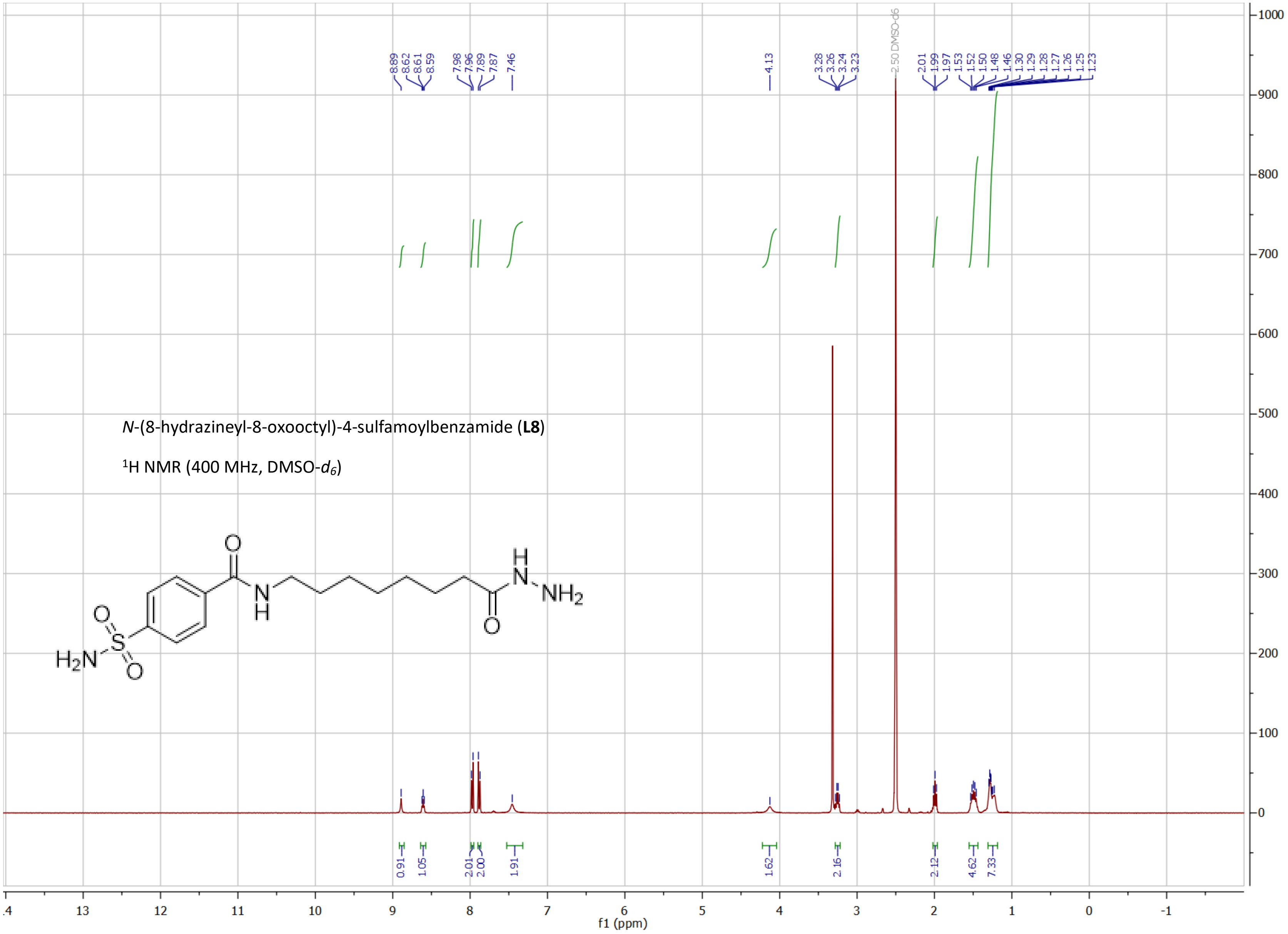
*N*-(8-hydrazineyl-8-oxooctyl)-4-sulfamoylbenzamide (**L8**)
$^1$H NMR (400 MHz, DMSO-$d_6$)
f1 (ppm)
2.50 DMSO-d6

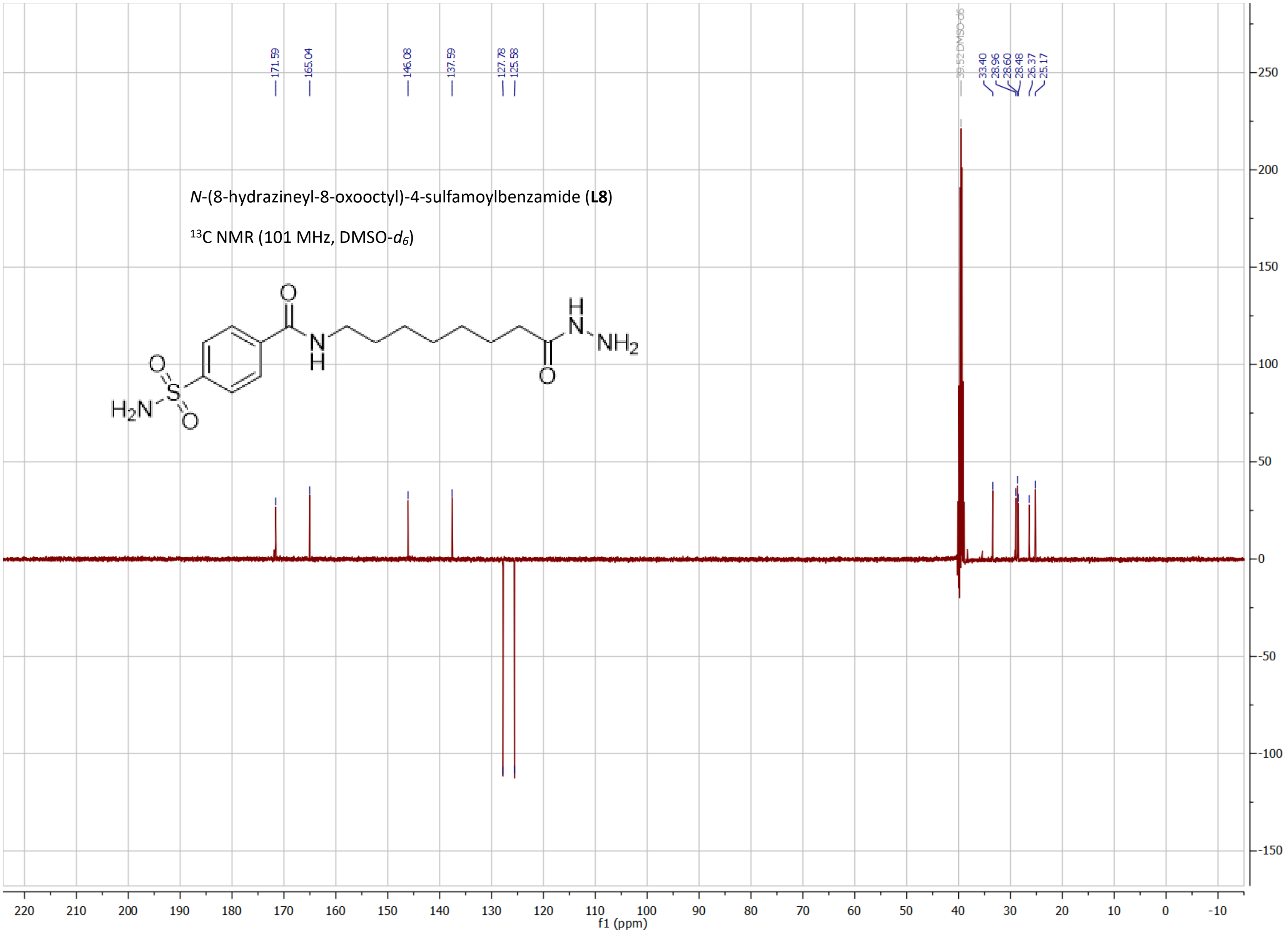
N-(8-hydrazineyl-8-oxooctyl)-4-sulfamoylbenzamide (L8)
13C NMR (101 MHz, DMSO-d6)
171.59
165.04
146.08
137.59
127.78
125.58
39.52 DMSO-d6
33.40
28.96
28.60
28.48
26.37
25.17
f1 (ppm)

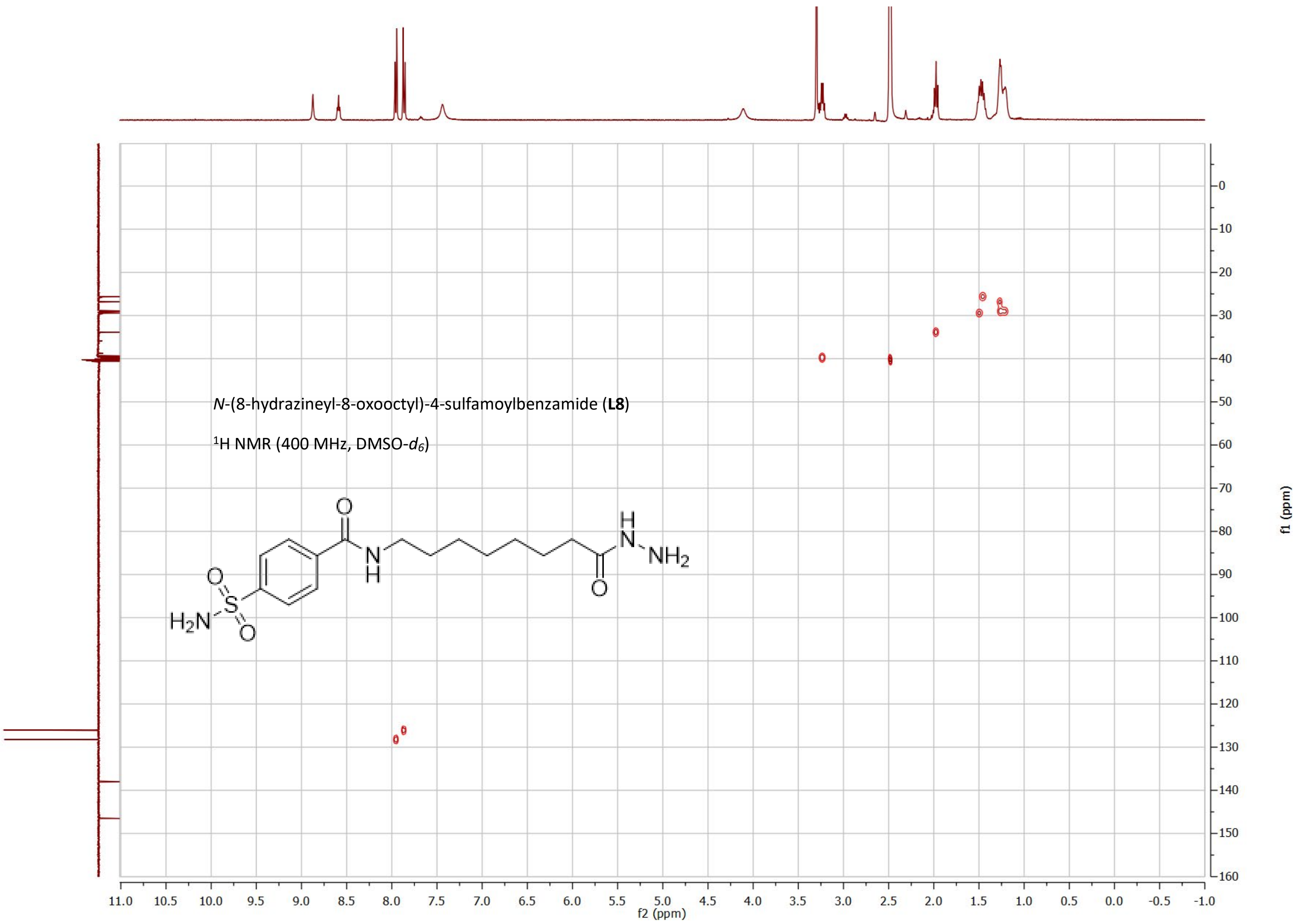

*N*-(8-hydrazineyl-8-oxooctyl)-4-sulfamoylbenzamide (**L8**)
$^{1}$H NMR (400 MHz, DMSO-$d_6$)
f2 (ppm)
f1 (ppm)

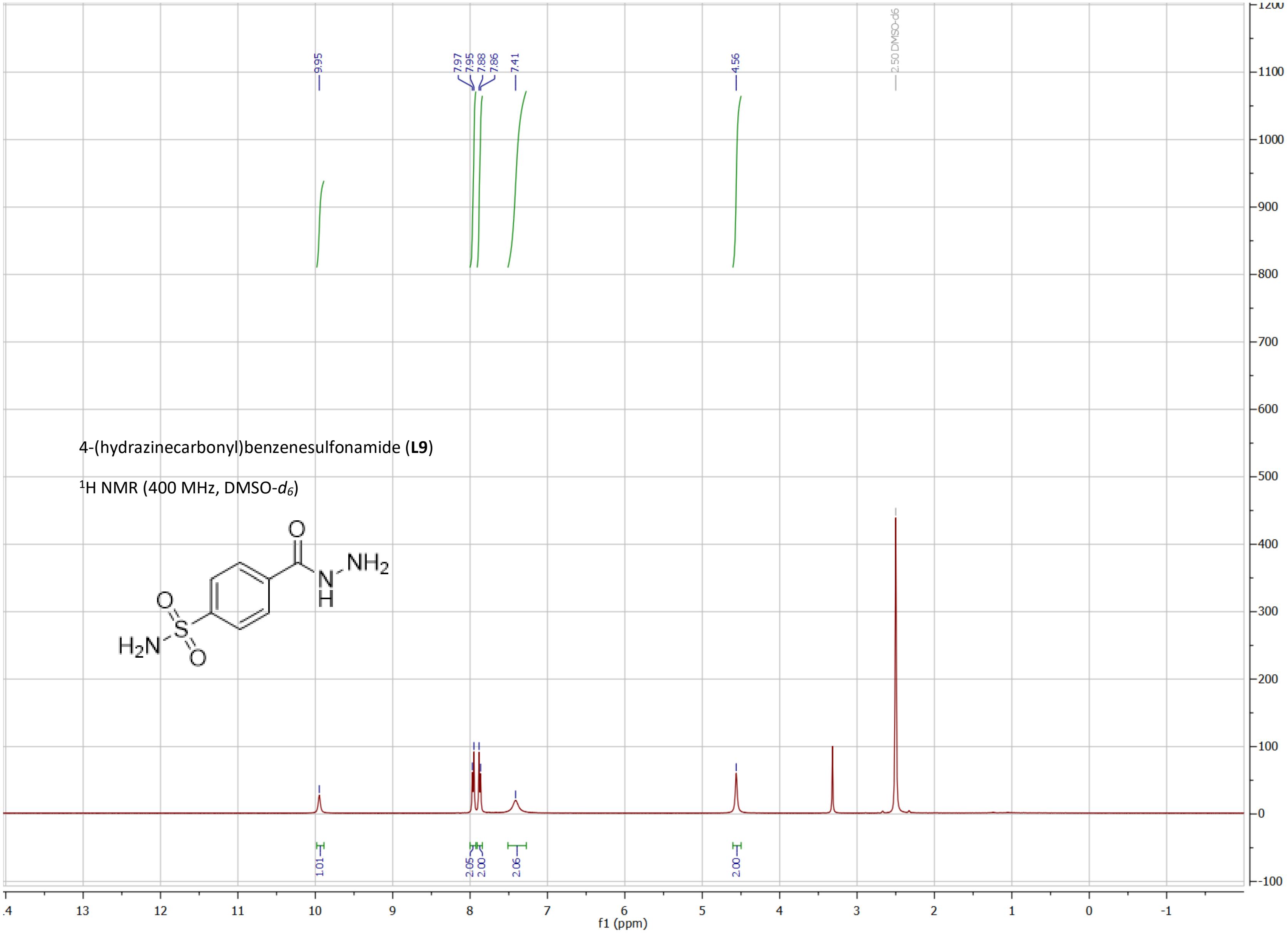
4-(hydrazinecarbonyl)benzenesulfonamide (**L9**)
$^{1}$H NMR (400 MHz, DMSO-$d_6$)
9.95
7.97
7.95
7.88
7.86
7.41
4.56
2.50 DMSO-d6
1.01
2.05
2.00
2.06
2.00
f1 (ppm)

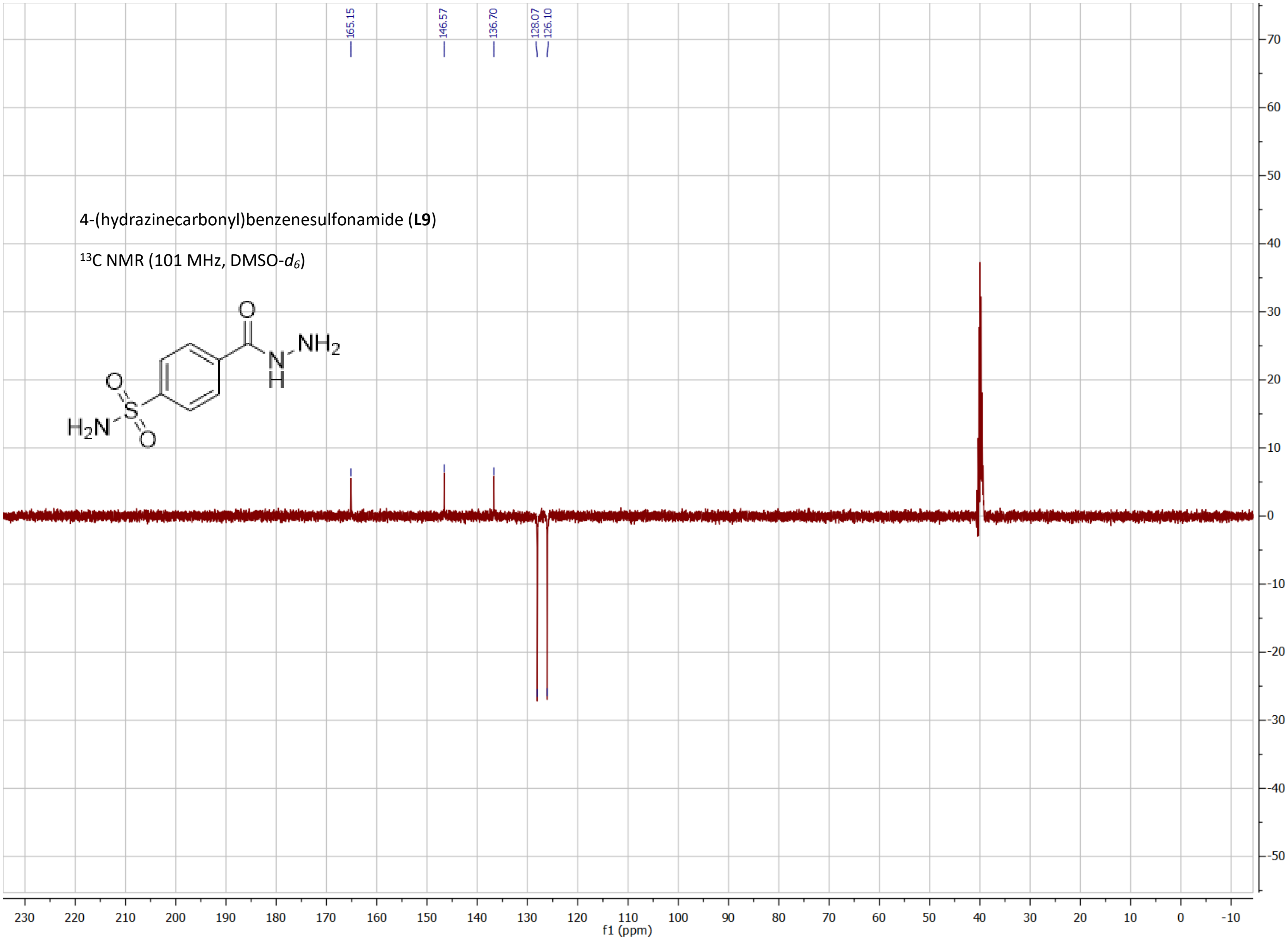
165.15
146.57
136.70
128.07
126.10
4-(hydrazinecarbonyl)benzenesulfonamide (**L9**)
$^{13}$C NMR (101 MHz, DMSO-$d_6$)
O
NH2
N
H
O
S
H2N
O
70
60
50
40
30
20
10
0
-10
-20
-30
-40
-50
230
220
210
200
190
180
170
160
150
140
130
120
110
100
90
80
70
60
50
40
30
20
10
0
-10
f1 (ppm)

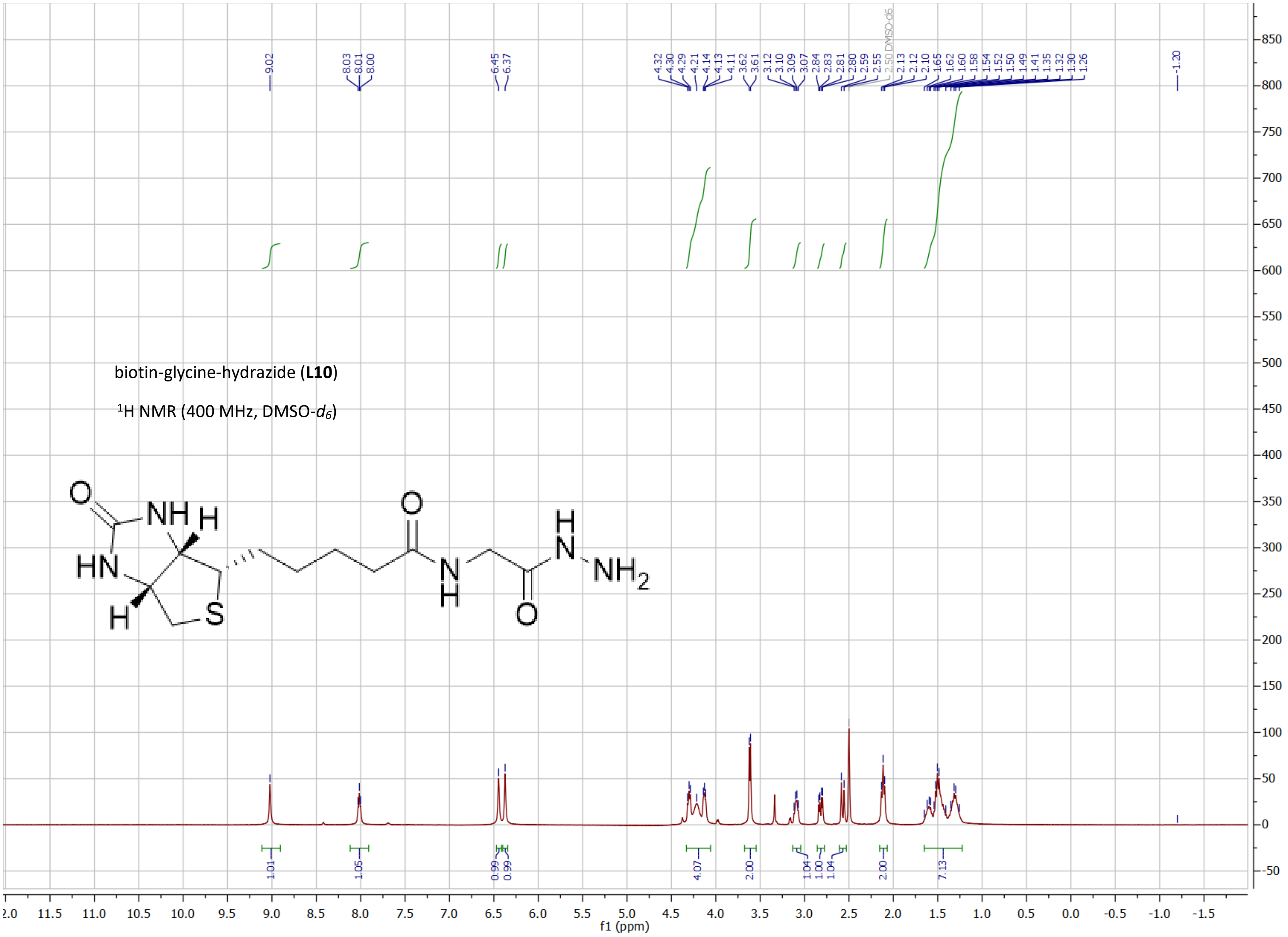
biotin-glycine-hydrazide (**L10**)
$^{1}$H NMR (400 MHz, DMSO-$d_6$)
f1 (ppm)

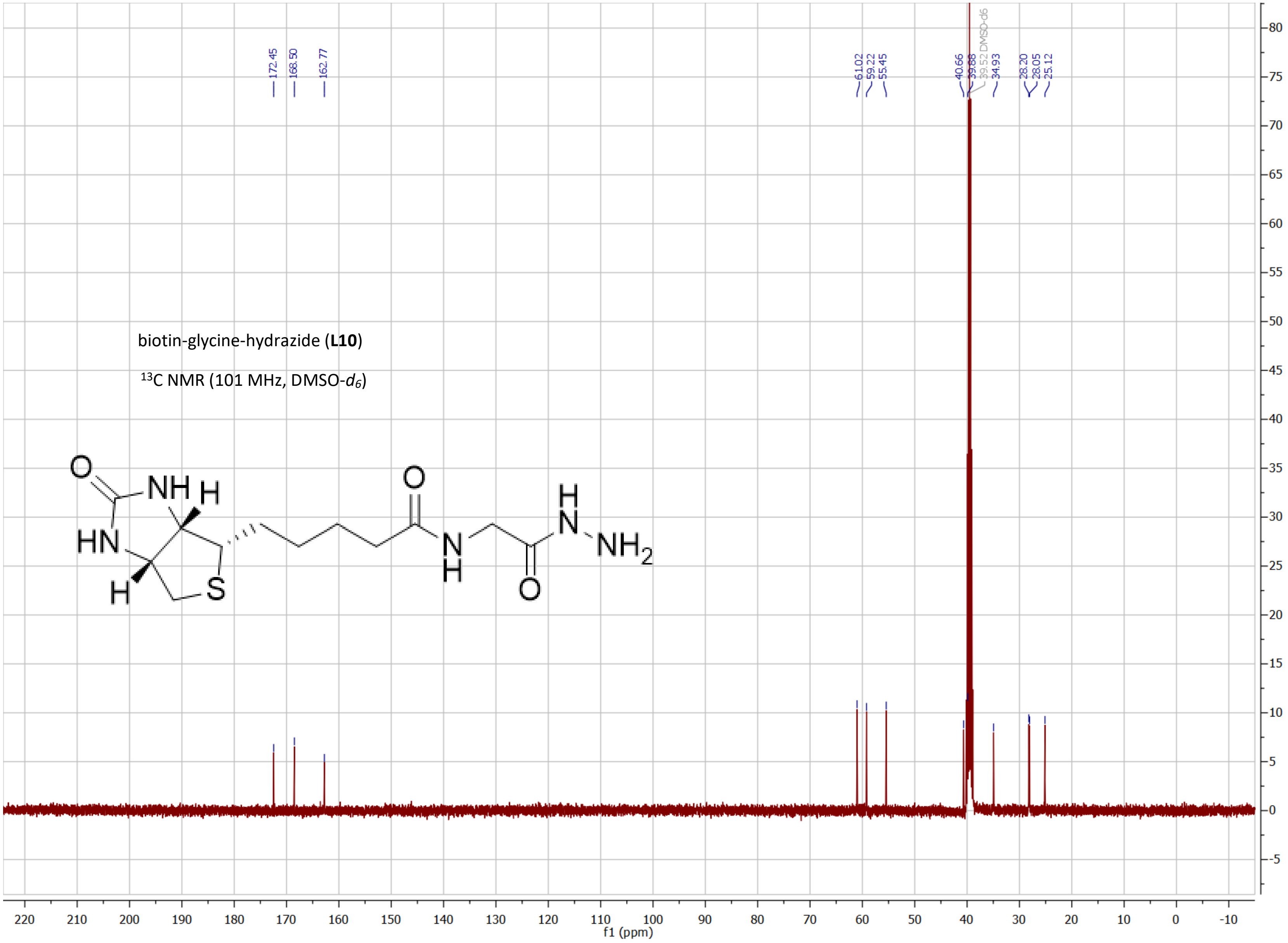
172.45
168.50
162.77
61.02
59.22
55.45
40.66
39.88
39.52 DMSO-d6
34.93
28.20
28.05
25.12
biotin-glycine-hydrazide (**L10**)
$^{13}$C NMR (101 MHz, DMSO-$d_6$)
f1 (ppm)

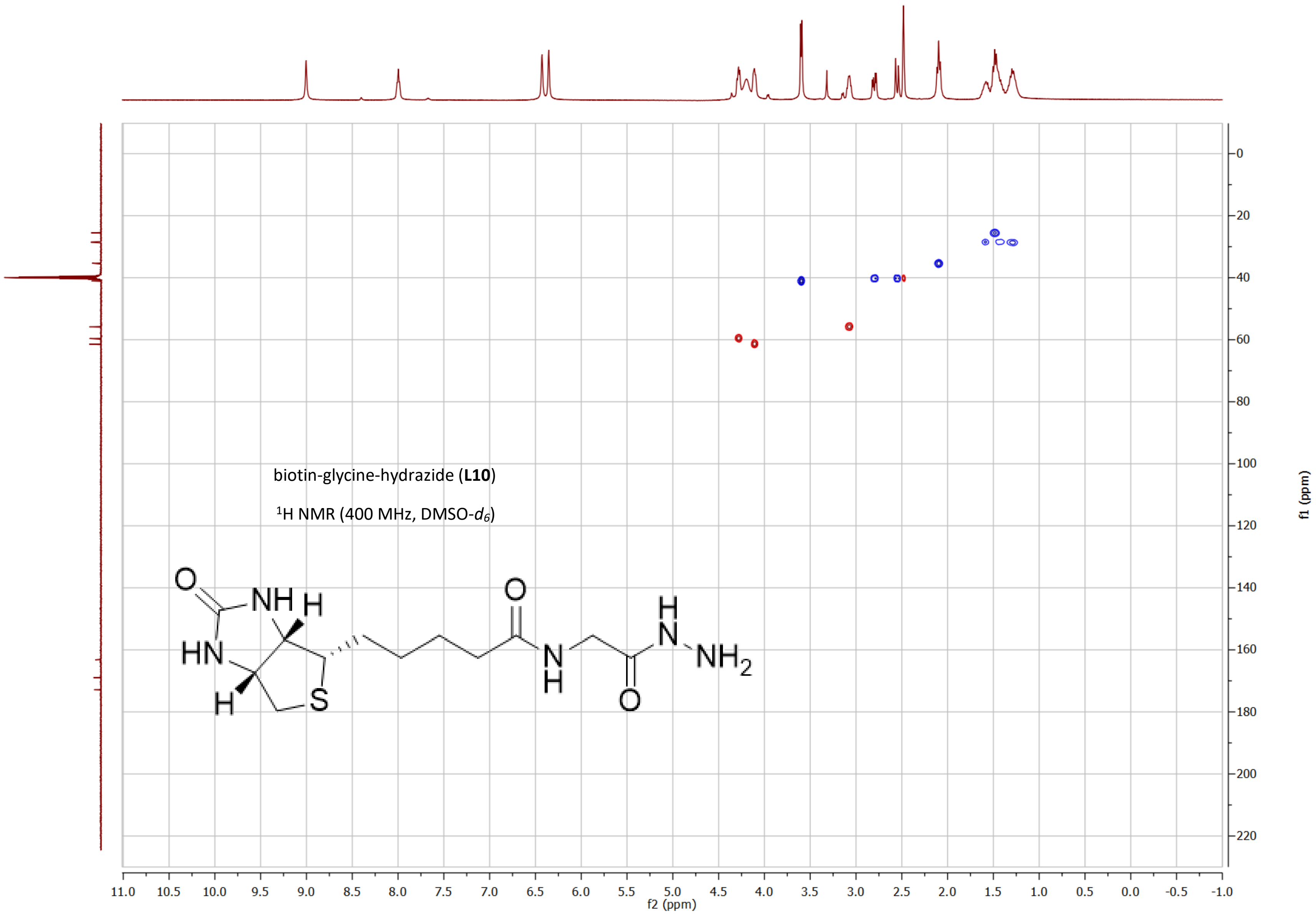

biotin-glycine-hydrazide (**L10**)
$^{1}$H NMR (400 MHz, DMSO-$d_6$)
f2 (ppm)
f1 (ppm)

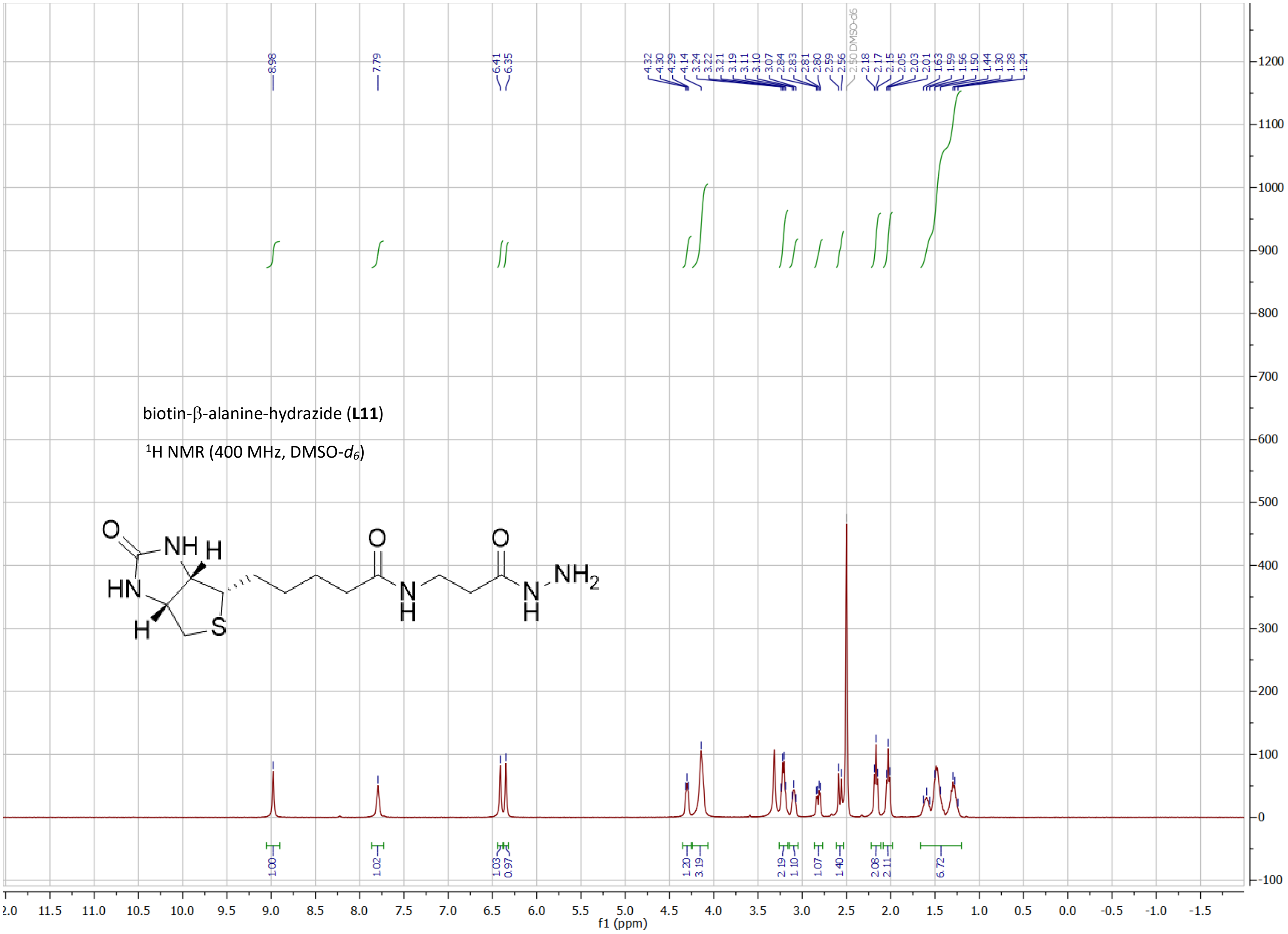
biotin-β-alanine-hydrazide (**L11**)
$^{1}$H NMR (400 MHz, DMSO-$d_6$)
8.98
7.79
6.41
6.35
4.32
4.30
4.29
4.14
3.24
3.22
3.21
3.19
3.11
3.10
3.07
2.84
2.83
2.81
2.80
2.59
2.56
2.50 DMSO-d6
2.18
2.17
2.15
2.05
2.03
2.01
1.63
1.59
1.56
1.50
1.44
1.30
1.28
1.24
1.00
1.02
1.03
0.97
1.20
3.19
2.19
1.10
1.07
1.40
2.08
2.11
6.72
f1 (ppm)

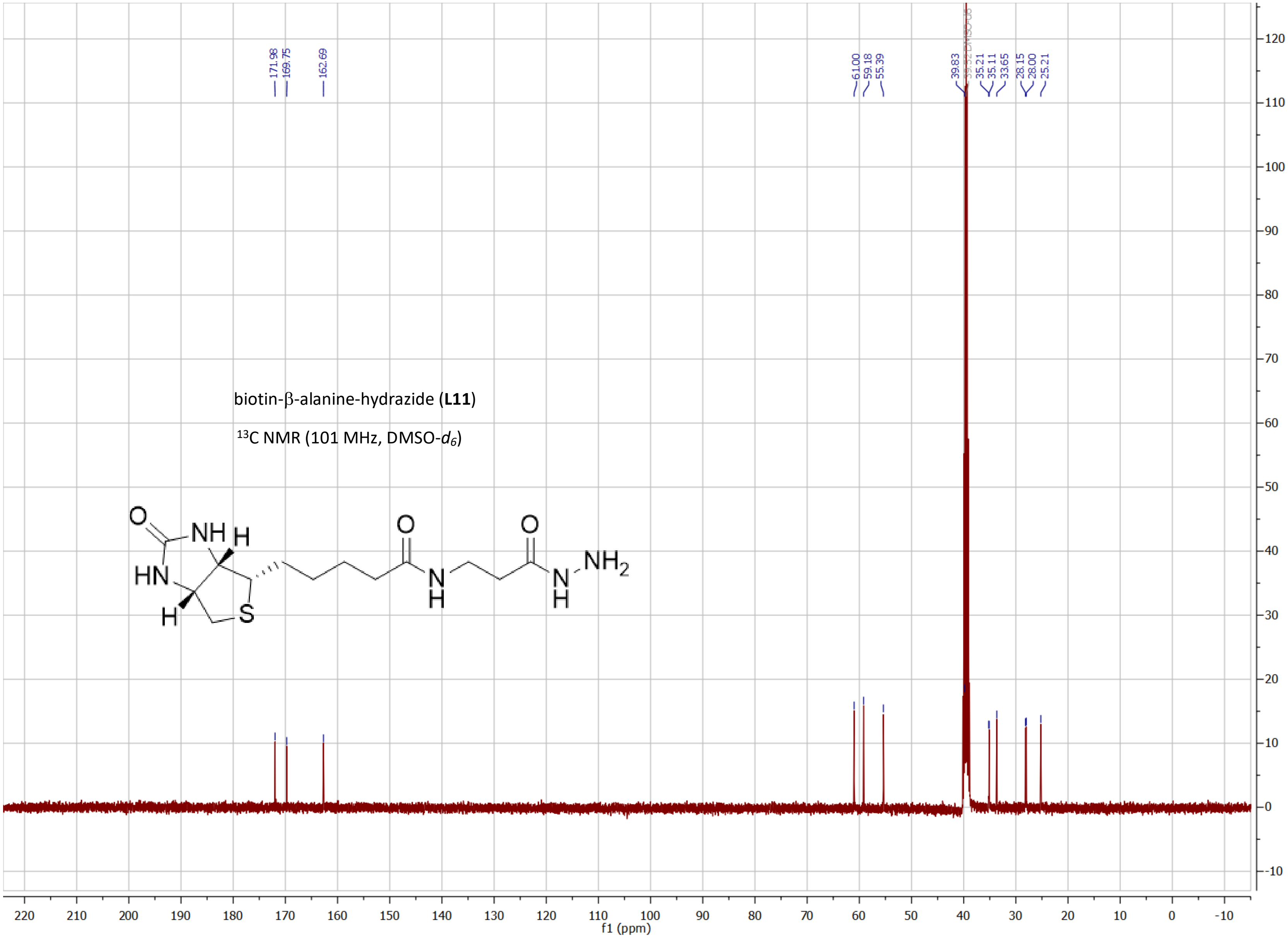
biotin-β-alanine-hydrazide (L11)
13C NMR (101 MHz, DMSO-d6)
171.98
169.75
162.69
61.00
59.18
55.39
39.83
35.21
35.11
33.65
28.15
28.00
25.21
f1 (ppm)

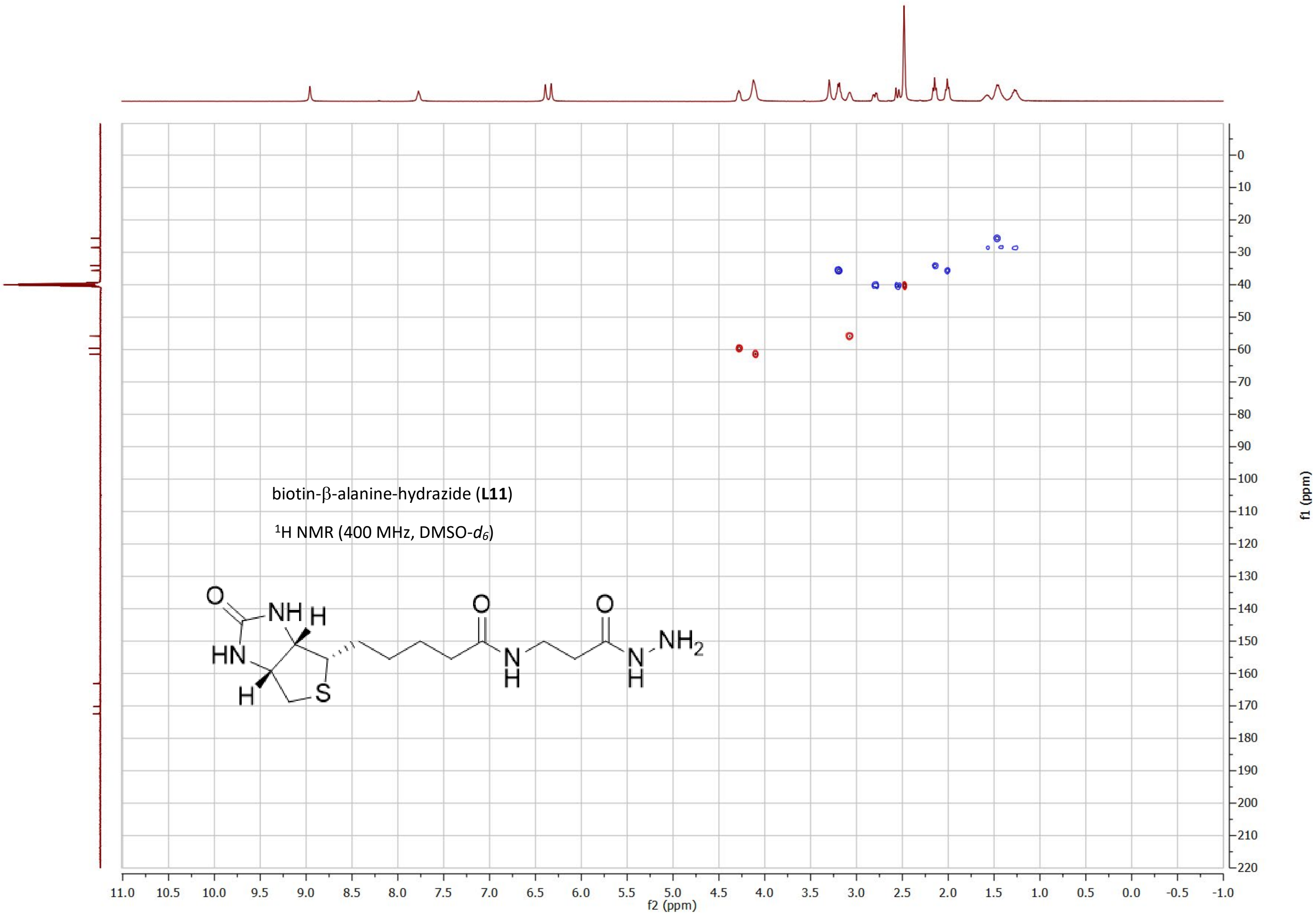
biotin-β-alanine-hydrazide (**L11**)
$^{1}$H NMR (400 MHz, DMSO-$d_6$)
f2 (ppm)
f1 (ppm)
11.0 10.5 10.0 9.5 9.0 8.5 8.0 7.5 7.0 6.5 6.0 5.5 5.0 4.5 4.0 3.5 3.0 2.5 2.0 1.5 1.0 0.5 0.0 -0.5 -1.0
0 10 20 30 40 50 60 70 80 90 100 110 120 130 140 150 160 170 180 190 200 210 220

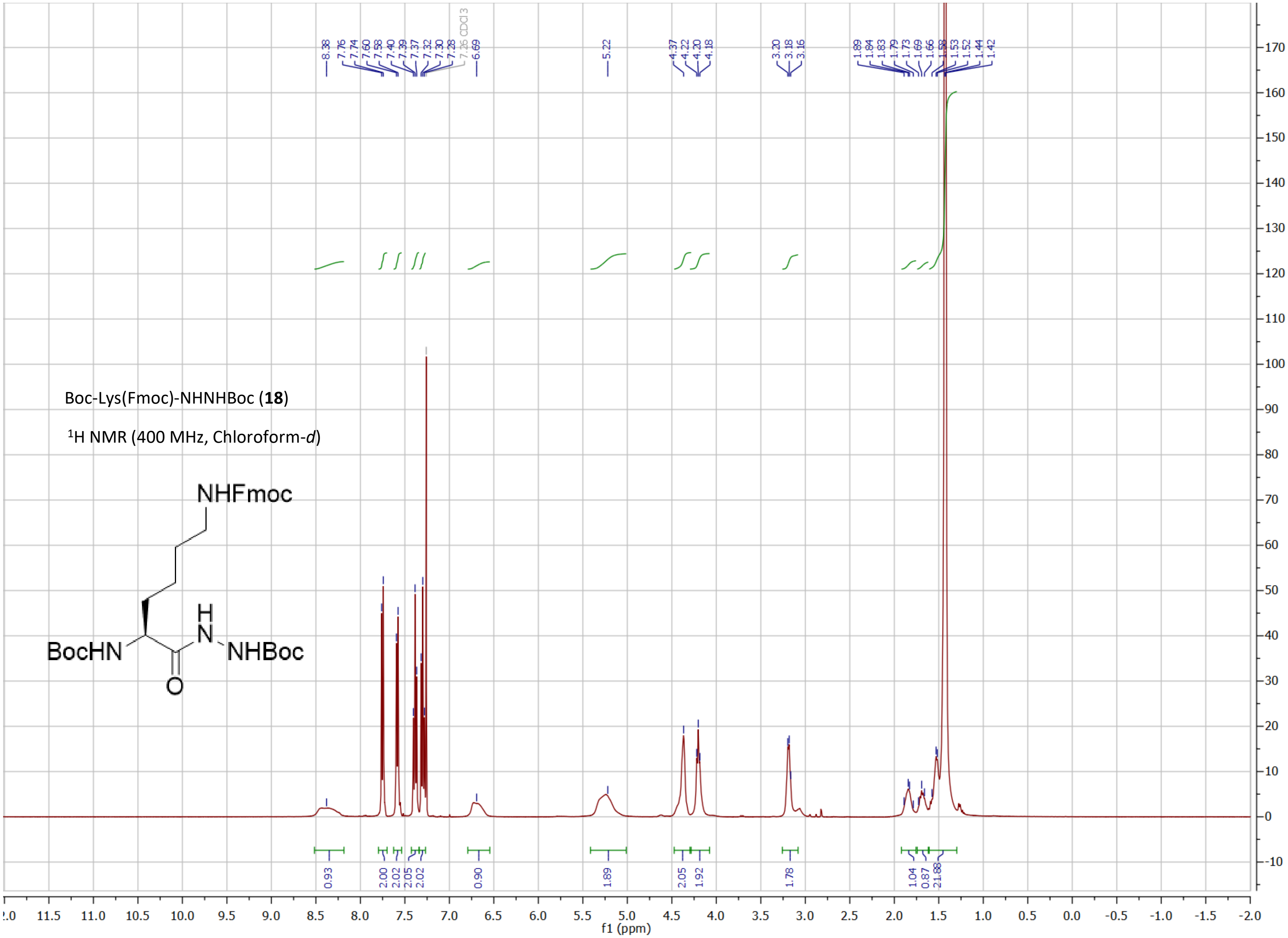

Boc-Lys(Fmoc)-NHNHBoc (**18**)
$^{1}$H NMR (400 MHz, Chloroform-*d*)
NHFmoc
BocHN
H
N
NHBoc
O
8.38
7.76
7.74
7.60
7.58
7.40
7.39
7.37
7.32
7.30
7.28
7.26 CDCl3
6.69
5.22
4.37
4.22
4.20
4.18
3.20
3.18
3.16
1.89
1.84
1.83
1.79
1.73
1.69
1.66
1.58
1.53
1.52
1.44
1.42
0.93
2.00
2.02
2.05
2.02
0.90
1.89
2.05
1.92
1.78
1.04
0.87
21.88
f1 (ppm)

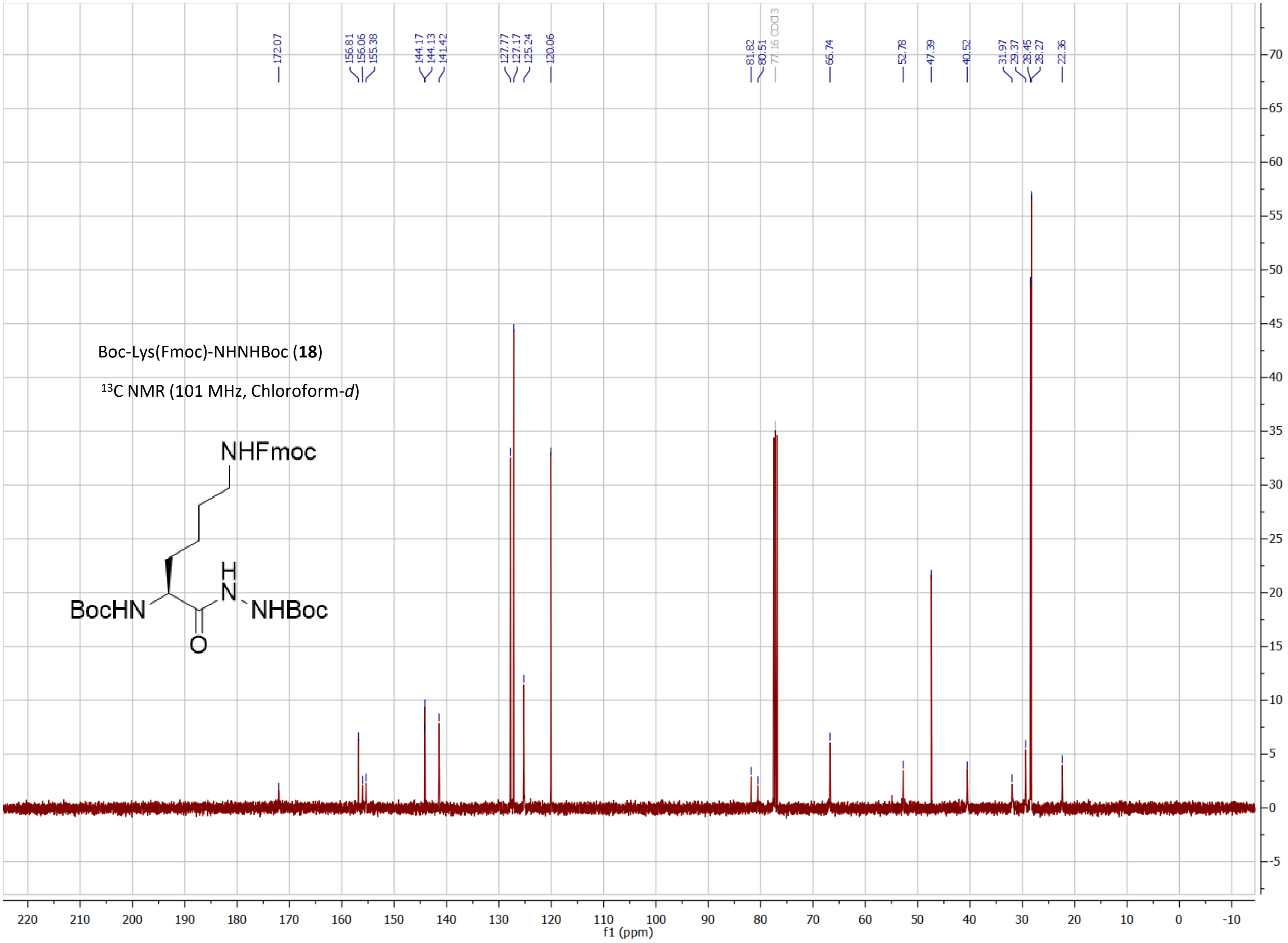
Boc-Lys(Fmoc)-NHNHBoc (**18**)
$^{13}$C NMR (101 MHz, Chloroform-*d*)
NHFmoc
BocHN
H
N
NHBoc
O
172.07
156.81
156.06
155.38
144.17
144.13
141.42
127.77
127.17
125.24
120.06
81.82
80.51
77.16 CDCl3
66.74
52.78
47.39
40.52
31.97
29.37
28.45
28.27
22.36
f1 (ppm)

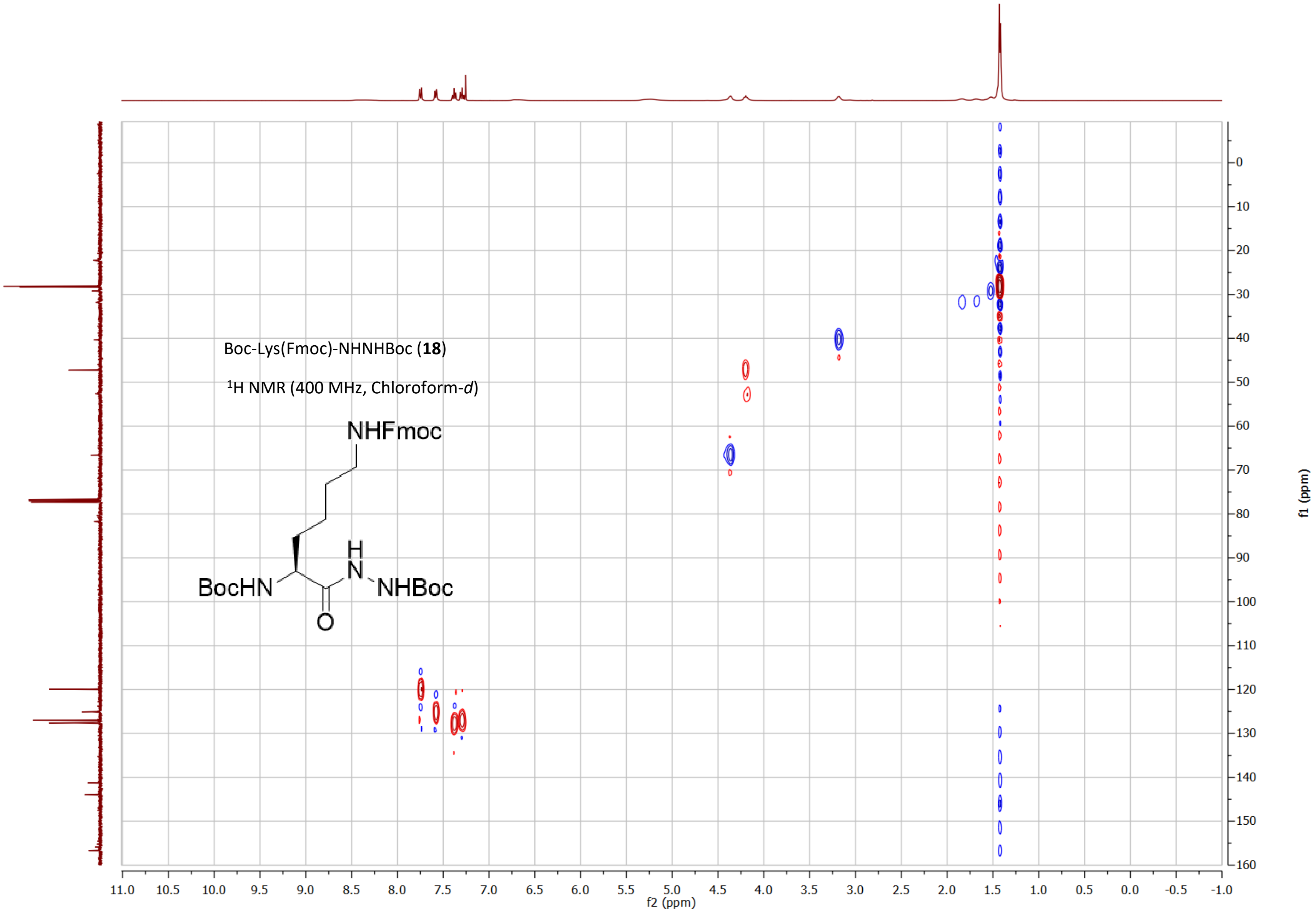

Boc-Lys(Fmoc)-NHNHBoc (**18**)
$^{1}$H NMR (400 MHz, Chloroform-*d*)
NHFmoc
BocHN
H
N
NHBoc
O
f2 (ppm)
f1 (ppm)

Boc-Lys-NHNHBoc (**18**)

$^{1}$H NMR (400 MHz, methanol-$d_4$)

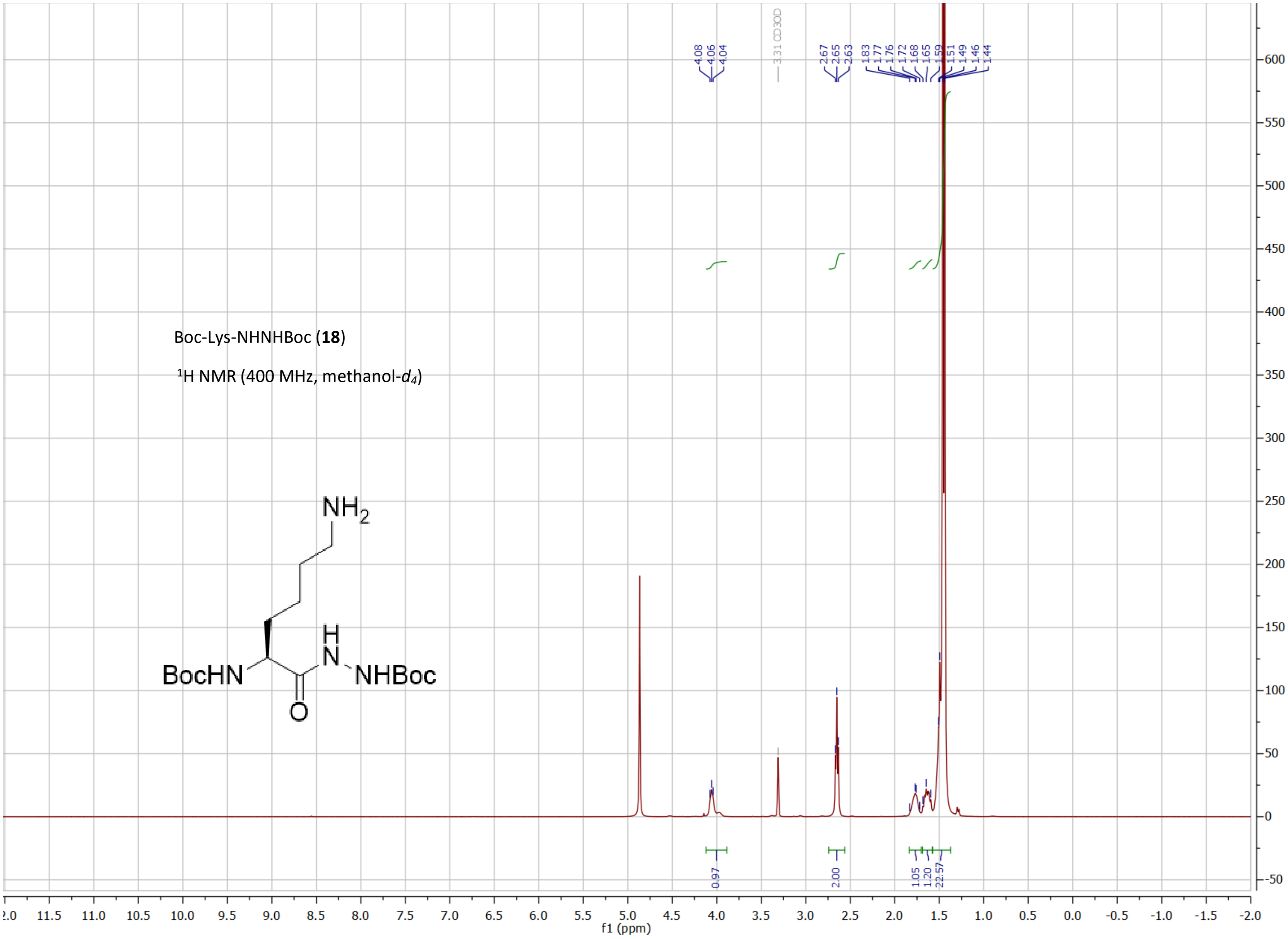

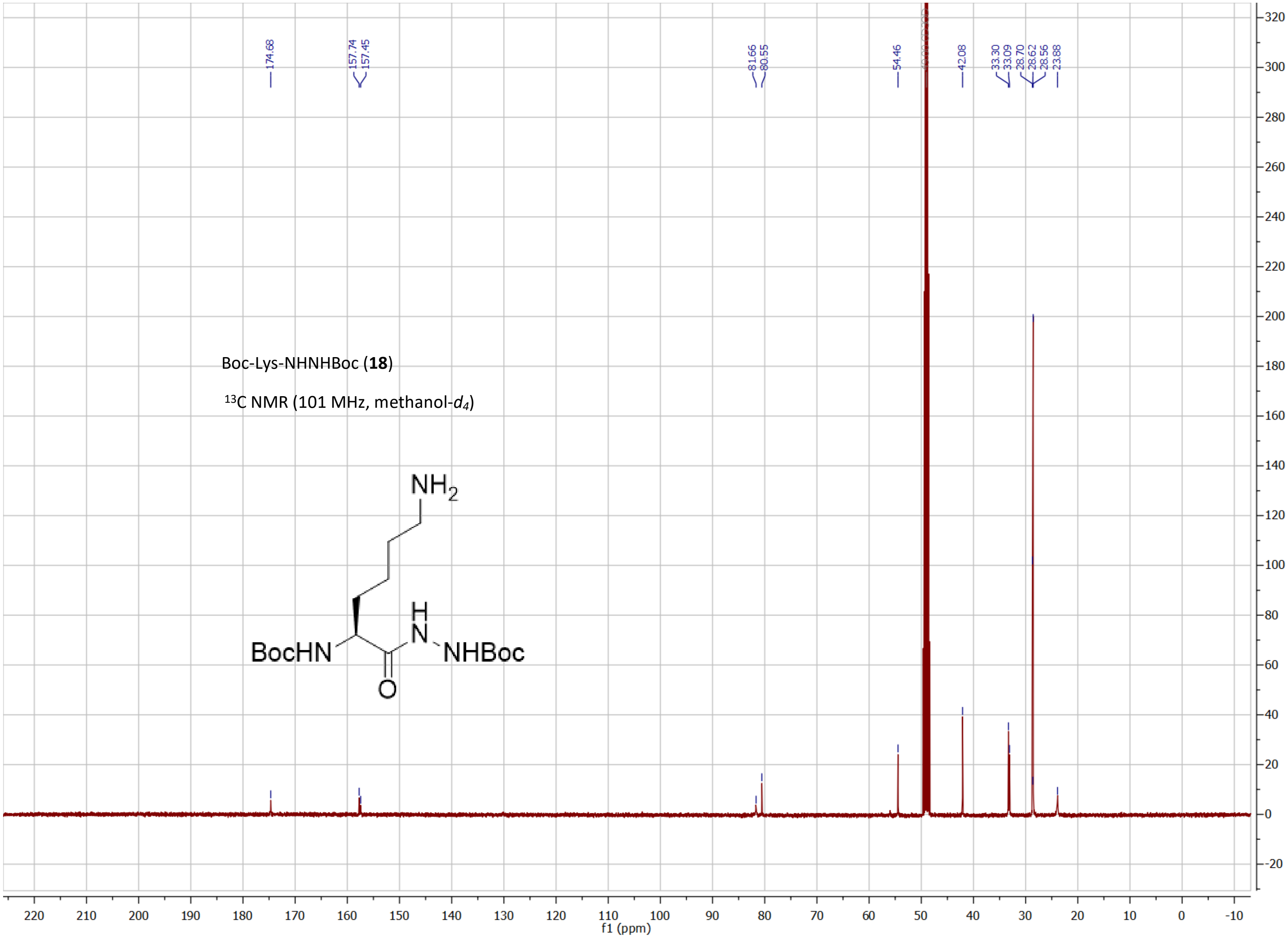
Boc-Lys-NHNHBoc (18)
13C NMR (101 MHz, methanol-d4)
174.68
157.74
157.45
81.66
80.55
54.46
42.08
33.30
33.09
28.70
28.62
28.56
23.88
NH2
BocHN
NHBoc
H
N
O
f1 (ppm)

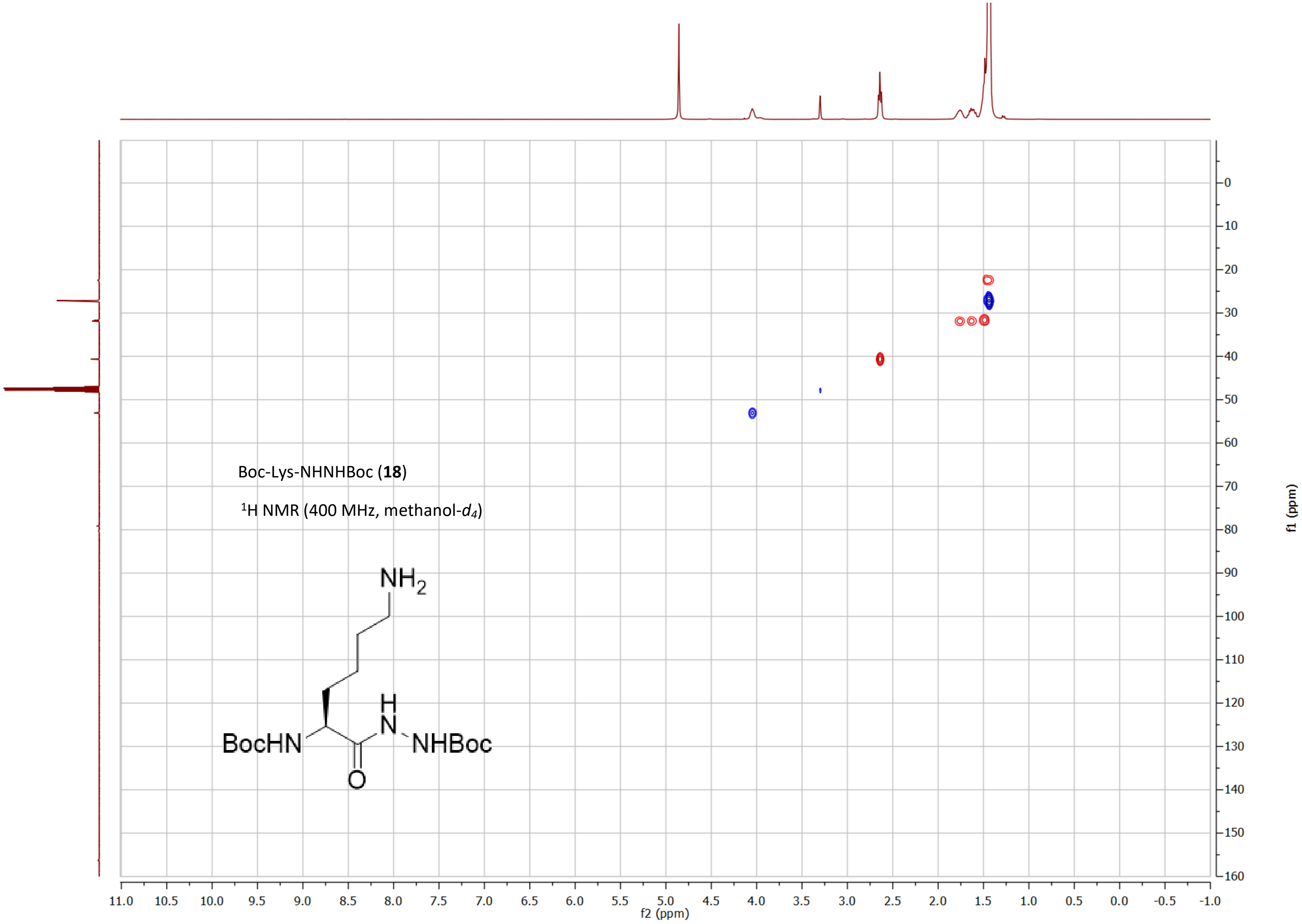
Boc-Lys-NHNHBoc (**18**)
1H NMR (400 MHz, methanol-$d_4$)
NH2
H
N
BocHN
NHBoc
O
f1 (ppm)
f2 (ppm)

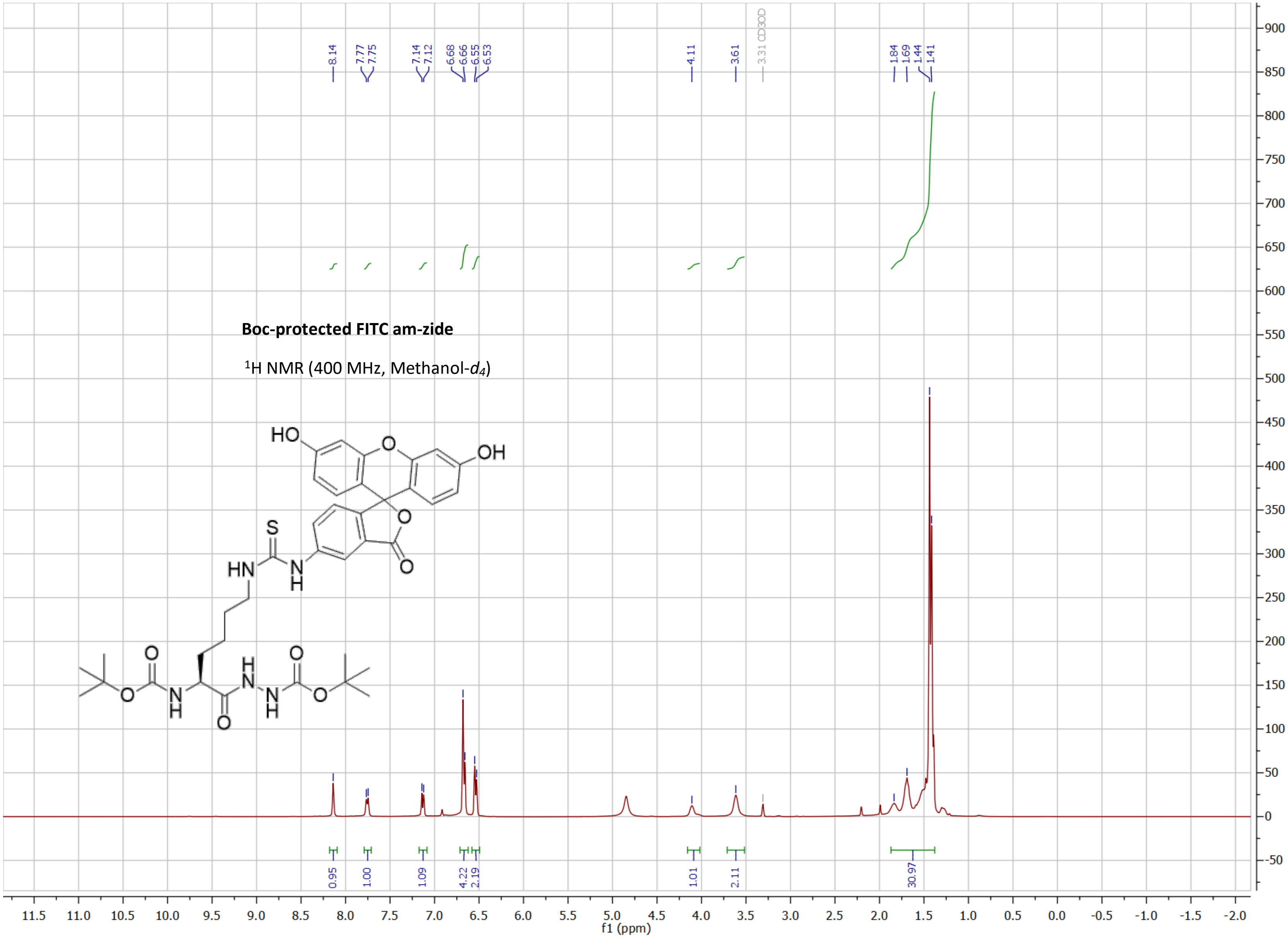

Boc-protected FITC am-zide
$^{1}$H NMR (400 MHz, Methanol-$d_4$)
8.14
7.77
7.75
7.14
7.12
6.68
6.66
6.55
6.53
4.11
3.61
3.31 CD3OD
1.84
1.69
1.44
1.41
0.95
1.00
1.09
4.22
2.19
1.01
2.11
30.97
f1 (ppm)

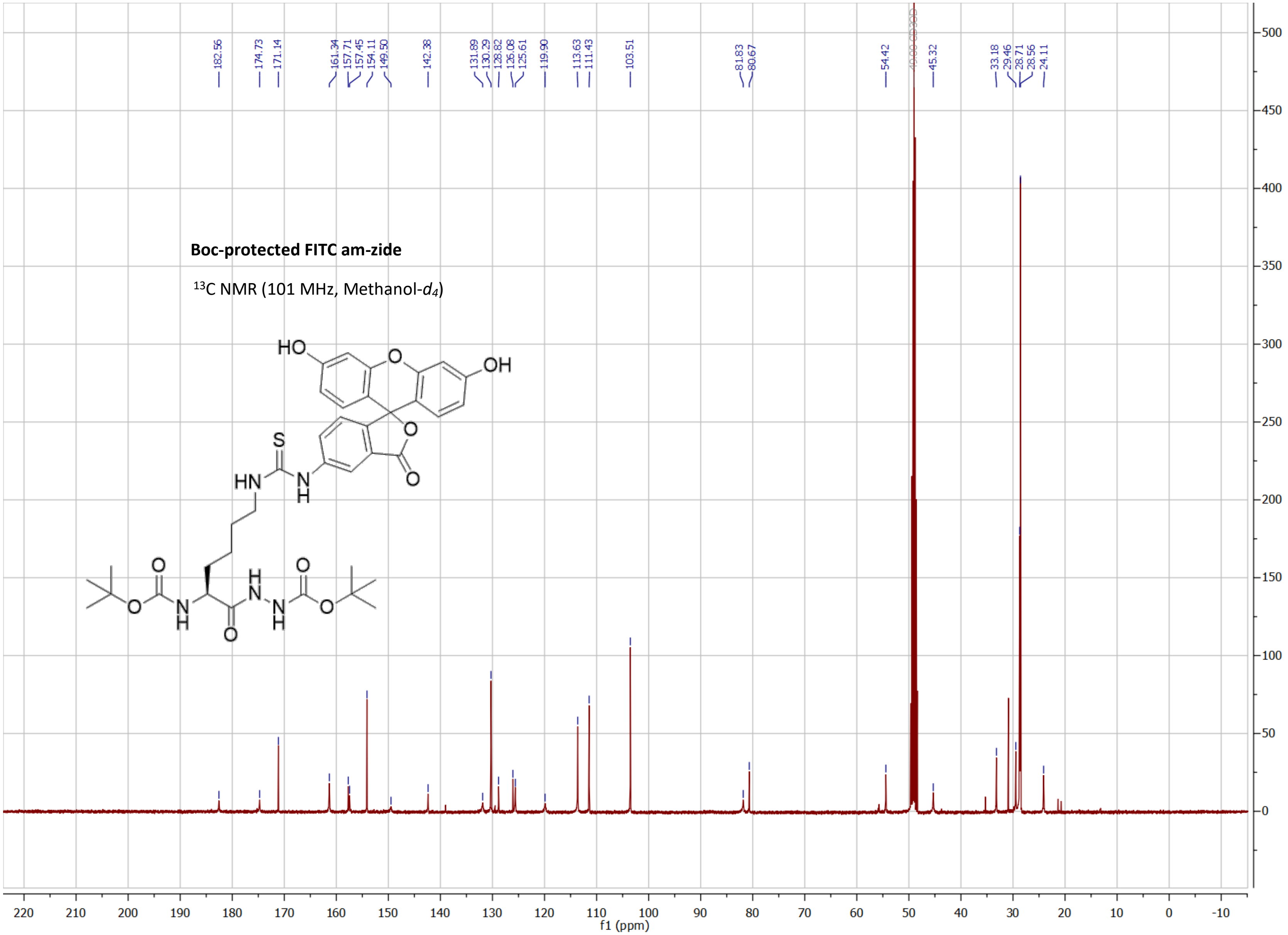
Boc-protected FITC am-zide
13C NMR (101 MHz, Methanol-d4)
182.56
174.73
171.14
161.34
157.71
157.45
154.11
149.50
142.38
131.89
130.29
128.82
126.08
125.61
119.90
113.63
111.43
103.51
81.83
80.67
54.42
45.32
33.18
29.46
28.71
28.56
24.11
f1 (ppm)

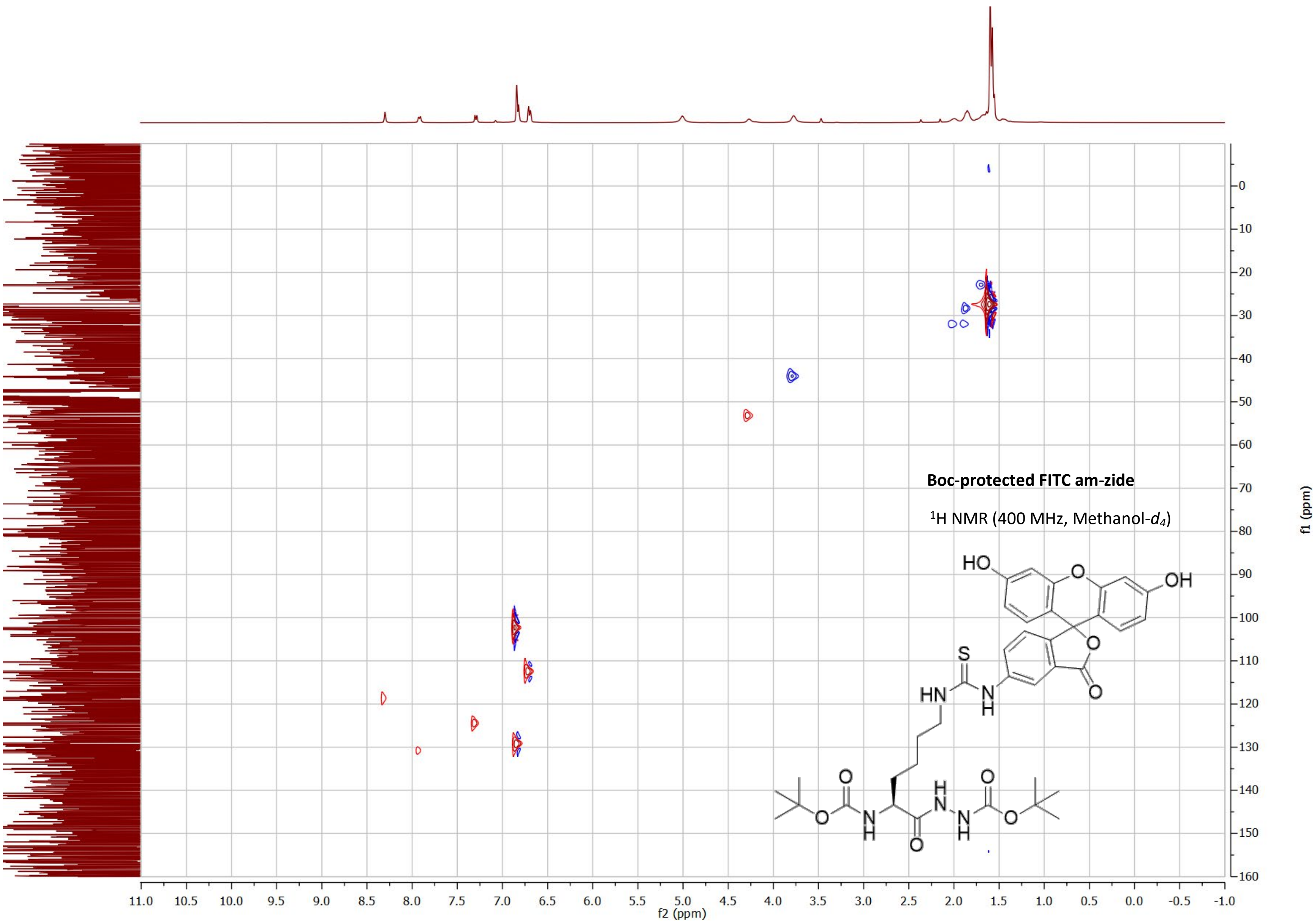
Boc-protected FITC am-zide
$^1$H NMR (400 MHz, Methanol-$d_4$)
HO
O
OH
S
O
O
HN
N H
O
H N
N H
O
O
O
N H
O
f1 (ppm)
f2 (ppm)
11.0 10.5 10.0 9.5 9.0 8.5 8.0 7.5 7.0 6.5 6.0 5.5 5.0 4.5 4.0 3.5 3.0 2.5 2.0 1.5 1.0 0.5 0.0 -0.5 -1.0
0 10 20 30 40 50 60 70 80 90 100 110 120 130 140 150 160

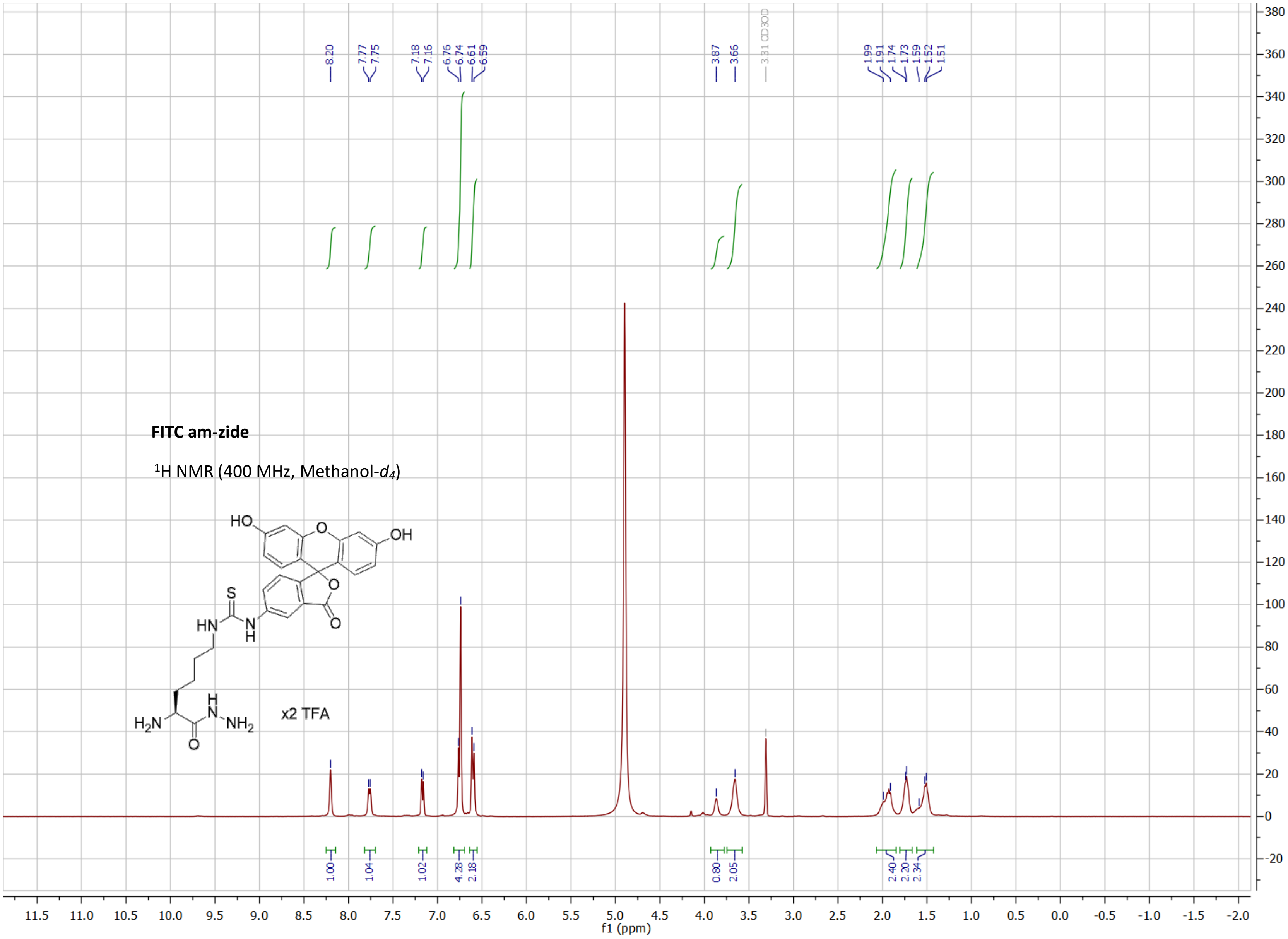
FITC am-zide
$^{1}$H NMR (400 MHz, Methanol-$d_4$)
x2 TFA
8.20
7.77
7.75
7.18
7.16
6.76
6.74
6.61
6.59
3.87
3.66
3.31 CD3OD
1.99
1.91
1.74
1.73
1.59
1.52
1.51
1.00
1.04
1.02
4.28
2.18
0.80
2.05
2.40
2.20
2.34
f1 (ppm)

**FITC am-zide**

$^{13}$C NMR (151 MHz, Methanol-$d_4$)

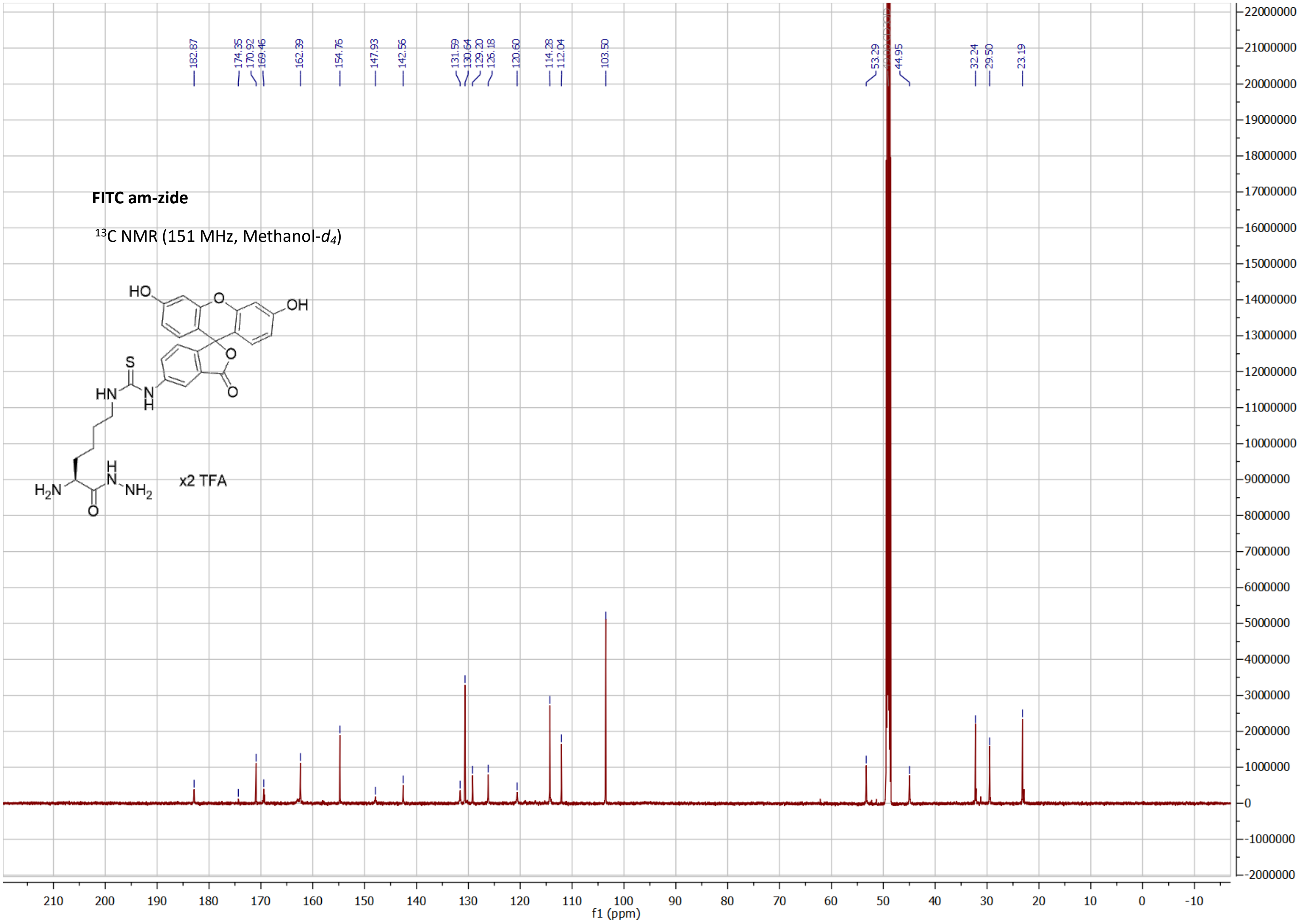

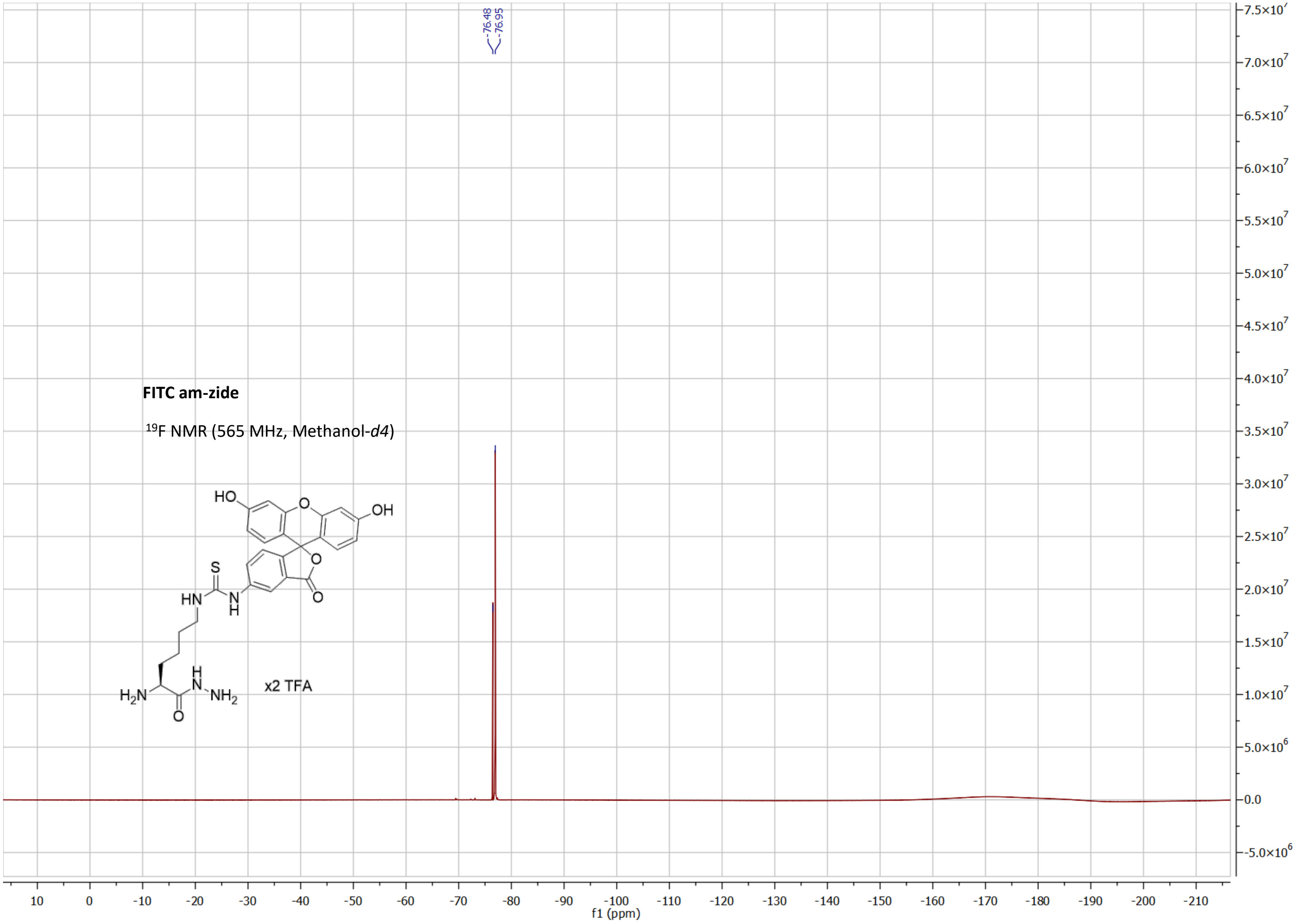

-76.48
-76.95
FITC am-zide
19F NMR (565 MHz, Methanol-d4)
HO
O
OH
S
O
HN
N
H
O
H
N
H2N
NH2
O
x2 TFA
7.5×10^7
7.0×10^7
6.5×10^7
6.0×10^7
5.5×10^7
5.0×10^7
4.5×10^7
4.0×10^7
3.5×10^7
3.0×10^7
2.5×10^7
2.0×10^7
1.5×10^7
1.0×10^7
5.0×10^6
0.0
-5.0×10^6
10 0 -10 -20 -30 -40 -50 -60 -70 -80 -90 -100 -110 -120 -130 -140 -150 -160 -170 -180 -190 -200 -210
f1 (ppm)

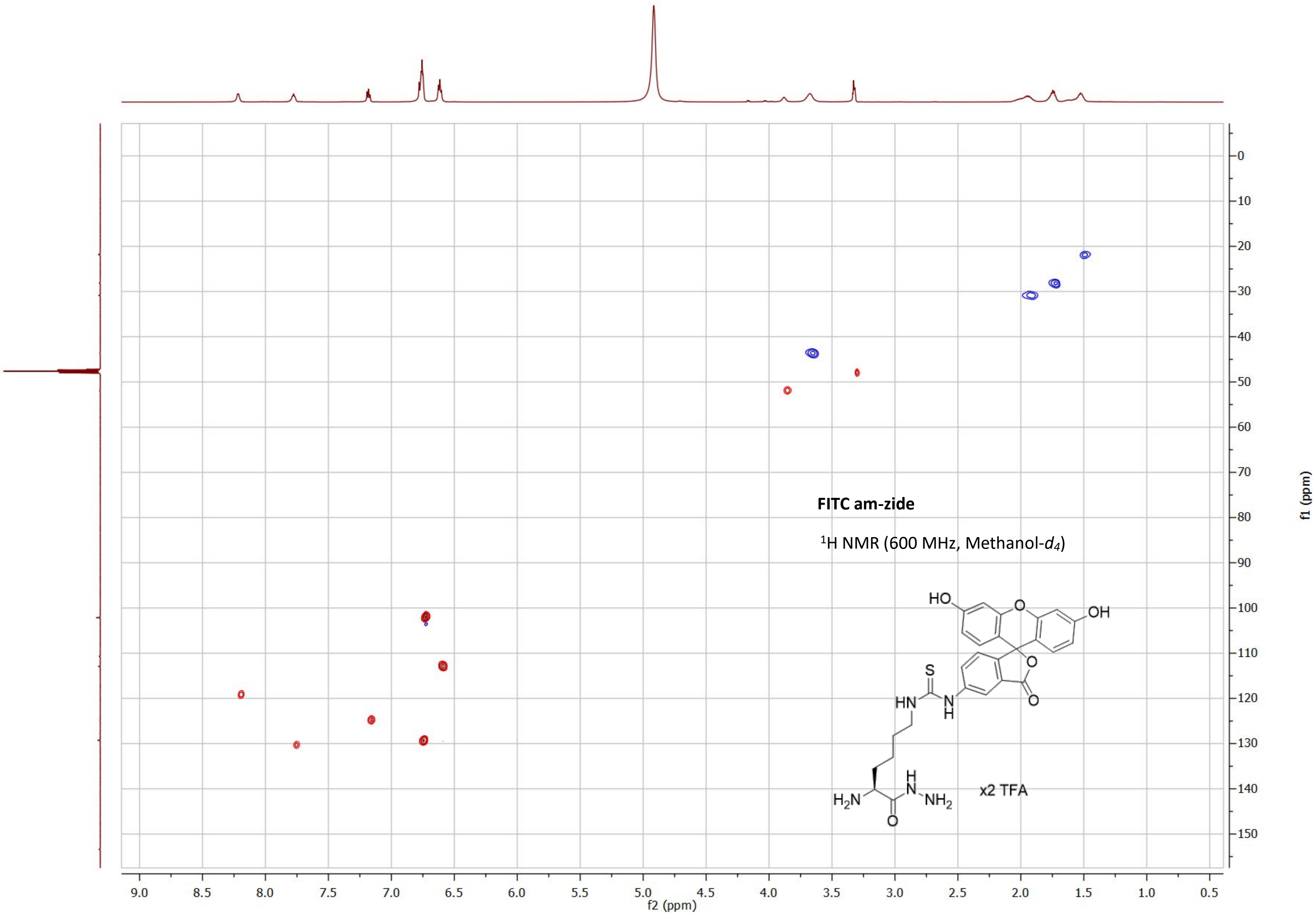
FITC am-zide
$^1$H NMR (600 MHz, Methanol-$d_4$)
HO
O
OH
O
O
S
HN
N
H
H
N
NH2
H2N
O
x2 TFA
f2 (ppm)
f1 (ppm)
9.0
8.5
8.0
7.5
7.0
6.5
6.0
5.5
5.0
4.5
4.0
3.5
3.0
2.5
2.0
1.5
1.0
0.5
0
10
20
30
40
50
60
70
80
90
100
110
120
130
140
150
